# Chronic exposure of soybean plants to nanomolar cadmium reveals specific additional high-affinity targets of cadmium toxicity

**DOI:** 10.1093/jxb/erz530

**Published:** 2019-11-24

**Authors:** Elisa Andresen, Lyudmila Lyubenova, Tomáš Hubáček, Syed Nadeem Hussain Bokhari, Šárka Matoušková, Ana Mijovilovich, Jan Rohovec, Hendrik Küpper

**Affiliations:** Czech Academy of Sciences, Biology Centre, Institute of Plant Molecular Biology, Department of Plant Biophysics and Biochemistry. Budějovice, Czech Republic; Czech Academy of Sciences, Biology Centre, Institute of Plant Molecular Biology, Department of Plant Biophysics and Biochemistry. Budějovice, Czech Republic; Czech Academy of Sciences, Biology Centre, Institute of Hydrobiology, Department of Hydrochemistry and Ecosystem Modelling, Budějovice, Czech Republic; Czech Academy of Sciences, Biology Centre, Institute of Plant Molecular Biology, Department of Plant Biophysics and Biochemistry. Budějovice, Czech Republic; Czech Academy of Sciences, Institute of Geology, Department of Geological Processes, Praha, Czech Republic; Czech Academy of Sciences, Biology Centre, Institute of Plant Molecular Biology, Department of Plant Biophysics and Biochemistry. Budějovice, Czech Republic; Czech Academy of Sciences, Institute of Geology, Department of Geological Processes, Praha, Czech Republic; Czech Academy of Sciences, Biology Centre, Institute of Plant Molecular Biology, Department of Plant Biophysics and Biochemistry. Budějovice, Czech Republic; University of South Bohemia, Faculty of Sciences, Department of Experimental Plant Biology, České Budějovice, Czech Republic; Bielefeld University, Germany

**Keywords:** Cadmium, lipidomics, metabolomics, metalloproteomics, metal stress, soybean (*Glycine max*), sublethal toxicity, XANES

## Abstract

Solving the global environmental and agricultural problem of chronic low-level cadmium (Cd) exposure requires better mechanistic understanding. Here, soybean (*Glycine max*) plants were exposed to Cd concentrations ranging from 0.5 nM (background concentration, control) to 3 µM. Plants were cultivated hydroponically under non-nodulating conditions for 10 weeks. Toxicity symptoms, net photosynthetic oxygen production and photosynthesis biophysics (chlorophyll fluorescence: Kautsky and OJIP) were measured in young mature leaves. Cd binding to proteins [metalloproteomics by HPLC-inductively coupled plasma (ICP)-MS] and Cd ligands in light-harvesting complex II (LHCII) [X-ray absorption near edge structure (XANES)], and accumulation of elements, chloropyll, and metabolites were determined in leaves after harvest. A distinct threshold concentration of toxicity onset (140 nM) was apparent in strongly decreased growth, the switch-like pattern for nutrient uptake and metal accumulation, and photosynthetic fluorescence parameters such as Φ _RE10_ (OJIP) and saturation of the net photosynthetic oxygen release rate. XANES analyses of isolated LHCII revealed that Cd was bound to nitrogen or oxygen (and not sulfur) atoms. Nutrient deficiencies caused by inhibited uptake could be due to transporter blockage by Cd ions. The changes in specific fluorescence kinetic parameters indicate electrons not being transferred from PSII to PSI. Inhibition of photosynthesis combined with inhibition of root function could explain why amino acid and carbohydrate metabolism decreased in favour of molecules involved in Cd stress tolerance (e.g. antioxidative system and detoxifying ligands).

## Teaser

Chronic, sublethal cadmium toxicity in soybean plants impacts primary and secondary metabolism by hampering photosynthesis and affecting the micronutrient nutrition status, leading to changes in metabolite and lipid composition.

## Introduction

Cadmium (Cd) is an important environmental pollutant and toxic to most organisms. Concentrations in the Earth’s crust are low (0.1–0.5 mg kg^–1^; Maret and Moulis, 2013), but anthropogenic activities such as mining, smelting, and industrial use keep increasing the Cd concentration in the environment (reviewed by Küpper and Andresen, 2016). Cd is highly water soluble and readily taken up by plants, leading to decreased growth and seed production of the plants, thereby reducing their yield and nutritional quality, and posing a threat for human nutrition (McLaughlin *et al.*, 1999).

Cd toxicity in plants may interfere with all parts of their metabolism (primary and secondary), but in many cases the relevance and interdependence of toxicity mechanisms is not clear (reviewed by Andresen and Küpper, 2013; Küpper and Andresen, 2016). Cd detoxification strategies include chelation by different ligands, immobilization, exclusion, and compartmentalization (Benavides *et al.*, 2005; Hasan *et al.*, 2009; Küpper and Leitenmaier, 2013).

Cd^2+^ ions generally have a high affinity for thiol groups (Dudev and Lim, 2014), so that during toxicity they tend to bind to proteins with Cys residues. This can lead to their misfolding, inhibition of enzymes, and inhibition of redox enzymatic regulation (Hall, 2002), with further consequences on carbon (C), sulfur (S), and nitrogen (N) metabolism. Due to its physical and chemical similarity to zinc (Zn; both belong to the group II transition metals), Cd can replace Zn in proteins and enzymes, making them non-functional (Clemens, 2006; Maret and Moulis, 2013; Dudev and Lim, 2014). In addition, Cd can also occupy binding sites for calcium (Ca) with consequences for Ca signalling (Perfus-Barbeoch *et al.*, 2002) or replace other divalent ions, such as magnesium (Mg) in Rubisco (Polatajko *et al.*, 2011) or the chlorophylls (Chls; reviewed by Küpper *et al.*, 2006). In the latter case, the substitution of Mg generates [Cd]–Chl, which is unsuitable for photosynthesis and therefore a great threat for photosynthetic organisms.

In the aquatic model plant *Ceratophyllum demersum*, the incorporation of Cd in light-harvesting complex II (LHCII), probably by formation of [Cd]–Chl, occurred already at very low Cd concentrations of only 5 nM, while strong decreases in photosynthesis (measured as *F*_v_/*F*_m_, Φ _PSII_, and oxygen production) occurred from 20 nM onwards (Andresen *et al.*, 2016). All negative effects were more pronounced under high-light conditions, emphasizing the buffering effect of a large antenna system with many more binding sites for Cd.

This study aimed at investigating to what extent the binding of Cd to the LHCs also occurs in land plants and to what extent it contributes to the mechanism of sublethal toxicity at nanomolar Cd concentrations in soybean (*Glycine max*), an agriculturally important plant.

## Materials and methods

### Pre-treatment

All glassware and plastic were thoroughly acid washed and rinsed with ultrapure water (double distilled after reverse osmosis and filtration; roddH_2_O) before use.

### Plant material and growth conditions

Soybean seeds (*Glycine max*, var. Erin, non-nodulating) obtained from the botanical garden in Konstanz, Germany, were soaked in ddH_2_O and then incubated in a glass Petri dish at room light and room temperature. Seeds were soaked daily. When the germination root was visible, the germinating seeds were placed on roddH_2_O-washed and moist perlite in a growth chamber (Microclima MC1000E, Sniders Scientific B.V., Tilburg, The Netherlands). Light and temperature followed a sinusoidal cycle from 18 °C to 25 °C and a maximum of 150 µmol photons m^–2^ s^–1^ with 16 h day/8 h night. Humidity was not determined. When two real leaves were developed (usually after 10 d), the seedlings were transferred to hydroponic cultures, with four plants per pot for each treatment condition. The hydroponic cultures were continuously aerated with filtered room air. The nutrient solution was supplied from stock barrels with a flow rate of 0.42 ml min^–1^, which was increased to 0.65 ml min^–1^ after 4 weeks when the biomass had increased.

The nutrient solution was half-strength HHNS (hyperaccumulator hydroponic nutrient solution) in de-ionized water (Küpper *et al.*, 2007*a*; pH 5.7 with KOH). Inductively coupled plasma mass spectrometry (ICP-MS) measurements revealed that even the control treatment, with no added Cd, had a concentration of 0.5 nM due to the inevitable Cd contamination of all commercially available chemicals. Thus the real Cd concentrations in the nutrient solutions were: 0.5±0.2 (control), 10±0.7, 20±1.3, 50±2.5, 140±12, 270±22, 550±33, 1270±86, 3100± 440, and 27 500±1900 nM. Four independent experiments were carried out, each with four individual plants per treatment condition.

### Oxygen exchange

In week 5 (w5) and 10 (w10) of the treatment duration, oxygen exchange was measured on leaf discs using an LD2/3 electrode chamber (Hansatech Instruments Ltd, Norfolk, UK) linked to a temperature-controlled circulating water bath kept at 25 °C. The electrode was calibrated as described in the manual by David Walker (Walker, 1990). The sandwich contained 200 µl of 1 M NaHCO_3_^–^ buffer and 100–200 µl of roddH_2_O (depending on the size of the disc). Oxygen consumption was determined in the dark and oxygen production with increasing light intensities from 9 µmol photons m^–2^ s^–1^ to 610 µmol photons m^–2^ s^–1^ using a slide projector. Irradiance was adjusted by neutral density filters (AHF Analysentechnik, Tübingen, Germany). Data were recorded with the OxyCorder measuring device with the software Oxywin 3.1 (Photon Systems Instruments, Brno, Czech Republic) and analysed using Origin 2015 (OriginLab Corporation, Northampton, MA, USA).

### Fluorescence kinetic microscopy (FKM) measurement

#### Quenching analysis in the millisecond–minute time range

In w5 and w10 of the metal exposure, Chl fluorescence kinetics were determined spatially (*n*=7) and spectrally (*n*=4) in young mature leaves (if available). The leaves were mounted in the measuring chamber, covered with a slightly moist cotton pad and a gas-permeable mesh, and aerated by water-saturated, filtered room air (Küpper *et al.*, 2007*a*). After the measurement, leaves were cut in half and frozen for metal and pigment determination. Analyses of the fluorescence data were done according to Küpper *et al.* (2007*a*) and Mishra *et al.* (2014). From each image, three representative areas each were selected in the mesophyll far away from veins and on top of veins, respectively, analysed individually, and then the calculated parameters were averaged.

#### OJIP fluorescence induction

OJIP fluorescence induction was measured using a newly developed instrument (Küpper *et al.*, 2019*a*). For exciting Chl fluorescence, an excitation filter with transmission from 420 nm to 500 nm (Semrock HQ 460/80, IDEX Corporation, Lake Forest, IL, USA) was used. For separating PSII-related fluorescence from exciting light, a dichroic mirror with a 505 nm edge wavelength (Semrock HC Beamsplitter BS 506, IDEX Corporation) in a second filter wheel and an emission filter with 672–698 nm transmission (Semrock 684/24 BrightLine HC, IDEX Corporation) in a third filter wheel was chosen. After focusing a suitable position on the leaf using the ×25 objective, it was acclimated to darkness for 5 min and the fluorescence kinetics were measured. Fluorescence emission filters for the same wavelengths as in the other FKM were used.

### Harvest

The plants exposed to the highest Cd concentration (2.7 µM) were practically dead and were harvested after 5 weeks; all others were harvested after 10 weeks when the seeds of the control plants were mature. Fresh weight of leaves, stems, and seed pods was determined individually for each of the four plants of every pot. The intertwined roots of all four plants per pot were washed in roddH_2_O, dried on cellulose paper, and weighed. All samples were frozen in polypropylene tubes in liquid nitrogen and stored at –80 °C.

### Protein isolation/LHCII details

Proteins were isolated from the harvested biomass (biomass of harvested leaves, *n*=3; biomass of leaves at w5, *n*=2) as described in Andresen *et al.* (2016) with the following changes: isolation and solubilization buffer had been treated with Chelex-100 (batch method, according to the manufactor’s protocol, BioRad, Hercules, CA, USA) to remove metals. Protein separation was accomplished by size exclusion chromatography using two coupled glass columns (Superose 6 10-300 and Superose 12 10-300, GE Healthcare, USA) with an elution time of 150 min. Vitamin B_12_ was added to the samples prior to injection in a final concentration of 0.05 mg ml^–1^ to be able to normalize the elemental counts from the ICP-MS to cobalt (Co) as internal standard. The delay time between the diode array detector and ICP-MS was re-calculated manually using the signal from Co and the visible (VIS) absorption signal of the vitamin B_12_ peak from the standard. Selected fractions were collected and identified by the proteomics centre of Madrid University as described in Supplementary (Protocol S1 at *JXB* online).

### XANES measurements of isolated LHCII

To analyse LHCII with X-ray absorption near edge structure (XANES) measurements (see below), two independent protein isolations were performed from pooled leaf biomass exposed to 10, 20, and 50 nM Cd. (i) 25 g FW, the following sequence of columns: Superdex 75 XK 26 1000–Fractogel DEAE 650 XK 16 350; and (ii) 21 g FW, Superdex 75 XK 26 1000–Mono Q HR 5/5–Superdex 30 Superformance columns. The buffers used with the Mono Q and Fractogel columns were as follows: eluent A, 0.15 M NH_4_HCO_3_ pH 7.8 + 0.2 mM *n*-dodecyl-β-maltoside (DDM); eluent B: 0.15 M NH_4_HCO_3_ pH 7.8 + 0.2 mM DDM+1 M NaCl. On the MonoQ column, after washing out unbound sample with three column volumes (cvs), a gradient of 40 cv, from 0 to 100% eluent B was run. After washing out unbound sample with 2 cvs, a gradient of 60 cvs from 0 to 100% eluent B was run on the Fractogel column. The Superdex columns were run only with eluent A (isocratic) for at least 1.5 cv (i.e. until beyond the lower molecular weight limit). Samples for synchrotron experiments were in buffer A (see above). Samples were pipetted into 1 mm diameter polyimide capillaries and shock-frozen in isopentane cooled in a liquid N_2_ bath. As a reference compound, Cd aquo complex (5 mM CdSO_4_ in water) mixed with 20% glycerol was measured.

XANES measurements were performed at the XAS beamline P64 at the synchrotron radiation source PETRA III (DESY, Hamburg, Germany) (Caliebe *et al.*, 2019). The beamline was equipped with a Si(331) monochromator. The beam energy was 6 GeV. The seventh harmonic of the undulator was used without detuning. The beam size was 0.2 mm (vertical)×0.5 mm (horizontal). The incoming beam intensity was monitored by an ionization chamber filled with 70% N and 30% krypton (Kr), and the intensity after the sample was monitored with an ionization chamber filled with 100% Kr. Samples were mounted in a helium exchange gas cryostat (Janis) and kept at temperatures in the range 5–12 K. Spectra were collected in fluorescence mode with a 100 pixel high-purity germanium (Ge) detector (Canberra GmbH). XANES was collected in fluorescence mode for an energy range (–200 eV to +200 eV) with respect to the calibrated edge at 26 711 eV, with an integration time of 2 s for the Cd aquo model and 10 s for the proteins. Calibration was performed with a Cd foil, setting the fluorescence peak values to the tabulated energies of the K_α_ (23 173 eV) and K_β_ (26 095 eV) peaks. The acquisition and calibration software were a set of Python routines developed at the P64 beamline. The data of each detector pixel were controlled for glitches before averaging. Data were reduced and normalized using ATHENA (Ravel *et al.*, 2005) by fitting splines to the pre-edge and after-edge regions. The data of Cd histidine and Cd glutathione from Küpper *et al.* (2004) were used for comparison. Those reference models were measured at the bending magnet beamline D2 EMBL at DORIS (DESY) which was equipped with a Si(111) monochromator and a 13 element Ge detector.

### Determination of elements in the nutrient solution

Trace element concentrations in the nutrient solutions were determined using an ICP sector field mass spectrometer ICP-MS Element 2 (Thermo Fisher Scientific, Prague, Czech Republic). Measurements were performed as described in the supporting information of Andresen *et al.* (2016).

### Inductively coupled plasma optical emission spectrometry (ICP- OES) of seeds, stems, and w5 leaves

Freeze-dried plant material was digested as described by Zhao *et al.* (1994) with the modifications of Andresen *et al.* (2013). Digested plant material was diluted 28 times with roddH_2_O and macronutrients and trace element concentrations [Ca, cadmium (Cd), copper (Cu), iron (Fe), potassium (K), Mg, manganese (Mn), sodium (Na), nickel (Ni), phosphorus (P), S, and Zn] were determined by the ICP-OES technique on a 5100 ICP-OES Agilent spectrometer at the Institute of Geology ASCR in Prague, Czech Republic. Standard operating conditions according to the manufacturer’s recommendation were used (concentric nebulizer, radiofrequency power 1100 W, sample uptake 2.5 ml min^–1^). The instrument was calibrated using aqueous multielement calibration solutions freshly admixed from commercially available single elemental standards (Astasol, Analytika s.r.o., 1000 ppm of each element). QC/QA procedures covered the stability of the instrument in the course of measurement, followed by a secondary standard inserted after each 10 samples, as well as repeated measurement of commercial multielement reference material (ANM012, Analytika s.r.o., three times in the analytical run).

### ICP-MS of leaves (w10, harvest) and roots

The previous procedure (see above) was further optimized as follows. A mixture of 85 ml/100 ml of 70% HClO_4_ (Suprapur^®^ grade, Carl Roth, Karlsruhe, Germany and 15 ml/100 ml of 69% HNO_3_ (Ultrapur^®^ grade, Carl Roth) was added to the plant material in glass tubes following the protocol of Zhao *et al.* (1994). After at least 30 min pre-digestion at room temperature, the glass tubes were uniformly heated using a Fuji PXG4 Thermoblock (AHF Analysentechnik AG, Germany). The heating program was optimized for uniform heating of the digestion mixture. It ramped the temperature of the acid mixture to 220 °C within 4 h. The acid mixture was then heated at 220 °C for another hour to dry the digest from acid contents. The digest was then cooled to room temperature and 0.5 ml of 5% HCl (Ultrapur^®^ grade, Carl Roth) was added to each test tube. With a program, glass tubes were heated to 90 °C for an hour to obtain clear solutions. The final volume was made to 1.5 ml with ddH_2_O and stored in 2 ml Eppendorf vials. Trace metals were quantified using a sector field ICP-mass spectrometer Element II (Thermo Fisher Scientific, USA). The conditions for the ICP-MS were set up according to the manufacturer’s recommendation [double pass spray chamber, PFA (perfluoroalkoxy) concentric nebulizer with a flow rate of 50 µl s^–1^, Ni cones, 1200 W radiofrequency power, sample time 1 min] and it was optimally tuned to achieve the highest sensitivity and acceptably low oxide formation using multielement standard solution with a concentration of 1 µg l^–1^ of each element. The instrumental calibration was performed using a multielement calibration solution by commercially available single elemental standards (EPOND), and ^115^In was used as an internal standard for the correction of the instrumental drift. The quantification was performed in medium resolution to avoid potential interferences.

### Determination of chlorophyll content

The leaves from the FKM measurements as well as mixed leaf biomass after the harvest were used for Chl determination. Pigments were extracted in 100% acetone as described in Andresen *et al.* (2016). Spectra were measured with the UV/VIS/near-IR absorption spectrophotometer Lambda 750 (Perkin-Elmer, Waltham, MA, USA) at a spectral bandwidth of 0.5 nm, with a 0.5 nm sampling interval from 330 nm to 750 nm. Pigment composition was analysed using the Gauss Peak Spectra method of Küpper *et al.* (2000, 2007*b*) with focus on Chls from 550 nm to 750 nm. Additionally, pigment extracts were measured by HPLC-ICP-MS as described in Küpper *et al.* (2019*b*).

### Metabolomics

Frozen biomass from leaves of the plants exposed to non-inhibitory (0.5, 20, and 50 nM), and sublethally toxic (140 nM and 270 nM) Cd concentrations were taken for analyses of metabolites and lipids. Extraction and analyses were done by the Metabolomic facilities at the University of Copenhagen, Denmark. A detailed description can be found in the Supplementary Protocols S2 and S3. Starch analyses were done as established for *C. demersum* (Thomas *et al.*, 2013).

### Statistics

Tests for normality and two- and three-way ANOVA were performed in SIGMAPLOT 12 (Systat Software Inc., San Jose, CA, USA) at a significance level of *P*<0.05 for Cd concentration and weeks of exposure for the w5 and w10 measured data, and Cd concentration, tissue, and individual experiment for the data obtained from the harvested material. In the case of significant effects, the Holm–Sidak method was used for an all-pairwise post-hoc multiple comparison. Tests for linear trends were done as linear regression in Origin 2015 (OriginLab Corporation, Northampton, MA, USA). The probability that the slope is zero is given as ‘Prob>*F*’ and Pearsson’s correlation coefficient as *R*^2^.

## Results

In the following, the results are presented grouped by subject area. In general, all described differences were statistically significant as tested with ANOVAs (see the Materials and methods) and with *P*<0.05, unless described otherwise. Details of the statistical test results can be found in Supplementary Table S5.

### Toxicity symptoms and biomass

**Fig. 1.**
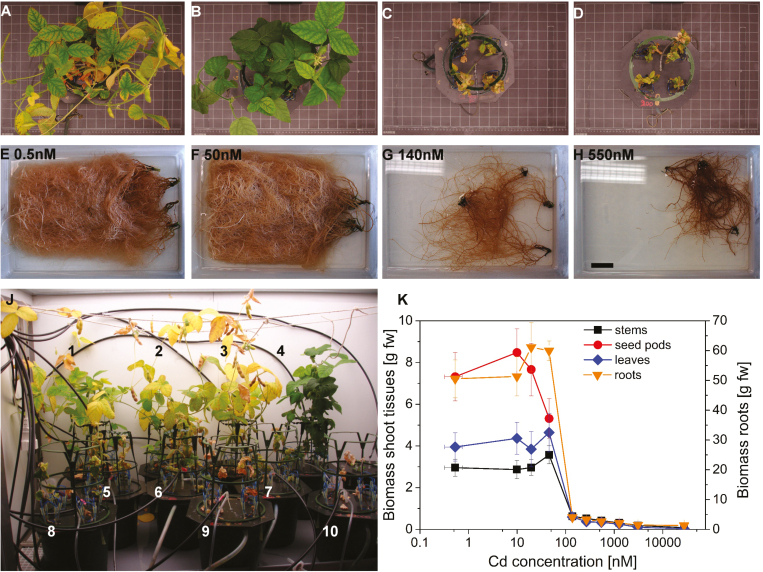
Visible toxicity symptoms and biomass production of soybean plants after 10 weeks of exposure to various Cd concentrations. (A and E) 0.5 nM (control), (B and F) 50 nM, (C and G) 140 nM, (D and H) 550 nM, (J) arrangement of the plants in the phytochamber with numbers representing the increasing Cd concentration [0.5±0.2 (control), 10±0.7, 20±1.3, 50±2.5, 140±12, 270±22, 550±33, 1270±86, 3100±440, and 27 500±1900 nM]. Photos are examples from one of four experiments. (K) Biomass of harvested plant material. Values are the mean of four experiments, and error bars represent the SE.

Exposure to Cd led to visible toxicity symptoms and decreased biomass production by the plants (Fig. 1). Necrosis and cessation of growth occurred at high Cd (1300–27 500 nM=1.3–27 µM) already after 1 week of exposure. With prolonged exposure time, toxicity symptoms were gradually observed down to the threshold concentration of 140 nM Cd (Fig. 1C, G), with a clear decrease in growth and no development of flowers, seed pods, and side roots. The leaves of those plants and all higher concentrations were reddish and crispy, easily breakable, and the plants developed only a few new leaves after the start of Cd exposure (Fig. 1C, D). Thus, ‘old’ or ‘young’ instead of ‘young mature’ leaves had to be taken for the measurements. The roots of the low Cd group were white/yellow, while the high Cd group roots had a brownish/reddish colour. Plants exposed to the highest concentration of 27 µM died after 3–4 weeks of exposure. The plants exposed to 50 nM Cd had the highest biomass of leaves and stems (therefore this concentration was still regarded as ‘non-inhibitory’), but had distorted flowers and the lowest biomass for seed pods (Fig. 1B, F). Significant differences occurred between the two groups of the non-inhibitory (control; 50 nM) and toxic to lethal (140–1300 nM) concentration (*P*<0.001, df=9 for Cd concentration; *P*<0.001, df=3 for tissue type).

### Nutrient uptake and metal accumulation

**Fig. 2.**
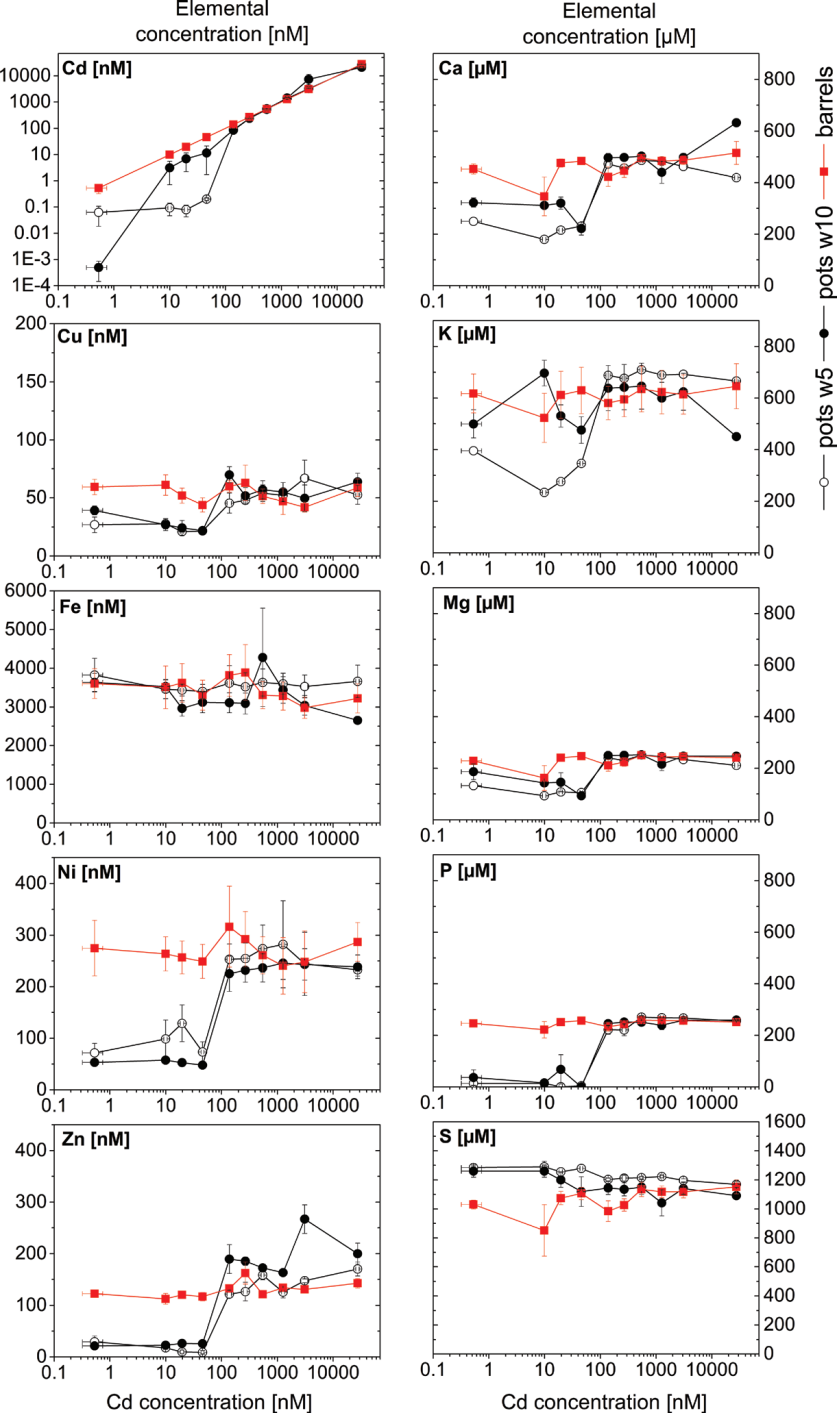
Concentration of micro- and macronutrients in the hydroponic nutrient solution in the barrels (*n*=4) and in the pots after 5 (*n*=2) and 10 (*n*=4) weeks of treatment duration. Values are the mean of four experiments, and error bars represent the SE.

**Fig. 3.**
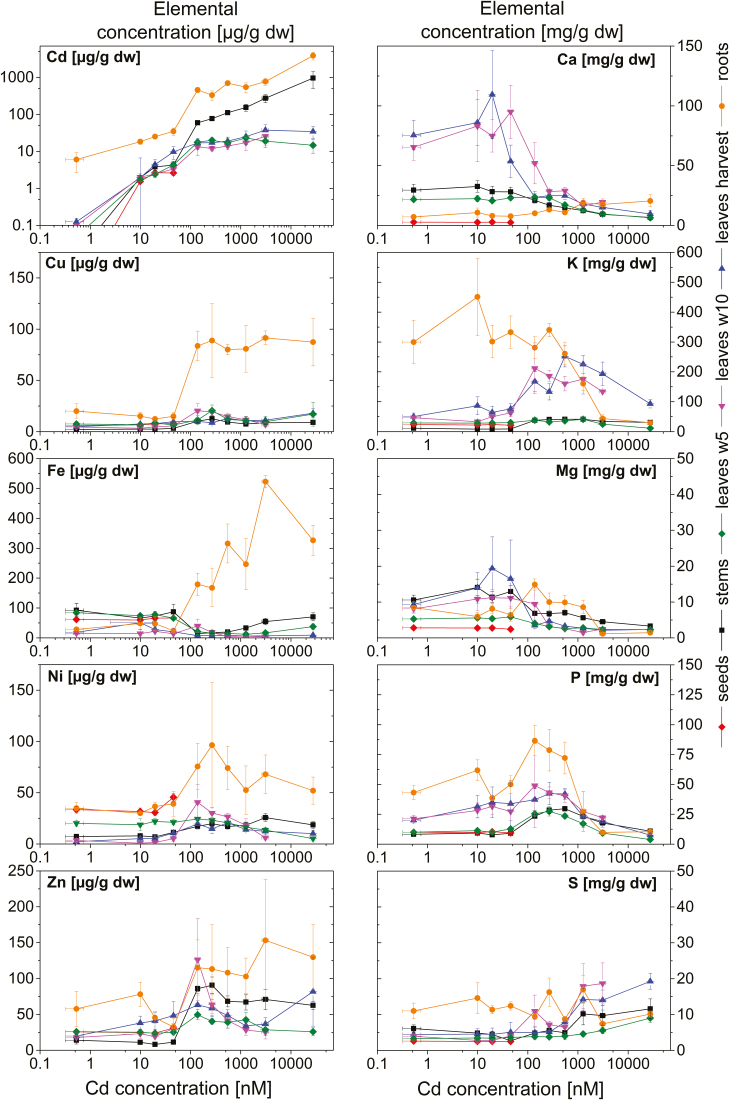
Element accumulation of micro- and macronutrients in different tissues of plants after 5 weeks (leaves only) and 10 weeks of exposure to various Cd concentrations. Values are the mean from four independent experiments and up to four individual plants per measurement (*n*=16) for harvest leaves, stems, and seeds. w5 and w10 leaves *n*=8. Error bars represent the SE.

The concentrations of micro- and macronutrients in the barrels were rather stable throughout all applied Cd concentrations, showing that no problem of precipitation occurred (Fig. 2). In the pots, concentrations represent the equilibrium between inflow from the barrel and outflow after the plants have taken up some of the nutrients. A clear trend was visible for most elements in the pots. For the low and sublethal Cd concentrations (up to 140 nM), the concentration of Cd, Cu, Ni, Zn, P, Ca, and K was much lower in the pots compared with the barrels in w5, indicating nutrient removal by the plants. From the threshold concentration of 140 nM Cd onwards, the depletion of the nutrients in the pots was less (Fig. 2), in accordance with the drastically decreased root and shoot biomass of those plants (Fig. 1). Significant differences occurred between the group of low Cd (0.5–50 nM) and higher (140–27 500 nM) Cd for P and Zn, and also between the barrels versus pots w5 (Cu, Ni, P, and Zn), and barrels versus pots w10 for Cu, Ni, and P. Higher concentrations of Zn were detected in the pots of w10 compared with the barrels for the higher and lethal Cd concentrations (from 140 nM onwards, ~50% higher than in the barrels, significantly different from the Zn concentrations in the 0.5–50 nM Cd pots; *P*<0.001). This indicates that the stressed plants actually released previously stored Zn, as also visible in the root tissue (Fig. 3).

Exposure to Cd affected the accumulation of all macro- and micronutrients in the tissues (*P*<0.001 for all except Fe: *P*=0.026). All micronutrients accumulated to a significantly higher extent in the roots than in the above-ground tissues with increasing applied Cd concentration (Fig. 3). Accumulation of Cd followed the uptake route: highest in roots, then stems, and followed by leaves, with higher values after longer exposure time (w10 and harvest), and was least in seeds. At the higher Cd concentrations, a plateau occurred for Cd accumulation in the leaves, indicating a maximum of uptake or transport, probably in the sense of a limit where intracellular concentrations become lethal. A tremendous increase was observed in the roots of the plants exposed to the threshold concentration of 140 nM. While exposure to 50 nM led to root Cd accumulation of 35 µg g^–1^ DW, roots treated with 140 nM had >10 times higher Cd accumulated (460 µg g^–1^ DW). Accumulation of Cu, Ni, and Zn increased in all shoot tissue types with increasing Cd, up to a peak in the plants exposed to the sublethally toxic Cd concentrations (140–550 nM) and a decrease towards lethally toxic Cd (>550 nM). A converse trend was found for Fe content, with a decrease in the leaves (w5 and harvest) and stems from the non-toxic to the sublethally toxic 140 nM Cd, and then an increase towards lethally toxic Cd.

Accumulation of Zn in the roots decreased slightly from the control towards 50 nM Cd, but increased drastically at 140 nM and then remained constant until 27 000 nM Cd. Fe in the roots remained about constant until 50 nM, and then increased continuously until 3100 nM Cd before it decreased again at 27 000 nM Cd, the latter probably due to disintegration of tissue in the dying roots.

Regarding the macronutrients, Ca increased slightly in response to Cd toxicity in the roots, but decreased in leaves. Towards toxic Cd, S and K levels increased in the shoot tissues, but not in the roots. Drastic leakage of K from the roots above 140 nM Cd, of P from 270 nM Cd, and of Mg from 1000 nM Cd corresponded to the lethal character of these treatments.

Calculating the nutrient uptake efficiency (NUE) and the efficiency index (comparing the stressed plants with the control plant, Siddiqi and Glass (1981) showed clearly that from the threshold concentration of 140 nM, Cd-stressed plants could no longer utilize the nutrients (Supplementary Fig. S1).

### Oxygen production and chlorophylls

**Fig. 4.**
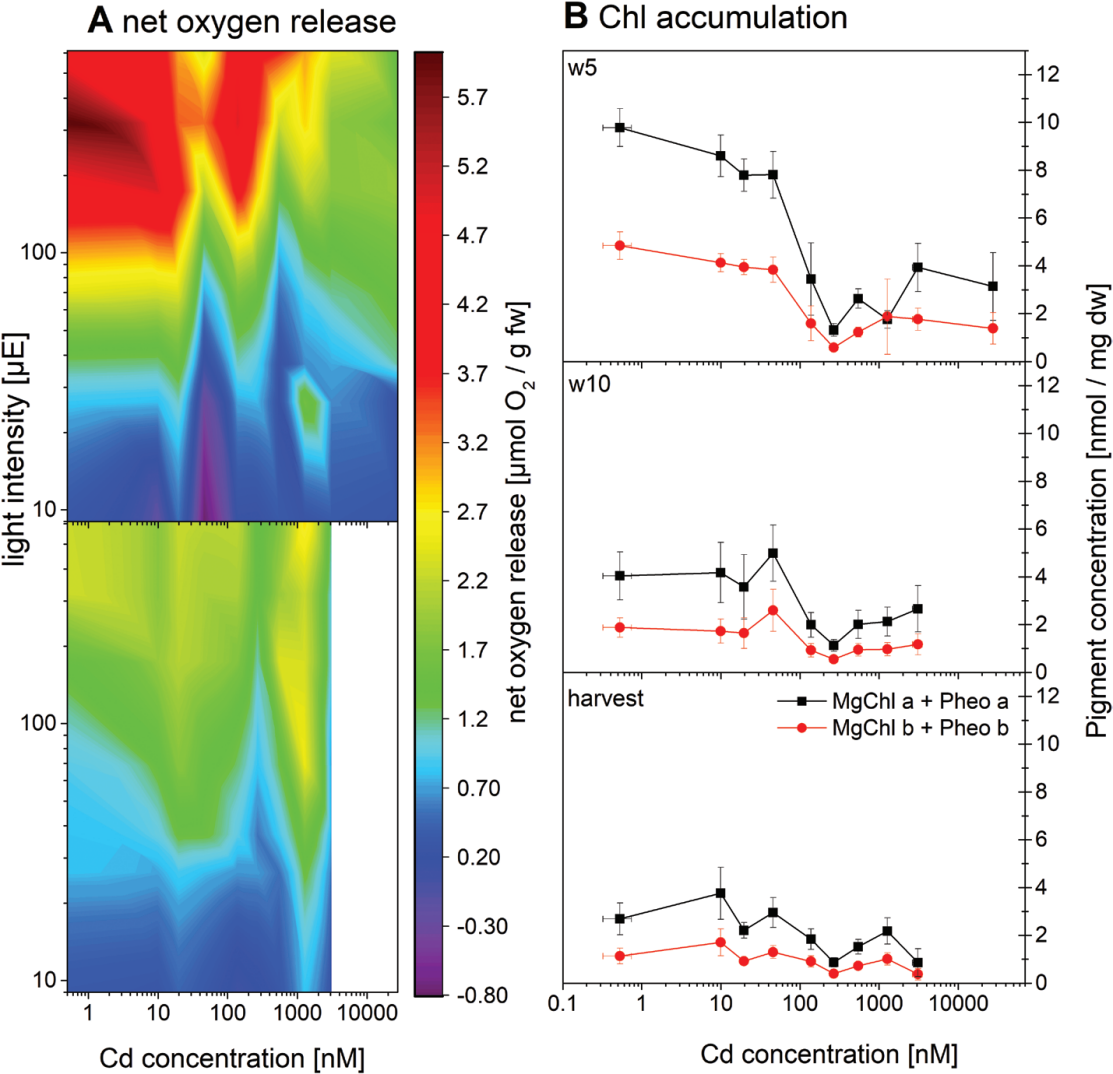
A: Net photosynthetic oxygen release of soybean leaves measured as leaf disks with increasing light intensities after 5 and 10 weeks of Cd exposure. Values are the mean values from two experiments ±SE. (B) Chlorophyll and pheophytin *a* and *b* in individual leaves (taken from the FKM measurement) after 5 and 10 weeks of Cd treatment and of the harvested, homogenized leaf material. Values are means from three experiments, and error bars represent the SE.

The net photosynthetic oxygen release rate (PS rate) was highest in the control plants in w5 at moderate irradiance (Fig. 4A). At the highest irradiance, even the control plants became slightly photoinhibited. No Cd-treated plant reached the same levels of oxygen production, showing that even very low Cd concentrations which did not yet inhibit growth already diminished photosynthesis. A drastic decrease of the net PS rate occurred for plants exposed to ≥270 nM. In w10, the PS rate generally decreased drastically, since plants entered senescence due to seed ripening at that time. Due to this physiological change, the PS rate was only dependent on the light (*P*<0.001), and not on the Cd concentration (*P*=0.073).

From w5 to w10, the Chl concentration of the control leaves decreased by 50%, reflecting the onset of senescence with seed ripening. The effect of Cd on Chl *a* was more pronounced than that on Chl *b* in w5 and w10 (Fig. 4B). Yet again, there was a clear drop to the threshold concentration of 140 nM, especially in w5 (50 nM versus 140 nM for Chl *a P*<0.001, Chl *b P*=0.025). The slight increase towards higher Cd is likely to be due to the measurement on older leaves (because no other leaves remained alive on these plants), which had more Chl compared with the young mature leaves (see images in Fig. 1). The differences were significant for all applied Cd concentrations (*P*<0.001, df=9). No distinct [Cd]–Chl was detected (Supplementary Fig. S2).

### Chlorophyll fluorescence kinetics

**Fig. 5.**
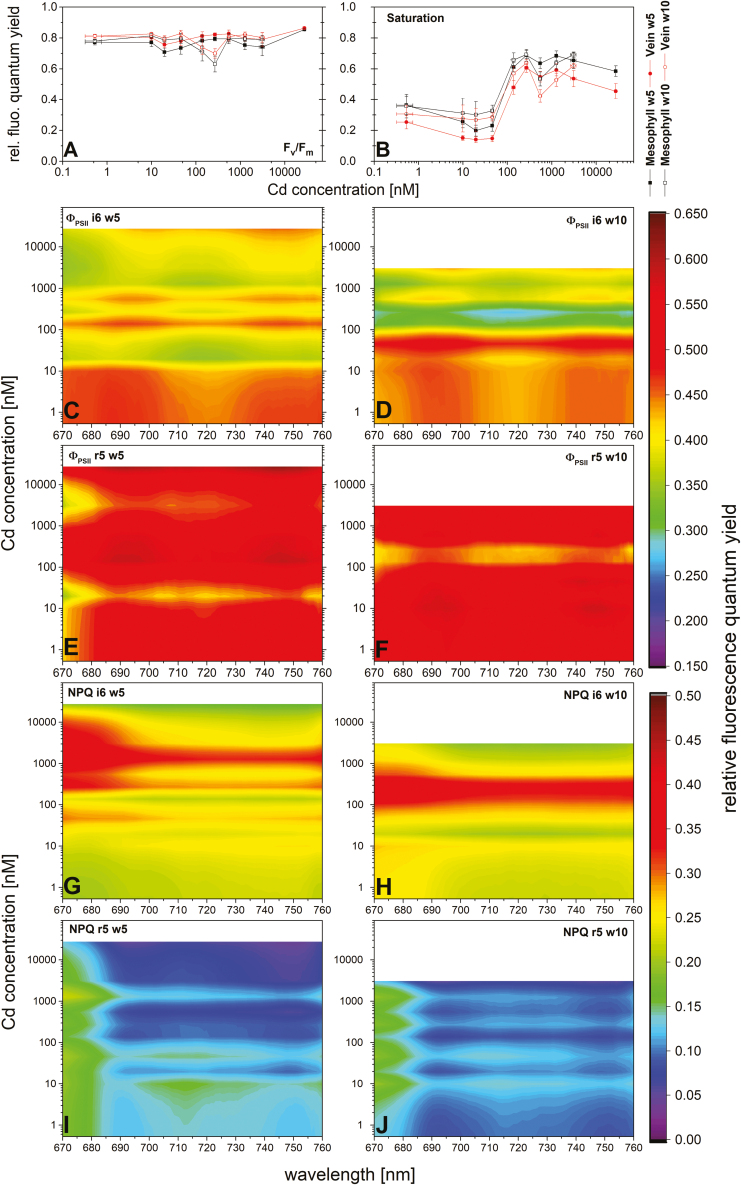
Chlorophyll fluorescence parameters obtained by measurements of ms–min time range Kautsky induction of leaves exposed to various Cd concentrations for 5 and 10 weeks, spatially and spectrally resolved. (A) *F*_v_/*F*_m_=(*F*_m_–*F*_0_)/*F*_m_, a measure for the maximum efficiency of PSII in the dark-adapted state. (B) Saturation=(*F*_p_–*F*_0_)/(*F*_m_–*F*_0_) spatially resolved. Values are the mean of four experiments and 1–2 individual leaves (*n*=7), and error bars represent the SE. (C–J) Spectrally resolved parameters of the effective quantum yield of photochemical energy conversion (Φ _PSII_) (C–F) and non-photochemical quenching (NPQ) (G–J), both after onset (200 s) of actinic light (i6), (C, D, G, H) and darkness (r5) (E, F, I, J) Values are the mean of four experiments.

The maximal photochemical quantum efficiency of the PSII reaction centres (measured as *F*_v_/*F*_m_) was surprisingly little affected by Cd stress (Fig. 5A). This parameter is often used as an indicator of metal stress. However, even at the toxic Cd concentrations, *F*_v_/*F*_m_ did not decrease significantly. A clear induction shape, however, was observable for the parameter ‘saturation’ (*F*_p_–*F*_0_)/(*F*_m_–*F*_0_) (Fig. 5B) (defined by Küpper *et al.*, 2007*a*). Significant differences (*P*<0.001) existed between the two groups of low (0.5–50 nM) and higher (140–27 500 nM) for the mesophyll and the veins. For the veins, a few additional differences were found even within the high Cd group.

The activity of PSII in actinic light (Φ _PSII_) was diminished already above 10 nM after 5 weeks, but no pronounced differences were observed between spectral regions within the range of *in vivo* Chl fluorescence (Fig. 5C). After 10 weeks, the threshold for inhibition shifted to 50 nM (Fig. 5D), corresponding to the still rather healthy plant exposed to 50 nM and the stressed plant exposed to 140 nM (see Fig. 1). After 200 s acclimation to darkness (Φ _PSII__r5), there was hardly any Cd-induced difference in the PSII activity (Fig. 5E, F). Similar results were observed for non-photochemical quenching (NPQ; Fig. 5G–J). While there was no Cd-related effect observable after 200 s of dark relaxation (r5), during the actinic light phase (i6), Cd toxicity led to a marked increase of NPQ, and the peak of NPQ was shifted towards shorter wavelengths, where the outer LHC antennae emit fluorescence.

**Fig. 6.**
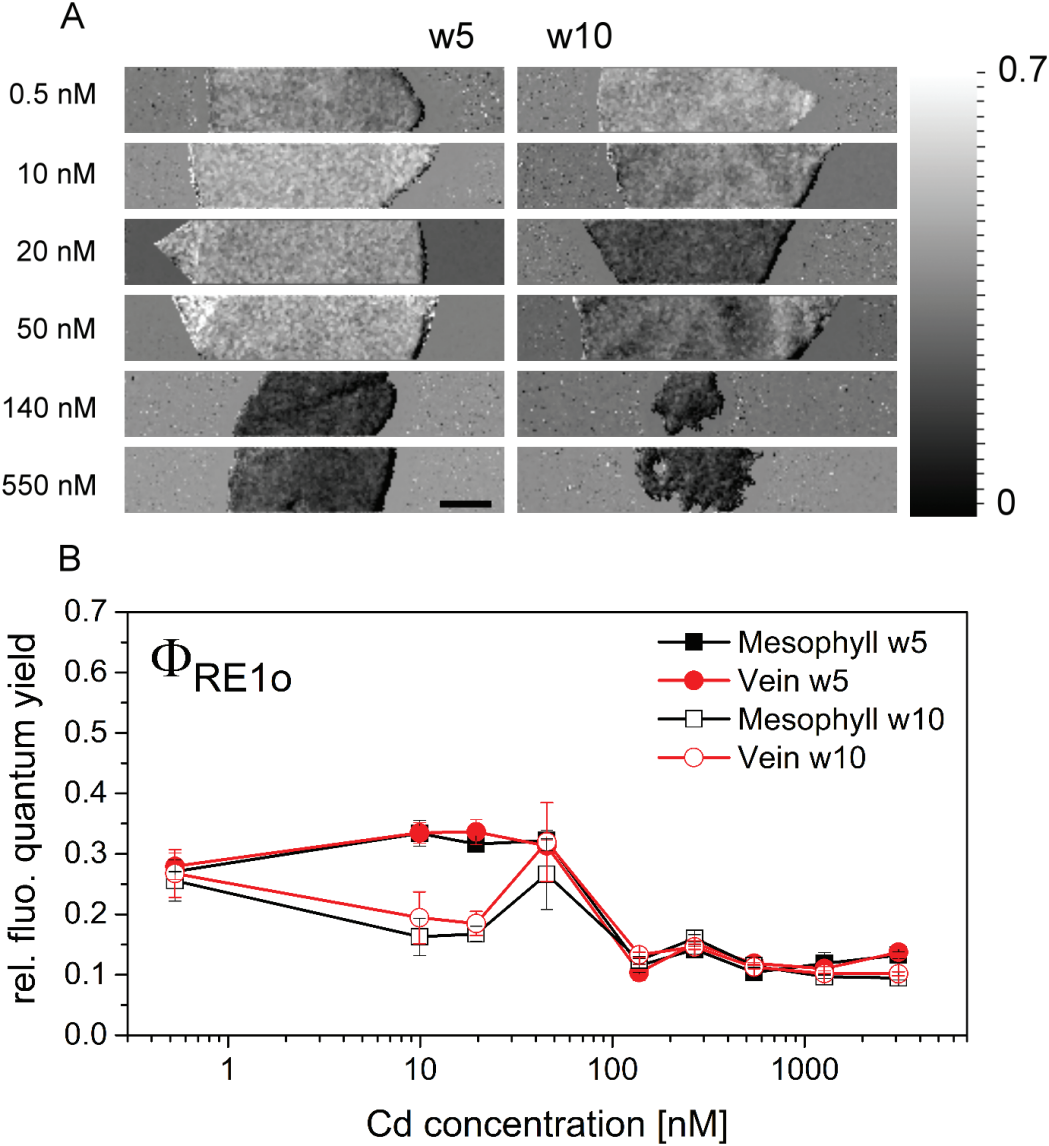
OJIP-derived parameter Φ _RE1o_, a measure for the quantum yield with which a PSII trapped electron is transferred to PSI acceptors. (A) Macroscopic images from plants exposed to various Cd concentrations for 5 and 10 weeks. (B) Derived parameter Φ _RE1o_ for mesophyll and vein after 5 and 10 weeks of treatment. Values are the mean from 2–3 leaves of at least two individual plants from one experiment. Error bars represent the SE. Scale bar=1 cm.

The efficiency with which a PSII-trapped electron is transferred from PSII to the first PSI acceptor (Φ _RE1o_) was diminished in the plants exposed to the lower, sublethal Cd concentrations (Fig. 6B), with an additional different behaviour in w5 compared with w10 (*P*<0.001, *P*=0.359, and *P*<0.001 for Cd concentration, tissue, and week). The images depicting Φ _RE1o_ (Fig. 6A) showed seemingly lower fluorescence values in the veins of the plants exposed to higher Cd (140 nM) for 5 weeks and, vice versa, lower fluorescence values within the veins in the plants exposed to lower Cd for 10 weeks, but the negative effects of Cd exposure were apparently not different enough in both tissues to be detected in the measured parameters.

### Metabolomics

**Fig. 7.**
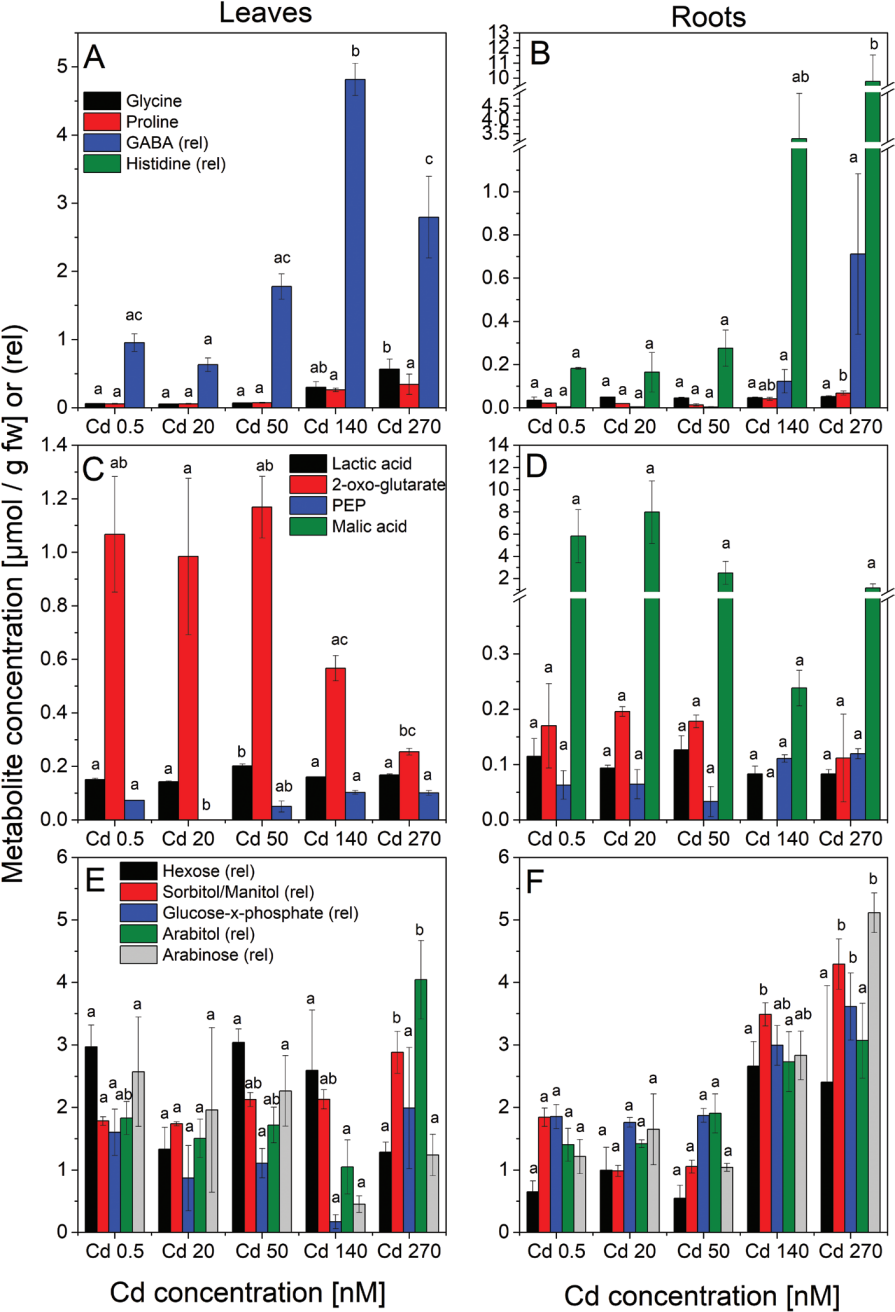
Selected metabolites in leaves (A, C, E) and roots (B, D, F) of soybean plants exposed to various Cd concentrations. Values are the mean from three experiments, and error bars represent the SE. (A and B) Amino acids and ligands; (C and D) ligands involved in the TCA cycle; (E and F) sugars and carbohydrates. Significant differences are depicted as different letters in the graphs.

For most analysed metabolites (Fig. 7; Supplementary Tables S1–S3), there was a clear distinction between the groups of low Cd (0.5–50 nM) and the high, but sublethal Cd (140–270 nM). Depicted significant increases or decreases are relative to the control, unless stated otherwise. In leaves, concentrations of all detected amino acids were similarly low for 0.5, 20, and 50 nM, but increased for 140 nM and 270 nM Cd. In roots, the trend was similar, though no changes were observed for Gly, Ile, and Tyr.

Metabolites connected to the citric acid cycle decreased (fumaric acid, malic acid, and 2-oxoglutarate), were relatively similar (succinic acid, lactic acid, and isocitric acid), or rather increased [phosphoenolpyruvate (PEP) and citric acid] with increasing Cd concentration in the leaves (significant changes for 2-oxoglutarate, PEP, and malic acid). In the roots, values were noisier compared with the leaves, but with significant increases towards higher Cd concentrations for PEP, and decreases for malic acid.

In the leaves, most sugars and sugar alcohols showed no linear trend in response to Cd exposure, but decreased until the threshold concentration 140 nM, and increased at higher Cd (significant for arabitol and arabinose). Only sorbitol/mannitol increased linearly until 270 nM Cd. In roots, arabitol, arabinose, glucose-x-phosphate, hexose, and sorbitol/mannitol increased significantly towards the highest Cd concentration. Starch accumulation in the leaves decreased towards higher Cd concentrations (Supplementary Table S1).

Regarding the lipidomics, there was a clear effect of Cd treatment on the composition of lipophilic compounds in leaves and roots. The Cd effect split the analysed samples into two groups: low Cd (0.5–50 nM) and high Cd (140–270 nM). Altogether 2376 compounds were detected in the leaves and nearly 7000 in roots. Among the 140 (leaves; Supplementary Table S2) or 50 (roots; Supplementary Table S3) lipophilic compounds that could be identified, and after omitting those originating from plasticware etc. instead of plant samples, the following showed the most remarkable response to Cd treatment in leaves (Table 1; Supplementary Table S2). The vitamin D metabolites or analogues [i.e. secalciferol (24-homo-1,25-dihydroxyvitamin D3) and (1*S*,3*R*,5*Z*,7*E*)-25-methoxy-9,10-secocholesta-5,7,10-triene-1,3-diol] were drastically down-regulated in the leaves. Slight decreases were observed in roots exposed to low, non-toxic Cd concentrations, but 22-fold or 30-fold increases were found in roots of the plants exposed to 140 nM or 270 nM Cd, respectively. Among the fatty acids, 9-KODE [(10*E*,12*Z*)-9-oxooctadeca-10,12-dienoic acid, a double unsaturated fatty acid] was most severely reduced, by a factor of 2.4, already at 20 nM Cd and by a factor of 10 at 140 nM Cd. The strongest up-regulation of fatty acids in response to Cd toxicity was found for the polyunsaturated MG 24:4 <{[2~(*S*)]-2,3-dihydroxypropyl} [5~(*E*),8~(*E*),11~(*E*),14~(*E*)]-tetracosa-5,8,11,14-tetraenoate>, where a gradual up-regulation during low Cd toxicity (6.7× at 50 nM) was followed by a drastic further increase at 270 nM Cd (110× higher than in the control). An unusual response was found among some saturated fatty acids, such as 2-hydroxyeicosanoic acid (=2-hydroxyarachidic acid), the levels of which dropped to 70% of the control at 20 nM Cd and 83% at 50 nM, but increased to 14× the control level at 140 nM Cd and to 64× at 270 nM Cd. The most up-regulated lipophilic compound in leaves was aflatoxin G1. This was almost 3× increased already at 20 nM Cd, 8.2× at 50 nM, 34× at 140 nM, and almost 100× at 270 nM Cd. It was not detected in roots, but aflatoxin B1 decreased up to 12× with respect to the control. Polyphenols such as psoralidin (3,9-dihydroxy-2-prenylcoumestan) and *N*-(3-chloro-4-morpholinophenyl)-6-oxo-1,4,5,6-tetrahydro-3-pyridazinecarboxamide, as well as phytoalexins such as musanolone F [2-hydroxy-9-(4-hydroxy-3-methoxyphenyl)-1H-phenalen-1-one] and sakuranetin, became much more abundant during sublethal Cd toxicity (up to 26× or 80×, respectively). Compounds of phytoalexin metabolism and general stress response (antibiotic and inflammatory activities) mostly increased in the roots due to Cd stress: coumafuryl increased up to 20-fold and formononetin up to 34-fold in the plants exposed to the highest Cd concentration. Osajin (a flavonoid) decreased up to 6× in the plants exposed to 20 nM and 50 nM, but increased 10× in 270 nM.

**Table 1.** Selected lipophilic compounds in leaves and roots of soybean plants exposed to various Cd concentrations with respect to the control samples

	(20Cd)/(0Cd)	(50Cd)/(0Cd)	(140Cd)/(0Cd)	(270Cd)/(0Cd)	Statistics
Lipophilic compounds in leaves					
2-Hydroxyarachidic acid	–0.48	–0.27	3.80	6.00	Prob>*F* 0.00001 *R*^2^=0.77
Musanolone F: 2-hydroxy-9-(4-hydroxy-3-methoxyphenyl)-1H-phenalen-1-one	1.56	1.95	4.25	4.68	Prob>*F* 0.01 *R*^2^=0.36
(+/–)-Sakuranetin	0.81	1.19	6.33	6.29	Prob>*F* 0.0015 *R*^2^=0.52
Aflatoxin G1	1.54	3.05	5.08	6.64	Prob>*F* 0.0089 *R*^2^=0.38
*N*-(3-Chloro-4-morpholinophenyl)-6-oxo-1,4,5,6-tetrahydro-3-pyridazinecarboxamide	1.11	1.79	4.31	4.75	Prob>*F* 0.002 *R*^2^=0.64
Psoralidin	1.03	2.41	4.16	5.58	Prob>*F* 0.0014 *R*^2^=0.66
MG 24:4	1.27	1.55	2.75	6.78	Prob>*F* 0.0102 *R*^2^=0.36
PG 34:4	–0.66	–0.87	–2.09	–3.32	Prob>*F* 0.004 *R*^2^=0.44
PG 34:3	–0.74	–0.88	–2.25	–3.32	Prob>*F* 0.008 *R*^2^=0.38
Secalciferol; 24-Homo-1,25-dihydroxyvitamin D3	–0.18	–1.61	–4.38	–3.22	Prob>*F* 0.046 *R*^2^=0.22
(1*S*,3*R*,5*Z*,7*E*)-25-Methoxy-9,10-secocholesta-5,7,10-triene-1,3-diol	–0.03	–1.38	–3.67	–2.94	Prob>*F* 0.015 *R*^2^=0.33
9-KODE	–1.26	–1.74	–3.34	–1.52	Prob>*F* 0.19 *R*^2^=0.06
Lipophilic compounds in roots					
Formononetin	–0.31	–0.37	**2.57**	**5.10**	Prob>*F* 1.6E-4 *R*^2^=0.68
Aflatoxin P1	2.75	2.15	1.55	4.90	Prob>*F* 0.33 *R*^2^=0.002
(20*R*,22*E*)-Stigmasta-4,22,25-trien-3-one	–0.29	–0.52	**4.48**	**4.93**	Prob>*F* 5.6E-4 *R*^2^=0.6
Coumafuryl	1.58	0.96	**3.13**	**4.37**	Prob>*F* 8E-5 *R*^2^=0.75
Osajin	–2.66	–1.65	0.73	3.31	Prob>*F* 0.014 *R*^2^=0.36
Aflatoxin B1	1.59	–3.16	–2.14	–3.56	Prob>*F* 0.08 *R*^2^=0.18

Up-regulation (positive values, shades of blue) or down-regulation (negative values, shades of red) are depicted in log2 mode. Data are the mean of three experiments.

### Cd metalloproteomics

**Fig. 8.**
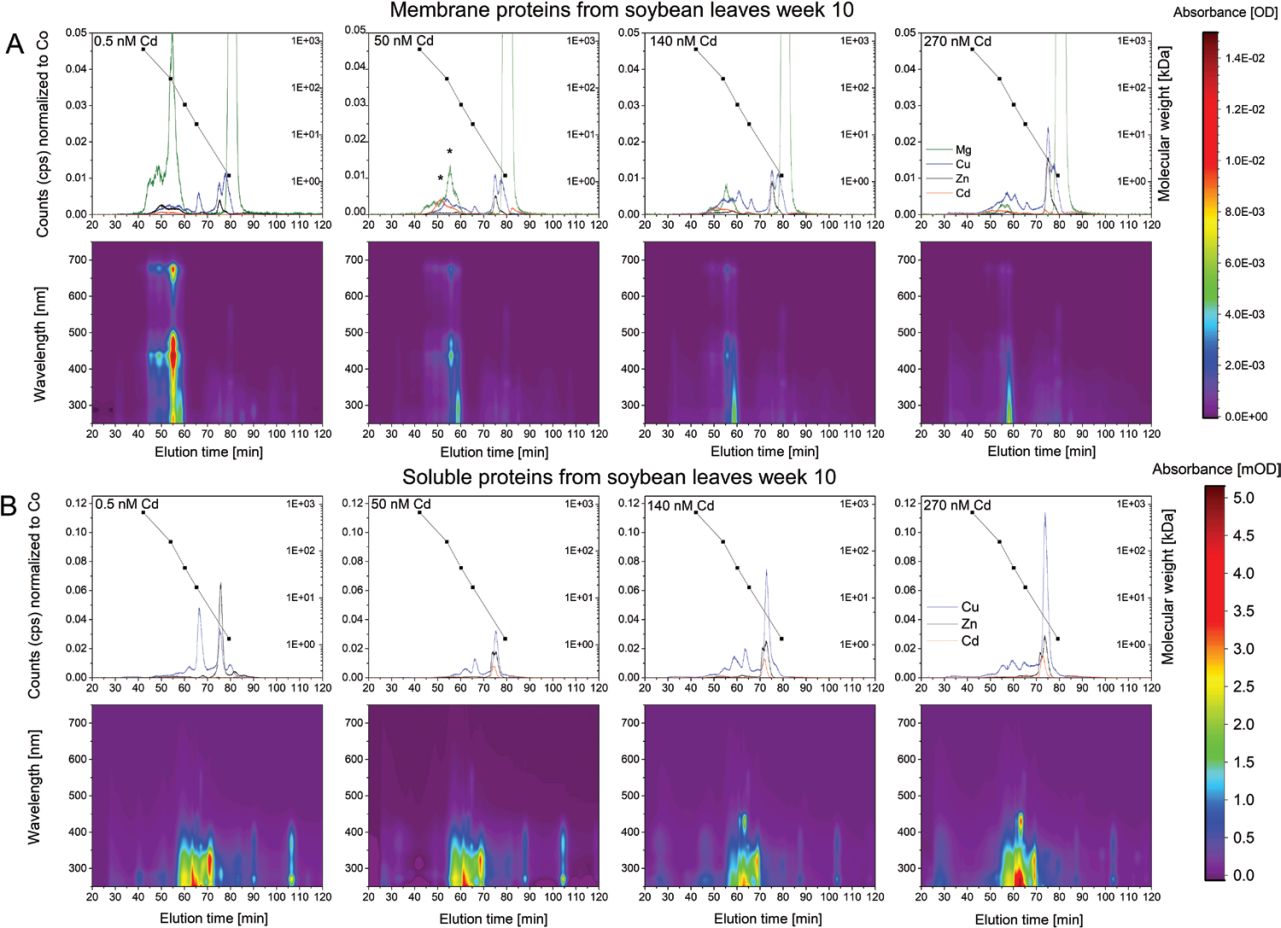
Effect of Cd exposure on membrane and soluble proteins of soybean leaves after 10 weeks of exposure. ICP-MS count rates and protein chromatographs were normalized to the Co signal of added vitamin B_12_. Chromatographs from one of three experiments are shown as examples. Fractions subjected to MS are labelled with an asterisk.

Binding of Cd to membrane proteins of leaves was visible from the lowest Cd concentration (Fig. 8; Supplementary Fig. S3). Though the photosynthetic complexes were not separated as well as for *C. demersum* (Andresen *et al.*, 2016), they were the most abundant individual proteins in the membrane fraction; the peaks at Chl-specific wavelengths of 280, 420, and 660 nm and the Mg content indicated their identity. From the 0.5 nM to 50 nM Cd treatment, Cd could be found in the peaks of the LHC trimers and monomers at 5 and 10 weeks of exposure to Cd. Contrary to the earlier study on *C. demersum*, however, the Cd peaks did not directly correlate with the peaks of Mg. This shows that additional high-affinity Cd-binding sites exist in soybean membrane proteins. Regarding the toxicity threshold Cd concentration, the peak in LHCII trimers and monomers decreased, while the one at ~42 min (=118 kDa, not correlated to Mg) became dominant. The Mg and protein content drastically decreased for all Cd concentrations compared with the control sample. Fractions eluted corresponding to the Cd peak at ~50 min and corresponding to the LHC peak (~55 min) had the highest similarity to Chl *a*/*b*-binding proteins, although >50% of the proteins in them could not be identified (Supplementary Table S4). Two further Cd peaks were observable at ~49 min (=290 kDa) and ~56 min (=104 kDa) already in the control sample, indicating high-affinity binding sites. With increasing Cd concentration, the height of the first peak increased as well. At higher Cd, representing low-affinity Cd-binding sites, peaks appeared at 64.8 min (=18.6 kDa), 67.5 min (=11.3 kDa), and 74 min (=3.5 kDa).

For soluble proteins of leaves, Cd, Cu, and Zn binding to a protein (or other ligand) eluting at 72 min (=5 kDa) was observed. This peak normally bound Cu and Zn, but started to include Cd at the highest shown Cd concentration of 270 nM. It could be some complex with a detoxifying ligand such as phytochelatins. A shift occurred for proteins binding Cu. The peak height ratio (min 62 and min 65) changed with increasing Cd concentration.

### XANES spectroscopy

**Fig. 9.**
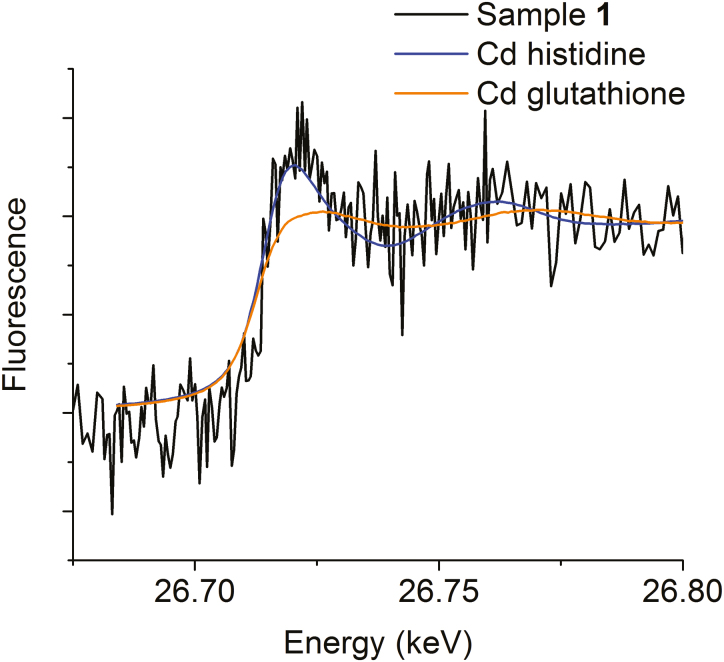
Cd K-edge XANES for sample 1 and the reference compounds Cd histidine and Cd glutathione.

Two replicate samples from independent protein purifications from different plant pools were analysed. Sample 1 contained more Cd than sample 2. XANES of sample 1 (Fig. 9) appeared coordinated to predominantly light ligands, such as N and/or O, which cannot be distinguished by XANES (George *et al.*, 1998). Sample 2 (Supplementary Fig. S4) yielded the same result, in spite of the lower signal to noise ratio. While Cd histidine seems to reproduce the data, a small contribution of S ligands cannot be discarded. Measurement of the extended X-ray absorption fine structure (EXAFS) region, that could determine accurate ligand distances and the presence of S, was not feasible due to the low metal concentration in the purified protein.

## Discussion

Cd toxicity in soybean plants followed, in several important aspects, different mechanisms from those expected from our previous findings in the aquatic model plant *C. demersum* (Andresen *et al.*, 2016) and from earlier published literature (reviewed by Andresen and Küpper, 2013).

### Photosynthesis

Decreases in photosynthetic performances and/or Chl content were found for many species, as reviewed, for example, by Küpper and Andresen (2016). Photosynthesis is, in many cases, the first target of Cd toxicity (Villiers *et al.*, 2011; Andresen *et al.*, 2016). The whole photosynthetic apparatus can be inhibited and, as a result of Cd stress, plants down-regulate enzymes that are connected to the electron transport chain and the Calvin cycle (Kieffer *et al.*, 2008; Fagioni and Zolla, 2009; Durand *et al.*, 2010).

Our data confirm some typical aspects of photosynthesis inhibition, such as a decrease in the contents of Mg (Fig. 3) and Chl (Fig. 4B), impaired oxygen production (Fig. 4A), and photosynthetic activity (Figs 5, 6). However, most parameters of Chl fluorescence induction, in particular *F*_v_/*F*_m_, were far less affected in soybean compared with *C. demersum*. Only the light saturation parameter increased, as was observed earlier (Küpper *et al.*, 2007*a*). A higher *F*_p_ peak could be the result of a large antenna system delivering electrons to a decreased reaction centre pool, or an electron traffic jam between PSII and PSI. The unaffected *F*_v_/*F*_m_ favours the latter, an inhibition of the electron transport between the two photosystems, disturbing the balance of photons absorbed in the antenna versus electrons withdrawn from the plastoquinone (PQ) pool at the beginning of the actinic light period. However, Cd binding to LHCII occurred, as purification of this protein from plants grown on 10–50 nM Cd showed. The inhibition of photosynthetic light reactions (Figs 2, 3) indicates that Cd binding occurred in the plant cells and not as an extraction artefact.

XANES analysis of how Cd is bound in this LHCII (after purification) showed a strong predominance of light ligands (Fig. 9; Supplementary Fig. S4). The incorporation of Cd in Chls could not be directly verified; HPLC-ICP-MS of pigments isolated from the stressed plants (Supplementary Fig. S2) did not reveal [Cd]–Chl. This might be caused, however, by the known chemical instability of [Cd]–Chl (Küpper *et al.*, 1996). While Cd was clearly eluted with the LHC trimers and monomers in membrane proteins in samples of *C. demersum* (Andresen *et al.*, 2016), the dominant Cd peak at low (=specific binding) Cd concentrations from soybean eluted with larger proteins than the antenna molecules (Fig. 8; Supplementary Fig. S3) and are yet to be identified.

For most parameters of photosynthetic activity, there was an initial decrease for the plants exposed to the threshold concentration of 140 nM followed by an increase at higher Cd, especially in w10 (e.g. *F*_v_/*F*_m_, Cd 270 nM onwards, Fig. 5A). Those plants stopped growing and had only very few mature leaves, and therefore the measurements were done on either rather ‘young’ or rather ‘old’ instead of ‘young mature’ leaves (see images in Fig. 1), which obviously behaved differently. In accordance with the uptake route, Cd-related stress symptoms and Cd content were found to be more pronounced in the basal compared with the apical leaves of several plant species (cucumber, Sárvári *et al.*, 1999; poplar, Kieffer *et al.*, 2008; spinach, Fagioni and Zolla, 2009). A similar heterogeneity with respect to leaf age is possible in the soybean leaves and may be responsible for the seemingly noisy results of some fluorescence parameters (Fig. 5) and metal accumulation in the leaves (w5 and w10; Fig. 3).

### Nutrient uptake and NUE

Besides direct toxic effects of Cd, the Cd-mediated limitation of various elements has negative downstream effects on plants. Symptoms of toxicity of one element can be the same as deficiency of another, especially when regarding visual symptoms or growth (Uchida, 2000). Cd is known to interfere with the accumulation and uptake of other nutrients in plants (Benavides *et al.*, 2005; Haouari *et al.*, 2012), but the current study showed that this already occurs at much lower Cd than what was used in most previous studies.

Root uptake of essential elements and their translocation into the different shoot organs becomes inhibited by Cd competing for the transport capacity or by blockage of the respective transporters (Clemens, 2006; Clemens *et al.*, 2013; Andresen *et al.*, 2018). The drastic decrease of K in roots already from 140 nM Cd indicates membrane leakiness as an additional mechanism. The Cd-induced increase of Zn in the leaves, the seeds, and after an initial decrease in the roots (Fig. 3) indicates increased expression of the respective Zn transporters as an attempt of the plant to counteract Cd toxicity by increased Zn uptake and internal re-distribution to the organs. The higher Cd accumulation in the roots reflects the usual pattern observed for non-accumulator plants, where Cd accumulates mostly in the root apoplasm and the symplastic accumulation occurs mostly in the vacuole, indicating chelation of Cd ions into less sensitive compartments (see review by Lux *et al.*, 2011).

In our study, contents of the metal macronutrients Ca, K, and Mg decreased gradually in leaves of the plants exposed to the threshold concentration and above. This is consistent with earlier results of de Almeida *et al.* (2017), who found that limitation of Ca>N>K>Mg>P>S was limiting dry weight biomass of soybean and leading to nutritional disorder symptoms. Vice versa, enhanced nutrition of those elements was shown to lessen Cd toxicity in soybean (Shamsi *et al.*, 2008, Nazar *et al.*, 2012). However, a transcriptomic study with Mg starvation in *Arabidopsis thaliana* revealed a protective effect of Mg-limited pre-treatment against Cd stress and Fe starvation, emphasizing the complexity of the nutritional status in plants in different situations (Hermans *et al.*, 2011). Identification of the Cd-binding proteins newly found in the current study, together with analysis of regulation of expression of these and other proteins (transcriptomics), is the task of planned future studies.

### Metabolomics including lipidomics

Cd treatment led to the increased accumulation of many amino acids in the leaves and roots (Fig. 7; Supplementary Table S1). Histidine is involved in the translocation mechanisms in both non-hyperaccumulating and hyperaccumulating plants (see reviews by Leitenmaier and Küpper, 2013; Andresen *et al.*, 2018). Cd exposure of Massai grass led to increased histidine concentrations in all plant tissues (Rabêlo *et al.*, 2018). In our study, concentrations of histidine in the leaves were below the detection limit, but increased up to 54-fold in the roots of Cd-treated plants compared with the control (Fig. 7; Supplementary Table S1), indicating different ligands involved in Cd detoxification in soybean leaves compared with roots.

Compounds with osmolytic or compatible-solute-like properties increased in the leaves exposed to sublethal Cd (Fig. 7). These compounds contribute to the detoxification of reactive oxygen species (ROS), the osmotic adjustment of the cell, and protection of membrane integrity under general stress conditions. Proline, γ-aminobutyric acid (GABA), and soluble sugars were shown to be involved in the attenuation of Cd-caused oxidative stress (Hayat *et al.*, 2012; Keunen *et al.*, 2013). Accumulation of proline is a well-known response to various stress conditions, possibly related to water balance, which can be affected by metal stress (Schat *et al.*, 1997; Balestrasse *et al.*, 2005). Soybean exposed to 200 μM Cd yielded proline concentrations 2.7 times higher than the control roots after 6 d of exposure (Balestrasse *et al.*, 2005). In our study, proline concentrations were 3.2 times higher compared with the control roots despite the much more gentle Cd stress (1000× lower, nanomolar range), indicating that this response occurs from low toxic Cd concentrations onwards, becomes saturated, and is not further up-regulated at the very high, lethal concentrations that were used in earlier studies. Further, the Cd-stressed soybean leaves had increased levels of the phytohormone salicylic acid, which also acts as a signalling molecule in global stress response (Fig. 7; Supplementary Table S1), as well as phytoalexins (e.g. psoraldin); that is, defence metabolites with antimicrobial and antioxidative properties (Supplementary Tables S2, S3).

Some immense changes were observed in the lipids of soybean leaves and roots, though by far not all substances could be identified. Most remarkably, levels of the polyunsaturated fatty acid MG 24:4 increased tremendously, while the phosphatidylglycerols (lipid head groups) PG 34:4 and PG 34:3 decreased. This, as well as the more complicated Cd response of saturated fatty acids, indicated structural changes of the membranes of Cd-treated soybean leaves. This might be a regulation of membrane fluidity, a possible topic for future work. Opposite to our results from nanomolar Cd treatments, earlier work with micromolar Cd levels had shown decreased levels of unsaturated fatty acids in Cd-exposed plants, which was explained by peroxidation of unsaturated lipids plus inhibited biosynthesis pathways (Ben Ammar *et al.*, 2008; reviewed by Upchurch, 2008). This lack of ROS-related effects in the current study is in line with the conclusions from our studies with arsenic (As), Cd, and Cu in *C. demersum* that sublethal, in contrast to lethal, metal(loid) toxicity is not dominated by oxidative stress (Thomas *et al.*, 2013; Mishra *et al.*, 2014; Andresen *et al.*, 2016).

Metal treatment was previously shown to influence metabolites in *Brassica rapa* drastically. Depending on the metal to which they were exposed (Fe, Cu, or Mn), more amino acids, phenolics, and glucosinolates were produced (Jahangir *et al.*, 2008; reviewed by Villiers *et al.*, 2011). High concentrations, however, led to a decrease in primary and secondary metabolites (Jahangir *et al.*, 2008), which is in accordance with the general toxicity response, as also observed in soybean (Fig. 7; Supplementary Tables S1–S3). Metabolites related to the tricarboxylic acid (TCA) cycle were lower in plants exposed to the higher Cd concentrations, reflecting their decreased energy production together with a decreased energy demand due to strongly reduced biomass production. However, only a few significant decreases due to Cd exposure were found for the sugars, which seems contradictory to the impairment of photosynthesis (Figs 5, 6). A possible explanation is that carbohydrates decreased in the control plants and those exposed to low Cd as well, due to senescence and seed ripening. A similar age dependency was found for Cd-treated tomato leaves: glucose concentrations decreased in mature leaves when exposed to 20 µM Cd for 90 d and increased at even higher Cd concentrations, while in young leaves all Cd concentrations led to significant decreases (Hédiji *et al.*, 2010).

Phenolic compounds were affected by Cd stress in the current study as well, among which two are particularly interesting. First, compounds related to vitamin D drastically decreased in leaves of Cd-stressed soybean. Secondly, the highly toxic aflatoxin drastically increased. Although this was now only found in leaves, as seeds were not a subject of this study, it is likely that similar effects will also occur in seeds, which would be important for agriculture.

### Conclusions

Numerous effects of Cd toxicity in plants have been studied, but the mechanisms are still not completely unravelled, in particular in the sublethal nanomolar concentration range. We showed that a combination of impairment of photosynthesis, probably by inhibiting LHCII as well as the further electron transport chain to PSI, lessens the necessary energy production in the cells already from low nanomolar Cd. The amino acid and carbohydrate metabolism decreased in favour of molecules involved in Cd stress tolerance (e.g. the antioxidative system and detoxifying ligands). Lipid composition changed at sublethal Cd toxicity probably as a tuning of membrane fluidity. Altogether, our data showed that the plants exposed to the threshold Cd concentration (140 nM) were stressed, but their stress tolerance mechanisms were still active. Plants exposed to the higher but still sublethal Cd concentrations (270 nM and onwards) showed more severe signs of Cd toxicity for most measured parameters, possibly due to worsened nutrient deficiency caused by blocked transporters, and increasing membrane leakiness in roots. However, the still increasing polyunsaturated fatty acid MG 24:4 indicated that even at almost lethal Cd concentrations the decrease of unsaturated fatty acids by oxidative stress did not yet occur. While the current study aimed at investigating the fitness of the plants and not food (i.e. seed) quality, the finding of drastically changed levels of beneficial and toxic secondary metabolites in response to nanomolar Cd indicates that future work should investigate to what extent low sublethal Cd also affects the quality and safety of soybean seeds.

## Supplementary data

Supplementary data are available at *JXB* online.

**Table S1.** List of metabolites detected in leaves and roots of soybean plants exposed to various Cd concentrations for 10 weeks.

**Table S2.** List of detected, and partly identified lipids isolated from leaves of soybean plants exposed to various Cd concentrations.

**Table S3.** List of detected, and partly identified lipids isolated from roots of soybean plants exposed to various Cd concentrations for 10 weeks.

**Table S4.** Identification of membrane proteins and peptides isolated from leaves of soybean plants exposed to 50 nM Cd for 10 weeks.

**Table S5.** Reports from all statistical tests that were used for the description of results in the manuscript.

**Fig. S1.** Nutrient utilization efficiency and utilization index of soybean plants exposed to various Cd concentrations for 10 weeks.

**Fig. S2.** Acetone extracts from soybean leaves exposed to low, medium, and high but sublethal Cd concentrations for 10 weeks with a focus on the Chl molecules.

**Fig. S3.** Effect of Cd exposure on membrane proteins from single soybean leaves after 5 weeks of exposure.

**Fig. S4.** XANES of sample 2 of the purified LHCII from Cd-exposed soybean leaves.

**Fig. S5.** Concentration of Mn in the hydroponic solutions (A) and accumulation of Mn in different tissues of plants after 5 weeks (leaves only) and 10 weeks of exposure to various Cd concentrations (B).

**Protocol S1.** Identification of isolated proteins from selected fractions of membrane proteins from the leaves of soybean plants exposed to 50 nM Cd.

**Protocol S2.** Isolation and identification of metabolites from leaves of soybean plants exposed to various Cd concentrations.

**Protocol S3.** Isolation and identification of lipids from soybean leaves of soybean plants exposed to various Cd concentrations.

erz530_suppl_supplementary_table_S1

erz530_suppl_supplementary_table_S2

erz530_suppl_supplementary_table_S3

erz530_suppl_supplementary_table_S4

erz530_suppl_supplementary_table_S5

erz530_suppl_supplementary_figures_S1-S5andprotocols_S1-S3

## Abbreviations

Chl: chlorophyll
LHC: light-harvesting complex
PS rate: net photosynthetic oxygen release rate
PEP: phosphoenolpyruvate
XANES: X-ray absorption near edge structure.
