# Supplementary material for: Chronic exposure of soybean plants to nanomolar cadmium reveals specific additional high-affinity targets of cadmium toxicity
Source: J Exp Bot. 2019 Nov 24;71(4):1628–44. doi: 10.1093/jxb/erz530 (PMC7242006; doi:10.1093/jxb/erz530)
Supplement: erz530_suppl_supplementary_table_S5 [file exbotj_71_4_1628_s15.pdf]

Supporting information

Chronic exposure of soybean plants to nanomolar cadmium reveals specific additional high-affinity targets of Cd toxicity.

Elisa Andresen[1], Lyudmila Lyubenova[1], Tomáš Hubáček[2], Syed Nadeem Hussain Bokhari[1], Šárka Matoušková[3], Ana Mijovilovich[1], Jan Rohovec[3] and Hendrik Küpper[1,4]*

1) Biology Centre of the Czech Academy of Sciences, Institute of Plant Molecular Biology, Department of Plant Biophysics and Biochemistry. Branišovská 31/1160. 370 05 České Budějovice. Czech Republic.
2) Biology Centre of the Czech Academy of Sciences, Institute of Hydrobiology, Department of Hydrochemistry and Ecosystem Modelling, Na Sádkách 7, CZ-37005 České Budějovice, Czech Republic;
3) Institute of Geology, Czech Academy of Sciences, Department of Geological Processes, Rozvojová 269, 165 00 Praha 6, Czech Republic.
4) University of South Bohemia, Faculty of Sciences, Department of Experimental Plant Biology, Branišovská 31/1160, 370 05 České Budějovice, Czech Republic.

Author for correspondence: Hendrik Küpper, Tel: +420 387 775 537, Email: Hendrik.Kuepper@umbr.cas.cz

Table S5: Reports from all statistical tests that were used for the description of results in the manuscript.

Order:
1) Biomass
2) Elements in the nutrient solution
3) Element accumulation in the plant tissues
4) Oxygen production and chlorophyll accumulation
5) Fluorescence parameters - F_v/F_m and Saturation
6) OJIP - Φ_{RE1o}
7) Metabolites in leaves and roots

1) Biomass

Biomass - Leaves

Two Way Analysis of Variance
General Linear Model (No Interactions)
Dependent Variable: biomass leaves
Normality Test (Shapiro-Wilk) Failed (P < 0.050)
Equal Variance Test: Failed (P < 0.050)

Source of Variation	DF	SS	MS	F	P
Exp	3	12.652	4.217	2.146	0.097
Cd conc	9	550.959	61.218	31.146	<0.001
Residual	140	275.167	1.965		
Total	152	852.724	5.610		

The difference in the mean values among the different levels of Exp is not great enough to exclude the possibility that the difference is just due to random sampling variability after allowing for the effects of differences in Cd conc. There is not a statistically significant difference (P = 0.097).

The difference in the mean values among the different levels of Cd conc is greater than would be expected by chance after allowing for effects of differences in Exp. There is a statistically significant difference (P = <0.001). To isolate which group(s) differ from the others use a multiple comparison procedure.

Power of performed test with alpha = 0.0500: for Exp : 0.294
Power of performed test with alpha = 0.0500: for Cd conc : 1.000

Least square means for Exp :

Group	Mean	SEM
272.000	1.364	0.238
273.000	1.843	0.222
278.000	2.160	0.222
280.000	1.975	0.231

Least square means for Cd conc :

Group	Mean	SEM
0.500	3.951	0.350
10.000	4.208	0.410
20.000	3.846	0.350
50.000	4.641	0.350
140.000	0.597	0.350
270.000	0.367	0.350
550.000	0.337	0.350
1270.000	0.252	0.350
3100.000	0.105	0.362
27500.000	0.0525	0.376

All Pairwise Multiple Comparison Procedures (Holm-Sidak method):
Overall significance level = 0.05

Comparisons for factor: **Exp**

Comparison	Diff of Means	t	P	P<0.050
278.000 vs. 272.000	0.797	2.452	0.089	No
280.000 vs. 272.000	0.611	1.844	0.294	No
273.000 vs. 272.000	0.479	1.475	0.459	No
278.000 vs. 273.000	0.317	1.013	0.676	No
278.000 vs. 280.000	0.185	0.578	0.810	No

280.000 vs. 273.000	0.132	0.413	0.680	No

Comparisons for factor: **Cd conc**

**Comparison**	**Diff of Means**	**t**	**P**	**P<0.050**
50.000 vs. 3100.000	4.537	9.001	<0.001	Yes
50.000 vs. 27500.000	4.589	8.930	<0.001	Yes
50.000 vs. 1270.000	4.389	8.854	<0.001	Yes
50.000 vs. 550.000	4.304	8.684	<0.001	Yes
50.000 vs. 270.000	4.274	8.622	<0.001	Yes
50.000 vs. 140.000	4.044	8.159	<0.001	Yes
0.500 vs. 3100.000	3.846	7.630	<0.001	Yes
0.500 vs. 27500.000	3.898	7.586	<0.001	Yes
10.000 vs. 3100.000	4.103	7.496	<0.001	Yes
10.000 vs. 27500.000	4.155	7.468	<0.001	Yes
0.500 vs. 1270.000	3.698	7.461	<0.001	Yes
20.000 vs. 3100.000	3.741	7.422	<0.001	Yes
20.000 vs. 27500.000	3.793	7.382	<0.001	Yes
10.000 vs. 1270.000	3.955	7.329	<0.001	Yes
0.500 vs. 550.000	3.614	7.291	<0.001	Yes
20.000 vs. 1270.000	3.593	7.249	<0.001	Yes
0.500 vs. 270.000	3.583	7.229	<0.001	Yes
10.000 vs. 550.000	3.871	7.173	<0.001	Yes
10.000 vs. 270.000	3.840	7.116	<0.001	Yes
20.000 vs. 550.000	3.509	7.079	<0.001	Yes
20.000 vs. 270.000	3.478	7.017	<0.001	Yes
0.500 vs. 140.000	3.354	6.766	<0.001	Yes
10.000 vs. 140.000	3.611	6.691	<0.001	Yes
20.000 vs. 140.000	3.249	6.554	<0.001	Yes
50.000 vs. 20.000	0.796	1.605	0.915	No
50.000 vs. 0.500	0.691	1.393	0.973	No
140.000 vs. 27500.000	0.544	1.059	0.999	No
140.000 vs. 3100.000	0.492	0.977	0.999	No
50.000 vs. 10.000	0.434	0.803	1.000	No
140.000 vs. 1270.000	0.344	0.695	1.000	No
10.000 vs. 20.000	0.362	0.671	1.000	No
270.000 vs. 27500.000	0.315	0.613	1.000	No
550.000 vs. 27500.000	0.284	0.553	1.000	No
140.000 vs. 550.000	0.260	0.525	1.000	No
270.000 vs. 3100.000	0.263	0.522	1.000	No
10.000 vs. 0.500	0.257	0.476	1.000	No
140.000 vs. 270.000	0.229	0.463	1.000	No
550.000 vs. 3100.000	0.232	0.461	1.000	No
1270.000 vs. 27500.000	0.200	0.389	1.000	No
1270.000 vs. 3100.000	0.148	0.293	1.000	No
270.000 vs. 1270.000	0.115	0.232	1.000	No
0.500 vs. 20.000	0.105	0.212	0.999	No
550.000 vs. 1270.000	0.0844	0.170	0.998	No
3100.000 vs. 27500.000	0.0521	0.0999	0.994	No
270.000 vs. 550.000	0.0306	0.0618	0.951	No

Biomass - Stems

Two Way Analysis of Variance
General Linear Model (No Interactions)
Dependent Variable: biomass stems
Normality Test (Shapiro-Wilk) Failed (P < 0.050)
Equal Variance Test: Failed (P < 0.050)

Source of Variation	DF	SS	MS	F	P
Exp	3	1.647	0.549	0.787	0.503
Cd conc	9	277.795	30.866	44.264	<0.001
Residual	140	97.626	0.697		
Total	152	381.200	2.508		

The difference in the mean values among the different levels of Exp is not great enough to exclude the possibility that the difference is just due to random sampling variability after allowing for the effects of differences in Cd conc. There is not a statistically significant difference (P = 0.503).

The difference in the mean values among the different levels of Cd conc is greater than would be expected by chance after allowing for effects of differences in Exp. There is a statistically significant difference (P = <0.001). To isolate which group(s) differ from the others use a multiple comparison procedure.

Power of performed test with alpha = 0.0500: for Exp : 0.0500
Power of performed test with alpha = 0.0500: for Cd conc : 1.000

Least square means for Exp :

Group	Mean	SEM
272.000	1.271	0.141
273.000	1.484	0.132
278.000	1.462	0.132
280.000	1.564	0.138

Least square means for Cd conc :

Group	Mean	SEM
0.500	2.956	0.209
10.000	2.808	0.244
20.000	2.963	0.209
50.000	3.569	0.209
140.000	0.624	0.209
270.000	0.526	0.209
550.000	0.423	0.209
1270.000	0.309	0.209
3100.000	0.177	0.216
27500.000	0.0982	0.224

All Pairwise Multiple Comparison Procedures (Holm-Sidak method):
Overall significance level = 0.05

Comparisons for factor: **Exp**

Comparison	Diff of Means	t	P	P<0.050
280.000 vs. 272.000	0.294	1.487	0.594	No
273.000 vs. 272.000	0.213	1.103	0.795	No
278.000 vs. 272.000	0.191	0.988	0.792	No
280.000 vs. 278.000	0.102	0.537	0.932	No
280.000 vs. 273.000	0.0802	0.420	0.894	No
273.000 vs. 278.000	0.0222	0.119	0.905	No

Comparisons for factor: **Cd conc**

Comparison	Diff of Means	t	P	P<0.050
50.000 vs. 27500.000	3.471	11.339	<0.001	Yes
50.000 vs. 3100.000	3.392	11.299	<0.001	Yes
50.000 vs. 1270.000	3.260	11.042	<0.001	Yes
50.000 vs. 550.000	3.146	10.657	<0.001	Yes
50.000 vs. 270.000	3.043	10.307	<0.001	Yes
50.000 vs. 140.000	2.944	9.973	<0.001	Yes
20.000 vs. 27500.000	2.864	9.358	<0.001	Yes
0.500 vs. 27500.000	2.858	9.338	<0.001	Yes
20.000 vs. 3100.000	2.786	9.279	<0.001	Yes

0.500 vs. 3100.000	2.780	9.259	<0.001	Yes
20.000 vs. 1270.000	2.654	8.989	<0.001	Yes
0.500 vs. 1270.000	2.648	8.967	<0.001	Yes
20.000 vs. 550.000	2.540	8.603	<0.001	Yes
0.500 vs. 550.000	2.534	8.582	<0.001	Yes
20.000 vs. 270.000	2.437	8.254	<0.001	Yes
0.500 vs. 270.000	2.431	8.233	<0.001	Yes
10.000 vs. 27500.000	2.709	8.175	<0.001	Yes
10.000 vs. 3100.000	2.631	8.070	<0.001	Yes
20.000 vs. 140.000	2.338	7.919	<0.001	Yes
0.500 vs. 140.000	2.332	7.898	<0.001	Yes
10.000 vs. 1270.000	2.499	7.774	<0.001	Yes
10.000 vs. 550.000	2.385	7.420	<0.001	Yes
10.000 vs. 270.000	2.282	7.099	<0.001	Yes
10.000 vs. 140.000	2.183	6.792	<0.001	Yes
50.000 vs. 10.000	0.761	2.368	0.335	No
50.000 vs. 0.500	0.613	2.075	0.557	No
50.000 vs. 20.000	0.606	2.053	0.557	No
140.000 vs. 27500.000	0.526	1.719	0.809	No
140.000 vs. 3100.000	0.448	1.491	0.920	No
270.000 vs. 27500.000	0.427	1.396	0.944	No
270.000 vs. 3100.000	0.349	1.163	0.986	No
140.000 vs. 1270.000	0.316	1.069	0.991	No
550.000 vs. 27500.000	0.324	1.060	0.989	No
550.000 vs. 3100.000	0.246	0.819	0.998	No
270.000 vs. 1270.000	0.217	0.735	0.999	No
1270.000 vs. 27500.000	0.211	0.688	0.999	No
140.000 vs. 550.000	0.202	0.684	0.998	No
20.000 vs. 10.000	0.155	0.482	1.000	No
0.500 vs. 10.000	0.149	0.462	0.999	No
1270.000 vs. 3100.000	0.132	0.440	0.998	No
550.000 vs. 1270.000	0.114	0.385	0.998	No
270.000 vs. 550.000	0.103	0.349	0.994	No
140.000 vs. 270.000	0.0987	0.334	0.982	No
3100.000 vs. 27500.000	0.0784	0.252	0.961	No
20.000 vs. 0.500	0.00625	0.0212	0.983	No

Biomass - Roots

Two Way Analysis of Variance
General Linear Model (No Interactions)
Dependent Variable: biomass roots
Normality Test (Shapiro-Wilk) Failed (P < 0.050)
Equal Variance Test: Passed (P = 1.000)

Source of Variation	DF	SS	MS	F	P
Exp	3	207.675	69.225 0	.844	0.482
Cd conc	9	26640.243	2960.027	36.106	<0.001
Residual	26	2131.547	81.983		
Total	38	29178.610	767.858		

The difference in the mean values among the different levels of Exp is not great enough to exclude the possibility that the difference is just due to random sampling variability after allowing for the effects of differences in Cd conc. There is not a statistically significant difference (P = 0.482).

The difference in the mean values among the different levels of Cd conc is greater than would be expected by chance after allowing for effects of differences in Exp. There is a statistically significant difference (P = <0.001). To isolate which group(s) differ from the others use a multiple comparison procedure.

Power of performed test with alpha = 0.0500: for Exp : 0.0500
Power of performed test with alpha = 0.0500: for Cd conc : 1.000

Least square means for Exp :

Group	Mean	SEM
272.000	21.228	3.068
273.000	26.864	2.863
278.000	21.734	2.863
280.000	24.963	2.863

Least square means for Cd conc :

Group	Mean	SEM
0.500	50.525	4.527
10.000	50.514	5.300
20.000	61.105	4.527
50.000	59.955	4.527
140.000	4.065	4.527
270.000	3.275	4.527
550.000	2.667	4.527
1270.000	2.098	4.527
3100.000	1.473	4.527
27500.000	1.297	4.527

All Pairwise Multiple Comparison Procedures (Holm-Sidak method):
Overall significance level = 0.05

Comparisons for factor: **Exp**

Comparison	Diff of Means	t	P	P<0.050
273.000 vs. 272.000	5.636	1.343	0.719	No
273.000 vs. 278.000	5.130	1.267	0.705	No
280.000 vs. 272.000	3.735	0.890	0.854	No
280.000 vs. 278.000	3.229	0.797	0.817	No
273.000 vs. 280.000	1.901	0.469	0.872	No
278.000 vs. 272.000	0.505	0.120	0.905	No

Comparisons for factor: **Cd conc**

Comparison	Diff of Means	t	P	P<0.050
20.000 vs. 27500.000	59.808	9.341	<0.001	Yes
20.000 vs. 3100.000	59.632	9.314	<0.001	Yes
20.000 vs. 1270.000	59.007	9.216	<0.001	Yes
50.000 vs. 27500.000	58.658	9.162	<0.001	Yes
50.000 vs. 3100.000	58.482	9.134	<0.001	Yes
20.000 vs. 550.000	58.437	9.127	<0.001	Yes
50.000 vs. 1270.000	57.857	9.037	<0.001	Yes
20.000 vs. 270.000	57.830	9.032	<0.001	Yes
50.000 vs. 550.000	57.287	8.948	<0.001	Yes
20.000 vs. 140.000	57.040	8.909	<0.001	Yes
50.000 vs. 270.000	56.680	8.853	<0.001	Yes
50.000 vs. 140.000	55.890	8.729	<0.001	Yes
0.500 vs. 27500.000	49.228	7.689	<0.001	Yes
0.500 vs. 3100.000	49.052	7.662	<0.001	Yes
0.500 vs. 1270.000	48.427	7.564	<0.001	Yes
0.500 vs. 550.000	47.857	7.475	<0.001	Yes
0.500 vs. 270.000	47.250	7.380	<0.001	Yes
0.500 vs. 140.000	46.460	7.257	<0.001	Yes
10.000 vs. 27500.000	49.217	7.061	<0.001	Yes
10.000 vs. 3100.000	49.041	7.036	<0.001	Yes
10.000 vs. 1270.000	48.416	6.946	<0.001	Yes
10.000 vs. 550.000	47.846	6.864	<0.001	Yes
10.000 vs. 270.000	47.239	6.777	<0.001	Yes
10.000 vs. 140.000	46.449	6.664	<0.001	Yes

20.000 vs. 0.500	10.580	1.652	0.914	No
20.000 vs. 10.000	10.591	1.520	0.952	No
50.000 vs. 0.500	9.430	1.473	0.957	No
50.000 vs. 10.000	9.441	1.355	0.976	No
140.000 vs. 27500.000	2.768	0.432	1.000	No
140.000 vs. 3100.000	2.592	0.405	1.000	No
270.000 vs. 27500.000	1.978	0.309	1.000	No
140.000 vs. 1270.000	1.967	0.307	1.000	No
270.000 vs. 3100.000	1.802	0.282	1.000	No
140.000 vs. 550.000	1.397	0.218	1.000	No
550.000 vs. 27500.000	1.370	0.214	1.000	No
550.000 vs. 3100.000	1.195	0.187	1.000	No
270.000 vs. 1270.000	1.177	0.184	1.000	No
20.000 vs. 50.000	1.150	0.180	1.000	No
1270.000 vs. 27500.000	0.800	0.125	1.000	No
140.000 vs. 270.000	0.790	0.123	1.000	No
1270.000 vs. 3100.000	0.625	0.0976	1.000	No
270.000 vs. 550.000	0.607	0.0949	1.000	No
550.000 vs. 1270.000	0.570	0.0890	1.000	No
3100.000 vs. 27500.000	0.175	0.0274	1.000	No
0.500 vs. 10.000	0.0113	0.00162	0.999	No

Biomass - Seed pods

Two Way Analysis of Variance
General Linear Model (No Interactions)
Dependent Variable: biomass seed pods
Normality Test (Shapiro-Wilk) Passed (P = 0.054)

Equal Variance Test: Passed (P = 0.249)

**Source of Variation**	**DF**	**SS**	**MS**	**F**	**P**
Exp	3	96.052	32.017	2.889	0.044
Cd conc	3	69.606	23.202	2.094	0.112
Residual	52	576.226	11.081		
Total	58	748.281	12.901		

The difference in the mean values among the different levels of Exp is greater than would be expected by chance after allowing for effects of differences in Cd conc. There is a statistically significant difference (P = 0.044). To isolate which group(s) differ from the others use a multiple comparison procedure.

The difference in the mean values among the different levels of Cd conc is not great enough to exclude the possibility that the difference is just due to random sampling variability after allowing for the effects of differences in Exp. There is not a statistically significant difference (P = 0.112).

Power of performed test with alpha = 0.0500: for Exp : 0.453
Power of performed test with alpha = 0.0500: for Cd conc : 0.270

Least square means for Exp :

**Group**	**Mean**	**SEM**
272.000	6.905	1.003
273.000	6.442	0.832
278.000	9.199	0.832
280.000	5.983	0.861

Least square means for Cd conc :

**Group**	**Mean**	**SEM**
0.500	7.319	0.832
10.000	8.231	1.043
20.000	7.665	0.832

50.000 5.314 0.832

All Pairwise Multiple Comparison Procedures (Holm-Sidak method):
Overall significance level = 0.05

Comparisons for factor: **Exp**

Comparison	Diff of Means	t	P	P<0.050
278.000 vs. 280.000	3.217	2.686	0.057	No
278.000 vs. 273.000	2.758	2.343	0.110	No
278.000 vs. 272.000	2.294	1.760	0.297	No
272.000 vs. 280.000	0.923	0.698	0.866	No
273.000 vs. 280.000	0.459	0.383	0.912	No
272.000 vs. 273.000	0.464	0.356	0.724	No

Comparisons for factor: **Cd conc**

Comparison	Diff of Means	t	P	P<0.050
10.000 vs. 50.000	2.917	2.186	0.184	No
20.000 vs. 50.000	2.351	1.998	0.230	No
0.500 vs. 50.000	2.006	1.704	0.327	No
10.000 vs. 0.500	0.912	0.683	0.873	No
10.000 vs. 20.000	0.566	0.424	0.893	No
20.000 vs. 0.500	0.346	0.294	0.770	No

----

2) Elements in the nutrient solutions

Elements in the nutrient solutions - Cadmium

Two Way Analysis of Variance
General Linear Model - Dependent Variable: Cd
Normality Test (Shapiro-Wilk) Failed (P < 0.050)
Equal Variance Test: Failed (P < 0.050)

Source of Variation	DF	SS	MS	F	P
Cd conc	9	3993684520.225	443742724.469	54.691	<0.001
type	2	820216.176	410108.088	0.0505	0.951
Cd conc x type	18	96763173.211	5375731.845	0.663	0.834
Residual	65	527381534.546	8113562.070		
Total	94	5298126313.071	56363045.884		

The difference in the mean values among the different levels of Cd conc is greater than would be expected by chance after allowing for effects of differences in type. There is a statistically significant difference (P = <0.001). To isolate which group(s) differ from the others use a multiple comparison procedure.

The difference in the mean values among the different levels of type is not great enough to exclude the possibility that the difference is just due to random sampling variability after allowing for the effects of differences in Cd conc. There is not a statistically significant difference (P = 0.951).

The effect of different levels of Cd conc does not depend on what level of type is present. There is not a statistically significant interaction between Cd conc and type. (P = 0.834)

Power of performed test with alpha = 0.0500: for Cd conc : 1.000
Power of performed test with alpha = 0.0500: for type : 0.0500
Power of performed test with alpha = 0.0500: for Cd conc x type : 0.0500

All Pairwise Multiple Comparison Procedures (Holm-Sidak method):
Overall significance level = 0.05

Comparisons for factor: **Cd conc**

Comparison	Diff of Means	t	P	P<0.050
27500 vs. 20	24877.678	17.468	<0.001	Yes
27500 vs. 50	24867.426	17.460	<0.001	Yes
27500 vs. 140	24783.183	17.401	<0.001	Yes
27500 vs. 270	24639.777	17.301	<0.001	Yes
27500 vs. 0.5	24886.308	17.159	<0.001	Yes
27500 vs. 550	24346.936	17.095	<0.001	Yes
27500 vs. 10	24881.775	16.857	<0.001	Yes
27500 vs. 1270	23531.663	16.523	<0.001	Yes
27500 vs. 3100	20341.230	14.282	<0.001	Yes
3100 vs. 20	4536.448	3.378	0.044	Yes
3100 vs. 50	4526.196	3.371	0.043	Yes
3100 vs. 0.5	4545.078	3.316	0.050	Yes
3100 vs. 140	4441.953	3.308	0.049	Yes
3100 vs. 10	4540.545	3.249	0.057	No
3100 vs. 270	4298.547	3.201	0.064	No
3100 vs. 550	4005.706	2.983	0.114	No
3100 vs. 1270	3190.433	2.376	0.451	No
1270 vs. 20	1346.015	1.002	1.000	No
1270 vs. 50	1335.764	0.995	1.000	No
1270 vs. 0.5	1354.645	0.988	1.000	No
1270 vs. 10	1350.113	0.966	1.000	No
1270 vs. 140	1251.521	0.932	1.000	No
1270 vs. 270	1108.114	0.825	1.000	No
1270 vs. 550	815.274	0.607	1.000	No
550 vs. 20	530.742	0.395	1.000	No
550 vs. 0.5	539.371	0.394	1.000	No
550 vs. 50	520.490	0.388	1.000	No
550 vs. 10	534.839	0.383	1.000	No
550 vs. 140	436.247	0.325	1.000	No
550 vs. 270	292.841	0.218	1.000	No
270 vs. 0.5	246.531	0.180	1.000	No
270 vs. 20	237.901	0.177	1.000	No
270 vs. 10	241.999	0.173	1.000	No
270 vs. 50	227.649	0.170	1.000	No
270 vs. 140	143.407	0.107	1.000	No
140 vs. 0.5	103.124	0.0752	1.000	No
140 vs. 10	98.592	0.0705	1.000	No
140 vs. 20	94.494	0.0704	1.000	No
140 vs. 50	84.243	0.0627	1.000	No
50 vs. 0.5	18.881	0.0138	1.000	No
50 vs. 10	14.349	0.0103	1.000	No
50 vs. 20	10.252	0.00763	1.000	No
20 vs. 0.5	8.630	0.00630	1.000	No
10 vs. 0.5	4.532	0.00318	1.000	No
20 vs. 10	4.097	0.00293	0.998	No

Comparisons for factor: **type**

Comparison	Diff of Means	t	P	P<0.050
Barrel vs. pot_w10	208.389	0.312	0.985	No
Barrel vs. pot_w5	142.069	0.181	0.980	No
pot_w5 vs. pot_w10	66.319	0.0827	0.934	No

Elements in the nutrient solutions - Copper

Two Way Analysis of Variance

General Linear Model
Dependent Variable: Cu
Normality Test (Shapiro-Wilk) Failed (P < 0.050)
Equal Variance Test: Failed (P < 0.050)

**Source of Variation**	**DF**	**SS**	**MS**	**F**	**P**
Cd conc	9	17034.972	1892.775	2.915	0.006
type	2	7113.187	3556.594	5.477	0.006
Cd conc x type	18	20247.764	1124.876	1.732	0.056
Residual	65	42206.782	649.335		
Total	94	90796.480	965.920		

The difference in the mean values among the different levels of Cd conc is greater than would be expected by chance after allowing for effects of differences in type. There is a statistically significant difference (P = 0.006). To isolate which group(s) differ from the others use a multiple comparison procedure.

The difference in the mean values among the different levels of type is greater than would be expected by chance after allowing for effects of differences in Cd conc. There is a statistically significant difference (P = 0.006). To isolate which group(s) differ from the others use a multiple comparison procedure.

The effect of different levels of Cd conc does not depend on what level of type is present. There is not a statistically significant interaction between Cd conc and type. (P = 0.056)

Power of performed test with alpha = 0.0500: for Cd conc : 0.787
Power of performed test with alpha = 0.0500: for type : 0.753
Power of performed test with alpha = 0.0500: for Cd conc x type : 0.456

All Pairwise Multiple Comparison Procedures (Holm-Sidak method):
Overall significance level = 0.05

Comparisons for factor: **Cd conc**

**Comparison**	**Diff of Means**	**t**	**P**	**P<0.050**
270 vs. 50	50.094	4.170	0.004	Yes
270 vs. 20	46.908	3.905	0.010	Yes
270 vs. 10	40.667	3.253	0.075	No
270 vs. 0.5	37.412	3.052	0.129	No
140 vs. 50	29.258	2.436	0.517	No
270 vs. 1270	27.903	2.323	0.611	No
270 vs. 3100	26.438	2.201	0.711	No
140 vs. 20	26.072	2.170	0.727	No
270 vs. 550	25.112	2.090	0.783	No
550 vs. 50	24.983	2.080	0.783	No
27500 vs. 50	26.234	2.059	0.789	No
3100 vs. 50	23.657	1.969	0.844	No
270 vs. 27500	23.860	1.873	0.893	No
1270 vs. 50	22.192	1.847	0.899	No
550 vs. 20	21.797	1.815	0.908	No
27500 vs. 20	23.048	1.809	0.904	No
270 vs. 140	20.836	1.735	0.930	No
3100 vs. 20	20.471	1.704	0.935	No
140 vs. 10	19.831	1.586	0.966	No
1270 vs. 20	19.006	1.582	0.962	No
140 vs. 0.5	16.576	1.352	0.993	No
27500 vs. 10	16.807	1.273	0.996	No
550 vs. 10	15.556	1.244	0.996	No
3100 vs. 10	14.230	1.138	0.999	No
27500 vs. 0.5	13.552	1.044	0.999	No
0.5 vs. 50	12.682	1.034	0.999	No
1270 vs. 10	12.765	1.021	0.999	No
550 vs. 0.5	12.300	1.003	0.999	No
3100 vs. 0.5	10.974	0.895	1.000	No

1270 vs. 0.5	9.509	0.776	1.000	No
0.5 vs. 20	9.496	0.775	1.000	No
10 vs. 50	9.427	0.754	1.000	No
140 vs. 1270	7.067	0.588	1.000	No
10 vs. 20	6.241	0.499	1.000	No
140 vs. 3100	5.601	0.466	1.000	No
140 vs. 550	4.276	0.356	1.000	No
27500 vs. 1270	4.043	0.317	1.000	No
20 vs. 50	3.186	0.265	1.000	No
0.5 vs. 10	3.255	0.256	1.000	No
140 vs. 27500	3.024	0.237	1.000	No
550 vs. 1270	2.791	0.232	1.000	No
27500 vs. 3100	2.577	0.202	0.999	No
3100 vs. 1270	1.465	0.122	0.999	No
550 vs. 3100	1.326	0.110	0.992	No
27500 vs. 550	1.252	0.0982	0.922	No

Comparisons for factor: **type**

**Comparison**	**Diff of Means**	**t**	**P**	**P<0.050**
Barrel vs. pot_w5	19.724	2.811	0.019	Yes
Barrel vs. pot_w10	16.291	2.726	0.016	Yes
pot_w10 vs. pot_w5	3.434	0.479	0.634	No

Elements in the nutrient solutions - Iron

Two Way Analysis of Variance
General Linear Model
Dependent Variable: Fe
Normality Test (Shapiro-Wilk) Failed (P < 0.050)
Equal Variance Test: Passed (P = 0.940)

**Source of Variation**	**DF**	**SS**	**MS**	**F**	**P**
Cd conc	9	2830138.711	314459.857	0.293	0.974
type	2	1092332.566	546166.283	0.508	0.604
Cd conc x type	18	5967323.899	331517.994	0.309	0.996
Residual	65	69817046.905	1074108.414		
Total	94	80378936.970	855095.074		

The difference in the mean values among the different levels of Cd conc is not great enough to exclude the possibility that the difference is just due to random sampling variability after allowing for the effects of differences in type. There is not a statistically significant difference (P = 0.974).

The difference in the mean values among the different levels of type is not great enough to exclude the possibility that the difference is just due to random sampling variability after allowing for the effects of differences in Cd conc. There is not a statistically significant difference (P = 0.604).

The effect of different levels of Cd conc does not depend on what level of type is present. There is not a statistically significant interaction between Cd conc and type. (P = 0.996)

Power of performed test with alpha = 0.0500: for Cd conc : 0.0500
Power of performed test with alpha = 0.0500: for type : 0.0500
Power of performed test with alpha = 0.0500: for Cd conc x type : 0.0500

Elements in the nutrient solutions - Nickel

Two Way Analysis of Variance

General Linear Model
Dependent Variable: Ni
Normality Test (Shapiro-Wilk) Failed (P < 0.050)
Equal Variance Test: Passed (P = 0.509)

**Source of Variation**	**DF**	**SS**	**MS**	**F**	**P**
Cd conc	9	303156.623	33684.069	3.208	0.003
type	2	184218.433	92109.216	8.772	<0.001
Cd conc x type	18	162709.246	9039.403	0.861	0.625
Residual	65	682551.912	10500.799		
Total	94	1291195.930	13736.127		

The difference in the mean values among the different levels of Cd conc is greater than would be expected by chance after allowing for effects of differences in type. There is a statistically significant difference (P = 0.003). To isolate which group(s) differ from the others use a multiple comparison procedure.

The difference in the mean values among the different levels of type is greater than would be expected by chance after allowing for effects of differences in Cd conc. There is a statistically significant difference (P = <0.001). To isolate which group(s) differ from the others use a multiple comparison procedure.

The effect of different levels of Cd conc does not depend on what level of type is present. There is not a statistically significant interaction between Cd conc and type. (P = 0.625)

Power of performed test with alpha = 0.0500: for Cd conc : 0.855
Power of performed test with alpha = 0.0500: for type : 0.953
Power of performed test with alpha = 0.0500: for Cd conc x type : 0.0500

All Pairwise Multiple Comparison Procedures (Holm-Sidak method):
Overall significance level = 0.05

Comparisons for factor: **Cd conc**

**Comparison**	**Diff of Means**	**t**	**P**	**P<0.050**
140 vs. 50	141.303	2.925	0.192	No
270 vs. 50	136.004	2.815	0.247	No
550 vs. 50	133.551	2.765	0.274	No
1270 vs. 50	132.608	2.745	0.281	No
140 vs. 0.5	131.607	2.669	0.326	No
270 vs. 0.5	126.308	2.562	0.401	No
27500 vs. 50	129.194	2.522	0.426	No
3100 vs. 50	121.739	2.520	0.419	No
550 vs. 0.5	123.855	2.512	0.417	No
1270 vs. 0.5	122.912	2.493	0.424	No
140 vs. 10	124.868	2.484	0.423	No
140 vs. 20	118.653	2.456	0.436	No
270 vs. 10	119.569	2.378	0.493	No
270 vs. 20	113.354	2.347	0.509	No
550 vs. 10	117.117	2.329	0.513	No
1270 vs. 10	116.173	2.311	0.518	No
550 vs. 20	110.902	2.296	0.519	No
27500 vs. 0.5	119.498	2.290	0.511	No
1270 vs. 20	109.958	2.276	0.511	No
3100 vs. 0.5	112.043	2.273	0.501	No
27500 vs. 10	112.759	2.124	0.616	No
3100 vs. 10	105.305	2.094	0.626	No
27500 vs. 20	106.544	2.079	0.623	No
3100 vs. 20	99.090	2.051	0.631	No
20 vs. 50	22.649	0.469	1.000	No
140 vs. 3100	19.563	0.405	1.000	No
10 vs. 50	16.434	0.327	1.000	No
270 vs. 3100	14.265	0.295	1.000	No
20 vs. 0.5	12.954	0.263	1.000	No

550 vs. 3100	11.812	0.245	1.000	No
140 vs. 27500	12.109	0.236	1.000	No
1270 vs. 3100	10.869	0.225	1.000	No
0.5 vs. 50	9.696	0.197	1.000	No
140 vs. 1270	8.695	0.180	1.000	No
140 vs. 550	7.751	0.160	1.000	No
27500 vs. 3100	7.455	0.145	1.000	No
270 vs. 27500	6.810	0.133	1.000	No
10 vs. 0.5	6.739	0.132	1.000	No
20 vs. 10	6.215	0.124	1.000	No
140 vs. 270	5.299	0.110	1.000	No
550 vs. 27500	4.357	0.0850	1.000	No
270 vs. 1270	3.396	0.0703	1.000	No
1270 vs. 27500	3.414	0.0666	1.000	No
270 vs. 550	2.453	0.0508	0.998	No
550 vs. 1270	0.943	0.0195	0.984	No

Comparisons for factor: **type**

**Comparison**	**Diff of Means**	**t**	**P**	**P<0.050**
Barrel vs. pot_w5	105.536	3.740	0.001	Yes
Barrel vs. pot_w10	77.489	3.224	0.004	Yes
pot_w10 vs. pot_w5	28.048	0.973	0.334	No

Elements in the nutrient solutions - Zinc

Two Way Analysis of Variance
General Linear Model
Dependent Variable: Zn
Normality Test (Shapiro-Wilk) Failed (P < 0.050)
Equal Variance Test: Failed (P < 0.050)

**Source of Variation**	**DF**	**SS**	**MS**	**F**	**P**
Cd conc	9	238661.832	26517.981	37.790	<0.001
type	2	21597.807	10798.904	15.389	<0.001
Cd conc x type	18	130399.607	7244.423	10.324	<0.001
Residual	65	45612.363	701.729		
Total	94	435585.332	4633.887		

Main effects cannot be properly interpreted if significant interaction is determined. This is because the size of a factor's effect depends upon the level of the other factor.

The effect of different levels of Cd conc depends on what level of type is present. There is a statistically significant interaction between Cd conc and type. (P = <0.001)

Power of performed test with alpha = 0.0500: for Cd conc : 1.000
Power of performed test with alpha = 0.0500: for type : 0.999
Power of performed test with alpha = 0.0500: for Cd conc x type : 1.000

All Pairwise Multiple Comparison Procedures (Holm-Sidak method):
Overall significance level = 0.05

Comparisons for factor: **Cd conc**

**Comparison**	**Diff of Means**	**t**	**P**	**P<0.050**
3100 vs. 50	131.271	10.512	<0.001	Yes
3100 vs. 20	129.651	10.382	<0.001	Yes
3100 vs. 10	130.951	10.075	<0.001	Yes
3100 vs. 0.5	124.119	9.739	<0.001	Yes

27500 vs. 50	120.396	9.090	<0.001	Yes
27500 vs. 20	118.776	8.968	<0.001	Yes
27500 vs. 10	120.076	8.748	<0.001	Yes
270 vs. 50	107.677	8.623	<0.001	Yes
270 vs. 20	106.057	8.493	<0.001	Yes
27500 vs. 0.5	113.244	8.396	<0.001	Yes
270 vs. 10	107.357	8.260	<0.001	Yes
550 vs. 50	100.172	8.022	<0.001	Yes
550 vs. 20	98.551	7.892	<0.001	Yes
270 vs. 0.5	100.525	7.887	<0.001	Yes
140 vs. 50	97.509	7.808	<0.001	Yes
550 vs. 10	99.852	7.682	<0.001	Yes
140 vs. 20	95.888	7.679	<0.001	Yes
140 vs. 10	97.189	7.478	<0.001	Yes
550 vs. 0.5	93.019	7.298	<0.001	Yes
1270 vs. 50	90.454	7.244	<0.001	Yes
1270 vs. 20	88.834	7.114	<0.001	Yes
140 vs. 0.5	90.356	7.089	<0.001	Yes
1270 vs. 10	90.134	6.935	<0.001	Yes
1270 vs. 0.5	83.302	6.536	<0.001	Yes
3100 vs. 1270	40.817	3.269	0.036	Yes
3100 vs. 140	33.762	2.704	0.161	No
3100 vs. 550	31.099	2.490	0.254	No
27500 vs. 1270	29.942	2.261	0.391	No
3100 vs. 270	23.594	1.889	0.671	No
27500 vs. 140	22.888	1.728	0.774	No
27500 vs. 550	20.224	1.527	0.880	No
270 vs. 1270	17.223	1.379	0.929	No
27500 vs. 270	12.719	0.960	0.996	No
3100 vs. 27500	10.875	0.821	0.998	No
270 vs. 140	10.169	0.814	0.997	No
550 vs. 1270	9.718	0.778	0.997	No
270 vs. 550	7.505	0.601	0.999	No
140 vs. 1270	7.055	0.565	0.999	No
0.5 vs. 50	7.153	0.561	0.998	No
0.5 vs. 10	6.833	0.516	0.996	No
0.5 vs. 20	5.532	0.434	0.996	No
550 vs. 140	2.663	0.213	0.999	No
20 vs. 50	1.621	0.130	0.999	No
20 vs. 10	1.301	0.100	0.994	No
10 vs. 50	0.320	0.0246	0.980	No

Comparisons for factor: **type**

**Comparison**	**Diff of Means**	**t**	**P**	**P<0.050**
Barrel vs. pot_w5	38.113	5.225	<0.001	Yes
pot_w10 vs. pot_w5	35.752	4.797	<0.001	Yes
Barrel vs. pot_w10	2.362	0.380	0.705	No

Elements in the nutrient solutions - Calcium

Two Way Analysis of Variance
General Linear Model
Dependent Variable: Ca
Normality Test (Shapiro-Wilk) Failed (P < 0.050)
Equal Variance Test: Passed (P = 0.286)

**Source of Variation**	**DF**	**SS**	**MS**	**F**	**P**
Cd conc	9	278424.861	30936.096	6.130	<0.001
type	2	50857.645	25428.823	5.039	0.012

Cd conc x type	18	165583.095	9199.061	1.823	0.063
Residual	35	176622.878	5046.368		
Total	64	702263.682	10972.870		

The difference in the mean values among the different levels of Cd conc is greater than would be expected by chance after allowing for effects of differences in type. There is a statistically significant difference (P = <0.001). To isolate which group(s) differ from the others use a multiple comparison procedure.

The difference in the mean values among the different levels of type is greater than would be expected by chance after allowing for effects of differences in Cd conc. There is a statistically significant difference (P = 0.012). To isolate which group(s) differ from the others use a multiple comparison procedure.

The effect of different levels of Cd conc does not depend on what level of type is present. There is not a statistically significant interaction between Cd conc and type. (P = 0.063)

Power of performed test with alpha = 0.0500: for Cd conc : 0.998
Power of performed test with alpha = 0.0500: for type : 0.683
Power of performed test with alpha = 0.0500: for Cd conc x type : 0.442

All Pairwise Multiple Comparison Procedures (Holm-Sidak method):
Overall significance level = 0.05

Comparisons for factor: **Cd conc**

**Comparison**	**Diff of Means**	**t**	**P**	**P<0.050**
27500 vs. 10	223.392	4.717	0.002	Yes
550 vs. 10	214.837	4.685	0.002	Yes
3100 vs. 10	201.599	4.396	0.004	Yes
1270 vs. 10	192.415	4.196	0.007	Yes
270 vs. 10	179.122	3.906	0.017	Yes
140 vs. 10	173.252	3.778	0.023	Yes
27500 vs. 50	168.779	3.681	0.030	Yes
550 vs. 50	160.224	3.617	0.035	Yes
27500 vs. 20	152.241	3.320	0.075	No
3100 vs. 50	146.986	3.318	0.074	No
550 vs. 20	143.686	3.243	0.087	No
27500 vs. 0.5	148.900	3.212	0.092	No
550 vs. 0.5	140.346	3.131	0.109	No
1270 vs. 50	137.802	3.111	0.112	No
3100 vs. 20	130.448	2.945	0.163	No
3100 vs. 0.5	127.107	2.836	0.203	No
270 vs. 50	124.509	2.811	0.209	No
1270 vs. 20	121.264	2.737	0.238	No
140 vs. 50	118.639	2.678	0.262	No
1270 vs. 0.5	117.923	2.631	0.280	No
270 vs. 20	107.971	2.437	0.397	No
270 vs. 0.5	104.630	2.334	0.461	No
140 vs. 20	102.101	2.305	0.470	No
140 vs. 0.5	98.760	2.203	0.535	No
0.5 vs. 10	74.492	1.607	0.927	No
20 vs. 10	71.151	1.552	0.938	No
50 vs. 10	54.613	1.191	0.995	No
27500 vs. 140	50.139	1.093	0.997	No
27500 vs. 270	44.270	0.965	0.999	No
550 vs. 140	41.585	0.939	0.999	No
550 vs. 270	35.716	0.806	1.000	No
27500 vs. 1270	30.977	0.676	1.000	No
3100 vs. 140	28.347	0.640	1.000	No
3100 vs. 270	22.477	0.507	1.000	No
550 vs. 1270	22.422	0.506	1.000	No
27500 vs. 3100	21.793	0.475	1.000	No
0.5 vs. 50	19.879	0.443	1.000	No

1270 vs. 140	19.163	0.433	1.000	No
20 vs. 50	16.538	0.373	1.000	No
1270 vs. 270	13.293	0.300	1.000	No
550 vs. 3100	13.239	0.299	0.999	No
3100 vs. 1270	9.184	0.207	0.999	No
27500 vs. 550	8.554	0.187	0.997	No
270 vs. 140	5.870	0.132	0.989	No
0.5 vs. 20	3.341	0.0745	0.941	No

Comparisons for factor: **type**

**Comparison**	**Diff of Means**	**t**	**P**	**P<0.050**
Barrel vs. pot_w5	81.724	2.970	0.016	Yes
pot_w10 vs. pot_w5	74.639	2.905	0.013	Yes
Barrel vs. pot_w10	7.085	0.351	0.728	No

Elements in the nutrient solutions - Potassium

Two Way Analysis of Variance
General Linear Model
Dependent Variable: K
Normality Test (Shapiro-Wilk) Failed (P < 0.050)
Equal Variance Test: Passed (P = 0.923)

**Source of Variation**	**DF**	**SS**	**MS**	**F**	**P**
Cd conc	9	117696.810	13077.423	0.491	0.873
type	1	6331.544	6331.544	0.238	0.628
Cd conc x type	9	126408.333	14045.370	0.528	0.847
Residual	44	1171346.698	26621.516		
Total	63	1456037.766	23111.711		

The difference in the mean values among the different levels of Cd conc is not great enough to exclude the possibility that the difference is just due to random sampling variability after allowing for the effects of differences in type. There is not a statistically significant difference (P = 0.873).

The difference in the mean values among the different levels of type is not great enough to exclude the possibility that the difference is just due to random sampling variability after allowing for the effects of differences in Cd conc. There is not a statistically significant difference (P = 0.628).

The effect of different levels of Cd conc does not depend on what level of type is present. There is not a statistically significant interaction between Cd conc and type. (P = 0.847)

Power of performed test with alpha = 0.0500: for Cd conc : 0.0500
Power of performed test with alpha = 0.0500: for type : 0.0500
Power of performed test with alpha = 0.0500: for Cd conc x type : 0.0500

Elements in the nutrient solutions - Magnesium

Two Way Analysis of Variance
General Linear Model
Dependent Variable: Mg
Normality Test (Shapiro-Wilk) Failed (P < 0.050)
Equal Variance Test: Failed (P < 0.050)

**Source of Variation**	**DF**	**SS**	**MS**	**F**	**P**
Cd conc	9	72091.213	8010.135	4.000	0.001

type	2	9821.951	4910.976	2.452	0.101
Cd conc x type	18	39749.380	2208.299	1.103	0.389
Residual	35	70086.650	2002.476		
Total	64	201661.616	3150.963		

The difference in the mean values among the different levels of Cd conc is greater than would be expected by chance after allowing for effects of differences in type. There is a statistically significant difference (P = 0.001). To isolate which group(s) differ from the others use a multiple comparison procedure.

The difference in the mean values among the different levels of type is not great enough to exclude the possibility that the difference is just due to random sampling variability after allowing for the effects of differences in Cd conc. There is not a statistically significant difference (P = 0.101).

The effect of different levels of Cd conc does not depend on what level of type is present. There is not a statistically significant interaction between Cd conc and type. (P = 0.389)

Power of performed test with alpha = 0.0500: for Cd conc : 0.929
Power of performed test with alpha = 0.0500: for type : 0.279
Power of performed test with alpha = 0.0500: for Cd conc x type : 0.0805

All Pairwise Multiple Comparison Procedures (Holm-Sidak method):
Overall significance level = 0.05

Comparisons for factor: **Cd conc**

**Comparison**	**Diff of Means**	**t**	**P**	**P<0.050**
550 vs. 10	117.836	4.079	0.011	Yes
3100 vs. 10	108.608	3.760	0.027	Yes
1270 vs. 10	103.777	3.593	0.042	Yes
270 vs. 10	97.739	3.384	0.072	No
27500 vs. 10	99.255	3.327	0.082	No
140 vs. 10	94.389	3.268	0.093	No
550 vs. 50	89.740	3.216	0.104	No
3100 vs. 50	80.512	2.885	0.224	No
550 vs. 20	77.901	2.792	0.269	No
1270 vs. 50	75.682	2.712	0.311	No
270 vs. 50	69.643	2.496	0.460	No
27500 vs. 50	71.160	2.464	0.476	No
3100 vs. 20	68.673	2.461	0.468	No
140 vs. 50	66.294	2.376	0.527	No
550 vs. 0.5	64.930	2.300	0.579	No
1270 vs. 20	63.843	2.288	0.577	No
270 vs. 20	57.804	2.071	0.743	No
27500 vs. 20	59.321	2.054	0.744	No
3100 vs. 0.5	55.702	1.973	0.792	No
140 vs. 20	54.455	1.951	0.795	No
0.5 vs. 10	52.905	1.812	0.871	No
1270 vs. 0.5	50.872	1.802	0.866	No
270 vs. 0.5	44.834	1.588	0.949	No
27500 vs. 0.5	46.350	1.587	0.942	No
140 vs. 0.5	41.484	1.469	0.968	No
20 vs. 10	39.935	1.383	0.979	No
50 vs. 10	28.096	0.973	1.000	No
0.5 vs. 50	24.809	0.879	1.000	No
550 vs. 140	23.446	0.840	1.000	No
550 vs. 270	20.097	0.720	1.000	No
550 vs. 27500	18.580	0.643	1.000	No
3100 vs. 140	14.218	0.510	1.000	No
550 vs. 1270	14.058	0.504	1.000	No
0.5 vs. 20	12.971	0.459	1.000	No
20 vs. 50	11.839	0.424	1.000	No
3100 vs. 270	10.869	0.389	1.000	No

1270 vs. 140	9.388	0.336	1.000	No
550 vs. 3100	9.228	0.331	1.000	No
3100 vs. 27500	9.352	0.324	1.000	No
1270 vs. 270	6.039	0.216	1.000	No
3100 vs. 1270	4.830	0.173	1.000	No
27500 vs. 140	4.866	0.168	1.000	No
1270 vs. 27500	4.522	0.157	0.998	No
270 vs. 140	3.349	0.120	0.991	No
27500 vs. 270	1.516	0.0525	0.958	No

Comparisons for factor: **type**

**Comparison**	**Diff of Means**	**t**	**P**	**P<0.050**
Barrel vs. pot_w5	38.282	2.209	0.098	No
pot_w10 vs. pot_w5	27.345	1.689	0.190	No
Barrel vs. pot_w10	10.937	0.860	0.396	No

Elements in the nutrient solutions - Phosphate

Two Way Analysis of Variance
General Linear Model
Dependent Variable: P
Normality Test (Shapiro-Wilk) Failed (P < 0.050)
Equal Variance Test: Passed (P = 0.789)

Source of Variation	DF	SS	MS	F	P
Cd conc	9	509565.067	56618.341	40.239	<0.001
type	2	177928.334	88964.167	63.227	<0.001
Cd conc x type	18	283362.727	15742.374	11.188	<0.001
Residual	65	91458.452	1407.053		
Total	94	984232.613	10470.560		

Main effects cannot be properly interpreted if significant interaction is determined. This is because the size of a factor's effect depends upon the level of the other factor.

The effect of different levels of Cd conc depends on what level of type is present. There is a statistically significant interaction between Cd conc and type. (P = <0.001)

Power of performed test with alpha = 0.0500: for Cd conc : 1.000
Power of performed test with alpha = 0.0500: for type : 1.000
Power of performed test with alpha = 0.0500: for Cd conc x type : 1.000

All Pairwise Multiple Comparison Procedures (Holm-Sidak method):
Overall significance level = 0.05

Comparisons for factor: Cd conc

**Comparison**	**Diff of Means**	**t**	**P**	**P<0.050**
3100 vs. 50	173.608	9.818	<0.001	Yes
550 vs. 50	173.010	9.784	<0.001	Yes
3100 vs. 5.000	177.007	9.617	<0.001	Yes
550 vs. 5.000	176.409	9.585	<0.001	Yes
1270 vs. 50	167.785	9.489	<0.001	Yes
1270 vs. 5.000	171.183	9.301	<0.001	Yes
27500 vs. 50	168.215	8.969	<0.001	Yes
3100 vs. 0.5	161.814	8.966	<0.001	Yes
550 vs. 0.5	161.215	8.933	<0.001	Yes
27500 vs. 5.000	171.614	8.829	<0.001	Yes
3100 vs. 20	153.980	8.708	<0.001	Yes
550 vs. 20	153.382	8.674	<0.001	Yes

1270 vs. 0.5	155.990	8.643	<0.001	Yes
270 vs. 50	151.115	8.546	<0.001	Yes
270 vs. 5.000	154.513	8.395	<0.001	Yes
1270 vs. 20	148.157	8.379	<0.001	Yes
140 vs. 50	146.492	8.284	<0.001	Yes
27500 vs. 0.5	156.420	8.190	<0.001	Yes
140 vs. 5.000	149.891	8.144	<0.001	Yes
27500 vs. 20	148.587	7.922	<0.001	Yes
270 vs. 0.5	139.320	7.720	<0.001	Yes
140 vs. 0.5	134.698	7.464	<0.001	Yes
270 vs. 20	131.487	7.436	<0.001	Yes
140 vs. 20	126.865	7.174	<0.001	Yes
3100 vs. 140	27.116	1.533	0.946	No
550 vs. 140	26.518	1.500	0.949	No
3100 vs. 270	22.493	1.272	0.988	No
20 vs. 5.000	23.027	1.251	0.987	No
550 vs. 270	21.895	1.238	0.985	No
1270 vs. 140	21.292	1.204	0.986	No
27500 vs. 140	21.722	1.158	0.987	No
20 vs. 50	19.628	1.110	0.988	No
1270 vs. 270	16.670	0.943	0.996	No
27500 vs. 270	17.100	0.912	0.996	No
0.5 vs. 5.000	15.193	0.810	0.998	No
0.5 vs. 50	11.794	0.654	0.999	No
20 vs. 0.5	7.833	0.434	1.000	No
3100 vs. 1270	5.823	0.329	1.000	No
550 vs. 1270	5.225	0.296	1.000	No
3100 vs. 27500	5.393	0.288	1.000	No
270 vs. 140	4.622	0.261	1.000	No
550 vs. 27500	4.795	0.256	0.998	No
50 vs. 5.000	3.399	0.185	0.997	No
3100 vs. 550	0.598	0.0338	0.999	No
27500 vs. 1270	0.430	0.0229	0.982	No

Comparisons for factor: type

**Comparison**	**Diff of Means**	**t**	**P**	**P<0.050**
Barrel vs. pot_w10	85.153	9.680	<0.001	Yes
Barrel vs. pot_w5	94.180	9.117	<0.001	Yes
pot_w10 vs. pot_w5	9.027	0.855	0.396	No

Elements in the nutrient solutions - Sulphate

Two Way Analysis of Variance
General Linear Model
Dependent Variable: S
Normality Test (Shapiro-Wilk) Failed (P < 0.050)
Equal Variance Test: Passed (P = 0.179)

**Source of Variation**	**DF**	**SS**	**MS**	**F**	**P**
Cd conc	9	54351.443	6039.049	0.350	0.954
type	2	423610.302	211805.151	12.288	<0.001
Cd conc x type	18	364972.410	20276.245	1.176	0.306
Residual	65	1120370.524	17236.470		
Total	94	1940504.439	20643.664		

The difference in the mean values among the different levels of Cd conc is not great enough to exclude the possibility that the difference is just due to random sampling variability after allowing for the effects of differences

in type. There is not a statistically significant difference (P = 0.954).

The difference in the mean values among the different levels of type is greater than would be expected by chance after allowing for effects of differences in Cd conc. There is a statistically significant difference (P = <0.001). To isolate which group(s) differ from the others use a multiple comparison procedure.

The effect of different levels of Cd conc does not depend on what level of type is present. There is not a statistically significant interaction between Cd conc and type. (P = 0.306)

Power of performed test with alpha = 0.0500: for Cd conc : 0.0500
Power of performed test with alpha = 0.0500: for type : 0.995
Power of performed test with alpha = 0.0500: for Cd conc x type : 0.117

Comparisons for factor: **type**

**Comparison**	**Diff of Means**	**t**	**P**	**P<0.050**
pot_w5 vs. Barrel	173.335	4.794	<0.001	Yes
pot_w10 vs. Barrel	94.635	3.074	0.006	Yes
pot_w5 vs. pot_w10	78.700	2.130	0.037	Yes

----

3) Elements in the plant tissues

Elements in the plant tissues - Cadmium - Harvested Leaves

Two Way Analysis of Variance
General Linear Model (No Interactions)
Dependent Variable: Cd in leaves
Normality Test (Shapiro-Wilk) Failed (P < 0.050)
Equal Variance Test: Failed (P < 0.050)

**Source of Variation**	**DF**	**SS**	**MS**	**F**	**P**
Exp	1	89.256	89.256	0.649	0.424
Cd conc	9	22353.923	2483.769	18.057	<0.001
Residual	59	8115.675	137.554		
Total	69	30656.789	444.301		

The difference in the mean values among the different levels of Exp is not great enough to exclude the possibility that the difference is just due to random sampling variability after allowing for the effects of differences in Cd conc. There is not a statistically significant difference (P = 0.424).

The difference in the mean values among the different levels of Cd conc is greater than would be expected by chance after allowing for effects of differences in Exp. There is a statistically significant difference (P = <0.001). To isolate which group(s) differ from the others use a multiple comparison procedure.

Power of performed test with alpha = 0.0500: for Exp : 0.0500
Power of performed test with alpha = 0.0500: for Cd conc : 1.000

Least square means for Exp :

**Group**	**Mean**	**SEM**
272.000	18.559	2.206
273.000	16.218	1.881

Least square means for Cd conc :

**Group**	**Mean**	**SEM**
0.5	0.129	4.147
20	3.812	4.147
50	4.666	4.438

140	13.977	4.438
270	14.864	4.438
550	26.678	4.438
1270	42.443	4.438
3100	54.541	4.147
27500	10.724	4.438
10	2.055	6.042

All Pairwise Multiple Comparison Procedures (Holm-Sidak method):
Overall significance level = 0.05

Comparisons for factor: **Cd conc**

**Comparison**	**Diff of Means**	**t**	**P**	**P<0.050**
3100 vs. 0.5	54.411	9.279	<0.001	Yes
3100 vs. 20	50.729	8.651	<0.001	Yes
3100 vs. 50	49.874	8.212	<0.001	Yes
3100 vs. 27500	43.817	7.214	<0.001	Yes
3100 vs. 10	52.486	7.163	<0.001	Yes
1270 vs. 0.5	42.314	6.967	<0.001	Yes
3100 vs. 140	40.564	6.679	<0.001	Yes
3100 vs. 270	39.677	6.533	<0.001	Yes
1270 vs. 20	38.631	6.361	<0.001	Yes
1270 vs. 50	37.777	6.019	<0.001	Yes
1270 vs. 10	40.389	5.388	<0.001	Yes
1270 vs. 27500	31.719	5.054	<0.001	Yes
3100 vs. 550	27.862	4.588	<0.001	Yes
1270 vs. 140	28.466	4.536	<0.001	Yes
1270 vs. 270	27.580	4.394	0.001	Yes
550 vs. 0.5	26.549	4.371	0.002	Yes
550 vs. 20	22.867	3.765	0.011	Yes
550 vs. 50	22.012	3.507	0.024	Yes
550 vs. 10	24.624	3.285	0.045	Yes
550 vs. 27500	15.954	2.542	0.301	No
1270 vs. 550	15.765	2.512	0.311	No
270 vs. 0.5	14.734	2.426	0.359	No
140 vs. 0.5	13.848	2.280	0.457	No
550 vs. 140	12.701	2.024	0.657	No
3100 vs. 1270	12.097	1.992	0.667	No
550 vs. 270	11.815	1.883	0.738	No
270 vs. 20	11.052	1.820	0.767	No
27500 vs. 0.5	10.595	1.744	0.803	No
270 vs. 10	12.809	1.709	0.809	No
140 vs. 20	10.165	1.674	0.813	No
270 vs. 50	10.197	1.625	0.825	No
140 vs. 10	11.923	1.590	0.825	No
140 vs. 50	9.311	1.484	0.866	No
27500 vs. 10	8.669	1.156	0.969	No
27500 vs. 20	6.912	1.138	0.963	No
27500 vs. 50	6.057	0.965	0.984	No
50 vs. 0.5	4.537	0.747	0.996	No
270 vs. 27500	4.140	0.660	0.997	No
20 vs. 0.5	3.682	0.628	0.995	No
140 vs. 27500	3.253	0.518	0.996	No
50 vs. 10	2.612	0.348	0.999	No
10 vs. 0.5	1.925	0.263	0.998	No
20 vs. 10	1.757	0.240	0.993	No
270 vs. 140	0.887	0.141	0.987	No
50 vs. 20	0.855	0.141	0.889	No

Elements in the plant tissues - Cadmium - Stems

Two Way Analysis of Variance
General Linear Model (No Interactions)
Dependent Variable: Cd stems
Normality Test (Shapiro-Wilk) Failed (P < 0.050)
Equal Variance Test: Failed (P < 0.050)

**Source of Variation**	**DF**	**SS**	**MS**	**F**	**P**
Exp	3	822257.892	274085.964	1.230	0.302
Cd conc	9	11961694.419	1329077.158	5.966	<0.001
Residual	127	28294054.379	222787.830		
Total	139	41174831.396	296221.809		

The difference in the mean values among the different levels of Exp is not great enough to exclude the possibility that the difference is just due to random sampling variability after allowing for the effects of differences in Cd conc. There is not a statistically significant difference (P = 0.302).

The difference in the mean values among the different levels of Cd conc is greater than would be expected by chance after allowing for effects of differences in Exp. There is a statistically significant difference (P = <0.001). To isolate which group(s) differ from the others use a multiple comparison procedure.

Power of performed test with alpha = 0.0500: for Exp : 0.0905
Power of performed test with alpha = 0.0500: for Cd conc : 1.000

Least square means for Exp :

**Group**	**Mean**	**SEM**
272.000	135.916	81.393
273.000	128.964	74.630
278.000	324.022	93.191
280.000	115.008	76.711

Least square means for Cd conc :

**Group**	**Mean**	**SEM**
0.5	0.0150	118.001
20	3.830	118.001
50	4.265	118.001
140	81.413	126.635
270	126.443	138.694
550	134.436	126.635
1270	156.708	126.282
3100	297.477	137.452
27500	960.583	118.001
10	-5.394	151.178

All Pairwise Multiple Comparison Procedures (Holm-Sidak method):
Overall significance level = 0.05

Comparisons for factor: **Exp**

**Comparison**	**Diff of Means**	**t**	**P**	**P<0.050**
278.000 vs. 280.000	209.015	1.732	0.416	No
278.000 vs. 273.000	195.059	1.634	0.425	No
278.000 vs. 272.000	188.106	1.520	0.430	No
272.000 vs. 280.000	20.908	0.187	0.997	No
273.000 vs. 280.000	13.956	0.130	0.989	No
272.000 vs. 273.000	6.952	0.0630	0.950	No

Comparisons for factor: **Cd conc**

**Comparison**	**Diff of Means**	**t**	**P**	**P<0.050**
27500 vs. 0.5	960.568	5.756	<0.001	Yes
27500 vs. 20	956.753	5.733	<0.001	Yes
27500 vs. 50	956.318	5.731	<0.001	Yes
27500 vs. 140	879.170	5.079	<0.001	Yes

27500 vs. 10	965.977	5.037	<0.001	Yes
27500 vs. 550	826.148	4.773	<0.001	Yes
27500 vs. 1270	803.875	4.651	<0.001	Yes
27500 vs. 270	834.140	4.581	<0.001	Yes
27500 vs. 3100	663.106	3.660	0.014	Yes
3100 vs. 0.5	297.462	1.642	0.980	No
3100 vs. 20	293.647	1.621	0.981	No
3100 vs. 50	293.212	1.619	0.979	No
3100 vs. 10	302.871	1.482	0.993	No
3100 vs. 140	216.064	1.156	1.000	No
1270 vs. 0.5	156.693	0.907	1.000	No
1270 vs. 20	152.878	0.885	1.000	No
1270 vs. 50	152.443	0.882	1.000	No
3100 vs. 270	171.034	0.876	1.000	No
3100 vs. 550	163.041	0.872	1.000	No
1270 vs. 10	162.102	0.823	1.000	No
550 vs. 0.5	134.421	0.777	1.000	No
550 vs. 20	130.606	0.755	1.000	No
3100 vs. 1270	140.769	0.754	1.000	No
550 vs. 50	130.170	0.752	1.000	No
550 vs. 10	139.829	0.709	1.000	No
270 vs. 0.5	126.428	0.694	1.000	No
270 vs. 20	122.613	0.673	1.000	No
270 vs. 50	122.177	0.671	1.000	No
270 vs. 10	131.837	0.643	1.000	No
140 vs. 0.5	81.398	0.470	1.000	No
140 vs. 20	77.583	0.448	1.000	No
140 vs. 50	77.148	0.446	1.000	No
140 vs. 10	86.807	0.440	1.000	No
1270 vs. 140	75.295	0.421	1.000	No
550 vs. 140	53.022	0.296	1.000	No
270 vs. 140	45.030	0.240	1.000	No
1270 vs. 270	30.265	0.161	1.000	No
1270 vs. 550	22.273	0.125	1.000	No
50 vs. 10	9.659	0.0504	1.000	No
20 vs. 10	9.224	0.0481	1.000	No
550 vs. 270	7.993	0.0426	1.000	No
0.5 vs. 10	5.409	0.0282	1.000	No
50 vs. 0.5	4.250	0.0255	1.000	No
20 vs. 0.5	3.815	0.0229	1.000	No
50 vs. 20	0.436	0.00261	0.998	No

Elements in the plant tissues - Cadmium- w5 leaves

Two Way Analysis of Variance
General Linear Model (No Interactions)
Dependent Variable: Cd w5
Normality Test (Shapiro-Wilk) Failed (P < 0.050)
Equal Variance Test: Failed (P < 0.050)

**Source of Variation**	**DF**	**SS**	**MS**	**F**	**P**
Exp	3	181.560	60.520	1.040	0.381
Cd conc	9	5388.909	598.768	10.285	<0.001
Residual	62	3609.537	58.218		
Total	74	9184.768	124.118		

The difference in the mean values among the different levels of Exp is not great enough to exclude the possibility that the difference is just due to random sampling variability after allowing for the effects of differences in Cd conc. There is not a statistically significant difference (P = 0.381).

The difference in the mean values among the different levels of Cd conc is greater than would be expected by chance after allowing for effects of differences in Exp. There is a statistically significant difference (P = <0.001). To isolate which group(s) differ from the others use a multiple comparison procedure.

Power of performed test with alpha = 0.0500: for Exp : 0.0557
Power of performed test with alpha = 0.0500: for Cd conc : 1.000

Least square means for Exp :

**Group**	**Mean**	**SEM**
272.000	11.498	1.828
273.000	12.207	1.706
278.000	13.733	1.759
280.000	9.359	1.834

Least square means for Cd conc :

**Group**	**Mean**	**SEM**
0.5	0.0501	2.698
20	2.402	2.698
50	4.326	2.698
140	17.815	2.698
270	20.183	2.698
550	17.123	2.698
1270	23.175	2.698
3100	19.058	2.698
27500	11.165	3.465
10	1.694	3.159

All Pairwise Multiple Comparison Procedures (Holm-Sidak method):
Overall significance level = 0.05

Comparisons for factor: **Exp**

**Comparison**	**Diff of Means**	**t**	**P**	**P<0.050**
278.000 vs. 280.000	4.373	1.721	0.433	No
273.000 vs. 280.000	2.847	1.137	0.778	No
278.000 vs. 272.000	2.235	0.881	0.854	No
272.000 vs. 280.000	2.139	0.826	0.797	No
278.000 vs. 273.000	1.526	0.623	0.785	No
273.000 vs. 272.000	0.709	0.283	0.778	No

Comparisons for factor: **Cd conc**

**Comparison**	**Diff of Means**	**t**	**P**	**P<0.050**
1270 vs. 0.5	23.125	6.062	<0.001	Yes
1270 vs. 20	20.773	5.445	<0.001	Yes
270 vs. 0.5	20.133	5.277	<0.001	Yes
1270 vs. 10	21.481	5.171	<0.001	Yes
3100 vs. 0.5	19.008	4.982	<0.001	Yes
1270 vs. 50	18.849	4.941	<0.001	Yes
270 vs. 20	17.781	4.661	<0.001	Yes
140 vs. 0.5	17.765	4.657	<0.001	Yes
550 vs. 0.5	17.073	4.475	0.001	Yes
270 vs. 10	18.488	4.451	0.001	Yes
3100 vs. 20	16.656	4.366	0.002	Yes
3100 vs. 10	17.364	4.180	0.003	Yes
270 vs. 50	15.857	4.156	0.003	Yes
140 vs. 20	15.413	4.040	0.005	Yes
140 vs. 10	16.120	3.881	0.008	Yes
3100 vs. 50	14.732	3.862	0.008	Yes
550 vs. 20	14.721	3.859	0.008	Yes
550 vs. 10	15.429	3.714	0.012	Yes
140 vs. 50	13.489	3.536	0.021	Yes
550 vs. 50	12.797	3.354	0.035	Yes

1270 vs. 27500	12.010	2.735	0.184	No
27500 vs. 0.5	11.115	2.531	0.286	No
270 vs. 27500	9.018	2.054	0.647	No
27500 vs. 10	9.471	2.020	0.659	No
27500 vs. 20	8.763	1.996	0.662	No
3100 vs. 27500	7.893	1.797	0.799	No
1270 vs. 550	6.052	1.586	0.907	No
27500 vs. 50	6.839	1.557	0.909	No
140 vs. 27500	6.649	1.514	0.915	No
1270 vs. 140	5.360	1.405	0.944	No
550 vs. 27500	5.958	1.357	0.949	No
50 vs. 0.5	4.276	1.121	0.987	No
1270 vs. 3100	4.117	1.079	0.987	No
270 vs. 550	3.060	0.802	0.999	No
1270 vs. 270	2.992	0.784	0.998	No
50 vs. 10	2.632	0.634	0.999	No
270 vs. 140	2.368	0.621	0.999	No
20 vs. 0.5	2.352	0.616	0.998	No
3100 vs. 550	1.935	0.507	0.999	No
50 vs. 20	1.924	0.504	0.997	No
10 vs. 0.5	1.644	0.396	0.997	No
3100 vs. 140	1.243	0.326	0.996	No
270 vs. 3100	1.125	0.295	0.988	No
140 vs. 550	0.692	0.181	0.979	No
20 vs. 10	0.708	0.170	0.865	No

Elements in the plant tissues - Cadmium - w10 leaves

Two Way Analysis of Variance
General Linear Model (No Interactions)
Dependent Variable: Cd w10
Normality Test (Shapiro-Wilk) Failed (P < 0.050)
Equal Variance Test: Failed (P < 0.050)

Source of Variation	DF	SS	MS	F	P
Exp	3	453.390	151.130	2.693	0.058
Cd conc	9	2896.467	321.830	5.735	<0.001
Residual	42	2356.970	56.118		
Total	54	5672.138	105.040		

The difference in the mean values among the different levels of Exp is not great enough to exclude the possibility that the difference is just due to random sampling variability after allowing for the effects of differences in Cd conc. There is not a statistically significant difference (P = 0.058).

The difference in the mean values among the different levels of Cd conc is greater than would be expected by chance after allowing for effects of differences in Exp. There is a statistically significant difference (P = <0.001). To isolate which group(s) differ from the others use a multiple comparison procedure.

Power of performed test with alpha = 0.0500: for Exp : 0.402
Power of performed test with alpha = 0.0500: for Cd conc : 0.997

Least square means for Exp :

Group	Mean	SEM
272.000	10.485	3.024
273.000	14.963	2.111
278.000	9.146	1.993
280.000	7.596	1.978

Least square means for Cd conc :

Group	Mean	SEM

0.5	0.0868	2.851
20	2.455	2.851
50	3.213	2.851
140	14.237	2.851
270	11.485	3.080
550	15.165	3.080
1270	17.860	3.080
10	2.002	3.154
3100	26.070	5.412
27500	12.899	7.709

All Pairwise Multiple Comparison Procedures (Holm-Sidak method):
Overall significance level = 0.05

Comparisons for factor: **Exp**

**Comparison**	**Diff of Means**	**t**	**P**	**P<0.050**
273.000 vs. 280.000	7.368	2.547	0.085	No
273.000 vs. 278.000	5.818	2.004	0.233	No
273.000 vs. 272.000	4.479	1.214	0.651	No
272.000 vs. 280.000	2.889	0.799	0.813	No
278.000 vs. 280.000	1.550	0.552	0.827	No
272.000 vs. 278.000	1.339	0.370	0.713	No

Comparisons for factor: **Cd conc**

**Comparison**	**Diff of Means**	**t**	**P**	**P<0.050**
3100 vs. 0.5	25.983	4.248	0.005	Yes
1270 vs. 0.5	17.773	4.235	0.005	Yes
3100 vs. 20	23.615	3.861	0.016	Yes
3100 vs. 10	24.068	3.842	0.017	Yes
3100 vs. 50	22.857	3.737	0.023	Yes
1270 vs. 20	15.405	3.671	0.027	Yes
1270 vs. 10	15.858	3.598	0.032	Yes
550 vs. 0.5	15.079	3.593	0.032	Yes
140 vs. 0.5	14.150	3.510	0.039	Yes
1270 vs. 50	14.647	3.490	0.040	Yes
550 vs. 20	12.710	3.029	0.137	No
550 vs. 10	13.164	2.986	0.148	No
140 vs. 20	11.782	2.923	0.168	No
140 vs. 10	12.235	2.878	0.182	No
550 vs. 50	11.953	2.848	0.190	No
140 vs. 50	11.024	2.735	0.240	No
270 vs. 0.5	11.398	2.716	0.243	No
3100 vs. 270	14.585	2.342	0.493	No
270 vs. 20	9.030	2.152	0.641	No
270 vs. 10	9.483	2.151	0.627	No
270 vs. 50	8.272	1.971	0.759	No
3100 vs. 140	11.833	1.934	0.772	No
3100 vs. 550	10.904	1.751	0.877	No
27500 vs. 0.5	12.812	1.559	0.949	No
1270 vs. 270	6.375	1.464	0.968	No
3100 vs. 27500	13.171	1.398	0.976	No
3100 vs. 1270	8.210	1.318	0.984	No
27500 vs. 10	10.897	1.308	0.981	No
27500 vs. 20	10.444	1.271	0.982	No
27500 vs. 50	9.686	1.179	0.989	No
1270 vs. 140	3.623	0.863	0.999	No
550 vs. 270	3.681	0.845	0.999	No
50 vs. 0.5	3.126	0.775	0.999	No
140 vs. 270	2.752	0.656	1.000	No
1270 vs. 550	2.695	0.619	1.000	No
1270 vs. 27500	4.961	0.598	1.000	No

20 vs. 0.5	2.368	0.587	0.999	No
10 vs. 0.5	1.915	0.451	1.000	No
50 vs. 10	1.211	0.285	1.000	No
550 vs. 27500	2.267	0.273	1.000	No
550 vs. 140	0.929	0.221	1.000	No
50 vs. 20	0.758	0.188	1.000	No
27500 vs. 270	1.414	0.170	0.998	No
140 vs. 27500	1.338	0.163	0.983	No
20 vs. 10	0.453	0.107	0.916	No

Elements in the plant tissues - Cadmium - Roots

Two Way Analysis of Variance
General Linear Model (No Interactions)
Dependent Variable: Cd roots
Normality Test (Shapiro-Wilk) Failed (P < 0.050)
Equal Variance Test: Passed (P = 1.000)

Source of Variation	DF	SS	MS	F	P
Exp	3	2578890.514	859630.171	1.875	0.159
Cd conc	9	48294279.969	5366031.108	11.702	<0.001
Residual	26	11922517.741	458558.375		
Total	38	62246760.867	1638072.654		

The difference in the mean values among the different levels of Exp is not great enough to exclude the possibility that the difference is just due to random sampling variability after allowing for the effects of differences in Cd conc. There is not a statistically significant difference (P = 0.159).

The difference in the mean values among the different levels of Cd conc is greater than would be expected by chance after allowing for effects of differences in Exp. There is a statistically significant difference (P = <0.001). To isolate which group(s) differ from the others use a multiple comparison procedure.

Power of performed test with alpha = 0.0500: for Exp : 0.208
Power of performed test with alpha = 0.0500: for Cd conc : 1.000

Least square means for Exp :

Group	Mean	SEM
272.000	252.734	229.454
273.000	646.468	214.140
278.000	763.018	214.140
280.000	981.640	214.140

Least square means for Cd conc :

Group	Mean	SEM
0.5	6.012	338.585
20	25.318	338.585
50	34.957	338.585
140	457.480	338.585
270	332.654	338.585
550	698.021	338.585
1270	543.452	338.585
3100	769.285	338.585
27500	3860.137	338.585
10	-117.666	396.357

All Pairwise Multiple Comparison Procedures (Holm-Sidak method):
Overall significance level = 0.05

Comparisons for factor: **Exp**

Comparison	Diff of Means	t	P	P<0.050

280.000 vs. 272.000	728.905	2.322	0.158	No
278.000 vs. 272.000	510.284	1.626	0.460	No
273.000 vs. 272.000	393.733	1.255	0.631	No
280.000 vs. 273.000	335.172	1.107	0.624	No
280.000 vs. 278.000	218.621	0.722	0.726	No
278.000 vs. 273.000	116.550	0.385	0.703	No

Comparisons for factor: **Cd conc**

**Comparison**	**Diff of Means**	**t**	**P**	**P<0.050**
27500 vs. 0.5	3854.126	8.049	<0.001	Yes
27500 vs. 20	3834.820	8.009	<0.001	Yes
27500 vs. 50	3825.180	7.989	<0.001	Yes
27500 vs. 10	3977.804	7.631	<0.001	Yes
27500 vs. 270	3527.484	7.367	<0.001	Yes
27500 vs. 140	3402.657	7.106	<0.001	Yes
27500 vs. 1270	3316.685	6.927	<0.001	Yes
27500 vs. 550	3162.116	6.604	<0.001	Yes
27500 vs. 3100	3090.852	6.455	<0.001	Yes
3100 vs. 10	886.952	1.701	0.978	No
3100 vs. 0.5	763.274	1.594	0.990	No
550 vs. 10	815.687	1.565	0.991	No
3100 vs. 20	743.968	1.554	0.991	No
3100 vs. 50	734.328	1.534	0.991	No
550 vs. 0.5	692.010	1.445	0.996	No
550 vs. 20	672.703	1.405	0.997	No
550 vs. 50	663.064	1.385	0.997	No
1270 vs. 10	661.118	1.268	0.999	No
1270 vs. 0.5	537.441	1.122	1.000	No
140 vs. 10	575.146	1.103	1.000	No
1270 vs. 20	518.135	1.082	1.000	No
1270 vs. 50	508.495	1.062	1.000	No
140 vs. 0.5	451.469	0.943	1.000	No
3100 vs. 270	436.632	0.912	1.000	No
140 vs. 20	432.162	0.903	1.000	No
140 vs. 50	422.523	0.882	1.000	No
270 vs. 10	450.320	0.864	1.000	No
550 vs. 270	365.367	0.763	1.000	No
270 vs. 0.5	326.642	0.682	1.000	No
3100 vs. 140	311.805	0.651	1.000	No
270 vs. 20	307.336	0.642	1.000	No
270 vs. 50	297.697	0.622	1.000	No
550 vs. 140	240.541	0.502	1.000	No
3100 vs. 1270	225.833	0.472	1.000	No
1270 vs. 270	210.799	0.440	1.000	No
550 vs. 1270	154.569	0.323	1.000	No
50 vs. 10	152.623	0.293	1.000	No
20 vs. 10	142.984	0.274	1.000	No
140 vs. 270	124.827	0.261	1.000	No
0.5 vs. 10	123.678	0.237	1.000	No
1270 vs. 140	85.972	0.180	1.000	No
3100 vs. 550	71.264	0.149	1.000	No
50 vs. 0.5	28.945	0.0605	1.000	No
20 vs. 0.5	19.306	0.0403	0.999	No
50 vs. 20	9.639	0.0201	0.984	No

Elements in the plant tissues - Cadmium - Seeds

Two Way Analysis of Variance
General Linear Model (No Interactions)
Dependent Variable: Cd seeds
Normality Test (Shapiro-Wilk) Failed ($P < 0.050$)

Equal Variance Test: Failed (P < 0.050)

**Source of Variation**	**DF**	**SS**	**MS**	**F**	**P**
Exp	3	2.005	0.668	1.787	0.161
Cd conc	3	74.106	24.702	66.043	<0.001
Residual	52	19.449	0.374		
Total	58	95.897	1.653		

The difference in the mean values among the different levels of Exp is not great enough to exclude the possibility that the difference is just due to random sampling variability after allowing for the effects of differences in Cd conc. There is not a statistically significant difference (P = 0.161).

The difference in the mean values among the different levels of Cd conc is greater than would be expected by chance after allowing for effects of differences in Exp. There is a statistically significant difference (P = <0.001). To isolate which group(s) differ from the others use a multiple comparison procedure.

Power of performed test with alpha = 0.0500: for Exp : 0.202
Power of performed test with alpha = 0.0500: for Cd conc : 1.000

Least square means for Exp :

**Group**	**Mean**	**SEM**
272.000	2.066	0.184
273.000	1.675	0.153
278.000	1.661	0.158
280.000	1.526	0.153

Least square means for Cd conc :

**Group**	**Mean**	**SEM**
0.500	0.00165	0.153
10.000	1.655	0.192
20.000	2.643	0.153
50.000	2.629	0.153

All Pairwise Multiple Comparison Procedures (Holm-Sidak method):
Overall significance level = 0.05

Comparisons for factor: **Exp**

**Comparison**	**Diff of Means**	**t**	**P**	**P<0.050**
272.000 vs. 280.000	0.541	2.259	0.157	No
272.000 vs. 278.000	0.405	1.667	0.414	No
272.000 vs. 273.000	0.392	1.635	0.367	No
273.000 vs. 280.000	0.149	0.690	0.870	No
278.000 vs. 280.000	0.136	0.617	0.788	No
273.000 vs. 278.000	0.0134	0.0610	0.952	No

Comparisons for factor: **Cd conc**

**Comparison**	**Diff of Means**	**t**	**P**	**P<0.050**
20.000 vs. 0.500	2.641	12.216	<0.001	Yes
50.000 vs. 0.500	2.627	12.149	<0.001	Yes
10.000 vs. 0.500	1.653	6.742	<0.001	Yes
20.000 vs. 10.000	0.988	4.032	<0.001	Yes
50.000 vs. 10.000	0.974	3.973	<0.001	Yes
20.000 vs. 50.000	0.0145	0.0669	0.947	No

Elements in the plant tissues - Copper - Seeds

Two Way Analysis of Variance

General Linear Model (No Interactions)
Dependent Variable: Cu acc seeds
Normality Test (Shapiro-Wilk) Failed (P < 0.050)
Equal Variance Test: Passed (P = 0.946)

Source of Variation	DF	SS	MS	F	P
Exp	3	16.863	5.621	3.466	0.023
Cd conc	3	42.550	14.183	8.746	<0.001
Residual	52	84.330	1.622		
Total	58	145.243	2.504		

The difference in the mean values among the different levels of Exp is greater than would be expected by chance after allowing for effects of differences in Cd conc. There is a statistically significant difference (P = 0.023). To isolate which group(s) differ from the others use a multiple comparison procedure.

The difference in the mean values among the different levels of Cd conc is greater than would be expected by chance after allowing for effects of differences in Exp. There is a statistically significant difference (P = <0.001). To isolate which group(s) differ from the others use a multiple comparison procedure.

Power of performed test with alpha = 0.0500: for Exp : 0.575
Power of performed test with alpha = 0.0500: for Cd conc : 0.988

Least square means for Exp :

Group	Mean	SEM
272.000	7.696	0.384
273.000	7.550	0.318
278.000	6.502	0.330
280.000	6.574	0.318

Least square means for Cd conc :

Group	Mean	SEM
0.5	5.925	0.318
20	7.455	0.318
50	8.146	0.318
10	6.797	0.399

All Pairwise Multiple Comparison Procedures (Holm-Sidak method):
Overall significance level = 0.05

Comparisons for factor: **Exp**

Comparison	Diff of Means	t	P	P<0.050
272.000 vs. 278.000	1.194	2.360	0.125	No
273.000 vs. 278.000	1.047	2.286	0.125	No
272.000 vs. 280.000	1.122	2.250	0.110	No
273.000 vs. 280.000	0.976	2.167	0.101	No
272.000 vs. 273.000	0.146	0.293	0.947	No
280.000 vs. 278.000	0.0718	0.157	0.876	No

Comparisons for factor: **Cd conc**

Comparison	Diff of Means	t	P	P<0.050
50 vs. 0.5	2.221	4.934	<0.001	Yes
20 vs. 0.5	1.530	3.398	0.007	Yes
50 vs. 10	1.349	2.643	0.043	Yes
10 vs. 0.5	0.872	1.708	0.255	No
50 vs. 20	0.691	1.536	0.244	No
20 vs. 10	0.658	1.288	0.203	No

Elements in the plant tissues - Copper - w10 leaves

Two Way Analysis of Variance
General Linear Model (No Interactions)
Dependent Variable: Cu in w10
Normality Test (Shapiro-Wilk) Failed (P < 0.050)
Equal Variance Test: Failed (P < 0.050)

**Source of Variation**	**DF**	**SS**	**MS**	**F**	**P**
Exp	3	2174.446	724.815	0.710	0.551
Cd conc	8	12842.502	1605.313	1.573	0.161
Residual	43	43879.485	1020.453		
Total	54	58774.477	1088.416		

The difference in the mean values among the different levels of Exp is not great enough to exclude the possibility that the difference is just due to random sampling variability after allowing for the effects of differences in Cd conc. There is not a statistically significant difference (P = 0.551).

The difference in the mean values among the different levels of Cd conc is not great enough to exclude the possibility that the difference is just due to random sampling variability after allowing for the effects of differences in Exp. There is not a statistically significant difference (P = 0.161).

Power of performed test with alpha = 0.0500: for Exp : 0.0500
Power of performed test with alpha = 0.0500: for Cd conc : 0.228

Least square means for Exp :

**Group**	**Mean**	**SEM**
272.000	9.682	12.475
273.000	10.445	8.397
278.000	8.515	7.843
280.000	23.661	8.377

Least square means for Cd conc :

**Group**	**Mean**	**SEM**
0.5	4.168	12.155
20	4.303	12.155
50	4.231	12.155
140	51.058	12.155
270	19.211	12.155
550	16.186	13.132
1270	12.305	13.132
10	2.313	13.446
3100	3.908	23.069

Elements in the plant tissues - Copper - w5 leaves

Two Way Analysis of Variance
General Linear Model (No Interactions)
Dependent Variable: Cu in w5
Normality Test (Shapiro-Wilk) Failed (P < 0.050)
Equal Variance Test: Failed (P < 0.050)

**Source of Variation**	**DF**	**SS**	**MS**	**F**	**P**
Exp	3	1334.550	444.850	5.755	0.002
Cd conc	9	1923.803	213.756	2.765	0.009
Residual	62	4792.382	77.296		
Total	74	8494.024	114.784		

The difference in the mean values among the different levels of Exp is greater than would be expected by chance after allowing for effects of differences in Cd conc. There is a statistically significant difference (P = 0.002). To isolate which group(s) differ from the others use a multiple comparison procedure.

The difference in the mean values among the different levels of Cd conc is greater than would be expected by chance after allowing for effects of differences in Exp. There is a statistically significant difference (P = 0.009). To isolate which group(s) differ from the others use a multiple comparison procedure.

Power of performed test with alpha = 0.0500: for Exp : 0.891
Power of performed test with alpha = 0.0500: for Cd conc : 0.741

Least square means for Exp :

**Group**	**Mean**	**SEM**
272.000	14.224	2.107
273.000	15.454	1.966
278.000	13.135	2.026
280.000	4.467	2.113

Least square means for Cd conc :

**Group**	**Mean**	**SEM**
0.5	7.451	3.108
20	8.492	3.108
50	6.447	3.108
140	11.382	3.108
270	20.532	3.108
550	12.103	3.108
1270	10.716	3.108
3100	9.622	3.108
27500	23.894	3.992
10	7.559	3.640

All Pairwise Multiple Comparison Procedures (Holm-Sidak method):
Overall significance level = 0.05

Comparisons for factor: **Exp**

**Comparison**	**Diff of Means**	**t**	**P**	**P<0.050**
273.000 vs. 280.000	10.987	3.807	0.002	Yes
272.000 vs. 280.000	9.757	3.270	0.009	Yes
278.000 vs. 280.000	8.668	2.961	0.017	Yes
273.000 vs. 278.000	2.319	0.821	0.799	No
273.000 vs. 272.000	1.230	0.427	0.892	No
272.000 vs. 278.000	1.089	0.372	0.711	No

Comparisons for factor: **Cd conc**

**Comparison**	**Diff of Means**	**t**	**P**	**P<0.050**
27500 vs. 50	17.447	3.448	0.045	Yes
27500 vs. 0.5	16.443	3.250	0.079	No
270 vs. 50	14.085	3.204	0.088	No
27500 vs. 20	15.402	3.044	0.134	No
27500 vs. 10	16.335	3.023	0.139	No
270 vs. 0.5	13.082	2.976	0.154	No
27500 vs. 3100	14.272	2.821	0.222	No
270 vs. 20	12.040	2.739	0.264	No
270 vs. 10	12.973	2.710	0.276	No
27500 vs. 1270	13.177	2.604	0.341	No
270 vs. 3100	10.910	2.482	0.427	No
27500 vs. 140	12.512	2.473	0.425	No
27500 vs. 550	11.791	2.330	0.537	No
270 vs. 1270	9.816	2.233	0.612	No
270 vs. 140	9.151	2.082	0.731	No
270 vs. 550	8.430	1.918	0.843	No
550 vs. 50	5.655	1.287	0.999	No
140 vs. 50	4.935	1.123	1.000	No
550 vs. 0.000	4.652	1.058	1.000	No
1270 vs. 50	4.269	0.971	1.000	No

550 vs. 10	4.543	0.949	1.000	No
140 vs. 0.5	3.931	0.894	1.000	No
550 vs. 20	3.611	0.821	1.000	No
140 vs. 10	3.823	0.799	1.000	No
1270 vs. 0.5	3.266	0.743	1.000	No
3100 vs. 50	3.175	0.722	1.000	No
27500 vs. 270	3.361	0.664	1.000	No
1270 vs. 10	3.157	0.660	1.000	No
140 vs. 20	2.890	0.657	1.000	No
550 vs. 3100	2.481	0.564	1.000	No
1270 vs. 20	2.225	0.506	1.000	No
3100 vs. 0.5	2.171	0.494	1.000	No
20 vs. 50	2.045	0.465	1.000	No
3100 vs. 10	2.063	0.431	1.000	No
140 vs. 3100	1.760	0.400	1.000	No
550 vs. 1270	1.386	0.315	1.000	No
3100 vs. 20	1.130	0.257	1.000	No
1270 vs. 3100	1.094	0.249	1.000	No
20 vs. 0.5	1.041	0.237	1.000	No
10 vs. 50	1.112	0.232	1.000	No
0.000 vs. 50	1.004	0.228	1.000	No
20 vs. 10	0.933	0.195	0.999	No
550 vs. 140	0.721	0.164	0.998	No
140 vs. 1270	0.665	0.151	0.986	No
10 vs. 0.5	0.108	0.0227	0.982	No

Elements in the plant tissues - Copper - Stems

Two Way Analysis of Variance
General Linear Model (No Interactions)
Dependent Variable: Cu in stems
Normality Test (Shapiro-Wilk) Failed (P < 0.050)
Equal Variance Test: Failed (P < 0.050)

**Source of Variation**	**DF**	**SS**	**MS**	**F**	**P**
Exp	3	834.931	278.310	39.270	<0.001
Cd conc	9	1603.993	178.221	25.148	<0.001
Residual	127	900.053	7.087		
Total	139	3685.656	26.516		

The difference in the mean values among the different levels of Exp is greater than would be expected by chance after allowing for effects of differences in Cd conc. There is a statistically significant difference (P = <0.001). To isolate which group(s) differ from the others use a multiple comparison procedure.

The difference in the mean values among the different levels of Cd conc is greater than would be expected by chance after allowing for effects of differences in Exp. There is a statistically significant difference (P = <0.001). To isolate which group(s) differ from the others use a multiple comparison procedure.

Power of performed test with alpha = 0.0500: for Exp : 1.000
Power of performed test with alpha = 0.0500: for Cd conc : 1.000

Least square means for Exp :

**Group**	**Mean**	**SEM**
272.000	11.082	0.459
273.000	5.302	0.421
278.000	4.562	0.526
280.000	6.458	0.433

Least square means for Cd conc :

**Group**	**Mean**	**SEM**

0.5	2.166	0.666
20	2.521	0.666
50	3.154	0.666
140	10.218	0.714
270	11.856	0.782
550	9.716	0.714
1270	7.432	0.712
3100	8.759	0.775
27500	8.914	0.666
10	3.773	0.853

All Pairwise Multiple Comparison Procedures (Holm-Sidak method):
Overall significance level = 0.05

Comparisons for factor: **Exp**

**Comparison**	**Diff of Means**	**t**	**P**	**P<0.050**
272.000 vs. 278.000	6.520	9.343	<0.001	Yes
272.000 vs. 273.000	5.780	9.280	<0.001	Yes
272.000 vs. 280.000	4.624	7.330	<0.001	Yes
280.000 vs. 278.000	1.896	2.785	0.018	Yes
280.000 vs. 273.000	1.156	1.915	0.112	No
273.000 vs. 278.000	0.740	1.099	0.274	No

Comparisons for factor: **Cd conc**

**Comparison**	**Diff of Means**	**t**	**P**	**P<0.050**
270 vs. 0.5	9.690	9.435	<0.001	Yes
270 vs. 20	9.336	9.090	<0.001	Yes
270 vs. 50	8.702	8.472	<0.001	Yes
140 vs. 0.5	8.051	8.247	<0.001	Yes
140 vs. 20	7.697	7.884	<0.001	Yes
550 vs. 0.5	7.549	7.733	<0.001	Yes
550 vs. 20	7.195	7.370	<0.001	Yes
140 vs. 50	7.063	7.235	<0.001	Yes
27500 vs. 0.5	6.747	7.169	<0.001	Yes
270 vs. 10	8.083	6.985	<0.001	Yes
27500 vs. 20	6.393	6.792	<0.001	Yes
550 vs. 50	6.561	6.721	<0.001	Yes
3100 vs. 0.5	6.593	6.453	<0.001	Yes
27500 vs. 50	5.759	6.119	<0.001	Yes
3100 vs. 20	6.239	6.106	<0.001	Yes
140 vs. 10	6.444	5.794	<0.001	Yes
3100 vs. 50	5.605	5.486	<0.001	Yes
1270 vs. 0.5	5.265	5.401	<0.001	Yes
550 vs. 10	5.942	5.343	<0.001	Yes
1270 vs. 20	4.911	5.038	<0.001	Yes
27500 vs. 10	5.140	4.752	<0.001	Yes
1270 vs. 50	4.277	4.388	<0.001	Yes
3100 vs. 10	4.986	4.327	<0.001	Yes
270 vs. 1270	4.425	4.182	0.001	Yes
1270 vs. 10	3.658	3.293	0.027	Yes
270 vs. 27500	2.942	2.865	0.093	No
270 vs. 3100	3.097	2.812	0.103	No
140 vs. 1270	2.786	2.762	0.112	No
550 vs. 1270	2.284	2.265	0.352	No
270 vs. 550	2.140	2.021	0.525	No
270 vs. 140	1.639	1.547	0.864	No
27500 vs. 1270	1.482	1.520	0.860	No
10 vs. 0.5	1.607	1.486	0.859	No
140 vs. 3100	1.458	1.383	0.891	No
140 vs. 27500	1.304	1.336	0.893	No
3100 vs. 1270	1.328	1.261	0.905	No

10 vs. 20	1.253	1.158	0.924	No
50 vs. 0.000	0.988	1.050	0.940	No
550 vs. 3100	0.957	0.908	0.959	No
550 vs. 27500	0.802	0.822	0.959	No
50 vs. 20	0.634	0.673	0.969	No
10 vs. 50	0.619	0.572	0.965	No
140 vs. 550	0.502	0.497	0.945	No
20 vs. 0.5	0.354	0.376	0.914	No
27500 vs. 3100	0.155	0.151	0.880	No

Elements in the plant tissues - Copper - Roots

Two Way Analysis of Variance
General Linear Model (No Interactions)
Dependent Variable: Cu in roots
Normality Test (Shapiro-Wilk) Failed (P < 0.050)
Equal Variance Test: Passed (P = 1.000)

Source of Variation	DF	SS	MS	F	P
Exp	3	13161.626	4387.209	3.669	0.026
Cd conc	9	41403.736	4600.415	3.848	0.004
Residual	25	29891.893	1195.676		
Total	37	86588.634	2340.233		

The difference in the mean values among the different levels of Exp is greater than would be expected by chance after allowing for effects of differences in Cd conc. There is a statistically significant difference (P = 0.026). To isolate which group(s) differ from the others use a multiple comparison procedure.

The difference in the mean values among the different levels of Cd conc is greater than would be expected by chance after allowing for effects of differences in Exp. There is a statistically significant difference (P = 0.004). To isolate which group(s) differ from the others use a multiple comparison procedure.

Power of performed test with alpha = 0.0500: for Exp : 0.575
Power of performed test with alpha = 0.0500: for Cd conc : 0.881

Least square means for Exp :

Group	Mean	SEM
272.000	54.633	11.811
273.000	54.353	10.935
278.000	36.951	10.935
280.000	89.313	11.811

Least square means for Cd conc :

Group	Mean	SEM
0.5	20.023	17.289
20	12.382	17.289
50	14.976	17.289
140	83.712	17.289
270	89.010	17.289
550	80.103	17.289
1270	80.757	17.289
3100	91.511	17.289
27500	87.406	17.289
10	28.243	25.121

All Pairwise Multiple Comparison Procedures (Holm-Sidak method):
Overall significance level = 0.05

Comparisons for factor: **Exp**

Comparison	Diff of Means	t	P	P<0.050

280.000 vs. 278.000	52.362	3.253	0.019	Yes
280.000 vs. 273.000	34.960	2.172	0.183	No
280.000 vs. 272.000	34.680	2.076	0.180	No
273.000 vs. 278.000	17.402	1.125	0.613	No
272.000 vs. 278.000	17.682	1.099	0.485	No
272.000 vs. 273.000	0.280	0.0174	0.986	No

Comparisons for factor: **Cd conc**

Comparison	Diff of Means	t	P	P<0.050
3100 vs. 20	79.129	3.236	0.142	No
270 vs. 20	76.628	3.134	0.175	No
3100 vs. 50	76.535	3.130	0.173	No
27500 vs. 20	75.024	3.068	0.194	No
270 vs. 50	74.034	3.028	0.207	No
27500 vs. 50	72.430	2.962	0.233	No
3100 vs. 0.5	71.488	2.924	0.247	No
140 vs. 20	71.331	2.917	0.245	No
270 vs. 0.5	68.987	2.821	0.290	No
140 vs. 50	68.737	2.811	0.290	No
1270 vs. 20	68.376	2.796	0.291	No
550 vs. 20	67.721	2.770	0.300	No
27500 vs. 0.5	67.383	2.756	0.300	No
1270 vs. 50	65.782	2.690	0.332	No
550 vs. 50	65.127	2.664	0.340	No
140 vs. 0.5	63.689	2.605	0.369	No
1270 vs. 0.5	60.734	2.484	0.444	No
550 vs. 0.5	60.080	2.457	0.453	No
3100 vs. 10	63.268	2.075	0.738	No
270 vs. 10	60.767	1.993	0.785	No
27500 vs. 10	59.162	1.940	0.807	No
140 vs. 10	55.469	1.819	0.868	No
1270 vs. 10	52.514	1.722	0.905	No
550 vs. 10	51.860	1.701	0.905	No
10 vs. 20	15.861	0.520	1.000	No
3100 vs. 550	11.408	0.467	1.000	No
3100 vs. 1270	10.753	0.440	1.000	No
10 vs. 50	13.268	0.435	1.000	No
270 vs. 550	8.907	0.364	1.000	No
270 vs. 1270	8.252	0.338	1.000	No
3100 vs. 140	7.798	0.319	1.000	No
0.000 vs. 20	7.641	0.313	1.000	No
27500 vs. 550	7.303	0.299	1.000	No
27500 vs. 1270	6.648	0.272	1.000	No
10 vs. 0.000	8.220	0.270	1.000	No
270 vs. 140	5.297	0.217	1.000	No
0.000 vs. 50	5.047	0.206	1.000	No
3100 vs. 27500	4.105	0.168	1.000	No
27500 vs. 140	3.693	0.151	1.000	No
140 vs. 550	3.610	0.148	1.000	No
140 vs. 1270	2.955	0.121	1.000	No
50 vs. 20	2.594	0.106	1.000	No
3100 vs. 270	2.501	0.102	0.999	No
270 vs. 27500	1.604	0.0656	0.997	No
1270 vs. 550	0.655	0.0268	0.979	No

Elements in the plant tissues - Copper - Harvested Leaves

Two Way Analysis of Variance
General Linear Model (No Interactions)
Dependent Variable: Cu harvest

Normality Test (Shapiro-Wilk) Failed (P < 0.050)
Equal Variance Test: Passed (P = 0.122)

**Source of Variation**	**DF**	**SS**	**MS**	**F**	**P**
Exp	3	267.917	89.306	1.999	0.118
Cd conc	9	1738.485	193.165	4.325	<0.001
Residual	115	5136.521	44.665		
Total	127	7130.120	56.143		

The difference in the mean values among the different levels of Exp is not great enough to exclude the possibility that the difference is just due to random sampling variability after allowing for the effects of differences in Cd conc. There is not a statistically significant difference (P = 0.118).

The difference in the mean values among the different levels of Cd conc is greater than would be expected by chance after allowing for effects of differences in Exp. There is a statistically significant difference (P = <0.001). To isolate which group(s) differ from the others use a multiple comparison procedure.

Power of performed test with alpha = 0.0500: for Exp : 0.258
Power of performed test with alpha = 0.0500: for Cd conc : 0.982

Least square means for Exp :

**Group**	**Mean**	**SEM**
272.000	9.969	1.223
273.000	9.126	1.086
278.000	9.031	1.414
280.000	12.548	1.136

Least square means for Cd conc :

**Group**	**Mean**	**SEM**
0.5	4.942	1.671
20	7.763	1.671
50	9.335	2.055
140	10.261	1.855
270	8.734	2.018
550	12.939	1.935
1270	10.870	1.861
3100	11.257	2.158
27500	18.253	1.727
10	7.333	2.046

All Pairwise Multiple Comparison Procedures (Holm-Sidak method):
Overall significance level = 0.05

Comparisons for factor: **Exp**

**Comparison**	**Diff of Means**	**t**	**P**	**P<0.050**
280.000 vs. 273.000	3.422	2.177	0.175	No
280.000 vs. 278.000	3.517	1.939	0.246	No
280.000 vs. 272.000	2.579	1.545	0.414	No
272.000 vs. 273.000	0.843	0.515	0.939	No
272.000 vs. 278.000	0.938	0.502	0.853	No
273.000 vs. 278.000	0.0955	0.0535	0.957	No

Comparisons for factor: **Cd conc**

**Comparison**	**Diff of Means**	**t**	**P**	**P<0.050**
27500 vs. 0.5	13.311	5.539	<0.001	Yes
27500 vs. 20	10.490	4.365	0.001	Yes
27500 vs. 10	10.920	4.078	0.004	Yes
27500 vs. 270	9.519	3.584	0.021	Yes
27500 vs. 50	8.918	3.322	0.048	Yes
27500 vs. 140	7.992	3.153	0.079	No

550 vs. 0.5	7.997	3.128	0.083	No
27500 vs. 1270	7.383	2.908	0.153	No
27500 vs. 3100	6.996	2.531	0.377	No
1270 vs. 0.5	5.928	2.370	0.507	No
3100 vs. 0.5	6.315	2.314	0.548	No
140 vs. 0.5	5.319	2.131	0.705	No
27500 vs. 550	5.314	2.049	0.764	No
550 vs. 20	5.176	2.025	0.772	No
550 vs. 10	5.606	1.991	0.789	No
50 vs. 0.5	4.393	1.659	0.957	No
550 vs. 270	4.205	1.504	0.985	No
270 vs. 0.5	3.792	1.447	0.990	No
3100 vs. 10	3.924	1.319	0.997	No
3100 vs. 20	3.494	1.280	0.997	No
1270 vs. 10	3.537	1.279	0.997	No
550 vs. 50	3.604	1.277	0.996	No
1270 vs. 20	3.107	1.242	0.996	No
20 vs. 0.5	2.821	1.194	0.997	No
140 vs. 10	2.928	1.060	0.999	No
140 vs. 20	2.498	1.001	1.000	No
550 vs. 140	2.678	0.999	0.999	No
10 vs. 0.5	2.391	0.905	1.000	No
3100 vs. 270	2.523	0.854	1.000	No
1270 vs. 270	2.136	0.778	1.000	No
550 vs. 1270	2.069	0.771	1.000	No
50 vs. 10	2.002	0.690	1.000	No
3100 vs. 50	1.922	0.645	1.000	No
50 vs. 20	1.572	0.594	1.000	No
550 vs. 3100	1.682	0.580	1.000	No
140 vs. 270	1.527	0.557	1.000	No
1270 vs. 50	1.535	0.554	1.000	No
270 vs. 10	1.401	0.487	1.000	No
270 vs. 20	0.971	0.371	1.000	No
3100 vs. 140	0.996	0.350	1.000	No
140 vs. 50	0.926	0.335	0.999	No
1270 vs. 140	0.609	0.232	0.999	No
50 vs. 270	0.601	0.209	0.996	No
20 vs. 10	0.430	0.163	0.983	No
3100 vs. 1270	0.387	0.136	0.892	No

Elements in the plant tissues - Iron - Harvested Leaves

Two Way Analysis of Variance
General Linear Model (No Interactions)
Dependent Variable: Fe harvest
Normality Test (Shapiro-Wilk) Failed (P < 0.050)
Equal Variance Test: Failed (P < 0.050)

**Source of Variation**	**DF**	**SS**	**MS**	**F**	**P**
Exp	3	1150.792	383.597	2.153	0.097
Cd conc	9	7248.477	805.386	4.521	<0.001
Residual	116	20666.452	178.159		
Total	128	29254.652	228.552		

The difference in the mean values among the different levels of Exp is not great enough to exclude the possibility that the difference is just due to random sampling variability after allowing for the effects of differences in Cd conc. There is not a statistically significant difference (P = 0.097).

The difference in the mean values among the different levels of Cd conc is greater than would be expected by chance after allowing for effects of differences in Exp. There is a statistically significant difference (P = <0.001). To isolate which group(s) differ from the others use a multiple comparison procedure.

Power of performed test with alpha = 0.0500: for Exp : 0.294
Power of performed test with alpha = 0.0500: for Cd conc : 0.988

Least square means for Exp :

**Group**	**Mean**	**SEM**
272.000	15.200	2.443
273.000	9.571	2.169
278.000	12.288	2.774
280.000	17.089	2.269

Least square means for Cd conc :

**Group**	**Mean**	**SEM**
0.5	18.379	3.337
20	27.722	3.337
50	20.355	4.102
140	7.743	3.572
270	7.246	4.030
550	8.713	3.864
1270	7.010	3.716
3100	6.678	4.308
27500	9.650	3.449
10	21.873	4.087

All Pairwise Multiple Comparison Procedures (Holm-Sidak method):
Overall significance level = 0.05

Comparisons for factor: **Exp**

**Comparison**	**Diff of Means**	**t**	**P**	**P<0.050**
280.000 vs. 273.000	7.518	2.395	0.105	No
272.000 vs. 273.000	5.629	1.723	0.367	No
280.000 vs. 278.000	4.801	1.340	0.554	No
272.000 vs. 278.000	2.912	0.788	0.817	No
278.000 vs. 273.000	2.717	0.771	0.689	No
280.000 vs. 272.000	1.889	0.567	0.572	No

Comparisons for factor: **Cd conc**

**Comparison**	**Diff of Means**	**t**	**P**	**P<0.050**
20 vs. 1270	20.712	4.147	0.003	Yes
20 vs. 140	19.979	4.088	0.004	Yes
20 vs. 270	20.476	3.914	0.007	Yes
20 vs. 3100	21.044	3.862	0.008	Yes
20 vs. 27500	18.072	3.766	0.011	Yes
20 vs. 550	19.009	3.723	0.012	Yes
10 vs. 1270	14.863	2.691	0.274	No
10 vs. 140	14.130	2.603	0.329	No
10 vs. 3100	15.195	2.559	0.355	No
10 vs. 270	14.627	2.548	0.355	No
50 vs. 1270	13.345	2.411	0.460	No
10 vs. 550	13.160	2.340	0.514	No
50 vs. 140	12.612	2.319	0.522	No
50 vs. 3100	13.677	2.299	0.529	No
10 vs. 27500	12.223	2.286	0.531	No
50 vs. 270	13.110	2.280	0.524	No
0.5 vs. 1270	11.369	2.276	0.515	No
0.5 vs. 140	10.636	2.176	0.593	No

0.5 vs. 3100	11.701	2.147	0.605	No
0.5 vs. 270	11.133	2.128	0.609	No
50 vs. 550	11.642	2.066	0.649	No
50 vs. 27500	10.705	1.998	0.694	No
20 vs. 0.5	9.343	1.980	0.693	No
0.5 vs. 550	9.666	1.893	0.749	No
0.5 vs. 27500	8.729	1.819	0.790	No
20 vs. 50	7.367	1.393	0.974	No
20 vs. 10	5.849	1.109	0.997	No
10 vs. 0.5	3.494	0.662	1.000	No
27500 vs. 3100	2.972	0.539	1.000	No
27500 vs. 1270	2.640	0.521	1.000	No
27500 vs. 270	2.404	0.453	1.000	No
27500 vs. 140	1.907	0.384	1.000	No
50 vs. 0.5	1.976	0.374	1.000	No
550 vs. 3100	2.035	0.352	1.000	No
550 vs. 1270	1.703	0.318	1.000	No
550 vs. 270	1.467	0.263	1.000	No
10 vs. 50	1.518	0.262	1.000	No
140 vs. 3100	1.065	0.190	1.000	No
550 vs. 140	0.970	0.184	1.000	No
27500 vs. 550	0.937	0.181	1.000	No
140 vs. 1270	0.733	0.142	1.000	No
270 vs. 3100	0.568	0.0962	1.000	No
140 vs. 270	0.497	0.0924	1.000	No
1270 vs. 3100	0.332	0.0584	0.998	No
270 vs. 1270	0.235	0.0430	0.966	No

Elements in the plant tissues - Iron -Seeds

Two Way Analysis of Variance
General Linear Model (No Interactions)
Dependent Variable: Fe seeds
Normality Test (Shapiro-Wilk) Passed (P = 0.325)
Equal Variance Test: Failed (P < 0.050)

**Source of Variation**	**DF**	**SS**	**MS**	**F**	**P**
Exp	3	1007.303	335.768	2.453	0.074
Cd conc	3	537.049	179.016	1.308	0.282
Residual	52	7118.373	136.892		
Total	58	8562.445	147.628		

The difference in the mean values among the different levels of Exp is not great enough to exclude the possibility that the difference is just due to random sampling variability after allowing for the effects of differences in Cd conc. There is not a statistically significant difference (P = 0.074).

The difference in the mean values among the different levels of Cd conc is not great enough to exclude the possibility that the difference is just due to random sampling variability after allowing for the effects of differences in Exp. There is not a statistically significant difference (P = 0.282).

Power of performed test with alpha = 0.0500: for Exp : 0.353
Power of performed test with alpha = 0.0500: for Cd conc : 0.103

Least square means for Exp :

**Group**	**Mean**	**SEM**
272.000	58.275	3.525
273.000	62.383	2.925
278.000	70.145	3.028
280.000	62.648	2.925

Least square means for Cd conc :

Group	Mean	SEM
0.5	61.809	2.925
20	66.408	2.925
50	66.429	2.925
10	58.804	3.667

Elements in the plant tissues - Iron -Stems

Two Way Analysis of Variance
General Linear Model (No Interactions)
Dependent Variable: Fe stems
Normality Test (Shapiro-Wilk) Failed (P < 0.050)
Equal Variance Test: Passed (P = 0.479)

Source of Variation	DF	SS	MS	F	P
Exp	3	15171.493	5057.164	4.069	0.008
Cd conc	9	112796.605	12532.956	10.084	<0.001
Residual	127	157845.769	1242.880		
Total	139	286999.404	2064.744		

The difference in the mean values among the different levels of Exp is greater than would be expected by chance after allowing for effects of differences in Cd conc. There is a statistically significant difference (P = 0.008). To isolate which group(s) differ from the others use a multiple comparison procedure.

The difference in the mean values among the different levels of Cd conc is greater than would be expected by chance after allowing for effects of differences in Exp. There is a statistically significant difference (P = <0.001). To isolate which group(s) differ from the others use a multiple comparison procedure.

Power of performed test with alpha = 0.0500: for Exp : 0.708
Power of performed test with alpha = 0.0500: for Cd conc : 1.000

Least square means for Exp :

Group	Mean	SEM
272.000	69.763	6.079
273.000	46.431	5.574
278.000	51.249	6.961
280.000	42.778	5.730

Least square means for Cd conc :

Group	Mean	SEM
0.5	92.637	8.814
20	73.147	8.814
50	87.182	8.814
140	14.764	9.459
270	16.903	10.359
550	19.160	9.459
1270	34.233	9.432
3100	46.029	10.266
27500	70.615	8.814
10	70.881	11.292

All Pairwise Multiple Comparison Procedures (Holm-Sidak method):
Overall significance level = 0.05

Comparisons for factor: **Exp**

Comparison	Diff of Means	t	P	P<0.050
272.000 vs. 280.000	26.984	3.230	0.009	Yes

272.000 vs. 273.000	23.332	2.829	0.027	Yes
272.000 vs. 278.000	18.513	2.003	0.176	No
278.000 vs. 280.000	8.471	0.940	0.724	No
278.000 vs. 273.000	4.818	0.540	0.832	No
273.000 vs. 280.000	3.653	0.457	0.649	No

Comparisons for factor: **Cd conc**

**Comparison**	**Diff of Means**	**t**	**P**	**P<0.050**
0.5 vs. 140	77.874	6.023	<0.001	Yes
0.5 vs. 550	73.477	5.683	<0.001	Yes
50 vs. 140	72.418	5.601	<0.001	Yes
0.5 vs. 270	75.734	5.568	<0.001	Yes
50 vs. 550	68.022	5.261	<0.001	Yes
50 vs. 270	70.278	5.167	<0.001	Yes
0.5 vs. 1270	58.404	4.524	<0.001	Yes
20 vs. 140	58.384	4.516	<0.001	Yes
27500 vs. 140	55.851	4.320	0.001	Yes
20 vs. 550	53.987	4.176	0.002	Yes
20 vs. 270	56.244	4.135	0.002	Yes
50 vs. 1270	52.948	4.102	0.002	Yes
27500 vs. 550	51.455	3.980	0.004	Yes
27500 vs. 270	53.712	3.949	0.004	Yes
10 vs. 140	56.118	3.810	0.007	Yes
10 vs. 270	53.978	3.523	0.018	Yes
10 vs. 550	51.721	3.511	0.018	Yes
0.5 vs. 3100	46.608	3.445	0.021	Yes
50 vs. 3100	41.152	3.041	0.074	No
20 vs. 1270	38.914	3.014	0.078	No
27500 vs. 1270	36.382	2.818	0.131	No
10 vs. 1270	36.648	2.491	0.288	No
3100 vs. 140	31.266	2.240	0.465	No
20 vs. 3100	27.118	2.004	0.655	No
3100 vs. 270	29.126	1.997	0.644	No
3100 vs. 550	26.869	1.925	0.687	No
27500 vs. 3100	24.586	1.817	0.756	No
0.5 vs. 27500	22.022	1.767	0.776	No
10 vs. 3100	24.852	1.628	0.851	No
0.5 vs. 20	19.490	1.564	0.872	No
0.5 vs. 10	21.756	1.519	0.879	No
1270 vs. 140	19.470	1.458	0.893	No
50 vs. 27500	16.566	1.329	0.931	No
1270 vs. 270	17.330	1.237	0.948	No
50 vs. 10	16.300	1.138	0.962	No
1270 vs. 550	15.073	1.128	0.952	No
50 vs. 20	14.034	1.126	0.935	No
3100 vs. 1270	11.796	0.846	0.983	No
0.5 vs. 50	5.456	0.438	0.999	No
550 vs. 140	4.396	0.329	1.000	No
20 vs. 27500	2.532	0.203	1.000	No
550 vs. 270	2.257	0.161	1.000	No
20 vs. 10	2.266	0.158	0.998	No
270 vs. 140	2.140	0.153	0.985	No
10 vs. 27500	0.266	0.0186	0.985	No

Elements in the plant tissues - Iron -Roots

Two Way Analysis of Variance
General Linear Model (No Interactions)
Dependent Variable: Fe roots
Normality Test (Shapiro-Wilk) Passed (P = 0.737)

Equal Variance Test: Passed (P = 1.000)

**Source of Variation**	**DF**	**SS**	**MS**	**F**	**P**
Exp	3	59064.508	19688.169	1.872	0.159
Cd conc	9	962902.339	106989.149	10.171	<0.001
Residual	26	273505.031	10519.424		
Total	38	1284887.867	33812.839		

The difference in the mean values among the different levels of Exp is not great enough to exclude the possibility that the difference is just due to random sampling variability after allowing for the effects of differences in Cd conc. There is not a statistically significant difference (P = 0.159).

The difference in the mean values among the different levels of Cd conc is greater than would be expected by chance after allowing for effects of differences in Exp. There is a statistically significant difference (P = <0.001). To isolate which group(s) differ from the others use a multiple comparison procedure.

Power of performed test with alpha = 0.0500: for Exp : 0.208
Power of performed test with alpha = 0.0500: for Cd conc : 1.000

Least square means for Exp :

**Group**	**Mean**	**SEM**
272.000	147.992	34.753
273.000	208.753	32.434
278.000	156.748	32.434
280.000	243.984	32.434

Least square means for Cd conc :

**Group**	**Mean**	**SEM**
0.5	28.628	51.282
20	47.539	51.282
50	24.083	51.282
140	179.024	51.282
270	167.878	51.282
550	316.077	51.282
1270	246.706	51.282
3100	523.084	51.282
27500	326.452	51.282
10	34.224	60.032

All Pairwise Multiple Comparison Procedures (Holm-Sidak method):
Overall significance level = 0.05

Comparisons for factor: **Exp**

**Comparison**	**Diff of Means**	**t**	**P**	**P<0.050**
280.000 vs. 272.000	95.992	2.019	0.283	No
280.000 vs. 278.000	87.236	1.902	0.298	No
273.000 vs. 272.000	60.761	1.278	0.615	No
273.000 vs. 278.000	52.005	1.134	0.607	No
280.000 vs. 273.000	35.231	0.768	0.697	No
278.000 vs. 272.000	8.755	0.184	0.855	No

Comparisons for factor: **Cd conc**

**Comparison**	**Diff of Means**	**t**	**P**	**P<0.050**
3100 vs. 50	499.001	6.881	<0.001	Yes
3100 vs. 0.5	494.456	6.818	<0.001	Yes
3100 vs. 20	475.545	6.557	<0.001	Yes
3100 vs. 10	488.860	6.192	<0.001	Yes
3100 vs. 270	355.206	4.898	0.002	Yes
3100 vs. 140	344.060	4.744	0.003	Yes
27500 vs. 50	302.370	4.169	0.012	Yes
27500 vs. 0.5	297.824	4.107	0.013	Yes

550 vs. 50	291.994	4.026	0.016	Yes
550 vs. 0.5	287.449	3.964	0.018	Yes
27500 vs. 20	278.913	3.846	0.024	Yes
3100 vs. 1270	276.378	3.811	0.026	Yes
550 vs. 20	268.538	3.703	0.033	Yes
27500 vs. 10	292.228	3.701	0.032	Yes
550 vs. 10	281.853	3.570	0.043	Yes
1270 vs. 50	222.623	3.070	0.139	No
1270 vs. 0.5	218.078	3.007	0.155	No
3100 vs. 550	207.007	2.854	0.209	No
1270 vs. 20	199.167	2.746	0.254	No
3100 vs. 27500	196.632	2.711	0.264	No
1270 vs. 10	212.482	2.691	0.266	No
27500 vs. 270	158.574	2.187	0.605	No
140 vs. 50	154.942	2.136	0.629	No
140 vs. 0.5	150.396	2.074	0.662	No
550 vs. 270	148.199	2.043	0.669	No
27500 vs. 140	147.428	2.033	0.659	No
270 vs. 50	143.795	1.983	0.679	No
270 vs. 0.5	139.250	1.920	0.707	No
550 vs. 140	137.053	1.890	0.709	No
140 vs. 10	144.800	1.834	0.728	No
140 vs. 20	131.485	1.813	0.720	No
270 vs. 10	133.654	1.693	0.780	No
270 vs. 20	120.339	1.659	0.777	No
27500 vs. 1270	79.746	1.100	0.981	No
1270 vs. 270	78.828	1.087	0.976	No
550 vs. 1270	69.371	0.957	0.986	No
1270 vs. 140	67.682	0.933	0.982	No
20 vs. 50	23.456	0.323	1.000	No
20 vs. 0.5	18.911	0.261	1.000	No
20 vs. 10	13.315	0.169	1.000	No
140 vs. 270	11.146	0.154	1.000	No
27500 vs. 550	10.375	0.143	1.000	No
10 vs. 50	10.141	0.128	0.999	No
10 vs. 0.5	5.596	0.0709	0.997	No
0.5 vs. 50	4.545	0.0627	0.951	No

Elements in the plant tissues - Iron -w10 leaves

Two Way Analysis of Variance
General Linear Model (No Interactions)
Dependent Variable: Fe w10
Normality Test (Shapiro-Wilk) Failed (P < 0.050)
Equal Variance Test: Failed (P < 0.050)

Source of Variation	DF	SS	MS	F	P
Exp	3	8472.070	2824.023	1.105	0.358
Cd conc	8	28740.709	3592.589	1.405	0.222
Residual	43	109934.368	2556.613		
Total	54	147780.745	2736.680		

The difference in the mean values among the different levels of Exp is not great enough to exclude the possibility that the difference is just due to random sampling variability after allowing for the effects of differences in Cd conc. There is not a statistically significant difference (P = 0.358).

The difference in the mean values among the different levels of Cd conc is not great enough to exclude the possibility that the difference is just due to random sampling variability after allowing for the effects of differences in Exp. There is not a statistically significant difference (P = 0.222).

Power of performed test with alpha = 0.0500: for Exp : 0.0664
Power of performed test with alpha = 0.0500: for Cd conc : 0.167

Least square means for Exp :

Group	Mean	SEM
272.000	11.750	19.745
273.000	11.946	13.290
278.000	10.824	12.415
280.000	39.615	13.259

Least square means for Cd conc :

Group	Mean	SEM
0.5	13.350	19.240
20	18.703	19.240
50	11.805	19.240
140	79.530	19.240
270	18.695	19.240
550	7.600	20.785
1270	7.762	20.785
10	12.483	21.283
3100	-3.125	36.515

Elements in the plant tissues - Iron -w5 leaves

Two Way Analysis of Variance
General Linear Model (No Interactions)
Dependent Variable: Fe w5
Normality Test (Shapiro-Wilk) Passed (P = 0.221)
Equal Variance Test: Failed (P < 0.050)

Source of Variation	DF	SS	MS	F	P
Exp	3	1416.610	472.203	2.875	0.043
Cd conc	9	62304.205	6922.689	42.143	<0.001
Residual	62	10184.628	164.268		
Total	74	74234.416	1003.168		

The difference in the mean values among the different levels of Exp is greater than would be expected by chance after allowing for effects of differences in Cd conc. There is a statistically significant difference (P = 0.043). To isolate which group(s) differ from the others use a multiple comparison procedure.

The difference in the mean values among the different levels of Cd conc is greater than would be expected by chance after allowing for effects of differences in Exp. There is a statistically significant difference (P = <0.001). To isolate which group(s) differ from the others use a multiple comparison procedure.

Power of performed test with alpha = 0.0500: for Exp : 0.454
Power of performed test with alpha = 0.0500: for Cd conc : 1.000

Least square means for Exp :

Group	Mean	SEM
272.000	40.380	3.071
273.000	44.550	2.866
278.000	47.042	2.954
280.000	35.469	3.081

Least square means for Cd conc :

Group	Mean	SEM
0.5	84.169	4.531
20	77.755	4.531
50	66.442	4.531

140	20.995	4.531
270	16.009	4.531
550	15.184	4.531
1270	12.209	4.531
3100	16.651	4.531
27500	35.133	5.820
10	74.055	5.306

All Pairwise Multiple Comparison Procedures (Holm-Sidak method):
Overall significance level = 0.05

Comparisons for factor: **Exp**

**Comparison**	**Diff of Means**	**t**	**P**	**P<0.050**
278.000 vs. 280.000	11.573	2.712	0.051	No
273.000 vs. 280.000	9.082	2.159	0.162	No
278.000 vs. 272.000	6.661	1.563	0.409	No
272.000 vs. 280.000	4.912	1.129	0.600	No
273.000 vs. 272.000	4.170	0.993	0.544	No
278.000 vs. 273.000	2.491	0.605	0.547	No

Comparisons for factor: **Cd conc**

**Comparison**	**Diff of Means**	**t**	**P**	**P<0.050**
0.5 vs. 1270	71.960	11.229	<0.001	Yes
0.5 vs. 550	68.985	10.765	<0.001	Yes
0.5 vs. 270	68.160	10.636	<0.001	Yes
0.5 vs. 3100	67.518	10.536	<0.001	Yes
20 vs. 1270	65.546	10.228	<0.001	Yes
0.5 vs. 140	63.174	9.858	<0.001	Yes
20 vs. 550	62.571	9.764	<0.001	Yes
20 vs. 270	61.747	9.635	<0.001	Yes
20 vs. 3100	61.104	9.535	<0.001	Yes
10 vs. 1270	61.845	8.863	<0.001	Yes
20 vs. 140	56.761	8.857	<0.001	Yes
50 vs. 1270	54.233	8.463	<0.001	Yes
10 vs. 550	58.870	8.437	<0.001	Yes
10 vs. 270	58.046	8.319	<0.001	Yes
10 vs. 3100	57.403	8.227	<0.001	Yes
50 vs. 550	51.258	7.999	<0.001	Yes
50 vs. 270	50.433	7.870	<0.001	Yes
50 vs. 3100	49.791	7.770	<0.001	Yes
10 vs. 140	53.060	7.604	<0.001	Yes
50 vs. 140	45.447	7.092	<0.001	Yes
0.5 vs. 27500	49.036	6.648	<0.001	Yes
20 vs. 27500	42.623	5.778	<0.001	Yes
10 vs. 27500	38.922	4.942	<0.001	Yes
50 vs. 27500	31.309	4.245	0.002	Yes
27500 vs. 1270	22.924	3.108	0.058	No
0.5 vs. 50	17.727	2.766	0.139	No
27500 vs. 550	19.949	2.704	0.155	No
27500 vs. 270	19.124	2.593	0.193	No
27500 vs. 3100	18.482	2.506	0.225	No
27500 vs. 140	14.138	1.917	0.628	No
20 vs. 50	11.313	1.765	0.725	No
0.5 vs. 10	10.115	1.450	0.901	No
140 vs. 1270	8.786	1.371	0.918	No
10 vs. 50	7.613	1.091	0.980	No
0.5 vs. 20	6.414	1.001	0.986	No
140 vs. 550	5.811	0.907	0.990	No
140 vs. 270	4.986	0.778	0.995	No
3100 vs. 1270	4.442	0.693	0.995	No
140 vs. 3100	4.344	0.678	0.992	No

270 vs. 1270	3.800	0.593	0.992	No
20 vs. 10	3.701	0.530	0.989	No
550 vs. 1270	2.975	0.464	0.984	No
3100 vs. 550	1.467	0.229	0.994	No
270 vs. 550	0.825	0.129	0.990	No
3100 vs. 270	0.642	0.100	0.920	No

Elements in the plant tissues - Nickel - Harvested leaves

Two Way Analysis of Variance
General Linear Model (No Interactions)
Dependent Variable: Ni harvest
Normality Test (Shapiro-Wilk) Failed (P < 0.050)
Equal Variance Test: Failed (P < 0.050)

**Source of Variation**	**DF**	**SS**	**MS**	**F**	**P**
Exp	3	1545.463	515.154	10.239	<0.001
Cd conc	9	4669.984	518.887	10.313	<0.001
Residual	115	5786.151	50.314		
Total	127	11914.446	93.815		

The difference in the mean values among the different levels of Exp is greater than would be expected by chance after allowing for effects of differences in Cd conc. There is a statistically significant difference (P = <0.001). To isolate which group(s) differ from the others use a multiple comparison procedure.

The difference in the mean values among the different levels of Cd conc is greater than would be expected by chance after allowing for effects of differences in Exp. There is a statistically significant difference (P = <0.001). To isolate which group(s) differ from the others use a multiple comparison procedure.

Power of performed test with alpha = 0.0500: for Exp : 0.998
Power of performed test with alpha = 0.0500: for Cd conc : 1.000

Least square means for Exp :

**Group**	**Mean**	**SEM**
272.000	10.167	1.298
273.000	9.162	1.153
278.000	9.376	1.501
280.000	17.344	1.206

Least square means for Cd conc :

**Group**	**Mean**	**SEM**
0.5	2.443	1.773
20	5.567	1.773
50	9.552	2.181
140	19.764	1.969
270	15.317	2.142
550	21.090	2.054
1270	14.126	1.975
3100	12.481	2.290
27500	10.208	1.833
10	4.573	2.172

All Pairwise Multiple Comparison Procedures (Holm-Sidak method):
Overall significance level = 0.05

Comparisons for factor: **Exp**

**Comparison**	**Diff of Means**	**t**	**P**	**P<0.050**
280.000 vs. 273.000	8.182	4.904	<0.001	Yes
280.000 vs. 278.000	7.968	4.138	<0.001	Yes

280.000 vs. 272.000	7.176	4.050	<0.001	Yes
272.000 vs. 273.000	1.006	0.579	0.917	No
272.000 vs. 278.000	0.791	0.399	0.904	No
278.000 vs. 273.000	0.214	0.113	0.910	No

Comparisons for factor: **Cd conc**

Comparison	Diff of Means	t	P	P<0.050
550 vs. 0.5	18.647	6.872	<0.001	Yes
140 vs. 0.5	17.321	6.537	<0.001	Yes
550 vs. 20	15.523	5.721	<0.001	Yes
550 vs. 10	16.517	5.526	<0.001	Yes
140 vs. 20	14.198	5.358	<0.001	Yes
140 vs. 10	15.192	5.182	<0.001	Yes
270 vs. 0.5	12.874	4.630	<0.001	Yes
1270 vs. 0.5	11.683	4.401	<0.001	Yes
550 vs. 27500	10.882	3.953	0.005	Yes
550 vs. 50	11.538	3.852	0.007	Yes
140 vs. 27500	9.556	3.552	0.019	Yes
270 vs. 10	10.744	3.522	0.021	Yes
270 vs. 20	9.750	3.507	0.021	Yes
140 vs. 50	10.212	3.476	0.023	Yes
3100 vs. 0.5	10.038	3.466	0.023	Yes
1270 vs. 10	9.553	3.254	0.044	Yes
1270 vs. 20	8.559	3.225	0.047	Yes
27500 vs. 0.5	7.765	3.045	0.078	No
550 vs. 3100	8.609	2.798	0.151	No
50 vs. 0.5	7.109	2.529	0.284	No
3100 vs. 10	7.909	2.506	0.290	No
550 vs. 1270	6.964	2.444	0.321	No
140 vs. 3100	7.283	2.411	0.333	No
3100 vs. 20	6.915	2.387	0.339	No
140 vs. 1270	5.639	2.022	0.624	No
27500 vs. 10	5.636	1.983	0.640	No
550 vs. 270	5.773	1.946	0.653	No
270 vs. 50	5.765	1.886	0.683	No
27500 vs. 20	4.641	1.820	0.716	No
270 vs. 27500	5.109	1.812	0.700	No
50 vs. 10	4.980	1.618	0.821	No
1270 vs. 50	4.573	1.554	0.840	No
140 vs. 270	4.448	1.529	0.834	No
1270 vs. 27500	3.917	1.454	0.855	No
50 vs. 20	3.985	1.418	0.851	No
20 vs. 0.5	3.124	1.246	0.912	No
3100 vs. 50	2.929	0.926	0.981	No
270 vs. 3100	2.835	0.904	0.974	No
3100 vs. 27500	2.273	0.775	0.983	No
10 vs. 0.5	2.130	0.760	0.972	No
1270 vs. 3100	1.644	0.544	0.988	No
550 vs. 140	1.326	0.466	0.984	No
270 vs. 1270	1.191	0.409	0.968	No
20 vs. 10	0.994	0.355	0.924	No
27500 vs. 50	0.656	0.230	0.818	No

Elements in the plant tissues - Nickel - w10 leaves

Two Way Analysis of Variance
General Linear Model (No Interactions)
Dependent Variable: Ni w10
Normality Test (Shapiro-Wilk) Failed (P < 0.050)
Equal Variance Test: Failed (P < 0.050)

Source of Variation	DF	SS	MS	F	P
Exp	3	6109.638	2036.546	1.105	0.358
Cd conc	8	34543.248	4317.906	2.342	0.035
Residual	43	79272.119	1843.538		
Total	54	119600.421	2214.823		

The difference in the mean values among the different levels of Exp is not great enough to exclude the possibility that the difference is just due to random sampling variability after allowing for the effects of differences in Cd conc. There is not a statistically significant difference (P = 0.358).

The difference in the mean values among the different levels of Cd conc is greater than would be expected by chance after allowing for effects of differences in Exp. There is a statistically significant difference (P = 0.035). To isolate which group(s) differ from the others use a multiple comparison procedure.

Power of performed test with alpha = 0.0500: for Exp : 0.0664
Power of performed test with alpha = 0.0500: for Cd conc : 0.533

Least square means for Exp :

Group	Mean	SEM
272.000	14.507	16.767
273.000	18.658	11.286
278.000	6.618	10.542
280.000	34.146	11.259

Least square means for Cd conc :

Group	Mean	SEM
0.5	2.981	16.338
20	0.803	16.338
50	3.170	16.338
140	78.508	16.338
270	29.640	16.338
550	28.259	17.650
1270	19.387	17.650
10	-0.550	18.073
3100	4.144	31.007

All Pairwise Multiple Comparison Procedures (Holm-Sidak method):
Overall significance level = 0.05

Comparisons for factor: **Exp**

Comparison	Diff of Means	t	P	P<0.050
280.000 vs. 278.000	27.528	1.785	0.399	No
280.000 vs. 272.000	19.639	0.972	0.871	No
280.000 vs. 273.000	15.488	0.972	0.806	No
273.000 vs. 278.000	12.040	0.780	0.824	No
272.000 vs. 278.000	7.888	0.398	0.905	No
273.000 vs. 272.000	4.151	0.205	0.838	No

Comparisons for factor: **Cd conc**

Comparison	Diff of Means	t	P	P<0.050
140 vs. 20	77.704	.363	0.057	No
140 vs. 0.5	75.527	3.269	0.072	No
140 vs. 50	75.338	3.261	0.071	No
140 vs. 10	79.057	3.245	0.072	No
140 vs. 1270	59.121	2.458	0.442	No
140 vs. 3100	74.363	2.122	0.715	No
140 vs. 270	48.867	2.115	0.709	No
140 vs. 550	50.248	2.089	0.717	No
270 vs. 20	28.837	1.248	0.999	No
270 vs. 10	30.190	1.239	0.999	No

270 vs. 0.5	26.659	1.154	1.000	No
270 vs. 50	26.470	1.146	0.999	No
550 vs. 20	27.456	1.142	0.999	No
550 vs. 10	28.809	1.140	0.999	No
550 vs. 0.5	25.278	1.051	1.000	No
550 vs. 50	25.089	1.043	0.999	No
1270 vs. 10	19.936	0.789	1.000	No
1270 vs. 20	18.583	0.773	1.000	No
270 vs. 3100	25.496	0.727	1.000	No
1270 vs. 0.5	16.406	0.682	1.000	No
550 vs. 3100	24.115	0.676	1.000	No
1270 vs. 50	16.217	0.674	1.000	No
1270 vs. 3100	15.242	0.427	1.000	No
270 vs. 1270	10.254	0.426	1.000	No
550 vs. 1270	8.873	0.355	1.000	No
50 vs. 10	3.720	0.153	1.000	No
0.5 vs. 10	3.531	0.145	1.000	No
3100 vs. 10	4.694	0.131	1.000	No
50 vs. 20	2.367	0.102	1.000	No
3100 vs. 20	3.341	0.0953	1.000	No
0.5 vs. 20	2.178	0.0943	1.000	No
270 vs. 550	1.381	0.0574	1.000	No
20 vs. 10	1.353	0.0555	1.000	No
3100 vs. 0.5	1.164	0.0332	1.000	No
3100 vs. 50	0.975	0.0278	1.000	No
50 vs. 0.5	0.189	0.00818	0.994	No

Elements in the plant tissues - Nickel - w5 leaves

Two Way Analysis of Variance
General Linear Model (No Interactions)
Dependent Variable: Ni w5
Normality Test (Shapiro-Wilk) Passed (P = 0.358)
Equal Variance Test: Failed (P < 0.050)

**Source of Variation**	**DF**	**SS**	**MS**	**F**	**P**
Exp	3	235.773	78.591	2.764	0.049
Cd conc	9	1568.005	174.223	6.127	<0.001
Residual	62	1762.870	28.433		
Total	74	3738.466	50.520		

The difference in the mean values among the different levels of Exp is greater than would be expected by chance after allowing for effects of differences in Cd conc. There is a statistically significant difference (P = 0.049). To isolate which group(s) differ from the others use a multiple comparison procedure.

The difference in the mean values among the different levels of Cd conc is greater than would be expected by chance after allowing for effects of differences in Exp. There is a statistically significant difference (P = <0.001). To isolate which group(s) differ from the others use a multiple comparison procedure.

Power of performed test with alpha = 0.0500: for Exp : 0.429
Power of performed test with alpha = 0.0500: for Cd conc : 0.999

Least square means for Exp :

**Group**	**Mean**	**SEM**
272.000	17.362	1.278
273.000	16.569	1.192
278.000	19.849	1.229
280.000	21.003	1.282

Least square means for Cd conc :

Group	Mean	SEM
0.5	20.244	1.885
20	22.141	1.885
50	21.299	1.885
140	24.361	1.885
270	23.231	1.885
550	20.697	1.885
1270	16.428	1.885
3100	13.618	1.885
27500	6.538	2.421
10	18.404	2.208

All Pairwise Multiple Comparison Procedures (Holm-Sidak method):
Overall significance level = 0.05

Comparisons for factor: **Exp**

Comparison	Diff of Means	t	P	P<0.050
280.000 vs. 273.000	4.434	2.533	0.080	No
280.000 vs. 272.000	3.642	2.012	0.220	No
278.000 vs. 273.000	3.280	1.915	0.219	No
278.000 vs. 272.000	2.488	1.403	0.419	No
280.000 vs. 278.000	1.154	0.650	0.768	No
272.000 vs. 273.000	0.792	0.453	0.652	No

Comparisons for factor: **Cd conc**

Comparison	Diff of Means	t	P	P<0.050
140 vs. 27500	17.823	5.808	<0.001	Yes
270 vs. 27500	16.693	5.439	<0.001	Yes
20 vs. 27500	15.603	5.084	<0.001	Yes
50 vs. 27500	14.761	4.810	<0.001	Yes
550 vs. 27500	14.159	4.614	<0.001	Yes
0.5 vs. 27500	13.706	4.466	0.001	Yes
140 vs. 3100	10.743	4.029	0.006	Yes
10 vs. 27500	11.866	3.621	0.022	Yes
270 vs. 3100	9.613	3.605	0.023	Yes
1270 vs. 27500	9.890	3.223	0.070	No
20 vs. 3100	8.523	3.197	0.074	No
140 vs. 1270	7.933	2.975	0.132	No
50 vs. 3100	7.681	2.881	0.165	No
550 vs. 3100	7.079	2.655	0.276	No
270 vs. 1270	6.803	2.552	0.338	No
0.5 vs. 3100	6.626	2.485	0.377	No
3100 vs. 27500	7.080	2.307	0.512	No
20 vs. 1270	5.714	2.143	0.642	No
140 vs. 10	5.957	2.052	0.707	No
50 vs. 1270	4.872	1.827	0.859	No
270 vs. 10	4.827	1.663	0.931	No
10 vs. 3100	4.786	1.648	0.929	No
550 vs. 1270	4.269	1.601	0.939	No
140 vs. 0.5	4.117	1.544	0.950	No
0.5 vs. 1270	3.816	1.431	0.973	No
140 vs. 550	3.664	1.374	0.978	No
20 vs. 10	3.738	1.287	0.986	No
140 vs. 50	3.061	1.148	0.995	No
270 vs. 0.5	2.987	1.120	0.995	No
1270 vs. 3100	2.810	1.054	0.996	No
50 vs. 10	2.896	0.997	0.997	No
270 vs. 550	2.534	0.950	0.997	No
140 vs. 20	2.220	0.832	0.999	No
550 vs. 10	2.293	0.790	0.999	No
270 vs. 50	1.931	0.724	0.999	No

20 vs. 0.5	1.898	0.712	0.999	No
10 vs. 1270	1.976	0.681	0.998	No
0.5 vs. 10	1.840	0.634	0.998	No
20 vs. 550	1.444	0.542	0.998	No
140 vs. 270	1.130	0.424	0.999	No
270 vs. 20	1.089	0.409	0.997	No
50 vs. 0.5	1.056	0.396	0.991	No
20 vs. 50	0.842	0.316	0.985	No
50 vs. 550	0.603	0.226	0.968	No
550 vs. 0.5	0.453	0.170	0.866	No

Elements in the plant tissues - Nickel - Stems

Two Way Analysis of Variance
General Linear Model (No Interactions)
Dependent Variable: Ni stems
Normality Test (Shapiro-Wilk) Failed (P < 0.050)
Equal Variance Test: Passed (P = 0.624)

Source of Variation	DF	SS	MS	F	P
Exp	3	194.920	64.973	1.381	0.252
Cd conc	9	4367.109	485.234	10.311	<0.001
Residual	127	5976.430	47.059		
Total	139	10871.720	78.214		

The difference in the mean values among the different levels of Exp is not great enough to exclude the possibility that the difference is just due to random sampling variability after allowing for the effects of differences in Cd conc. There is not a statistically significant difference (P = 0.252).

The difference in the mean values among the different levels of Cd conc is greater than would be expected by chance after allowing for effects of differences in Exp. There is a statistically significant difference (P = <0.001). To isolate which group(s) differ from the others use a multiple comparison procedure.

Power of performed test with alpha = 0.0500: for Exp : 0.120
Power of performed test with alpha = 0.0500: for Cd conc : 1.000

Least square means for Exp :

Group	Mean	SEM
272.000	17.143	1.183
273.000	15.057	1.085
278.000	14.025	1.354
280.000	14.281	1.115

Least square means for Cd conc :

Group	Mean	SEM
0.5	7.347	1.715
20	7.033	1.715
50	11.451	1.715
140	17.661	1.840
270	19.561	2.016
550	17.580	1.840
1270	18.165	1.835
3100	24.813	1.998
27500	18.717	1.715
10	8.936	2.197

All Pairwise Multiple Comparison Procedures (Holm-Sidak method):
Overall significance level = 0.05

Comparisons for factor: **Exp**

Comparison	Diff of Means	t	P	P<0.050
272.000 vs. 280.000	2.863	1.761	0.396	No
272.000 vs. 278.000	3.118	1.734	0.360	No
272.000 vs. 273.000	2.087	1.300	0.582	No
273.000 vs. 278.000	1.031	0.594	0.911	No
273.000 vs. 280.000	0.776	0.499	0.855	No
280.000 vs. 278.000	0.255	0.146	0.884	No

Comparisons for factor: **Cd conc**

Comparison	Diff of Means	t	P	P<0.050
3100 vs. 20	17.780	6.753	<0.001	Yes
3100 vs. 0.5	17.467	6.634	<0.001	Yes
3100 vs. 10	15.877	5.347	<0.001	Yes
3100 vs. 50	13.362	5.075	<0.001	Yes
27500 vs. 20	11.684	4.817	<0.001	Yes
270 vs. 20	12.528	4.734	<0.001	Yes
27500 vs. 0.5	11.371	4.688	<0.001	Yes
270 vs. 0.5	12.214	4.615	<0.001	Yes
1270 vs. 20	11.132	4.432	<0.001	Yes
1270 vs. 0.5	10.818	4.307	0.001	Yes
140 vs. 20	10.628	4.225	0.002	Yes
550 vs. 20	10.547	4.192	0.002	Yes
140 vs. 0.5	10.314	4.100	0.002	Yes
550 vs. 0.5	10.233	4.068	0.003	Yes
270 vs. 10	10.625	3.563	0.016	Yes
27500 vs. 10	9.781	3.509	0.019	Yes
1270 vs. 10	9.229	3.224	0.046	Yes
270 vs. 50	8.110	3.064	0.072	No
140 vs. 10	8.724	3.044	0.074	No
550 vs. 10	8.643	3.016	0.077	No
27500 vs. 50	7.266	2.996	0.079	No
1270 vs. 50	6.714	2.673	0.185	No
3100 vs. 550	7.233	2.663	0.183	No
3100 vs. 140	7.152	2.633	0.190	No
140 vs. 50	6.209	2.468	0.270	No
3100 vs. 1270	6.648	2.451	0.270	No
550 vs. 50	6.129	2.436	0.267	No
3100 vs. 27500	6.096	2.315	0.332	No
3100 vs. 270	5.252	1.851	0.690	No
50 vs. 20	4.418	1.822	0.692	No
50 vs. 0.5	4.105	1.692	0.769	No
50 vs. 10	2.515	0.902	0.998	No
270 vs. 550	1.981	0.726	1.000	No
270 vs. 140	1.900	0.696	1.000	No
10 vs. 20	1.903	0.683	0.999	No
10 vs. 0.5	1.590	0.570	1.000	No
270 vs. 1270	1.396	0.512	1.000	No
27500 vs. 550	1.137	0.452	1.000	No
27500 vs. 140	1.056	0.420	1.000	No
270 vs. 27500	0.844	0.319	1.000	No
1270 vs. 550	0.585	0.225	1.000	No
27500 vs. 1270	0.552	0.220	0.999	No
1270 vs. 140	0.504	0.194	0.996	No
0.5 vs. 20	0.313	0.129	0.989	No
140 vs. 550	0.0808	0.0310	0.975	No

Elements in the plant tissues - Nickel - Roots

Two Way Analysis of Variance
General Linear Model (No Interactions)

Dependent Variable: Ni roots
Normality Test (Shapiro-Wilk) Failed (P < 0.050)
Equal Variance Test: Passed (P = 1.000)

**Source of Variation**	**DF**	**SS**	**MS**	**F**	**P**
Exp	3	50916.965	16972.322	9.449	<0.001
Cd conc	9	14499.885	1611.098	0.897	0.542
Residual	25	44903.387	1796.135		
Total	37	111616.262	3016.656		

The difference in the mean values among the different levels of Exp is greater than would be expected by chance after allowing for effects of differences in Cd conc. There is a statistically significant difference (P = <0.001). To isolate which group(s) differ from the others use a multiple comparison procedure.

The difference in the mean values among the different levels of Cd conc is not great enough to exclude the possibility that the difference is just due to random sampling variability after allowing for the effects of differences in Exp. There is not a statistically significant difference (P = 0.542).

Power of performed test with alpha = 0.0500: for Exp : 0.988
Power of performed test with alpha = 0.0500: for Cd conc : 0.0500

Least square means for Exp :

**Group**	**Mean**	**SEM**
272.000	26.623	14.476
273.000	35.853	13.402
278.000	46.531	13.402
280.000	121.601	14.476

Least square means for Cd conc :

**Group**	**Mean**	**SEM**
0.5	34.954	21.190
20	36.615	21.190
50	39.247	21.190
140	75.659	21.190
270	96.504	21.190
550	74.249	21.190
1270	52.584	21.190
3100	68.048	21.190
27500	52.093	21.190
10	46.566	30.789

All Pairwise Multiple Comparison Procedures (Holm-Sidak method):
Overall significance level = 0.05

Comparisons for factor: **Exp**

**Comparison**	**Diff of Means**	**t**	**P**	**P<0.050**
280.000 vs. 272.000	94.978	4.639	<0.001	Yes
280.000 vs. 273.000	85.747	4.347	0.001	Yes
280.000 vs. 278.000	75.070	3.805	0.003	Yes
278.000 vs. 272.000	19.908	1.009	0.689	No
278.000 vs. 273.000	10.677	0.563	0.822	No
273.000 vs. 272.000	9.231	0.468	0.644	No

Comparisons for factor: **Cd conc**

**Comparison**	**Diff of Means**	**t**	**P**	**P<0.050**
270 vs. 0.5	61.550	2.054	0.903	No
270 vs. 20	59.889	1.998	0.923	No
270 vs. 50	57.256	1.911	0.951	No
270 vs. 27500	44.411	1.482	0.999	No
270 vs. 1270	43.919	1.466	0.999	No
140 vs. 0.5	40.705	1.358	1.000	No

270 vs. 10	49.938	1.336	1.000	No
550 vs. 0.5	39.295	1.311	1.000	No
140 vs. 20	39.045	1.303	1.000	No
550 vs. 20	37.634	1.256	1.000	No
140 vs. 50	36.412	1.215	1.000	No
550 vs. 50	35.002	1.168	1.000	No
3100 vs. 0.5	33.094	1.104	1.000	No
3100 vs. 20	31.434	1.049	1.000	No
3100 vs. 50	28.801	0.961	1.000	No
270 vs. 3100	28.455	0.950	1.000	No
140 vs. 27500	23.567	0.786	1.000	No
140 vs. 10	29.094	0.778	1.000	No
140 vs. 1270	23.075	0.770	1.000	No
270 vs. 550	22.255	0.743	1.000	No
550 vs. 10	27.683	0.741	1.000	No
550 vs. 27500	22.156	0.739	1.000	No
550 vs. 1270	21.665	0.723	1.000	No
270 vs. 140	20.844	0.696	1.000	No
1270 vs. 0.5	17.630	0.588	1.000	No
3100 vs. 10	21.483	0.575	1.000	No
27500 vs. 0.5	17.139	0.572	1.000	No
1270 vs. 20	15.969	0.533	1.000	No
3100 vs. 27500	15.956	0.532	1.000	No
27500 vs. 20	15.478	0.516	1.000	No
3100 vs. 1270	15.464	0.516	1.000	No
1270 vs. 50	13.337	0.445	1.000	No
27500 vs. 50	12.846	0.429	1.000	No
10 vs. 0.5	11.612	0.311	1.000	No
10 vs. 20	9.951	0.266	1.000	No
140 vs. 3100	7.611	0.254	1.000	No
550 vs. 3100	6.200	0.207	1.000	No
10 vs. 50	7.319	0.196	1.000	No
1270 vs. 10	6.018	0.161	1.000	No
27500 vs. 10	5.527	0.148	1.000	No
50 vs. 0.5	4.293	0.143	1.000	No
50 vs. 20	2.632	0.0878	1.000	No
20 vs. 0.5	1.661	0.0554	1.000	No
140 vs. 550	1.411	0.0471	0.999	No
1270 vs. 27500	0.491	0.0164	0.987	No

Elements in the plant tissues - Nickel - Seeds

Two Way Analysis of Variance
General Linear Model (No Interactions)
Dependent Variable: Ni seeds
Normality Test (Shapiro-Wilk) Passed (P = 0.792)
Equal Variance Test: Passed (P = 0.393)

**Source of Variation**	**DF**	**SS**	**MS**	**F**	**P**
Exp	3	225.215	75.072	0.898	0.449
Cd conc	3	2231.509	743.836	8.894	<0.001
Residual	52	4349.062	83.636		
Total	58	6759.311	116.540		

The difference in the mean values among the different levels of Exp is not great enough to exclude the possibility that the difference is just due to random sampling variability after allowing for the effects of differences in Cd conc. There is not a statistically significant difference (P = 0.449).

The difference in the mean values among the different levels of Cd conc is greater than would be expected by chance after allowing for effects of differences in Exp. There is a statistically significant difference (P = <0.001).

To isolate which group(s) differ from the others use a multiple comparison procedure.

Power of performed test with alpha = 0.0500: for Exp : 0.0500
Power of performed test with alpha = 0.0500: for Cd conc : 0.990

Least square means for Exp :

**Group**	**Mean**	**SEM**
272.000	34.059	2.755
273.000	37.624	2.286
278.000	32.987	2.367
280.000	37.046	2.286

Least square means for Cd conc :

**Group**	**Mean**	**SEM**
0.5	33.680	2.286
20	30.700	2.286
50	45.697	2.286
10	31.639	2.866

All Pairwise Multiple Comparison Procedures (Holm-Sidak method):
Overall significance level = 0.05

Comparisons for factor: **Exp**

**Comparison**	**Diff of Means**	**t**	**P**	**P<0.050**
273.000 vs. 278.000	4.637	1.409	0.660	No
280.000 vs. 278.000	4.059	1.234	0.717	No
273.000 vs. 272.000	3.565	0.996	0.791	No
280.000 vs. 272.000	2.987	0.834	0.792	No
272.000 vs. 278.000	1.072	0.295	0.947	No
273.000 vs. 280.000	0.578	0.179	0.859	No

Comparisons for factor: **Cd conc**

**Comparison**	**Diff of Means**	**t**	**P**	**P<0.050**
50 vs. 20	14.997	4.638	<0.001	Yes
50 vs. 10	14.058	3.834	0.002	Yes
50 vs. 0.5	12.017	3.717	0.002	Yes
0.5 vs. 20	2.980	0.922	0.739	No
0.5 vs. 10	2.041	0.557	0.824	No
10 vs. 20	0.939	0.256	0.799	No

Elements in the plant tissues - Zinc - Seeds

Two Way Analysis of Variance
General Linear Model (No Interactions)
Dependent Variable: Zn seeds
Normality Test (Shapiro-Wilk) Failed (P < 0.050)
Equal Variance Test: Failed (P < 0.050)

**Source of Variation**	**DF**	**SS**	**MS**	**F**	**P**
Exp	3	131.712	43.904	2.455	0.073
Cd conc	3	507.301	169.100	9.455	<0.001
Residual	52	930.025	17.885		
Total	58	1533.674	26.443		

The difference in the mean values among the different levels of Exp is not great enough to exclude the possibility that the difference is just due to random sampling variability after allowing for the effects of differences in Cd conc. There is not a statistically significant difference (P = 0.073).

The difference in the mean values among the different levels of Cd conc is greater than would be expected by

chance after allowing for effects of differences in Exp. There is a statistically significant difference (P = <0.001). To isolate which group(s) differ from the others use a multiple comparison procedure.

Power of performed test with alpha = 0.0500: for Exp : 0.354
Power of performed test with alpha = 0.0500: for Cd conc : 0.994

Least square means for Exp :

**Group**	**Mean**	**SEM**
272.000	23.887	1.274
273.000	28.204	1.057
278.000	25.673	1.094
280.000	26.838	1.057

Least square means for Cd conc :

**Group**	**Mean**	**SEM**
0.5	25.746	1.057
20	23.971	1.057
50	31.016	1.057
10	23.870	1.325

All Pairwise Multiple Comparison Procedures (Holm-Sidak method):
Overall significance level = 0.05

Comparisons for factor: **Exp**

**Comparison**	**Diff of Means**	**t**	**P**	**P<0.050**
273.000 vs. 272.000	4.318	2.608	0.069	No
280.000 vs. 272.000	2.952	1.783	0.343	No
273.000 vs. 278.000	2.531	1.663	0.350	No
278.000 vs. 272.000	1.787	1.064	0.646	No
273.000 vs. 280.000	1.366	0.914	0.597	No
280.000 vs. 278.000	1.165	0.766	0.447	No

Comparisons for factor: **Cd conc**

**Comparison**	**Diff of Means**	**t**	**P**	**P<0.050**
50 vs. 20	7.045	4.712	<0.001	Yes
50 vs. 10	7.146	4.215	<0.001	Yes
50 vs. 0.5	5.270	3.525	0.004	Yes
0.5 vs. 20	1.775	1.187	0.562	No
0.5 vs. 10	1.875	1.106	0.473	No
20 vs. 10	0.101	0.0593	0.953	No

Elements in the plant tissues - Zinc - Roots

Two Way Analysis of Variance
General Linear Model (No Interactions)
Dependent Variable: Zn roots
Normality Test (Shapiro-Wilk) Failed (P < 0.050)
Equal Variance Test: Passed (P = 1.000)

**Source of Variation**	**DF**	**SS**	**MS**	**F**	**P**
Exp	3	121852.083	40617.361	6.516	0.002
Cd conc	9	54521.291	6057.921	0.972	0.486
Residual	25	155840.288	6233.612		
Total	37	331674.339	8964.171		

The difference in the mean values among the different levels of Exp is greater than would be expected by chance after allowing for effects of differences in Cd conc. There is a statistically significant difference (P = 0.002). To isolate which group(s) differ from the others use a multiple comparison procedure.

The difference in the mean values among the different levels of Cd conc is not great enough to exclude the

possibility that the difference is just due to random sampling variability after allowing for the effects of differences in Exp. There is not a statistically significant difference (P = 0.486).

Power of performed test with alpha = 0.0500: for Exp : 0.907
Power of performed test with alpha = 0.0500: for Cd conc : 0.0500

Least square means for Exp :

**Group**	**Mean**	**SEM**
272.000	85.190	26.968
273.000	40.918	24.967
278.000	70.619	24.967
280.000	194.530	26.968

Least square means for Cd conc :

**Group**	**Mean**	**SEM**
0.5	57.661	39.477
20	45.315	39.477
50	32.503	39.477
140	115.260	39.477
270	113.317	39.477
550	108.103	39.477
1270	102.816	39.477
3100	153.316	39.477
27500	129.743	39.477
10	120.110	57.358

All Pairwise Multiple Comparison Procedures (Holm-Sidak method):
Overall significance level = 0.05

Comparisons for factor: **Exp**

**Comparison**	**Diff of Means**	**t**	**P**	**P<0.050**
280.000 vs. 273.000	153.612	4.180	0.002	Yes
280.000 vs. 278.000	123.911	3.372	0.012	Yes
280.000 vs. 272.000	109.340	2.867	0.033	Yes
272.000 vs. 273.000	44.272	1.205	0.560	No
278.000 vs. 273.000	29.701	0.841	0.650	No
272.000 vs. 278.000	14.571	0.396	0.695	No

Comparisons for factor: **Cd conc**

**Comparison**	**Diff of Means**	**t**	**P**	**P<0.050**
3100 vs. 50	120.812	2.164	0.842	No
3100 vs. 20	108.001	1.935	0.947	No
27500 vs. 50	97.240	1.742	0.986	No
3100 vs. 0.5	95.655	1.713	0.987	No
27500 vs. 20	84.428	1.512	0.998	No
140 vs. 50	82.756	1.482	0.999	No
270 vs. 50	80.814	1.448	0.999	No
550 vs. 50	75.600	1.354	1.000	No
27500 vs. 0.5	72.082	1.291	1.000	No
1270 vs. 50	70.312	1.259	1.000	No
10 vs. 50	87.606	1.258	1.000	No
140 vs. 20	69.945	1.253	1.000	No
270 vs. 20	68.002	1.218	1.000	No
550 vs. 20	62.788	1.125	1.000	No
10 vs. 20	74.795	1.074	1.000	No
140 vs. 0.5	57.599	1.032	1.000	No
1270 vs. 20	57.501	1.030	1.000	No
270 vs. 0.5	55.656	0.997	1.000	No
3100 vs. 1270	50.500	0.905	1.000	No
550 vs. 0.5	50.443	0.904	1.000	No
10 vs. 0.5	62.449	0.897	1.000	No

3100 vs. 550	45.212	0.810	1.000	No
1270 vs. 0.5	45.155	0.809	1.000	No
3100 vs. 270	39.999	0.716	1.000	No
3100 vs. 140	38.056	0.682	1.000	No
27500 vs. 1270	26.927	0.482	1.000	No
3100 vs. 10	33.206	0.477	1.000	No
0.5 vs. 50	25.157	0.451	1.000	No
3100 vs. 27500	23.573	0.422	1.000	No
27500 vs. 550	21.640	0.388	1.000	No
27500 vs. 270	16.426	0.294	1.000	No
27500 vs. 140	14.483	0.259	1.000	No
10 vs. 1270	17.294	0.248	1.000	No
20 vs. 50	12.812	0.229	1.000	No
140 vs. 1270	12.444	0.223	1.000	No
0.5 vs. 20	12.346	0.221	1.000	No
270 vs. 1270	10.501	0.188	1.000	No
10 vs. 550	12.007	0.172	1.000	No
27500 vs. 10	9.633	0.138	1.000	No
140 vs. 550	7.157	0.128	1.000	No
10 vs. 270	6.793	0.0976	1.000	No
550 vs. 1270	5.287	0.0947	1.000	No
270 vs. 550	5.214	0.0934	1.000	No
10 vs. 140	4.850	0.0697	0.997	No
140 vs. 270	1.943	0.0348	0.973	No

Elements in the plant tissues - Zinc - w10 leaves

Two Way Analysis of Variance
General Linear Model (No Interactions)
Dependent Variable: Zn w10
Normality Test (Shapiro-Wilk) Failed (P < 0.050)
Equal Variance Test: Failed (P < 0.050)

Source of Variation	DF	SS	MS	F	P
Exp	3	7482.040	2494.013	0.639	0.594
Cd conc	8	121080.165	15135.021	3.876	0.002
Residual	43	167885.417	3904.312		
Total	54	296118.315	5483.673		

The difference in the mean values among the different levels of Exp is not great enough to exclude the possibility that the difference is just due to random sampling variability after allowing for the effects of differences in Cd conc. There is not a statistically significant difference (P = 0.594).

The difference in the mean values among the different levels of Cd conc is greater than would be expected by chance after allowing for effects of differences in Exp. There is a statistically significant difference (P = 0.002). To isolate which group(s) differ from the others use a multiple comparison procedure.

Power of performed test with alpha = 0.0500: for Exp : 0.0500
Power of performed test with alpha = 0.0500: for Cd conc : 0.909

Least square means for Exp :

Group	Mean	SEM
272.000	29.753	24.401
273.000	50.704	16.424
278.000	35.864	15.342
280.000	61.652	16.385

Least square means for Cd conc :

Group	Mean	SEM
0.5	16.250	23.776

20	17.208	23.776
50	24.846	23.776
140	163.199	23.776
270	64.728	23.776
550	45.958	25.686
1270	29.260	25.686
10	18.089	26.301
3100	20.901	45.124

All Pairwise Multiple Comparison Procedures (Holm-Sidak method):
Overall significance level = 0.05

Comparisons for factor: **Exp**

**Comparison**	**Diff of Means**	**t**	**P**	**P<0.050**
280.000 vs. 278.000	25.788	1.149	0.832	No
280.000 vs. 272.000	31.899	1.085	0.812	No
273.000 vs. 272.000	20.951	0.712	0.927	No
273.000 vs. 278.000	14.841	0.660	0.884	No
280.000 vs. 273.000	10.947	0.472	0.870	No
278.000 vs. 272.000	6.111	0.212	0.833	No

Comparisons for factor: **Cd conc**

**Comparison**	**Diff of Means**	**t**	**P**	**P<0.050**
140 vs. 0.5	146.949	4.370	0.003	Yes
140 vs. 20	145.991	4.342	0.003	Yes
140 vs. 50	138.353	4.115	0.006	Yes
140 vs. 10	145.110	4.093	0.006	Yes
140 vs. 1270	133.939	3.827	0.013	Yes
140 vs. 550	117.241	3.350	0.051	No
140 vs. 270	98.471	2.929	0.151	No
140 vs. 3100	142.298	2.790	0.204	No
270 vs. 0.5	48.478	1.442	0.992	No
270 vs. 20	47.520	1.413	0.992	No
270 vs. 10	46.638	1.315	0.996	No
270 vs. 50	39.882	1.186	0.999	No
270 vs. 1270	35.468	1.013	1.000	No
270 vs. 3100	43.826	0.859	1.000	No
550 vs. 0.5	29.708	0.849	1.000	No
550 vs. 20	28.750	0.821	1.000	No
550 vs. 10	27.868	0.758	1.000	No
550 vs. 50	21.112	0.603	1.000	No
270 vs. 550	18.770	0.536	1.000	No
550 vs. 3100	25.056	0.483	1.000	No
550 vs. 1270	16.698	0.460	1.000	No
1270 vs. 0.5	13.010	0.372	1.000	No
1270 vs. 20	12.052	0.344	1.000	No
1270 vs. 10	11.171	0.304	1.000	No
50 vs. 0.5	8.596	0.256	1.000	No
50 vs. 20	7.638	0.227	1.000	No
50 vs. 10	6.757	0.191	1.000	No
1270 vs. 3100	8.359	0.161	1.000	No
1270 vs. 50	4.414	0.126	1.000	No
3100 vs. 0.5	4.652	0.0912	1.000	No
50 vs. 3100	3.945	0.0773	1.000	No
3100 vs. 20	3.694	0.0724	1.000	No
3100 vs. 10	2.812	0.0538	1.000	No
10 vs. 0.5	1.840	0.0519	1.000	No
20 vs. 0.5	0.958	0.0285	0.999	No
10 vs. 20	0.882	0.0249	0.980	No

Elements in the plant tissues - Zinc - w5 leaves

Two Way Analysis of Variance
General Linear Model (No Interactions)
Dependent Variable: Zn w5
Normality Test (Shapiro-Wilk) Passed (P = 0.224)
Equal Variance Test: Failed (P < 0.050)

**Source of Variation**	**DF**	**SS**	**MS**	**F**	**P**
Exp	3	2297.122	765.707	5.897	0.001
Cd conc	9	5834.395	648.266	4.992	<0.001
Residual	62	8050.669	129.849		
Total	74	16121.203	217.854		

The difference in the mean values among the different levels of Exp is greater than would be expected by chance after allowing for effects of differences in Cd conc. There is a statistically significant difference (P = 0.001). To isolate which group(s) differ from the others use a multiple comparison procedure.

The difference in the mean values among the different levels of Cd conc is greater than would be expected by chance after allowing for effects of differences in Exp. There is a statistically significant difference (P = <0.001). To isolate which group(s) differ from the others use a multiple comparison procedure.

Power of performed test with alpha = 0.0500: for Exp : 0.901
Power of performed test with alpha = 0.0500: for Cd conc : 0.992

Least square means for Exp :

**Group**	**Mean**	**SEM**
272.000	31.688	2.731
273.000	30.794	2.548
278.000	41.698	2.627
280.000	26.597	2.739

Least square means for Cd conc :

**Group**	**Mean**	**SEM**
0.5	25.942	4.029
20	23.775	4.029
50	25.322	4.029
140	49.444	4.029
270	40.248	4.029
550	39.066	4.029
1270	42.383	4.029
3100	28.721	4.029
27500	27.100	5.175
10	24.937	4.718

All Pairwise Multiple Comparison Procedures (Holm-Sidak method):
Overall significance level = 0.05

Comparisons for factor: **Exp**

**Comparison**	**Diff of Means**	**t**	**P**	**P<0.050**
278.000 vs. 280.000	15.101	3.979	0.001	Yes
278.000 vs. 273.000	10.904	2.980	0.020	Yes
278.000 vs. 272.000	10.010	2.642	0.041	Yes
272.000 vs. 280.000	5.091	1.316	0.474	No
273.000 vs. 280.000	4.197	1.122	0.462	No
272.000 vs. 273.000	0.894	0.239	0.812	No

Comparisons for factor: **Cd conc**

**Comparison**	**Diff of Means**	**t**	**P**	**P<0.050**
140 vs. 20	25.669	4.505	0.001	Yes
140 vs. 50	24.122	4.234	0.003	Yes

140 vs. 0.5	23.502	4.125	0.005	Yes
140 vs. 10	24.507	3.950	0.008	Yes
140 vs. 3100	20.723	3.637	0.023	Yes
140 vs. 27500	22.344	3.407	0.045	Yes
1270 vs. 20	18.607	3.266	0.067	No
1270 vs. 50	17.061	2.994	0.140	No
270 vs. 20	16.473	2.891	0.178	No
1270 vs. 0.5	16.441	2.886	0.176	No
1270 vs. 10	17.445	2.812	0.206	No
550 vs. 20	15.290	2.684	0.273	No
270 vs. 50	14.926	2.620	0.307	No
270 vs. 0.5	14.306	2.511	0.377	No
270 vs. 10	15.311	2.468	0.400	No
550 vs. 50	13.744	2.412	0.435	No
1270 vs. 3100	13.661	2.398	0.435	No
1270 vs. 27500	15.282	2.330	0.480	No
550 vs. 0.5	13.124	2.303	0.490	No
550 vs. 10	14.128	2.277	0.499	No
270 vs. 3100	11.527	2.023	0.703	No
270 vs. 27500	13.148	2.005	0.703	No
550 vs. 27500	11.965	1.825	0.825	No
140 vs. 550	10.378	1.822	0.813	No
550 vs. 3100	10.344	1.816	0.802	No
140 vs. 270	9.196	1.614	0.906	No
140 vs. 1270	7.062	1.239	0.991	No
3100 vs. 20	4.946	0.868	1.000	No
3100 vs. 10	3.784	0.610	1.000	No
3100 vs. 50	3.399	0.597	1.000	No
1270 vs. 550	3.317	0.582	1.000	No
27500 vs. 20	3.325	0.507	1.000	No
3100 vs. 0.5	2.780	0.488	1.000	No
0.5 vs. 20	2.166	0.380	1.000	No
1270 vs. 270	2.134	0.375	1.000	No
27500 vs. 10	2.163	0.309	1.000	No
50 vs. 20	1.547	0.271	1.000	No
27500 vs. 50	1.778	0.271	1.000	No
3100 vs. 27500	1.621	0.247	1.000	No
270 vs. 550	1.182	0.208	1.000	No
10 vs. 20	1.162	0.187	1.000	No
27500 vs. 0.5	1.159	0.177	1.000	No
0.5 vs. 10	1.004	0.162	0.998	No
0.5 vs. 50	0.620	0.109	0.993	No
50 vs. 10	0.385	0.0620	0.951	No

Elements in the plant tissues - Zinc - harvested leaves

Two Way Analysis of Variance
General Linear Model (No Interactions)
Dependent Variable: Zn harvest
Normality Test (Shapiro-Wilk) Failed (P < 0.050)
Equal Variance Test: Failed (P < 0.050)

**Source of Variation**	**DF**	**SS**	**MS**	**F**	**P**
Exp	3	25883.988	8627.996	3.234	0.025
Cd conc	9	43495.873	4832.875	1.811	0.073
Residual	115	306830.947	2668.095		
Total	127	375497.929	2956.677		

The difference in the mean values among the different levels of Exp is greater than would be expected by chance after allowing for effects of differences in Cd conc. There is a statistically significant difference (P = 0.025). To

isolate which group(s) differ from the others use a multiple comparison procedure.

The difference in the mean values among the different levels of Cd conc is not great enough to exclude the possibility that the difference is just due to random sampling variability after allowing for the effects of differences in Exp. There is not a statistically significant difference (P = 0.073).

Power of performed test with alpha = 0.0500: for Exp : 0.546
Power of performed test with alpha = 0.0500: for Cd conc : 0.381

Least square means for Exp :

**Group**	**Mean**	**SEM**
272.000	44.626	9.453
273.000	31.654	8.395
278.000	41.433	10.932
280.000	68.545	8.782

Least square means for Cd conc :

**Group**	**Mean**	**SEM**
0.5	19.781	12.913
20	41.046	12.913
50	40.547	15.881
140	64.614	14.338
270	58.985	15.596
550	48.782	14.955
1270	32.095	14.382
3100	39.638	16.679
27500	83.870	13.348
10	36.288	15.817

All Pairwise Multiple Comparison Procedures (Holm-Sidak method):
Overall significance level = 0.05

Comparisons for factor: **Exp**

**Comparison**	**Diff of Means**	**t**	**P**	**P<0.050**
280.000 vs. 273.000	36.892	3.037	0.018	Yes
280.000 vs. 278.000	27.112	1.933	0.249	No
280.000 vs. 272.000	23.919	1.854	0.240	No
272.000 vs. 273.000	12.973	1.026	0.667	No
278.000 vs. 273.000	9.779	0.710	0.729	No
272.000 vs. 278.000	3.193	0.221	0.826	No

Comparisons for factor: **Cd conc**

**Comparison**	**Diff of Means**	**t**	**P**	**P<0.050**
27500 vs. 0.5	64.089	3.451	0.035	Yes
27500 vs. 1270	51.775	2.639	0.342	No
140 vs. 0.5	44.833	2.323	0.614	No
27500 vs. 20	42.824	2.306	0.622	No
27500 vs. 10	47.582	2.299	0.620	No
27500 vs. 50	43.323	2.088	0.796	No
27500 vs. 3100	44.232	2.071	0.802	No
270 vs. 0.5	39.204	1.936	0.885	No
27500 vs. 550	35.088	1.750	0.959	No
140 vs. 1270	32.519	1.601	0.986	No
550 vs. 0.5	29.001	1.468	0.996	No
140 vs. 10	28.326	1.327	0.999	No
270 vs. 1270	26.890	1.268	1.000	No
140 vs. 20	23.568	1.221	1.000	No
27500 vs. 270	24.885	1.212	1.000	No
20 vs. 0.5	21.265	1.164	1.000	No
140 vs. 3100	24.976	1.136	1.000	No
140 vs. 50	24.067	1.125	1.000	No

270 vs. 10	22.697	1.022	1.000	No
50 vs. 0.5	20.766	1.015	1.000	No
27500 vs. 140	19.256	0.983	1.000	No
3100 vs. 0.5	19.857	0.941	1.000	No
270 vs. 20	17.939	0.886	1.000	No
270 vs. 3100	19.347	0.847	1.000	No
270 vs. 50	18.438	0.828	1.000	No
10 vs. 0.5	16.507	0.808	1.000	No
550 vs. 1270	16.687	0.804	1.000	No
140 vs. 550	15.832	0.764	1.000	No
1270 vs. 0.5	12.314	0.637	1.000	No
550 vs. 10	12.494	0.574	1.000	No
270 vs. 550	10.203	0.472	1.000	No
20 vs. 1270	8.951	0.463	1.000	No
550 vs. 3100	9.144	0.408	1.000	No
50 vs. 1270	8.452	0.394	1.000	No
550 vs. 20	7.736	0.392	1.000	No
550 vs. 50	8.235	0.378	1.000	No
3100 vs. 1270	7.543	0.342	1.000	No
140 vs. 270	5.629	0.266	1.000	No
20 vs. 10	4.758	0.233	1.000	No
10 vs. 1270	4.193	0.196	1.000	No
50 vs. 10	4.259	0.190	1.000	No
3100 vs. 10	3.350	0.146	1.000	No
20 vs. 3100	1.408	0.0668	1.000	No
50 vs. 3100	0.909	0.0395	0.999	No
20 vs. 50	0.499	0.0244	0.981	No

Elements in the plant tissues - Zinc - Stems

Two Way Analysis of Variance
General Linear Model (No Interactions)
Dependent Variable: Zn stems
Normality Test (Shapiro-Wilk) Failed (P < 0.050)
Equal Variance Test: Passed (P = 0.205)

Source of Variation	DF	SS	MS	F	P
Exp	3	6999.055	2333.018	7.714	<0.001
Cd conc	9	135326.789	15036.310	49.716	<0.001
Residual	127	38410.609	302.446		
Total	139	189943.416	1366.499		

The difference in the mean values among the different levels of Exp is greater than would be expected by chance after allowing for effects of differences in Cd conc. There is a statistically significant difference (P = <0.001). To isolate which group(s) differ from the others use a multiple comparison procedure.

The difference in the mean values among the different levels of Cd conc is greater than would be expected by chance after allowing for effects of differences in Exp. There is a statistically significant difference (P = <0.001). To isolate which group(s) differ from the others use a multiple comparison procedure.

Power of performed test with alpha = 0.0500: for Exp : 0.980
Power of performed test with alpha = 0.0500: for Cd conc : 1.000

Least square means for Exp :

Group	Mean	SEM
272.000	61.672	2.999
273.000	47.125	2.750
278.000	46.166	3.434
280.000	43.243	2.826

Least square means for Cd conc :

Group	Mean	SEM
0.5	13.983	4.348
20	8.184	4.348
50	11.348	4.348
140	85.663	4.666
270	89.491	5.110
550	70.962	4.666
1270	66.994	4.653
3100	71.494	5.064
27500	62.594	4.348
10	14.801	5.570

All Pairwise Multiple Comparison Procedures (Holm-Sidak method):
Overall significance level = 0.05

Comparisons for factor: **Exp**

Comparison	Diff of Means	t	P	P<0.050
272.000 vs. 280.000	18.430	4.472	<0.001	Yes
272.000 vs. 273.000	14.548	3.575	0.002	Yes
272.000 vs. 278.000	15.506	3.401	0.004	Yes
273.000 vs. 280.000	3.882	0.984	0.695	No
278.000 vs. 280.000	2.923	0.657	0.762	No
273.000 vs. 278.000	0.959	0.218	0.828	No

Comparisons for factor: **Cd conc**

Comparison	Diff of Means	t	P	P<0.050
140 vs. 20	77.478	12.149	<0.001	Yes
270 vs. 20	81.307	12.118	<0.001	Yes
140 vs. 50	74.315	11.653	<0.001	Yes
270 vs. 50	78.144	11.647	<0.001	Yes
270 vs. 0.5	75.508	11.254	<0.001	Yes
140 vs. 0.5	71.680	11.239	<0.001	Yes
270 vs. 10	74.690	9.881	<0.001	Yes
550 vs. 20	62.777	9.843	<0.001	Yes
140 vs. 10	70.861	9.752	<0.001	Yes
3100 vs. 20	63.310	9.485	<0.001	Yes
550 vs. 50	59.614	9.348	<0.001	Yes
1270 vs. 20	58.809	9.235	<0.001	Yes
3100 vs. 50	60.147	9.011	<0.001	Yes
550 vs. 0.5	56.979	8.934	<0.001	Yes
27500 vs. 20	54.410	8.849	<0.001	Yes
1270 vs. 50	55.646	8.738	<0.001	Yes
3100 vs. 0.5	57.511	8.616	<0.001	Yes
27500 vs. 50	51.247	8.335	<0.001	Yes
1270 vs. 0.5	53.011	8.324	<0.001	Yes
27500 vs. 0.5	48.611	7.906	<0.001	Yes
550 vs. 10	56.160	7.729	<0.001	Yes
3100 vs. 10	56.693	7.531	<0.001	Yes
1270 vs. 10	52.192	7.191	<0.001	Yes
27500 vs. 10	47.793	6.764	<0.001	Yes
270 vs. 27500	26.897	4.009	0.002	Yes
140 vs. 27500	23.068	3.617	0.009	Yes
270 vs. 1270	22.498	3.255	0.027	Yes
140 vs. 1270	18.669	2.833	0.092	No
270 vs. 550	18.530	2.678	0.133	No
270 vs. 3100	17.997	2.501	0.197	No
140 vs. 550	14.701	2.228	0.343	No
140 vs. 3100	14.169	2.058	0.449	No
3100 vs. 27500	8.900	1.333	0.930	No
550 vs. 27500	8.367	1.312	0.922	No

0.5 vs. 20	5.798	0.943	0.991	No
10 vs. 20	6.617	0.936	0.987	No
1270 vs. 27500	4.399	0.691	0.998	No
3100 vs. 1270	4.501	0.654	0.997	No
550 vs. 1270	3.968	0.602	0.996	No
270 vs. 140	3.829	0.553	0.995	No
50 vs. 20	3.163	0.514	0.991	No
10 vs. 50	3.454	0.489	0.980	No
0.5 vs. 50	2.635	0.429	0.964	No
10 vs. 0.5	0.819	0.116	0.992	No
3100 vs. 550	0.532	0.0773	0.938	No

Elements in the plant tissues -Calcium - Seeds

Two Way Analysis of Variance
General Linear Model (No Interactions)
Dependent Variable: Ca seeds
Normality Test (Shapiro-Wilk) Passed (P = 0.105)
Equal Variance Test: Failed (P < 0.050)

Source of Variation	DF	SS	MS	F	P
Exp	3	3.709	1.236	5.173	0.003
Cd conc	3	0.871	0.290	1.215	0.313
Residual	52	12.426	0.239		
Total	58	17.019	0.293		

The difference in the mean values among the different levels of Exp is greater than would be expected by chance after allowing for effects of differences in Cd conc. There is a statistically significant difference (P = 0.003). To isolate which group(s) differ from the others use a multiple comparison procedure.

The difference in the mean values among the different levels of Cd conc is not great enough to exclude the possibility that the difference is just due to random sampling variability after allowing for the effects of differences in Exp. There is not a statistically significant difference (P = 0.313).

Power of performed test with alpha = 0.0500: for Exp : 0.834
Power of performed test with alpha = 0.0500: for Cd conc : 0.0861

Least square means for Exp :

Group	Mean	SEM
272.000	3.135	0.147
273.000	2.799	0.122
278.000	2.475	0.127
280.000	2.497	0.122

Least square means for Cd conc :

Group	Mean	SEM
0.5	2.796	0.122
20	2.815	0.122
50	2.525	0.122
10	2.770	0.153

All Pairwise Multiple Comparison Procedures (Holm-Sidak method):
Overall significance level = 0.05

Comparisons for factor: **Exp**

Comparison	Diff of Means	t	P	P<0.050

272.000 vs. 278.000	0.660	3.402	0.008	Yes
272.000 vs. 280.000	0.638	3.336	0.008	Yes
273.000 vs. 278.000	0.324	1.843	0.255	No
272.000 vs. 273.000	0.336	1.757	0.233	No
273.000 vs. 280.000	0.302	1.748	0.165	No
280.000 vs. 278.000	0.0220	0.125	0.901	No

Comparisons for factor: **Cd conc**

Comparison	Diff of Means	t	P	P<0.050
20 vs. 50	0.290	1.678	0.466	No
0.5 vs. 50	0.271	1.569	0.480	No
10 vs. 50	0.245	1.252	0.622	No
20 vs. 10	0.0447	0.228	0.994	No
0.5 vs. 10	0.0258	0.132	0.989	No
20 vs. 0.5	0.0188	0.109	0.914	No

Elements in the plant tissues -Calcium - Roots

Two Way Analysis of Variance
General Linear Model (No Interactions)
Dependent Variable: Ca roots
Normality Test (Shapiro-Wilk) Passed (P = 0.196)
Equal Variance Test: Passed (P = 1.000)

Source of Variation	DF	SS	MS	F	P
Exp	3	536.585	178.862	6.662	0.002
Cd conc	9	860.971	95.663	3.563	0.005
Residual	26	698.007	26.846		
Total	38	2105.591	55.410		

The difference in the mean values among the different levels of Exp is greater than would be expected by chance after allowing for effects of differences in Cd conc. There is a statistically significant difference (P = 0.002). To isolate which group(s) differ from the others use a multiple comparison procedure.

The difference in the mean values among the different levels of Cd conc is greater than would be expected by chance after allowing for effects of differences in Exp. There is a statistically significant difference (P = 0.005). To isolate which group(s) differ from the others use a multiple comparison procedure.

Power of performed test with alpha = 0.0500: for Exp : 0.917
Power of performed test with alpha = 0.0500: for Cd conc : 0.842

Least square means for Exp :

Group	Mean	SEM
272.000	19.172	1.756
273.000	11.662	1.638
278.000	9.008	1.638
280.000	11.090	1.638

Least square means for Cd conc :

Group	Mean	SEM
0.5	7.137	2.591
20	8.010	2.591
50	7.643	2.591
140	10.185	2.591
270	13.283	2.591
550	10.884	2.591
1270	19.079	2.591
3100	17.584	2.591
27500	20.586	2.591
10	12.940	3.033

All Pairwise Multiple Comparison Procedures (Holm-Sidak method):
Overall significance level = 0.05

Comparisons for factor: **Exp**

Comparison	Diff of Means	t	P	P<0.050
272.000 vs. 278.000	10.164	4.232	0.002	Yes
272.000 vs. 280.000	8.083	3.366	0.012	Yes
272.000 vs. 273.000	7.510	3.127	0.017	Yes
273.000 vs. 278.000	2.654	1.145	0.599	No
280.000 vs. 278.000	2.081	0.898	0.612	No
273.000 vs. 280.000	0.572	0.247	0.807	No

Comparisons for factor: **Cd conc**

Comparison	Diff of Means	t	P	P<0.050
27500 vs. 0.5	13.449	3.671	0.048	Yes
27500 vs. 50	12.943	3.533	0.066	No
27500 vs. 20	12.576	3.432	0.083	No
1270 vs. 0.5	11.942	3.260	0.123	No
1270 vs. 50	11.436	3.121	0.165	No
1270 vs. 20	11.069	3.021	0.201	No
3100 vs. 0.5	10.447	2.851	0.281	No
27500 vs. 140	10.400	2.839	0.282	No
3100 vs. 50	9.941	2.713	0.352	No
27500 vs. 550	9.702	2.648	0.389	No
3100 vs. 20	9.574	2.613	0.405	No
1270 vs. 140	8.894	2.428	0.538	No
1270 vs. 550	8.195	2.237	0.682	No
3100 vs. 140	7.399	2.019	0.830	No
27500 vs. 270	7.303	1.993	0.837	No
27500 vs. 10	7.646	1.917	0.872	No
3100 vs. 550	6.700	1.829	0.908	No
270 vs. 0.5	6.146	1.677	0.956	No
1270 vs. 270	5.796	1.582	0.973	No
270 vs. 50	5.640	1.539	0.978	No
1270 vs. 10	6.139	1.539	0.974	No
10 vs. 0.5	5.803	1.455	0.984	No
270 vs. 20	5.273	1.439	0.983	No
10 vs. 50	5.297	1.328	0.992	No
10 vs. 20	4.930	1.236	0.996	No
3100 vs. 270	4.301	1.174	0.997	No
3100 vs. 10	4.644	1.164	0.996	No
550 vs. 0.5	3.747	1.023	0.999	No
550 vs. 50	3.241	0.885	1.000	No
270 vs. 140	3.098	0.845	1.000	No
140 vs. 0.5	3.048	0.832	1.000	No
27500 vs. 3100	3.002	0.819	1.000	No
550 vs. 20	2.874	0.784	0.999	No
140 vs. 50	2.542	0.694	1.000	No
10 vs. 140	2.755	0.691	0.999	No
270 vs. 550	2.399	0.655	0.999	No
140 vs. 20	2.175	0.594	0.999	No
10 vs. 550	2.056	0.516	0.999	No
27500 vs. 1270	1.506	0.411	1.000	No
1270 vs. 3100	1.495	0.408	0.999	No
20 vs. 0.5	0.873	0.238	1.000	No
550 vs. 140	0.699	0.191	0.999	No
50 vs. 0.5	0.506	0.138	0.999	No
20 vs. 50	0.367	0.100	0.994	No
270 vs. 10	0.343	0.0859	0.932	No

Elements in the plant tissues -Calcium - Stems

Two Way Analysis of Variance
General Linear Model (No Interactions)
Dependent Variable: Ca stems
Normality Test (Shapiro-Wilk) Failed (P < 0.050)
Equal Variance Test: Passed (P = 0.102)

Source of Variation	DF	SS	MS	F	P
Exp	3	1109.809	369.936	6.650	<0.001
Cd conc	9	10969.971	1218.886	21.912	<0.001
Residual	127	7064.647	55.627		
Total	139	19038.647	136.969		

The difference in the mean values among the different levels of Exp is greater than would be expected by chance after allowing for effects of differences in Cd conc. There is a statistically significant difference (P = <0.001). To isolate which group(s) differ from the others use a multiple comparison procedure.

The difference in the mean values among the different levels of Cd conc is greater than would be expected by chance after allowing for effects of differences in Exp. There is a statistically significant difference (P = <0.001). To isolate which group(s) differ from the others use a multiple comparison procedure.

Power of performed test with alpha = 0.0500: for Exp : 0.951
Power of performed test with alpha = 0.0500: for Cd conc : 1.000

Least square means for Exp :

Group	Mean	SEM
272.000	23.119	1.286
273.000	20.930	1.179
278.000	20.058	1.473
280.000	15.568	1.212

Least square means for Cd conc :

Group	Mean	SEM
0.5	29.543	1.865
20	28.210	1.865
50	28.036	1.865
140	20.780	2.001
270	16.853	2.192
550	14.712	2.001
1270	12.498	1.995
3100	8.415	2.172
27500	6.699	1.865
10	33.441	2.389

All Pairwise Multiple Comparison Procedures (Holm-Sidak method):
Overall significance level = 0.05

Comparisons for factor: **Exp**

Comparison	Diff of Means	t	P	P<0.050
272.000 vs. 280.000	7.551	4.273	<0.001	Yes
273.000 vs. 280.000	5.362	3.171	0.009	Yes
278.000 vs. 280.000	4.491	2.355	0.078	No
272.000 vs. 278.000	3.061	1.565	0.318	No
272.000 vs. 273.000	2.189	1.255	0.379	No
273.000 vs. 278.000	0.871	0.462	0.645	No

Comparisons for factor: **Cd conc**

Comparison	Diff of Means	t	P	P<0.050
10 vs. 27500	26.742	8.825	<0.001	Yes

0.5 vs. 27500	22.844	8.663	<0.001	Yes
20 vs. 27500	21.511	8.158	<0.001	Yes
50 vs. 27500	21.337	8.092	<0.001	Yes
10 vs. 3100	25.025	7.751	<0.001	Yes
0.5 vs. 3100	21.128	7.381	<0.001	Yes
20 vs. 3100	19.795	6.915	<0.001	Yes
50 vs. 3100	19.621	6.854	<0.001	Yes
10 vs. 1270	20.943	6.728	<0.001	Yes
0.5 vs. 1270	17.045	6.241	<0.001	Yes
10 vs. 550	18.728	6.010	<0.001	Yes
20 vs. 1270	15.712	5.753	<0.001	Yes
50 vs. 1270	15.538	5.690	<0.001	Yes
0.5 vs. 550	14.831	5.422	<0.001	Yes
140 vs. 27500	14.081	5.148	<0.001	Yes
10 vs. 270	16.588	5.117	<0.001	Yes
20 vs. 550	13.498	4.935	<0.001	Yes
50 vs. 550	13.324	4.872	<0.001	Yes
0.5 vs. 270	12.690	4.410	<0.001	Yes
140 vs. 3100	12.365	4.187	0.001	Yes
10 vs. 140	12.660	4.063	0.002	Yes
20 vs. 270	11.357	3.947	0.003	Yes
50 vs. 270	11.183	3.887	0.004	Yes
270 vs. 27500	10.154	3.529	0.013	Yes
0.5 vs. 140	8.763	3.204	0.035	Yes
140 vs. 1270	8.282	2.931	0.077	No
550 vs. 27500	8.013	2.930	0.074	No
270 vs. 3100	8.438	2.735	0.121	No
20 vs. 140	7.430	2.716	0.120	No
50 vs. 140	7.256	2.653	0.135	No
140 vs. 550	6.068	2.144	0.404	No
550 vs. 3100	6.297	2.132	0.392	No
1270 vs. 27500	5.799	2.123	0.376	No
10 vs. 50	5.404	1.783	0.617	No
10 vs. 20	5.231	1.726	0.631	No
270 vs. 1270	4.355	1.469	0.789	No
1270 vs. 3100	4.083	1.384	0.810	No
140 vs. 270	3.927	1.323	0.811	No
10 vs. 0.5	3.897	1.286	0.792	No
550 vs. 1270	2.214	0.784	0.967	No
270 vs. 550	2.141	0.721	0.959	No
3100 vs. 27500	1.716	0.600	0.959	No
0.5 vs. 50	1.507	0.571	0.920	No
0.5 vs. 20	1.333	0.506	0.851	No
20 vs. 50	0.174	0.0658	0.948	No

Elements in the plant tissues -Calcium - w5 leaves

Two Way Analysis of Variance
General Linear Model (No Interactions)
Dependent Variable: Ca w5
Normality Test (Shapiro-Wilk) Passed (P = 0.998)
Equal Variance Test: Failed (P < 0.050)

**Source of Variation**	**DF**	**SS**	**MS**	**F**	**P**
Exp	3	156.561	52.187	3.132	0.032
Cd conc	9	2611.673	290.186	17.416	<0.001
Residual	62	1033.033	16.662		
Total	74	3718.979	50.256		

The difference in the mean values among the different levels of Exp is greater than would be expected by chance after allowing for effects of differences in Cd conc. There is a statistically significant difference (P = 0.032). To isolate which group(s) differ from the others use a multiple comparison procedure.

The difference in the mean values among the different levels of Cd conc is greater than would be expected by chance after allowing for effects of differences in Exp. There is a statistically significant difference (P = <0.001). To isolate which group(s) differ from the others use a multiple comparison procedure.

Power of performed test with alpha = 0.0500: for Exp : 0.511
Power of performed test with alpha = 0.0500: for Cd conc : 1.000

Least square means for Exp :

**Group**	**Mean**	**SEM**
272.000	18.321	0.978
273.000	19.462	0.913
278.000	18.377	0.941
280.000	15.493	0.981

Least square means for Cd conc :

**Group**	**Mean**	**SEM**
0.5	21.686	1.443
20	20.594	1.443
50	23.148	1.443
140	23.772	1.443
270	23.074	1.443
550	17.238	1.443
1270	12.877	1.443
3100	9.565	1.443
27500	4.567	1.854
10	22.613	1.690

All Pairwise Multiple Comparison Procedures (Holm-Sidak method):
Overall significance level = 0.05

Comparisons for factor: **Exp**

**Comparison**	**Diff of Means**	**t**	**P**	**P<0.050**
273.000 vs. 280.000	3.969	2.962	0.026	Yes
278.000 vs. 280.000	2.884	2.122	0.175	No
272.000 vs. 280.000	2.828	2.041	0.170	No
273.000 vs. 272.000	1.141	0.853	0.781	No
273.000 vs. 278.000	1.085	0.828	0.653	No
278.000 vs. 272.000	0.0560	0.0413	0.967	No

Comparisons for factor: **Cd conc**

**Comparison**	**Diff of Means**	**t**	**P**	**P<0.050**
140 vs. 27500	19.205	8.175	<0.001	Yes
50 vs. 27500	18.581	7.910	<0.001	Yes
270 vs. 27500	18.507	7.878	<0.001	Yes
0.5 vs. 27500	17.119	7.287	<0.001	Yes
10 vs. 27500	18.046	7.194	<0.001	Yes
140 vs. 3100	14.207	6.961	<0.001	Yes
20 vs. 27500	16.027	6.823	<0.001	Yes
50 vs. 3100	13.583	6.655	<0.001	Yes
270 vs. 3100	13.509	6.619	<0.001	Yes
0.5 vs. 3100	12.121	5.939	<0.001	Yes
10 vs. 3100	13.048	5.871	<0.001	Yes
20 vs. 3100	11.029	5.404	<0.001	Yes
550 vs. 27500	12.671	5.394	<0.001	Yes
140 vs. 1270	10.895	5.338	<0.001	Yes
50 vs. 1270	10.271	5.032	<0.001	Yes
270 vs. 1270	10.197	4.996	<0.001	Yes

10 vs. 1270	9.736	4.381	0.001	Yes
0.5 vs. 1270	8.809	4.316	0.002	Yes
20 vs. 1270	7.717	3.781	0.009	Yes
550 vs. 3100	7.673	3.759	0.010	Yes
1270 vs. 27500	8.310	3.537	0.019	Yes
140 vs. 550	6.534	3.202	0.050	No
50 vs. 550	5.910	2.896	0.113	No
270 vs. 550	5.837	2.860	0.120	No
10 vs. 550	5.375	2.419	0.325	No
0.5 vs. 550	4.448	2.180	0.490	No
550 vs. 1270	4.361	2.137	0.507	No
3100 vs. 27500	4.998	2.128	0.496	No
20 vs. 550	3.357	1.645	0.849	No
1270 vs. 3100	3.312	1.623	0.844	No
140 vs. 20	3.178	1.557	0.864	No
50 vs. 20	2.554	1.251	0.967	No
270 vs. 20	2.480	1.215	0.966	No
140 vs. 0.5	2.086	1.022	0.988	No
10 vs. 20	2.019	0.908	0.993	No
50 vs. 0.5	1.462	0.716	0.998	No
270 vs. 0.5	1.388	0.680	0.998	No
0.5 vs. 20	1.092	0.535	0.999	No
140 vs. 10	1.159	0.522	0.998	No
10 vs. 0.5	0.927	0.417	0.999	No
140 vs. 270	0.698	0.342	0.999	No
140 vs. 50	0.624	0.306	0.997	No
50 vs. 10	0.535	0.241	0.993	No
270 vs. 10	0.461	0.208	0.973	No
50 vs. 270	0.0736	0.0361	0.971	No

Elements in the plant tissues -Calcium - Harvested leaves

Two Way Analysis of Variance
General Linear Model (No Interactions)
Dependent Variable: Ca
Normality Test (Shapiro-Wilk) Failed (P < 0.050)
Equal Variance Test: Passed (P = 0.055)

**Source of Variation**	**DF**	**SS**	**MS**	**F**	**P**
Exp	3	9512.083	3170.694	0.517	0.671
Cd conc	9	210120.013	23346.668	3.806	<0.001
Residual	117	717705.687	6134.237		
Total	129	939132.915	7280.100		

The difference in the mean values among the different levels of Exp is not great enough to exclude the possibility that the difference is just due to random sampling variability after allowing for the effects of differences in Cd conc. There is not a statistically significant difference (P = 0.671).

The difference in the mean values among the different levels of Cd conc is greater than would be expected by chance after allowing for effects of differences in Exp. There is a statistically significant difference (P = <0.001). To isolate which group(s) differ from the others use a multiple comparison procedure.

Power of performed test with alpha = 0.0500: for Exp : 0.0500
Power of performed test with alpha = 0.0500: for Cd conc : 0.954

Least square means for Exp :

**Group**	**Mean**	**SEM**
272.000	62.890	14.333
273.000	46.545	12.729

278.000	37.745	16.229
280.000	54.953	13.128

Least square means for Cd conc :

**Group**	**Mean**	**SEM**
0.5	75.480	19.580
20	109.417	19.580
50	117.802	24.064
140	24.234	21.740
270	25.056	23.646
550	24.795	20.956
1270	17.139	21.804
3100	11.162	25.275
27500	10.945	20.240
10	89.303	23.981

All Pairwise Multiple Comparison Procedures (Holm-Sidak method):
Overall significance level = 0.05

Comparisons for factor: **Exp**

**Comparison**	**Diff of Means**	**t**	**P**	**P<0.050**
272.000 vs. 278.000	25.145	1.161	0.819	No
272.000 vs. 273.000	16.345	0.853	0.919	No
280.000 vs. 278.000	17.208	0.824	0.880	No
280.000 vs. 273.000	8.408	0.460	0.956	No
273.000 vs. 278.000	8.800	0.427	0.891	No
272.000 vs. 280.000	7.937	0.408	0.684	No

Comparisons for factor: **Cd conc**

**Comparison**	**Diff of Means**	**t**	**P**	**P<0.050**
20 vs. 27500	98.471	3.497	0.030	Yes
50 vs. 27500	106.857	3.398	0.040	Yes
20 vs. 1270	92.277	3.149	0.086	No
50 vs. 1270	100.663	3.100	0.097	No
20 vs. 3100	98.254	3.073	0.103	No
50 vs. 3100	106.640	3.056	0.105	No
20 vs. 550	84.622	2.951	0.139	No
50 vs. 550	93.007	2.915	0.150	No
20 vs. 140	85.182	2.911	0.148	No
50 vs. 140	93.568	2.885	0.155	No
50 vs. 270	92.746	2.749	0.216	No
20 vs. 270	84.361	2.748	0.211	No
10 vs. 27500	78.358	2.497	0.370	No
0.5 vs. 27500	64.535	2.292	0.536	No
10 vs. 3100	78.140	2.243	0.569	No
10 vs. 1270	72.164	2.226	0.572	No
10 vs. 550	64.508	2.026	0.738	No
0.5 vs. 3100	64.317	2.012	0.737	No
10 vs. 140	65.069	2.010	0.725	No
0.5 vs. 1270	58.341	1.991	0.728	No
10 vs. 270	64.247	1.908	0.781	No
0.5 vs. 550	50.685	1.767	0.864	No
0.5 vs. 140	51.246	1.752	0.862	No
0.5 vs. 270	50.424	1.642	0.909	No
50 vs. 0.5	42.322	1.364	0.982	No
20 vs. 0.5	33.937	1.226	0.994	No
50 vs. 10	28.499	0.839	1.000	No
20 vs. 10	20.114	0.650	1.000	No
550 vs. 27500	13.850	0.475	1.000	No
270 vs. 27500	14.111	0.453	1.000	No
140 vs. 27500	13.289	0.447	1.000	No

10 vs. 0.5	13.823	0.446	1.000	No
550 vs. 3100	13.632	0.415	1.000	No
270 vs. 3100	13.893	0.401	1.000	No
140 vs. 3100	13.072	0.392	1.000	No
50 vs. 20	8.385	0.270	1.000	No
550 vs. 1270	7.656	0.253	1.000	No
270 vs. 1270	7.917	0.246	1.000	No
140 vs. 1270	7.095	0.230	1.000	No
1270 vs. 27500	6.194	0.208	1.000	No
1270 vs. 3100	5.977	0.179	1.000	No
270 vs. 140	0.822	0.0256	1.000	No
550 vs. 140	0.561	0.0186	1.000	No
270 vs. 550	0.261	0.00825	1.000	No
3100 vs. 27500	0.217	0.00671	0.995	No

Elements in the plant tissues -Calcium - w10 leaves

Two Way Analysis of Variance
General Linear Model (No Interactions)
Dependent Variable: Ca w10
Normality Test (Shapiro-Wilk) Failed (P < 0.050)
Equal Variance Test: Failed (P < 0.050)

**Source of Variation**	**DF**	**SS**	**MS**	**F**	**P**
Exp	3	3104.032	1034.677	1.018	0.394
Cd conc	8	35301.783	4412.723	4.341	<0.001
Residual	43	43711.632	1016.550		
Total	54	82544.139	1528.595		

The difference in the mean values among the different levels of Exp is not great enough to exclude the possibility that the difference is just due to random sampling variability after allowing for the effects of differences in Cd conc. There is not a statistically significant difference (P = 0.394).

The difference in the mean values among the different levels of Cd conc is greater than would be expected by chance after allowing for effects of differences in Exp. There is a statistically significant difference (P = <0.001). To isolate which group(s) differ from the others use a multiple comparison procedure.

Power of performed test with alpha = 0.0500: for Exp : 0.0521
Power of performed test with alpha = 0.0500: for Cd conc : 0.951

Least square means for Exp :

**Group**	**Mean**	**SEM**
272.000	62.723	12.451
273.000	53.560	8.381
278.000	40.296	7.828
280.000	54.041	8.361

Least square means for Cd conc :

**Group**	**Mean**	**SEM**
0.5	62.477	12.132
20	76.776	12.132
50	88.417	12.132
140	56.533	12.132
270	29.523	12.132
550	30.657	13.107
1270	18.081	13.107
10	86.462	13.420
3100	24.970	23.025

All Pairwise Multiple Comparison Procedures (Holm-Sidak method):
Overall significance level = 0.05

Comparisons for factor: **Exp**

Comparison	Diff of Means	t	P	P<0.050
272.000 vs. 278.000	22.426	1.525	0.580	No
280.000 vs. 278.000	13.744	1.200	0.741	No
273.000 vs. 278.000	13.264	1.157	0.690	No
272.000 vs. 273.000	9.163	0.610	0.906	No
272.000 vs. 280.000	8.682	0.579	0.811	No
280.000 vs. 273.000	0.481	0.0406	0.968	No

Comparisons for factor: **Cd conc**

Comparison	Diff of Means	t	P	P<0.050
50 vs. 1270	70.335	3.938	0.011	Yes
10 vs. 1270	68.380	3.645	0.025	Yes
50 vs. 270	58.894	3.433	0.044	Yes
20 vs. 1270	58.695	3.286	0.065	No
50 vs. 550	57.760	3.234	0.072	No
10 vs. 270	56.939	3.147	0.089	No
10 vs. 550	55.805	2.975	0.134	No
20 vs. 270	47.253	2.754	0.221	No
20 vs. 550	46.120	2.582	0.313	No
0.5 vs. 1270	44.395	2.486	0.369	No
50 vs. 3100	63.447	2.438	0.392	No
10 vs. 3100	61.492	2.307	0.481	No
140 vs. 1270	38.451	2.153	0.595	No
20 vs. 3100	51.806	1.991	0.714	No
0.5 vs. 270	32.954	1.921	0.752	No
50 vs. 140	31.884	1.858	0.782	No
0.5 vs. 550	31.820	1.782	0.819	No
10 vs. 140	29.929	1.654	0.879	No
140 vs. 270	27.010	1.574	0.905	No
50 vs. 0.5	25.940	1.512	0.920	No
140 vs. 550	25.876	1.449	0.932	No
0.5 vs. 3100	37.507	1.441	0.923	No
10 vs. 0.5	23.985	1.326	0.949	No
140 vs. 3100	31.563	1.213	0.968	No
20 vs. 140	20.244	1.180	0.965	No
20 vs. 0.5	14.300	0.833	0.997	No
50 vs. 20	11.640	0.678	0.999	No
550 vs. 1270	12.575	0.678	0.998	No
270 vs. 1270	11.442	0.641	0.997	No
10 vs. 20	9.685	0.535	0.998	No
0.5 vs. 140	5.944	0.346	1.000	No
3100 vs. 1270	6.888	0.260	1.000	No
550 vs. 3100	5.687	0.215	0.999	No
270 vs. 3100	4.553	0.175	0.997	No
50 vs. 10	1.955	0.108	0.993	No
550 vs. 270	1.133	0.0635	0.950	No

Elements in the plant tissues - Potassium - harvested leaves

Two Way Analysis of Variance
General Linear Model (No Interactions)
Dependent Variable: K harvest
Normality Test (Shapiro-Wilk) Failed (P < 0.050)
Equal Variance Test: Failed (P < 0.050)

Source of Variation	DF	SS	MS	F	P
Exp	3	10015.122	3338.374	0.770	0.513
Cd conc	9	590826.541	65647.393	15.136	<0.001
Residual	110	477092.547	4337.205		
Total	122	1069770.088	8768.607		

The difference in the mean values among the different levels of Exp is not great enough to exclude the possibility that the difference is just due to random sampling variability after allowing for the effects of differences in Cd conc. There is not a statistically significant difference (P = 0.513).

The difference in the mean values among the different levels of Cd conc is greater than would be expected by chance after allowing for effects of differences in Exp. There is a statistically significant difference (P = <0.001). To isolate which group(s) differ from the others use a multiple comparison procedure.

Power of performed test with alpha = 0.0500: for Exp : 0.0500
Power of performed test with alpha = 0.0500: for Cd conc : 1.000

Least square means for Exp :

Group	Mean	SEM
272.000	126.585	12.051
273.000	127.872	10.704
278.000	149.593	15.935
280.000	143.725	11.198

Least square means for Cd conc :

Group	Mean	SEM
0.5	70.399	17.026
20	78.514	19.498
50	79.217	20.347
140	170.667	20.058
270	135.828	19.892
550	249.339	18.281
1270	224.519	18.351
3100	215.313	21.362
27500	92.342	17.020
10	53.300	19.349

All Pairwise Multiple Comparison Procedures (Holm-Sidak method):
Overall significance level = 0.05

Comparisons for factor: **Exp**

Comparison	Diff of Means	t	P	P<0.050
278.000 vs. 272.000	23.008	1.152	0.825	No
278.000 vs. 273.000	21.722	1.132	0.779	No
280.000 vs. 272.000	17.139	1.042	0.760	No
280.000 vs. 273.000	15.853	1.023	0.669	No
278.000 vs. 280.000	5.869	0.301	0.944	No
273.000 vs. 272.000	1.287	0.0798	0.937	No

Comparisons for factor: **Cd conc**

Comparison	Diff of Means	t	P	P<0.050
550 vs. 10	196.039	7.365	<0.001	Yes
550 vs. 0.5	178.940	7.163	<0.001	Yes
1270 vs. 10	171.219	6.421	<0.001	Yes
550 vs. 20	170.825	6.391	<0.001	Yes
550 vs. 27500	156.997	6.286	<0.001	Yes
550 vs. 50	170.122	6.219	<0.001	Yes
1270 vs. 0.5	154.120	6.157	<0.001	Yes
3100 vs. 10	162.013	5.621	<0.001	Yes
1270 vs. 20	146.005	5.453	<0.001	Yes

3100 vs. 0.5	144.914	5.305	<0.001	Yes
1270 vs. 50	145.302	5.303	<0.001	Yes
1270 vs. 27500	132.176	5.281	<0.001	Yes
3100 vs. 20	136.799	4.730	<0.001	Yes
3100 vs. 50	136.096	4.613	<0.001	Yes
3100 vs. 27500	122.971	4.502	<0.001	Yes
140 vs. 10	117.368	4.211	0.002	Yes
550 vs. 270	113.511	4.202	0.002	Yes
140 vs. 0.5	100.269	3.811	0.006	Yes
140 vs. 20	92.153	3.294	0.035	Yes
1270 vs. 270	88.691	3.277	0.036	Yes
140 vs. 50	91.450	3.201	0.044	Yes
140 vs. 27500	78.325	2.977	0.082	No
270 vs. 10	82.528	2.974	0.080	No
550 vs. 140	78.672	2.899	0.095	No
3100 vs. 270	79.485	2.723	0.147	No
270 vs. 0.5	65.430	2.499	0.245	No
270 vs. 20	57.314	2.058	0.557	No
270 vs. 50	56.611	1.989	0.596	No
1270 vs. 140	53.851	1.981	0.583	No
270 vs. 27500	43.486	1.661	0.813	No
3100 vs. 140	44.646	1.524	0.877	No
27500 vs. 10	39.042	1.515	0.864	No
140 vs. 270	34.839	1.233	0.961	No
550 vs. 3100	34.026	1.210	0.956	No
550 vs. 1270	24.820	0.958	0.990	No
50 vs. 10	25.917	0.923	0.988	No
20 vs. 10	25.214	0.918	0.982	No
27500 vs. 0.5	21.944	0.912	0.973	No
0.5 vs. 10	17.099	0.663	0.993	No
27500 vs. 20	13.828	0.534	0.996	No
27500 vs. 50	13.125	0.495	0.992	No
50 vs. 0.5	8.819	0.332	0.995	No
1270 vs. 3100	9.206	0.327	0.983	No
20 vs. 0.5	8.115	0.314	0.940	No
50 vs. 20	0.703	0.0250	0.980	No

Elements in the plant tissues - Potassium - w10 leaves

Two Way Analysis of Variance
General Linear Model (No Interactions)
Dependent Variable: K w10
Normality Test (Shapiro-Wilk) Failed (P < 0.050)
Equal Variance Test: Failed (P < 0.050)

**Source of Variation**	**DF**	**SS**	**MS**	**F**	**P**
Exp	3	184443.542	61481.181	1.961	0.134
Cd conc	8	596383.143	74547.893	2.378	0.032
Residual	43	1348010.205	31349.075		
Total	54	2127029.153	39389.429		

The difference in the mean values among the different levels of Exp is not great enough to exclude the possibility that the difference is just due to random sampling variability after allowing for the effects of differences in Cd conc. There is not a statistically significant difference (P = 0.134).

The difference in the mean values among the different levels of Cd conc is greater than would be expected by chance after allowing for effects of differences in Exp. There is a statistically significant difference (P = 0.032). To isolate which group(s) differ from the others use a multiple comparison procedure.

Power of performed test with alpha = 0.0500: for Exp : 0.237
Power of performed test with alpha = 0.0500: for Cd conc : 0.546

Least square means for Exp :

**Group**	**Mean**	**SEM**
272.000	124.441	69.143
273.000	221.036	46.539
278.000	71.375	43.472
280.000	143.169	46.429

Least square means for Cd conc :

**Group**	**Mean**	**SEM**
0.5	45.756	67.372
20	40.491	67.372
50	53.724	67.372
140	229.149	67.372
270	189.789	67.372
550	154.965	72.784
1270	351.142	72.784
10	28.158	74.527
3100	166.870	127.865

All Pairwise Multiple Comparison Procedures (Holm-Sidak method):
Overall significance level = 0.05

Comparisons for factor: **Exp**

**Comparison**	**Diff of Means**	**t**	**P**	**P<0.050**
273.000 vs. 278.000	149.660	2.350	0.133	No
273.000 vs. 280.000	77.867	1.184	0.751	No
273.000 vs. 272.000	96.595	1.159	0.688	No
280.000 vs. 278.000	71.793	1.129	0.603	No
272.000 vs. 278.000	53.066	0.650	0.769	No
280.000 vs. 272.000	18.728	0.225	0.823	No

Comparisons for factor: **Cd conc**

**Comparison**	**Diff of Means**	**t**	**P**	**P<0.050**
1270 vs. 20	310.650	3.132	0.106	No
1270 vs. 10	322.983	3.100	0.112	No
1270 vs. 0.5	305.385	3.079	0.116	No
1270 vs. 50	297.418	2.999	0.138	No
140 vs. 10	200.991	2.001	0.818	No
140 vs. 20	188.658	1.980	0.822	No
140 vs. 0.5	183.393	1.925	0.848	No
1270 vs. 550	196.177	1.906	0.850	No
140 vs. 50	175.426	1.841	0.878	No
1270 vs. 270	161.353	1.627	0.958	No
270 vs. 10	161.630	1.609	0.958	No
270 vs. 20	149.297	1.567	0.964	No
270 vs. 0.5	144.032	1.512	0.972	No
270 vs. 50	136.065	1.428	0.982	No
1270 vs. 3100	184.272	1.252	0.995	No
1270 vs. 140	121.993	1.230	0.995	No
550 vs. 10	126.807	1.217	0.995	No
550 vs. 20	114.474	1.154	0.996	No
550 vs. 0.5	109.209	1.101	0.997	No
550 vs. 50	101.241	1.021	0.998	No
3100 vs. 10	138.712	0.937	0.999	No
3100 vs. 20	126.379	0.874	0.999	No
3100 vs. 0.5	121.114	0.838	0.999	No
3100 vs. 50	113.147	0.783	0.999	No

140 vs. 550	74.184	0.748	0.999	No
140 vs. 3100	62.279	0.431	1.000	No
140 vs. 270	39.361	0.413	1.000	No
270 vs. 550	34.824	0.351	1.000	No
50 vs. 10	25.565	0.254	1.000	No
0.5 vs. 10	17.598	0.175	1.000	No
270 vs. 3100	22.918	0.159	1.000	No
50 vs. 20	13.232	0.139	1.000	No
20 vs. 10	12.333	0.123	1.000	No
50 vs. 0.5	7.967	0.0836	1.000	No
3100 vs. 550	11.905	0.0809	0.996	No
0.5 vs. 20	5.265	0.0553	0.956	No

Elements in the plant tissues - Potassium - w5 leaves

Two Way Analysis of Variance
General Linear Model (No Interactions)
Dependent Variable: K w5
Normality Test (Shapiro-Wilk) Passed (P = 0.221)
Equal Variance Test: Failed (P < 0.050)

**Source of Variation**	**DF**	**SS**	**MS**	**F**	**P**
Exp	3	1366.040	455.347	13.524	<0.001
Cd conc	9	3155.029	350.559	10.412	<0.001
Residual	62	2087.524	33.670		
Total	74	7001.301	94.612		

The difference in the mean values among the different levels of Exp is greater than would be expected by chance after allowing for effects of differences in Cd conc. There is a statistically significant difference (P = <0.001). To isolate which group(s) differ from the others use a multiple comparison procedure.

The difference in the mean values among the different levels of Cd conc is greater than would be expected by chance after allowing for effects of differences in Exp. There is a statistically significant difference (P = <0.001). To isolate which group(s) differ from the others use a multiple comparison procedure.

Power of performed test with alpha = 0.0500: for Exp : 1.000
Power of performed test with alpha = 0.0500: for Cd conc : 1.000

Least square means for Exp :

**Group**	**Mean**	**SEM**
272.000	24.856	1.391
273.000	30.283	1.297
278.000	29.168	1.337
280.000	37.211	1.395

Least square means for Cd conc :

**Group**	**Mean**	**SEM**
0.5	29.182	2.052
20	29.103	2.052
50	30.438	2.052
140	38.273	2.052
270	32.483	2.052
550	36.162	2.052
1270	41.089	2.052
3100	25.240	2.052
27500	14.908	2.635
10	26.919	2.402

All Pairwise Multiple Comparison Procedures (Holm-Sidak method):
Overall significance level = 0.05

Comparisons for factor: **Exp**

Comparison	Diff of Means	t	P	P<0.050
280.000 vs. 272.000	12.355	6.274	<0.001	Yes
280.000 vs. 278.000	8.044	4.163	<0.001	Yes
280.000 vs. 273.000	6.928	3.637	0.002	Yes
273.000 vs. 272.000	5.427	2.854	0.018	Yes
278.000 vs. 272.000	4.312	2.235	0.057	No
273.000 vs. 278.000	1.115	0.599	0.552	No

Comparisons for factor: **Cd conc**

Comparison	Diff of Means	t	P	P<0.050
1270 vs. 27500	26.181	7.840	<0.001	Yes
140 vs. 27500	23.365	6.997	<0.001	Yes
550 vs. 27500	21.254	6.365	<0.001	Yes
1270 vs. 3100	15.849	5.463	<0.001	Yes
270 vs. 27500	17.576	5.263	<0.001	Yes
50 vs. 27500	15.530	4.650	<0.001	Yes
140 vs. 3100	13.033	4.492	0.001	Yes
1270 vs. 10	14.170	4.485	0.001	Yes
0.5 vs. 27500	14.275	4.275	0.002	Yes
20 vs. 27500	14.195	4.251	0.003	Yes
1270 vs. 20	11.986	4.131	0.004	Yes
1270 vs. 0.5	11.907	4.104	0.004	Yes
550 vs. 3100	10.922	3.765	0.012	Yes
1270 vs. 50	10.651	3.671	0.016	Yes
140 vs. 10	11.354	3.594	0.020	Yes
10 vs. 27500	12.012	3.369	0.038	Yes
140 vs. 20	9.170	3.161	0.068	No
140 vs. 0.5	9.091	3.133	0.071	No
3100 vs. 27500	10.332	3.094	0.077	No
1270 vs. 270	8.606	2.966	0.105	No
550 vs. 10	9.242	2.926	0.113	No
140 vs. 50	7.835	2.701	0.193	No
270 vs. 3100	7.243	2.497	0.297	No
550 vs. 20	7.059	2.433	0.327	No
550 vs. 0.5	6.980	2.406	0.334	No
140 vs. 270	5.790	1.996	0.644	No
550 vs. 50	5.724	1.973	0.644	No
50 vs. 3100	5.198	1.792	0.769	No
270 vs. 10	5.564	1.761	0.771	No
1270 vs. 550	4.927	1.698	0.796	No
0.5 vs. 3100	3.942	1.359	0.948	No
20 vs. 3100	3.863	1.331	0.946	No
550 vs. 270	3.678	1.268	0.953	No
270 vs. 20	3.380	1.165	0.968	No
270 vs. 0.5	3.301	1.138	0.963	No
50 vs. 10	3.518	1.114	0.957	No
1270 vs. 140	2.816	0.971	0.975	No
140 vs. 550	2.111	0.728	0.994	No
0.5 vs. 10	2.263	0.716	0.989	No
270 vs. 50	2.046	0.705	0.981	No
20 vs. 10	2.183	0.691	0.966	No
10 vs. 3100	1.680	0.532	0.974	No
50 vs. 20	1.335	0.460	0.956	No
50 vs. 0.5	1.255	0.433	0.889	No
0.5 vs. 20	0.0794	0.0274	0.978	No

Elements in the plant tissues - Potassium - Stems

Two Way Analysis of Variance
General Linear Model (No Interactions)
Dependent Variable: K stems
Normality Test (Shapiro-Wilk) Passed (P = 0.095)
Equal Variance Test: Passed (P = 0.065)

Source of Variation	DF	SS	MS	F	P
Exp	3	869.993	289.998	3.403	0.020
Cd conc	9	28612.701	3179.189	37.308	<0.001
Residual	127	10822.408	85.216		
Total	139	40771.920	293.323		

The difference in the mean values among the different levels of Exp is greater than would be expected by chance after allowing for effects of differences in Cd conc. There is a statistically significant difference (P = 0.020). To isolate which group(s) differ from the others use a multiple comparison procedure.

The difference in the mean values among the different levels of Cd conc is greater than would be expected by chance after allowing for effects of differences in Exp. There is a statistically significant difference (P = <0.001). To isolate which group(s) differ from the others use a multiple comparison procedure.

Power of performed test with alpha = 0.0500: for Exp : 0.584
Power of performed test with alpha = 0.0500: for Cd conc : 1.000

Least square means for Exp :

Group	Mean	SEM
272.000	23.455	1.592
273.000	26.781	1.460
278.000	26.723	1.823
280.000	30.395	1.500

Least square means for Cd conc :

Group	Mean	SEM
0.5	11.815	2.308
20	8.548	2.308
50	9.137	2.308
140	38.257	2.477
270	40.785	2.713
550	42.003	2.477
1270	41.090	2.470
3100	37.662	2.688
27500	31.031	2.308
10	8.059	2.957

All Pairwise Multiple Comparison Procedures (Holm-Sidak method):
Overall significance level = 0.05

Comparisons for factor: **Exp**

Comparison	Diff of Means	t	P	P<0.050
280.000 vs. 272.000	6.940	3.173	0.011	Yes
280.000 vs. 273.000	3.615	1.727	0.364	No
280.000 vs. 278.000	3.672	1.556	0.407	No
273.000 vs. 272.000	3.326	1.540	0.333	No
278.000 vs. 272.000	3.268	1.351	0.326	No
273.000 vs. 278.000	0.0575	0.0246	0.980	No

Comparisons for factor: **Cd conc**

Comparison	Diff of Means	t	P	P<0.050
550 vs. 20	33.456	9.883	<0.001	Yes
550 vs. 50	32.867	9.709	<0.001	Yes
1270 vs. 20	32.542	9.627	<0.001	Yes
1270 vs. 50	31.953	9.453	<0.001	Yes
270 vs. 20	32.237	9.052	<0.001	Yes
550 vs. 0.5	30.189	8.918	<0.001	Yes
270 vs. 50	31.648	8.886	<0.001	Yes
550 vs. 10	33.945	8.801	<0.001	Yes
140 vs. 20	29.709	8.776	<0.001	Yes
1270 vs. 0.5	29.275	8.661	<0.001	Yes
140 vs. 50	29.120	8.602	<0.001	Yes
1270 vs. 10	33.031	8.574	<0.001	Yes
3100 vs. 20	29.114	8.218	<0.001	Yes
270 vs. 10	32.726	8.156	<0.001	Yes
270 vs. 0.5	28.970	8.134	<0.001	Yes
3100 vs. 50	28.526	8.051	<0.001	Yes
140 vs. 10	30.198	7.830	<0.001	Yes
140 vs. 0.5	26.442	7.811	<0.001	Yes
3100 vs. 10	29.603	7.408	<0.001	Yes
3100 vs. 0.5	25.847	7.295	<0.001	Yes
27500 vs. 20	22.483	6.889	<0.001	Yes
27500 vs. 50	21.894	6.708	<0.001	Yes
27500 vs. 10	22.972	6.125	<0.001	Yes
27500 vs. 0.5	19.216	5.888	<0.001	Yes
550 vs. 27500	10.972	3.241	0.031	Yes
1270 vs. 27500	10.059	2.976	0.068	No
270 vs. 27500	9.754	2.739	0.126	No
140 vs. 27500	7.226	2.134	0.471	No
3100 vs. 27500	6.631	1.872	0.673	No
550 vs. 3100	4.341	1.188	0.987	No
550 vs. 140	3.747	1.070	0.994	No
0.5 vs. 10	3.756	1.001	0.995	No
0.5 vs. 20	3.267	1.001	0.993	No
1270 vs. 3100	3.428	0.939	0.994	No
0.5 vs. 50	2.678	0.821	0.997	No
270 vs. 3100	3.123	0.818	0.995	No
1270 vs. 140	2.833	0.810	0.993	No
270 vs. 140	2.528	0.688	0.996	No
550 vs. 270	1.219	0.332	1.000	No
50 vs. 10	1.078	0.287	1.000	No
550 vs. 1270	0.913	0.261	1.000	No
50 vs. 20	0.589	0.180	1.000	No
140 vs. 3100	0.595	0.163	0.998	No
20 vs. 10	0.489	0.130	0.989	No
1270 vs. 270	0.305	0.0832	0.934	No

Elements in the plant tissues - Potassium - Roots

Two Way Analysis of Variance
General Linear Model (No Interactions)
Dependent Variable: K roots
Normality Test (Shapiro-Wilk) Passed (P = 0.675)
Equal Variance Test: Passed (P = 1.000)

Source of Variation	DF	SS	MS	F	P
Exp	3	51963.747	17321.249	1.323	0.288
Cd conc	9	587946.741	65327.416	4.991	<0.001
Residual	26	340295.561	13088.291		

Total	38	992327.555	26113.883

The difference in the mean values among the different levels of Exp is not great enough to exclude the possibility that the difference is just due to random sampling variability after allowing for the effects of differences in Cd conc. There is not a statistically significant difference (P = 0.288).

The difference in the mean values among the different levels of Cd conc is greater than would be expected by chance after allowing for effects of differences in Exp. There is a statistically significant difference (P = <0.001). To isolate which group(s) differ from the others use a multiple comparison procedure.

Power of performed test with alpha = 0.0500: for Exp : 0.102
Power of performed test with alpha = 0.0500: for Cd conc : 0.974

Least square means for Exp :

**Group**	**Mean**	**SEM**
272.000	227.375	38.765
273.000	201.358	36.178
278.000	280.866	36.178
280.000	287.840	36.178

Least square means for Cd conc :

**Group**	**Mean**	**SEM**
0.5	299.550	57.202
20	301.220	57.202
50	332.707	57.202
140	280.905	57.202
270	340.597	57.202
550	260.566	57.202
1270	160.663	57.202
3100	43.782	57.202
27500	30.156	57.202
10	443.451	66.962

All Pairwise Multiple Comparison Procedures (Holm-Sidak method):
Overall significance level = 0.05

Comparisons for factor: **Exp**

**Comparison**	**Diff of Means**	**t**	**P**	**P<0.050**
280.000 vs. 273.000	86.482	1.690	0.479	No
278.000 vs. 273.000	79.507	1.554	0.508	No
280.000 vs. 272.000	60.465	1.140	0.707	No
278.000 vs. 272.000	53.491	1.009	0.689	No
272.000 vs. 273.000	26.016	0.491	0.861	No
280.000 vs. 278.000	6.975	0.136	0.893	No

Comparisons for factor: **Cd conc**

**Comparison**	**Diff of Means**	**t**	**P**	**P<0.050**
10 vs. 27500	413.294	4.693	0.003	Yes
10 vs. 3100	399.669	4.538	0.005	Yes
270 vs. 27500	310.441	3.838	0.030	Yes
50 vs. 27500	302.551	3.740	0.038	Yes
270 vs. 3100	296.815	3.669	0.044	Yes
50 vs. 3100	288.925	3.572	0.055	No
20 vs. 27500	271.064	3.351	0.092	No
0.5 vs. 27500	269.393	3.330	0.094	No
10 vs. 1270	282.787	3.211	0.122	No
20 vs. 3100	257.438	3.182	0.127	No
0.5 vs. 3100	255.768	3.162	0.130	No
140 vs. 27500	250.749	3.100	0.146	No
140 vs. 3100	237.123	2.931	0.206	No
550 vs. 27500	230.409	2.848	0.239	No

550 vs. 3100	216.784	2.680	0.325	No
270 vs. 1270	179.934	2.224	0.657	No
50 vs. 1270	172.044	2.127	0.721	No
10 vs. 550	182.885	2.077	0.747	No
10 vs. 140	162.546	1.846	0.883	No
20 vs. 1270	140.557	1.738	0.923	No
0.5 vs. 1270	138.886	1.717	0.924	No
10 vs. 0.5	143.901	1.634	0.946	No
10 vs. 20	142.230	1.615	0.945	No
1270 vs. 27500	130.507	1.613	0.938	No
140 vs. 1270	120.241	1.486	0.966	No
1270 vs. 3100	116.881	1.445	0.970	No
10 vs. 50	110.743	1.257	0.991	No
550 vs. 1270	99.902	1.235	0.990	No
10 vs. 270	102.853	1.168	0.993	No
270 vs. 550	80.031	0.989	0.998	No
50 vs. 550	72.142	0.892	0.999	No
270 vs. 140	59.692	0.738	1.000	No
50 vs. 140	51.802	0.640	1.000	No
270 vs. 0.5	41.047	0.507	1.000	No
20 vs. 550	40.655	0.503	1.000	No
270 vs. 20	39.377	0.487	1.000	No
0.5 vs. 550	38.984	0.482	1.000	No
50 vs. 0.5	33.158	0.410	1.000	No
50 vs. 20	31.487	0.389	1.000	No
140 vs. 550	20.339	0.251	1.000	No
20 vs. 140	20.316	0.251	1.000	No
0.5 vs. 140	18.645	0.230	0.999	No
3100 vs. 27500	13.626	0.168	0.998	No
270 vs. 50	7.890	0.0975	0.994	No
20 vs. 0.5	1.671	0.0207	0.984	No

Elements in the plant tissues - Potassium - Seeds

Two Way Analysis of Variance
General Linear Model (No Interactions)
Dependent Variable: K seeds
Normality Test (Shapiro-Wilk) Failed (P < 0.050)
Equal Variance Test: Passed (P = 0.209)

**Source of Variation**	**DF**	**SS**	**MS**	**F**	**P**
Exp	3	151.439	50.480	11.022	<0.001
Cd conc	3	53.008	17.669	3.858	0.014
Residual	52	238.151	4.580		
Total	58	445.930	7.688		

The difference in the mean values among the different levels of Exp is greater than would be expected by chance after allowing for effects of differences in Cd conc. There is a statistically significant difference (P = <0.001). To isolate which group(s) differ from the others use a multiple comparison procedure.

The difference in the mean values among the different levels of Cd conc is greater than would be expected by chance after allowing for effects of differences in Exp. There is a statistically significant difference (P = 0.014). To isolate which group(s) differ from the others use a multiple comparison procedure.

Power of performed test with alpha = 0.0500: for Exp : 0.999
Power of performed test with alpha = 0.0500: for Cd conc : 0.649

Least square means for Exp :
Group Mean SEM

272.000	25.875	0.645
273.000	22.893	0.535
278.000	22.349	0.554
280.000	21.153	0.535

Least square means for Cd conc :

**Group**	**Mean**	**SEM**
0.5	23.702	0.535
20	23.767	0.535
50	21.520	0.535
10	23.282	0.671

All Pairwise Multiple Comparison Procedures (Holm-Sidak method):
Overall significance level = 0.05

Comparisons for factor: **Exp**

**Comparison**	**Diff of Means**	**t**	**P**	**P<0.050**
272.000 vs. 280.000	4.722	5.636	<0.001	Yes
272.000 vs. 278.000	3.526	4.148	<0.001	Yes
272.000 vs. 273.000	2.982	3.559	0.003	Yes
273.000 vs. 280.000	1.740	2.300	0.075	No
278.000 vs. 280.000	1.196	1.554	0.237	No
273.000 vs. 278.000	0.544	0.706	0.483	No

Comparisons for factor: **Cd conc**

**Comparison**	**Diff of Means**	**t**	**P**	**P<0.050**
20 vs. 50	2.247	2.969	0.027	Yes
0.5 vs. 50	2.181	2.883	0.028	Yes
10 vs. 50	1.762	2.053	0.168	No
20 vs. 10	0.485	0.565	0.923	No
0.5 vs. 10	0.420	0.489	0.861	No
20 vs. 0.5	0.0655	0.0865	0.931	No

Elements in the plant tissues - Magnesium - Harvested leaves

Two Way Analysis of Variance
General Linear Model (No Interactions)
Dependent Variable: Mg content harvest
Normality Test (Shapiro-Wilk) Failed (P < 0.050)
Equal Variance Test: Passed (P = 0.167)

**Source of Variation**	**DF**	**SS**	**MS**	**F**	**P**
Exp	3	363.444	121.148	0.726	0.538
Cd conc	9	5087.950	565.328	3.390	<0.001
Residual	116	19344.346	166.762		
Total	128	24913.180	194.634		

The difference in the mean values among the different levels of Exp is not great enough to exclude the possibility that the difference is just due to random sampling variability after allowing for the effects of differences in Cd conc. There is not a statistically significant difference (P = 0.538).

The difference in the mean values among the different levels of Cd conc is greater than would be expected by chance after allowing for effects of differences in Exp. There is a statistically significant difference (P = <0.001). To isolate which group(s) differ from the others use a multiple comparison procedure.

Power of performed test with alpha = 0.0500: for Exp : 0.0500
Power of performed test with alpha = 0.0500: for Cd conc : 0.908

Least square means for Exp :

Group	Mean	SEM
272.000	8.610	2.363
273.000	6.230	2.070
278.000	6.268	2.733
280.000	10.177	2.196

Least square means for Cd conc :

Group	Mean	SEM
0.5	9.333	3.228
20	19.397	3.228
50	16.440	3.970
140	3.683	3.584
270	4.776	3.739
550	3.384	3.739
1270	2.561	3.595
3100	2.120	4.169
27500	2.464	3.337
10	14.056	3.954

All Pairwise Multiple Comparison Procedures (Holm-Sidak method):
Overall significance level = 0.05

Comparisons for factor: **Exp**

Comparison	Diff of Means	t	P	P<0.050
280.000 vs. 273.000	3.947	1.308	0.725	No
280.000 vs. 278.000	3.908	1.115	0.789	No
272.000 vs. 273.000	2.380	0.758	0.909	No
272.000 vs. 278.000	2.342	0.648	0.888	No
280.000 vs. 272.000	1.566	0.486	0.862	No
278.000 vs. 273.000	0.0385	0.0112	0.991	No

Comparisons for factor: **Cd conc**

Comparison	Diff of Means	t	P	P<0.050
20 vs. 27500	16.934	3.647	0.018	Yes
20 vs. 1270	16.836	3.484	0.030	Yes
20 vs. 3100	17.278	3.276	0.058	No
20 vs. 140	15.715	3.258	0.060	No
20 vs. 550	16.013	3.242	0.062	No
20 vs. 270	14.621	2.960	0.139	No
50 vs. 27500	13.977	2.695	0.271	No
50 vs. 1270	13.879	2.591	0.338	No
50 vs. 3100	14.321	2.487	0.413	No
50 vs. 550	13.057	2.394	0.485	No
50 vs. 140	12.758	2.385	0.483	No
10 vs. 27500	11.592	2.240	0.605	No
20 vs. 0.5	10.065	2.204	0.627	No
10 vs. 1270	11.495	2.151	0.665	No
50 vs. 270	11.664	2.139	0.664	No
10 vs. 3100	11.936	2.077	0.706	No
10 vs. 550	10.672	1.961	0.789	No
10 vs. 140	10.373	1.944	0.791	No
10 vs. 270	9.280	1.705	0.924	No
0.5 vs. 27500	6.869	1.479	0.981	No
0.5 vs. 1270	6.772	1.401	0.989	No
50 vs. 0.5	7.108	1.389	0.988	No
0.5 vs. 3100	7.213	1.368	0.988	No
0.5 vs. 550	5.949	1.204	0.997	No
0.5 vs. 140	5.650	1.171	0.997	No
20 vs. 10	5.341	1.046	0.999	No
10 vs. 0.5	4.723	0.925	1.000	No
0.5 vs. 270	4.557	0.922	1.000	No

20 vs. 50	2.957	0.578	1.000	No
270 vs. 3100	2.656	0.474	1.000	No
270 vs. 27500	2.312	0.461	1.000	No
270 vs. 1270	2.215	0.427	1.000	No
50 vs. 10	2.385	0.426	1.000	No
140 vs. 3100	1.563	0.284	1.000	No
270 vs. 550	1.392	0.263	1.000	No
140 vs. 27500	1.219	0.249	1.000	No
550 vs. 3100	1.264	0.226	1.000	No
140 vs. 1270	1.121	0.221	1.000	No
270 vs. 140	1.093	0.211	1.000	No
550 vs. 27500	0.920	0.184	1.000	No
550 vs. 1270	0.823	0.159	1.000	No
1270 vs. 3100	0.441	0.0802	1.000	No
27500 vs. 3100	0.344	0.0644	1.000	No
140 vs. 550	0.299	0.0577	0.998	No
1270 vs. 27500	0.0975	0.0199	0.984	No

Elements in the plant tissues - Magnesium - w10 leaves

Two Way Analysis of Variance
General Linear Model (No Interactions)
Dependent Variable: Mg w10
Normality Test (Shapiro-Wilk) Failed (P < 0.050)
Equal Variance Test: Failed (P < 0.050)

**Source of Variation**	**DF**	**SS**	**MS**	**F**	**P**
Exp	3	91.347	30.449	1.316	0.282
Cd conc	8	814.139	101.767	4.399	<0.001
Residual	43	994.862	23.136		
Total	54	1946.287	36.042		

The difference in the mean values among the different levels of Exp is not great enough to exclude the possibility that the difference is just due to random sampling variability after allowing for the effects of differences in Cd conc. There is not a statistically significant difference (P = 0.282).

The difference in the mean values among the different levels of Cd conc is greater than would be expected by chance after allowing for effects of differences in Exp. There is a statistically significant difference (P = <0.001). To isolate which group(s) differ from the others use a multiple comparison procedure.

Power of performed test with alpha = 0.0500: for Exp : 0.104
Power of performed test with alpha = 0.0500: for Cd conc : 0.955

Least square means for Exp :

**Group**	**Mean**	**SEM**
272.000	5.482	1.878
273.000	7.381	1.264
278.000	5.383	1.181
280.000	8.449	1.261

Least square means for Cd conc :

**Group**	**Mean**	**SEM**
0.5	8.012	1.830
20	11.547	1.830
50	10.247	1.830
140	10.330	1.830
270	3.064	1.830
550	2.526	1.977
1270	1.650	1.977
10	10.450	2.025

3100	2.236	3.474

All Pairwise Multiple Comparison Procedures (Holm-Sidak method):
Overall significance level = 0.05

Comparisons for factor: **Exp**

**Comparison**	**Diff of Means**	**t**	**P**	**P<0.050**
280.000 vs. 278.000	3.066	1.774	0.406	No
280.000 vs. 272.000	2.966	1.311	0.666	No
273.000 vs. 278.000	1.998	1.155	0.691	No
273.000 vs. 272.000	1.899	0.839	0.791	No
280.000 vs. 273.000	1.068	0.598	0.800	No
272.000 vs. 278.000	0.0996	0.0449	0.964	No

Comparisons for factor: **Cd conc**

**Comparison**	**Diff of Means**	**t**	**P**	**P<0.050**
20 vs. 1270	9.898	3.674	0.023	Yes
20 vs. 550	9.021	3.348	0.058	No
20 vs. 270	8.484	3.278	0.068	No
140 vs. 1270	8.681	3.222	0.077	No
50 vs. 1270	8.598	3.191	0.081	No
10 vs. 1270	8.800	3.110	0.098	No
140 vs. 550	7.804	2.896	0.163	No
50 vs. 550	7.721	2.866	0.170	No
140 vs. 270	7.267	2.807	0.190	No
10 vs. 550	7.923	2.800	0.187	No
50 vs. 270	7.184	2.775	0.191	No
10 vs. 270	7.386	2.706	0.217	No
20 vs. 3100	9.311	2.371	0.418	No
0.5 vs. 1270	6.363	2.362	0.412	No
140 vs. 3100	8.094	2.061	0.640	No
10 vs. 3100	8.214	2.043	0.638	No
50 vs. 3100	8.011	2.040	0.622	No
0.5 vs. 550	5.486	2.036	0.607	No
0.5 vs. 270	4.949	1.912	0.687	No
0.5 vs. 3100	5.776	1.471	0.935	No
20 vs. 0.5	3.535	1.366	0.957	No
140 vs. 0.5	2.318	0.896	0.999	No
10 vs. 0.5	2.437	0.893	0.999	No
50 vs. 0.5	2.235	0.863	0.998	No
270 vs. 1270	1.414	0.525	1.000	No
20 vs. 50	1.300	0.502	1.000	No
20 vs. 140	1.217	0.470	1.000	No
20 vs. 10	1.098	0.402	1.000	No
550 vs. 1270	0.877	0.314	1.000	No
270 vs. 3100	0.827	0.211	1.000	No
270 vs. 550	0.537	0.199	1.000	No
3100 vs. 1270	0.587	0.147	1.000	No
10 vs. 50	0.203	0.0743	1.000	No
550 vs. 3100	0.290	0.0726	1.000	No
10 vs. 140	0.120	0.0438	0.999	No
140 vs. 50	0.0832	0.0321	0.975	No

Elements in the plant tissues - Magnesium - w5 leaves

Two Way Analysis of Variance
General Linear Model (No Interactions)
Dependent Variable: Mg w5
Normality Test (Shapiro-Wilk) Failed (P < 0.050)
Equal Variance Test: Failed (P < 0.050)

**Source of Variation**	**DF**	**SS**	**M**	**S**	**F**	**P**
Exp	3	4.454		1.485	3.031	0.036
Cd conc	9	136.725		15.192	31.015	<0.001
Residual	62	30.368		0.490		
Total	74	176.625		2.387		

The difference in the mean values among the different levels of Exp is greater than would be expected by chance after allowing for effects of differences in Cd conc. There is a statistically significant difference (P = 0.036). To isolate which group(s) differ from the others use a multiple comparison procedure.

The difference in the mean values among the different levels of Cd conc is greater than would be expected by chance after allowing for effects of differences in Exp. There is a statistically significant difference (P = <0.001). To isolate which group(s) differ from the others use a multiple comparison procedure.

Power of performed test with alpha = 0.0500: for Exp : 0.489
Power of performed test with alpha = 0.0500: for Cd conc : 1.000

Least square means for Exp :

**Group**	**Mean**	**SEM**
272.000	3.506	0.168
273.000	4.030	0.156
278.000	4.169	0.161
280.000	3.992	0.168

Least square means for Cd conc :

**Group**	**Mean**	**SEM**
0.5	5.310	0.247
20	5.362	0.247
50	5.869	0.247
140	4.015	0.247
270	3.146	0.247
550	2.616	0.247
1270	2.801	0.247
3100	2.330	0.247
27500	2.335	0.318
10	5.457	0.290

All Pairwise Multiple Comparison Procedures (Holm-Sidak method):
Overall significance level = 0.05

Comparisons for factor: **Exp**

**Comparison**	**Diff of Means**	**t**	**P**	**P<0.050**
278.000 vs. 272.000	0.663	2.850	0.035	Yes
273.000 vs. 272.000	0.525	2.287	0.122	No
280.000 vs. 272.000	0.487	2.049	0.167	No
278.000 vs. 280.000	0.176	0.757	0.835	No
278.000 vs. 273.000	0.139	0.617	0.788	No
273.000 vs. 280.000	0.0377	0.164	0.870	No

Comparisons for factor: **Cd conc**

**Comparison**	**Diff of Means**	**t**	**P**	**P<0.050**
50 vs. 3100	3.540	10.115	<0.001	Yes
50 vs. 550	3.253	9.297	<0.001	Yes
50 vs. 27500	3.534	8.774	<0.001	Yes
50 vs. 1270	3.068	8.768	<0.001	Yes
20 vs. 3100	3.033	8.666	<0.001	Yes
0.5 vs. 3100	2.981	8.518	<0.001	Yes
10 vs. 3100	3.127	8.208	<0.001	Yes
20 vs. 550	2.746	7.848	<0.001	Yes
50 vs. 270	2.723	7.782	<0.001	Yes

0.5 vs. 550	2.694	7.700	<0.001	Yes
20 vs. 27500	3.027	7.516	<0.001	Yes
10 vs. 550	2.841	7.457	<0.001	Yes
0.5 vs. 27500	2.975	7.386	<0.001	Yes
20 vs. 1270	2.561	7.319	<0.001	Yes
10 vs. 27500	3.122	7.259	<0.001	Yes
0.5 vs. 1270	2.509	7.171	<0.001	Yes
10 vs. 1270	2.656	6.971	<0.001	Yes
20 vs. 270	2.216	6.334	<0.001	Yes
0.5 vs. 270	2.164	6.185	<0.001	Yes
10 vs. 270	2.311	6.065	<0.001	Yes
50 vs. 140	1.854	5.298	<0.001	Yes
140 vs. 3100	1.686	4.817	<0.001	Yes
140 vs. 27500	1.680	4.172	0.002	Yes
140 vs. 550	1.400	3.999	0.004	Yes
20 vs. 140	1.347	3.849	0.006	Yes
10 vs. 140	1.442	3.784	0.007	Yes
0.5 vs. 140	1.295	3.700	0.009	Yes
140 vs. 1270	1.214	3.470	0.017	Yes
140 vs. 270	0.869	2.485	0.236	No
270 vs. 3100	0.816	2.333	0.310	No
270 vs. 27500	0.811	2.013	0.525	No
50 vs. 0.5	0.559	1.597	0.820	No
270 vs. 550	0.530	1.515	0.848	No
50 vs. 20	0.507	1.449	0.863	No
1270 vs. 3100	0.471	1.347	0.892	No
1270 vs. 27500	0.466	1.157	0.945	No
50 vs. 10	0.412	1.082	0.950	No
270 vs. 1270	0.345	0.986	0.958	No
550 vs. 3100	0.286	0.818	0.977	No
550 vs. 27500	0.281	0.697	0.982	No
1270 vs. 550	0.185	0.529	0.990	No
10 vs. 0.5	0.147	0.385	0.992	No
10 vs. 20	0.0948	0.249	0.993	No
20 vs. 0.5	0.0520	0.149	0.986	No
27500 vs. 3100	0.00544	0.0135	0.989	No

Elements in the plant tissues - Magnesium - Stems

Two Way Analysis of Variance
General Linear Model (No Interactions)
Dependent Variable: Mg stems
Normality Test (Shapiro-Wilk) Failed (P < 0.050)
Equal Variance Test: Failed (P < 0.050)

**Source of Variation**	**DF**	**SS**	**MS**	**F**	**P**
Exp	3	116.110	38.703	3.941	0.010
Cd conc	9	1584.968	176.108	17.932	<0.001
Residual	127	1247.225	9.821		
Total	139	3010.944	21.661		

The difference in the mean values among the different levels of Exp is greater than would be expected by chance after allowing for effects of differences in Cd conc. There is a statistically significant difference (P = 0.010). To isolate which group(s) differ from the others use a multiple comparison procedure.

The difference in the mean values among the different levels of Cd conc is greater than would be expected by chance after allowing for effects of differences in Exp. There is a statistically significant difference (P = <0.001). To isolate which group(s) differ from the others use a multiple comparison procedure.

Power of performed test with alpha = 0.0500: for Exp : 0.686
Power of performed test with alpha = 0.0500: for Cd conc : 1.000

Least square means for Exp :

**Group**	**Mean**	**SEM**
272.000	8.522	0.540
273.000	8.687	0.495
278.000	9.419	0.619
280.000	6.885	0.509

Least square means for Cd conc :

**Group**	**Mean**	**SEM**
0.5	10.580	0.783
20	11.354	0.783
50	12.949	0.783
140	6.884	0.841
270	7.137	0.921
550	7.306	0.841
1270	5.745	0.838
3100	4.627	0.913
27500	3.279	0.783
10	13.921	1.004

All Pairwise Multiple Comparison Procedures (Holm-Sidak method):
Overall significance level = 0.05

Comparisons for factor: **Exp**

**Comparison**	**Diff of Means**	**t**	**P**	**P<0.050**
278.000 vs. 280.000	2.534	3.162	0.012	Yes
273.000 vs. 280.000	1.802	2.536	0.061	No
272.000 vs. 280.000	1.637	2.205	0.112	No
278.000 vs. 272.000	0.897	1.091	0.622	No
278.000 vs. 273.000	0.732	0.923	0.588	No
273.000 vs. 272.000	0.165	0.225	0.822	No

Comparisons for factor: **Cd conc**

**Comparison**	**Diff of Means**	**t**	**P**	**P<0.050**
50 vs. 27500	9.670	8.728	<0.001	Yes
10 vs. 27500	10.642	8.358	<0.001	Yes
20 vs. 27500	8.075	7.288	<0.001	Yes
50 vs. 3100	8.322	6.919	<0.001	Yes
10 vs. 3100	9.294	6.851	<0.001	Yes
0.5 vs. 27500	7.302	6.590	<0.001	Yes
50 vs. 1270	7.204	6.278	<0.001	Yes
10 vs. 1270	8.176	6.252	<0.001	Yes
20 vs. 3100	6.726	5.592	<0.001	Yes
10 vs. 140	7.037	5.374	<0.001	Yes
50 vs. 140	6.065	5.277	<0.001	Yes
10 vs. 550	6.615	5.052	<0.001	Yes
10 vs. 270	6.784	4.981	<0.001	Yes
0.5 vs. 3100	5.953	4.950	<0.001	Yes
50 vs. 550	5.643	4.910	<0.001	Yes
20 vs. 1270	5.609	4.888	<0.001	Yes
50 vs. 270	5.812	4.808	<0.001	Yes
0.5 vs. 1270	4.836	4.214	0.001	Yes
20 vs. 140	4.469	3.889	0.004	Yes
20 vs. 550	4.047	3.522	0.015	Yes
550 vs. 27500	4.027	3.505	0.016	Yes
20 vs. 270	4.217	3.488	0.016	Yes
0.5 vs. 140	3.696	3.216	0.037	Yes
270 vs. 27500	3.858	3.191	0.039	Yes

140 vs. 27500	3.606	3.137	0.044	Yes
0.5 vs. 550	3.274	2.849	0.098	No
0.5 vs. 270	3.444	2.848	0.093	No
10 vs. 0.5	3.340	2.623	0.162	No
550 vs. 3100	2.679	2.159	0.432	No
1270 vs. 27500	2.466	2.149	0.421	No
50 vs. 0.5	2.369	2.138	0.409	No
10 vs. 20	2.567	2.016	0.482	No
270 vs. 3100	2.509	1.936	0.522	No
140 vs. 3100	2.257	1.819	0.588	No
50 vs. 20	1.595	1.440	0.838	No
550 vs. 1270	1.562	1.315	0.880	No
3100 vs. 27500	1.349	1.121	0.937	No
270 vs. 1270	1.392	1.118	0.916	No
140 vs. 1270	1.140	0.960	0.945	No
1270 vs. 3100	1.117	0.901	0.937	No
10 vs. 50	0.972	0.763	0.948	No
20 vs. 0.5	0.773	0.698	0.931	No
550 vs. 140	0.422	0.355	0.979	No
270 vs. 140	0.252	0.202	0.974	No
550 vs. 270	0.170	0.136	0.892	No

Elements in the plant tissues - Magnesium - Roots

Two Way Analysis of Variance
General Linear Model (No Interactions)
Dependent Variable: Mg roots
Normality Test (Shapiro-Wilk) Passed (P = 0.383)
Equal Variance Test: Passed (P = 1.000)

Source of Variation	DF	SS	MS	F	P
Exp	3	127.291	42.430	4.062	0.017
Cd conc	9	592.325	65.814	6.300	<0.001
Residual	26	271.621	10.447		
Total	38	992.930	26.130		

The difference in the mean values among the different levels of Exp is greater than would be expected by chance after allowing for effects of differences in Cd conc. There is a statistically significant difference (P = 0.017). To isolate which group(s) differ from the others use a multiple comparison procedure.

The difference in the mean values among the different levels of Cd conc is greater than would be expected by chance after allowing for effects of differences in Exp. There is a statistically significant difference (P = <0.001). To isolate which group(s) differ from the others use a multiple comparison procedure.

Power of performed test with alpha = 0.0500: for Exp : 0.647
Power of performed test with alpha = 0.0500: for Cd conc : 0.996

Least square means for Exp :

Group	Mean	SEM
272.000	8.025	1.095
273.000	9.210	1.022
278.000	8.293	1.022
280.000	4.511	1.022

Least square means for Cd conc :

Group	Mean	SEM
0.5	8.475	1.616
20	8.119	1.616
50	6.397	1.616
140	14.880	1.616

270	9.981	1.616
550	9.891	1.616
1270	8.596	1.616
3100	1.150	1.616
27500	1.472	1.616
10	6.137	1.892

All Pairwise Multiple Comparison Procedures (Holm-Sidak method):
Overall significance level = 0.05

Comparisons for factor: **Exp**

**Comparison**	**Diff of Means**	**t**	**P**	**P<0.050**
273.000 vs. 280.000	4.699	3.251	0.019	Yes
278.000 vs. 280.000	3.783	2.617	0.071	No
272.000 vs. 280.000	3.514	2.346	0.103	No
273.000 vs. 272.000	1.185	0.791	0.821	No
273.000 vs. 278.000	0.917	0.634	0.781	No
278.000 vs. 272.000	0.269	0.179	0.859	No

Comparisons for factor: **Cd conc**

**Comparison**	**Diff of Means**	**t**	**P**	**P<0.050**
140 vs. 3100	13.731	6.008	<0.001	Yes
140 vs. 27500	13.408	5.867	<0.001	Yes
270 vs. 3100	8.831	3.864	0.028	Yes
550 vs. 3100	8.741	3.824	0.031	Yes
270 vs. 27500	8.508	3.723	0.039	Yes
140 vs. 50	8.484	3.712	0.039	Yes
550 vs. 27500	8.418	3.683	0.041	Yes
140 vs. 10	8.744	3.514	0.060	No
1270 vs. 3100	7.446	3.258	0.109	No
0.5 vs. 3100	7.325	3.205	0.120	No
1270 vs. 27500	7.123	3.117	0.144	No
0.5 vs. 27500	7.002	3.064	0.158	No
20 vs. 3100	6.969	3.049	0.159	No
140 vs. 20	6.761	2.958	0.189	No
20 vs. 27500	6.647	2.908	0.204	No
140 vs. 0.5	6.406	2.803	0.248	No
140 vs. 1270	6.285	2.750	0.268	No
50 vs. 3100	5.247	2.296	0.574	No
140 vs. 550	4.990	2.183	0.651	No
50 vs. 27500	4.925	2.155	0.660	No
140 vs. 270	4.900	2.144	0.654	No
10 vs. 3100	4.987	2.004	0.746	No
10 vs. 27500	4.664	1.875	0.821	No
270 vs. 50	3.584	1.568	0.952	No
270 vs. 10	3.844	1.545	0.952	No
550 vs. 50	3.494	1.529	0.949	No
550 vs. 10	3.754	1.509	0.947	No
1270 vs. 10	2.459	0.988	0.999	No
1270 vs. 50	2.199	0.962	0.999	No
0.5 vs. 10	2.338	0.940	0.999	No
0.5 vs. 50	2.078	0.909	0.999	No
270 vs. 20	1.862	0.815	1.000	No
20 vs. 10	1.982	0.797	0.999	No
550 vs. 20	1.772	0.775	0.999	No
20 vs. 50	1.722	0.754	0.999	No
270 vs. 0.5	1.506	0.659	0.999	No
550 vs. 0.5	1.416	0.620	0.999	No
270 vs. 1270	1.385	0.606	0.998	No
550 vs. 1270	1.295	0.567	0.998	No
1270 vs. 20	0.477	0.209	1.000	No

0.5 vs. 20	0.356	0.156	1.000	No
27500 vs. 3100	0.323	0.141	1.000	No
50 vs. 10	0.260	0.105	0.999	No
1270 vs. 0.5	0.121	0.0530	0.998	No
270 vs. 550	0.0902	0.0395	0.969	No

Elements in the plant tissues - Magnesium - Seeds

Two Way Analysis of Variance
General Linear Model (No Interactions)
Dependent Variable: Mg seeds
Normality Test (Shapiro-Wilk) Passed (P = 0.078)
Equal Variance Test: Passed (P = 0.152)

**Source of Variation**	**DF**	**SS**	**MS**	**F**	**P**
Exp	3	1.025	0.342	3.070	0.036
Cd conc	3	1.602	0.534	4.801	0.005
Residual	52	5.785	0.111		
Total	58	8.207	0.141		

The difference in the mean values among the different levels of Exp is greater than would be expected by chance after allowing for effects of differences in Cd conc. There is a statistically significant difference (P = 0.036). To isolate which group(s) differ from the others use a multiple comparison procedure.

The difference in the mean values among the different levels of Cd conc is greater than would be expected by chance after allowing for effects of differences in Exp. There is a statistically significant difference (P = 0.005). To isolate which group(s) differ from the others use a multiple comparison procedure.

Power of performed test with alpha = 0.0500: for Exp : 0.492
Power of performed test with alpha = 0.0500: for Cd conc : 0.792

Least square means for Exp :

**Group**	**Mean**	**SEM**
272.000	2.965	0.100
273.000	2.691	0.0834
278.000	2.601	0.0863
280.000	2.625	0.0834

Least square means for Cd conc :

**Group**	**Mean**	**SEM**
0.5	2.799	0.0834
20	2.770	0.0834
50	2.442	0.0834
10	2.872	0.105

All Pairwise Multiple Comparison Procedures (Holm-Sidak method):
Overall significance level = 0.05

Comparisons for factor: **Exp**

**Comparison**	**Diff of Means**	**t**	**P**	**P<0.050**
272.000 vs. 278.000	0.364	2.747	0.048	Yes
272.000 vs. 280.000	0.340	2.606	0.058	No
272.000 vs. 273.000	0.274	2.095	0.154	No
273.000 vs. 278.000	0.0903	0.752	0.838	No
273.000 vs. 280.000	0.0667	0.566	0.818	No
280.000 vs. 278.000	0.0236	0.196	0.845	No

Comparisons for factor: **Cd conc**

**Comparison**	**Diff of Means**	**t**	**P**	**P<0.050**
10 vs. 50	0.430	3.218	0.013	Yes

0.5 vs. 50	0.357	3.028	0.019	Yes
20 vs. 50	0.329	2.788	0.029	Yes
10 vs. 20	0.102	0.760	0.834	No
10 vs. 0.5	0.0733	0.548	0.829	No
0.5 vs. 20	0.0283	0.240	0.811	No

Elements in the plant tissues - Phosphate - Harvested leaves

Two Way Analysis of Variance
General Linear Model (No Interactions)
Dependent Variable: P harvest
Normality Test (Shapiro-Wilk) Failed (P < 0.050)
Equal Variance Test: Failed (P < 0.050)

**Source of Variation**	**DF**	**SS**	**MS**	**F**	**P**
Exp	3	2132.531	710.844	1.218	0.306
Cd conc	9	15548.172	1727.575	2.961	0.003
Residual	116	67671.790	583.377		
Total	128	85505.331	668.010		

The difference in the mean values among the different levels of Exp is not great enough to exclude the possibility that the difference is just due to random sampling variability after allowing for the effects of differences in Cd conc. There is not a statistically significant difference (P = 0.306).

The difference in the mean values among the different levels of Cd conc is greater than would be expected by chance after allowing for effects of differences in Exp. There is a statistically significant difference (P = 0.003). To isolate which group(s) differ from the others use a multiple comparison procedure.

Power of performed test with alpha = 0.0500: for Exp : 0.0882
Power of performed test with alpha = 0.0500: for Cd conc : 0.826

Least square means for Exp :

**Group**	**Mean**	**SEM**
272.000	31.508	4.420
273.000	26.463	3.872
278.000	24.400	5.112
280.000	35.050	4.106

Least square means for Cd conc :

**Group**	**Mean**	**SEM**
0.5	20.098	6.038
20	35.079	6.038
50	32.931	7.426
140	37.986	6.704
270	43.215	6.993
550	42.262	6.993
1270	23.141	6.725
3100	20.005	7.798
27500	7.931	6.242
10	30.904	7.396

All Pairwise Multiple Comparison Procedures (Holm-Sidak method):
Overall significance level = 0.05

Comparisons for factor: **Exp**

**Comparison**	**Diff of Means**	**t**	**P**	**P<0.050**
280.000 vs. 278.000	10.650	1.624	0.493	No
280.000 vs. 273.000	8.586	1.521	0.504	No
272.000 vs. 278.000	7.108	1.052	0.753	No

272.000 vs. 273.000	5.045	0.859	0.776	No
280.000 vs. 272.000	3.542	0.587	0.805	No
273.000 vs. 278.000	2.063	0.322	0.748	No

Comparisons for factor: **Cd conc**

**Comparison**	**Diff of Means**	**t**	**P**	**P<0.050**
270 vs. 27500	35.283	3.764	0.012	Yes
550 vs. 27500	34.331	3.663	0.016	Yes
140 vs. 27500	30.055	3.281	0.057	No
20 vs. 27500	27.148	3.126	0.090	No
50 vs. 27500	24.999	2.577	0.370	No
270 vs. 0.5	23.116	2.502	0.425	No
550 vs. 0.5	22.164	2.399	0.508	No
10 vs. 27500	22.972	2.374	0.522	No
270 vs. 3100	23.210	2.216	0.659	No
550 vs. 3100	22.257	2.125	0.730	No
270 vs. 1270	20.074	2.069	0.767	No
140 vs. 0.5	17.888	1.983	0.824	No
550 vs. 1270	19.121	1.971	0.823	No
20 vs. 0.5	14.981	1.754	0.935	No
140 vs. 3100	17.982	1.748	0.932	No
1270 vs. 27500	15.210	1.658	0.958	No
140 vs. 1270	14.845	1.563	0.976	No
20 vs. 3100	15.075	1.528	0.979	No
0.5 vs. 27500	12.167	1.401	0.992	No
50 vs. 0.5	12.832	1.341	0.995	No
20 vs. 1270	11.938	1.321	0.995	No
270 vs. 10	12.311	1.210	0.998	No
3100 vs. 27500	12.073	1.209	0.997	No
50 vs. 3100	12.926	1.200	0.997	No
10 vs. 0.5	10.805	1.132	0.998	No
550 vs. 10	11.359	1.116	0.998	No
10 vs. 3100	10.899	1.014	0.999	No
270 vs. 50	10.284	1.008	0.999	No
50 vs. 1270	9.790	0.977	0.999	No
550 vs. 50	9.331	0.915	0.999	No
270 vs. 20	8.135	0.881	0.999	No
550 vs. 20	7.183	0.777	1.000	No
10 vs. 1270	7.763	0.777	0.999	No
140 vs. 10	7.083	0.710	1.000	No
270 vs. 140	5.228	0.540	1.000	No
140 vs. 50	5.055	0.505	1.000	No
550 vs. 140	4.276	0.441	1.000	No
20 vs. 10	4.176	0.437	1.000	No
1270 vs. 0.5	3.042	0.337	1.000	No
140 vs. 20	2.907	0.322	1.000	No
1270 vs. 3100	3.136	0.305	0.999	No
20 vs. 50	2.148	0.224	0.999	No
50 vs. 10	2.027	0.193	0.996	No
270 vs. 550	0.953	0.0963	0.994	No
0.5 vs. 3100	0.0939	0.00952	0.992	No

Elements in the plant tissues - Phosphate - w10 leaves

Two Way Analysis of Variance
General Linear Model (No Interactions)
Dependent Variable: P w10
Normality Test (Shapiro-Wilk) Failed (P < 0.050)
Equal Variance Test: Failed (P < 0.050)

Source of Variation	DF	SS	MS	F	P
Exp	3	7552.456	2517.485	2.295	0.092
Cd conc	8	17885.718	2235.715	2.038	0.065
Residual	42	46064.193	1096.766		
Total	53	70808.139	1336.003		

The difference in the mean values among the different levels of Exp is not great enough to exclude the possibility that the difference is just due to random sampling variability after allowing for the effects of differences in Cd conc. There is not a statistically significant difference (P = 0.092).

The difference in the mean values among the different levels of Cd conc is not great enough to exclude the possibility that the difference is just due to random sampling variability after allowing for the effects of differences in Exp. There is not a statistically significant difference (P = 0.065).

Power of performed test with alpha = 0.0500: for Exp : 0.312
Power of performed test with alpha = 0.0500: for Cd conc : 0.412

Least square means for Exp :

Group	Mean	SEM
272.000	32.969	12.935
273.000	43.711	8.706
278.000	19.262	8.131
280.000	47.464	8.995

Least square means for Cd conc :

Group	Mean	SEM
0.5	19.613	12.602
20	28.599	12.602
50	25.878	12.602
140	83.904	13.615
270	43.383	12.602
550	38.745	13.615
1270	30.675	13.615
10	27.299	13.942
3100	24.570	23.926

Elements in the plant tissues - Phosphate - w5 leaves

Two Way Analysis of Variance
General Linear Model (No Interactions)
Dependent Variable: P w5
Normality Test (Shapiro-Wilk) Failed (P < 0.050)
Equal Variance Test: Failed (P < 0.050)

Source of Variation	DF	SS	MS	F	P
Exp	3	37.526	12.509	0.551	0.649
Cd conc	9	4093.383	454.820	20.047	<0.001
Residual	62	1406.619	22.687		
Total	74	5555.543	75.075		

The difference in the mean values among the different levels of Exp is not great enough to exclude the possibility that the difference is just due to random sampling variability after allowing for the effects of differences in Cd conc. There is not a statistically significant difference (P = 0.649).

The difference in the mean values among the different levels of Cd conc is greater than would be expected by chance after allowing for effects of differences in Exp. There is a statistically significant difference (P = <0.001). To isolate which group(s) differ from the others use a multiple comparison procedure.

Power of performed test with alpha = 0.0500: for Exp : 0.0500
Power of performed test with alpha = 0.0500: for Cd conc : 1.000

Least square means for Exp :

Group	Mean	SEM
272.000	14.233	1.141
273.000	15.026	1.065
278.000	16.223	1.098
280.000	14.923	1.145

Least square means for Cd conc :

Group	Mean	SEM
0.5	10.089	1.684
20	10.089	1.684
50	12.869	1.684
140	25.468	1.684
270	27.468	1.684
550	23.580	1.684
1270	17.185	1.684
3100	9.334	1.684
27500	3.835	2.163
10	11.095	1.972

All Pairwise Multiple Comparison Procedures (Holm-Sidak method):
Overall significance level = 0.05

Comparisons for factor: **Exp**

Comparison	Diff of Means	t	P	P<0.050
278.000 vs. 272.000	1.990	1.256	0.764	No
278.000 vs. 280.000	1.299	0.819	0.932	No
278.000 vs. 273.000	1.197	0.783	0.899	No
273.000 vs. 272.000	0.793	0.508	0.942	No
280.000 vs. 272.000	0.690	0.427	0.892	No
273.000 vs. 280.000	0.102	0.0653	0.948	No

Comparisons for factor: **Cd conc**

Comparison	Diff of Means	t	P	P<0.050
270 vs. 27500	23.633	8.621	<0.001	Yes
140 vs. 27500	21.633	7.892	<0.001	Yes
270 vs. 3100	18.134	7.614	<0.001	Yes
270 vs. 0.5	17.379	7.297	<0.001	Yes
270 vs. 20	17.378	7.297	<0.001	Yes
550 vs. 27500	19.745	7.203	<0.001	Yes
140 vs. 3100	16.134	6.774	<0.001	Yes
140 vs. 0.5	15.379	6.457	<0.001	Yes
140 vs. 20	15.378	6.457	<0.001	Yes
270 vs. 10	16.373	6.314	<0.001	Yes
270 vs. 50	14.599	6.130	<0.001	Yes
550 vs. 3100	14.246	5.982	<0.001	Yes
550 vs. 0.5	13.491	5.665	<0.001	Yes
550 vs. 20	13.490	5.664	<0.001	Yes
140 vs. 10	14.373	5.543	<0.001	Yes
140 vs. 50	12.599	5.290	<0.001	Yes
1270 vs. 27500	13.350	4.870	<0.001	Yes
550 vs. 10	12.485	4.815	<0.001	Yes
550 vs. 50	10.711	4.498	<0.001	Yes
270 vs. 1270	10.283	4.318	0.002	Yes
140 vs. 1270	8.283	3.478	0.023	Yes
1270 vs. 3100	7.850	3.296	0.038	Yes
50 vs. 27500	9.034	3.296	0.037	Yes
1270 vs. 0.5	7.095	2.979	0.087	No
1270 vs. 20	7.095	2.979	0.083	No
550 vs. 1270	6.395	2.685	0.170	No
10 vs. 27500	7.260	2.480	0.262	No

1270 vs. 10	6.090	2.348	0.331	No	
20 vs. 27500	6.255	2.282	0.360	No	
0.5 vs. 27500	6.254	2.282	0.343	No	
3100 vs. 27500	5.499	2.006	0.531	No	
1270 vs. 50	4.316	1.812	0.663	No	
270 vs. 550	3.888	1.633	0.772	No	
50 vs. 3100	3.534	1.484	0.843	No	
50 vs. 0.5	2.779	1.167	0.956	No	
50 vs. 20	2.779	1.167	0.942	No	
270 vs. 140	2.000	0.840	0.991	No	
140 vs. 550	1.888	0.793	0.989	No	
50 vs. 10	1.774	0.684	0.992	No	
10 vs. 3100	1.760	0.679	0.984	No	
10 vs. 0.5	1.005	0.388	0.998	No	
10 vs. 20	1.005	0.388	0.992	No	
20 vs. 3100	0.755	0.317	0.985	No	
0.5 vs. 3100	0.755	0.317		0.939	No
20 vs. 0.5	0.000218	0.0000917		1.000	No

Elements in the plant tissues - Phosphate - Stems

Two Way Analysis of Variance
General Linear Model (No Interactions)
Dependent Variable: P stems
Normality Test (Shapiro-Wilk) Passed (P = 0.526)
Equal Variance Test: Failed (P < 0.050)

Source of Variation	DF	SS	MS	F	P
Exp	3	108.339	36.113	2.023	0.114
Cd conc	9	9502.978	1055.886	59.151	<0.001
Residual	127	2267.045	17.851		
Total	139	12379.652	89.062		

The difference in the mean values among the different levels of Exp is not great enough to exclude the possibility that the difference is just due to random sampling variability after allowing for the effects of differences in Cd conc. There is not a statistically significant difference (P = 0.114).

The difference in the mean values among the different levels of Cd conc is greater than would be expected by chance after allowing for effects of differences in Exp. There is a statistically significant difference (P = <0.001). To isolate which group(s) differ from the others use a multiple comparison procedure.

Power of performed test with alpha = 0.0500: for Exp : 0.264
Power of performed test with alpha = 0.0500: for Cd conc : 1.000

Least square means for Exp :

Group	Mean	SEM
272.000	17.931	0.729
273.000	16.070	0.668
278.000	16.018	0.834
280.000	17.737	0.687

Least square means for Cd conc :

Group	Mean	SEM
0.5	8.272	1.056
20	7.903	1.056
50	9.201	1.056
140	23.700	1.134
270	28.160	1.241
550	30.346	1.134
1270	23.132	1.130

3100	18.137	1.230
27500	10.903	1.056
10	9.636	1.353

All Pairwise Multiple Comparison Procedures (Holm-Sidak method):
Overall significance level = 0.05

Comparisons for factor: **Exp**

**Comparison**	**Diff of Means**	**t**	**P**	**P<0.050**
272.000 vs. 273.000	1.861	1.883	0.319	No
280.000 vs. 273.000	1.667	1.740	0.356	No
272.000 vs. 278.000	1.914	1.728	0.304	No
280.000 vs. 278.000	1.719	1.591	0.305	No
272.000 vs. 280.000	0.194	0.194	0.976	No
273.000 vs. 278.000	0.0528	0.0494	0.961	No

Comparisons for factor: **Cd conc**

**Comparison**	**Diff of Means**	**t**	**P**	**P<0.050**
550 vs. 20	22.443	14.485	<0.001	Yes
550 vs. 0.5	22.074	14.247	<0.001	Yes
550 vs. 50	21.145	13.647	<0.001	Yes
550 vs. 27500	19.442	12.548	<0.001	Yes
270 vs. 20	20.257	12.427	<0.001	Yes
270 vs. 0.5	19.888	12.201	<0.001	Yes
550 vs. 10	20.710	11.732	<0.001	Yes
270 vs. 50	18.959	11.631	<0.001	Yes
270 vs. 27500	17.257	10.587	<0.001	Yes
140 vs. 20	15.797	10.196	<0.001	Yes
270 vs. 10	18.524	10.087	<0.001	Yes
140 vs. 0.5	15.428	9.958	<0.001	Yes
1270 vs. 20	15.229	9.844	<0.001	Yes
1270 vs. 0.5	14.861	9.606	<0.001	Yes
140 vs. 50	14.499	9.358	<0.001	Yes
1270 vs. 50	13.931	9.005	<0.001	Yes
140 vs. 27500	12.797	8.259	<0.001	Yes
140 vs. 10	14.064	7.967	<0.001	Yes
1270 vs. 27500	12.229	7.905	<0.001	Yes
1270 vs. 10	13.496	7.654	<0.001	Yes
550 vs. 3100	12.208	7.297	<0.001	Yes
3100 vs. 20	10.234	6.311	<0.001	Yes
3100 vs. 0.5	9.866	6.084	<0.001	Yes
270 vs. 3100	10.022	5.734	<0.001	Yes
3100 vs. 50	8.937	5.511	<0.001	Yes
3100 vs. 10	8.501	4.648	<0.001	Yes
550 vs. 1270	7.213	4.506	<0.001	Yes
3100 vs. 27500	7.234	4.461	<0.001	Yes
550 vs. 140	6.646	4.146	0.001	Yes
140 vs. 3100	5.563	3.325	0.018	Yes
270 vs. 1270	5.028	2.994	0.048	Yes
1270 vs. 3100	4.995	2.989	0.046	Yes
270 vs. 140	4.460	2.653	0.111	No
27500 vs. 20	3.000	2.009	0.437	No
27500 vs. 0.5	2.632	1.762	0.603	No
550 vs. 270	2.186	1.300	0.887	No
27500 vs. 50	1.703	1.140	0.931	No
10 vs. 20	1.733	1.009	0.951	No
50 vs. 20	1.298	0.869	0.967	No
10 vs. 0.5	1.364	0.795	0.965	No
27500 vs. 10	1.267	0.738	0.955	No
50 vs. 0.5	0.929	0.622	0.953	No
140 vs. 1270	0.568	0.355	0.979	No

10 vs. 50	0.435	0.253	0.960	No
0.5 vs. 20	0.369	0.247	0.805	No

Elements in the plant tissues - Phosphate - Roots

Two Way Analysis of Variance
General Linear Model (No Interactions)
Dependent Variable: P roots
Normality Test (Shapiro-Wilk) Passed (P = 0.528)
Equal Variance Test: Passed (P = 1.000)

Source of Variation	DF	SS	MS	F	P
Exp	3	1501.617	500.539	1.165	0.342
Cd con	9	26269.776	2918.864	6.792	<0.001
Residual	26	11174.071	429.772		
Total	38	38956.399	1025.168		

The difference in the mean values among the different levels of Exp is not great enough to exclude the possibility that the difference is just due to random sampling variability after allowing for the effects of differences in Cd con. There is not a statistically significant difference (P = 0.342).

The difference in the mean values among the different levels of Cd con is greater than would be expected by chance after allowing for effects of differences in Exp. There is a statistically significant difference (P = <0.001). To isolate which group(s) differ from the others use a multiple comparison procedure.

Power of performed test with alpha = 0.0500: for Exp : 0.0752
Power of performed test with alpha = 0.0500: for Cd con : 0.998

Least square means for Exp :

Group	Mean	SEM
272.000	48.039	7.025
273.000	55.763	6.556
278.000	48.948	6.556
280.000	38.560	6.556

Least square means for Cd con :

Group	Mean	SEM
0.5	43.151	10.365
20	38.546	10.365
50	50.024	10.365
140	86.560	10.365
270	78.640	10.365
550	72.099	10.365
1270	26.978	10.365
3100	9.841	10.365
27500	10.489	10.365
10	61.950	12.134

All Pairwise Multiple Comparison Procedures (Holm-Sidak method):
Overall significance level = 0.05

Comparisons for factor: **Exp**

Comparison	Diff of Means	t	P	P<0.050
273.000 vs. 280.000	17.203	1.856	0.373	No
278.000 vs. 280.000	10.388	1.121	0.797	No
272.000 vs. 280.000	9.479	0.987	0.802	No
273.000 vs. 272.000	7.724	0.804	0.814	No
273.000 vs. 278.000	6.815	0.735	0.718	No

278.000 vs. 272.000	0.909	0.0946	0.925	No

Comparisons for factor: **Cd con**

Comparison	Diff of Means	t	P	P<0.050
140 vs. 3100	76.720	5.234	<0.001	Yes
140 vs. 27500	76.071	5.189	<0.001	Yes
270 vs. 3100	68.799	4.693	0.003	Yes
270 vs. 27500	68.151	4.649	0.004	Yes
550 vs. 3100	62.258	4.247	0.010	Yes
550 vs. 27500	61.610	4.203	0.011	Yes
140 vs. 1270	59.582	4.065	0.015	Yes
270 vs. 1270	51.662	3.524	0.059	No
140 vs. 20	48.015	3.275	0.105	No
10 vs. 3100	52.109	3.265	0.105	No
10 vs. 27500	51.461	3.225	0.112	No
550 vs. 1270	45.120	3.078	0.153	No
140 vs. 0.5	43.410	2.961	0.193	No
50 vs. 3100	40.183	2.741	0.296	No
270 vs. 20	40.094	2.735	0.292	No
50 vs. 27500	39.534	2.697	0.306	No
140 vs. 50	36.537	2.492	0.433	No
270 vs. 0.5	35.489	2.421	0.475	No
550 vs. 20	33.553	2.289	0.566	No
0.5 vs. 3100	33.310	2.272	0.566	No
0.5 vs. 27500	32.661	2.228	0.587	No
10 vs. 1270	34.971	2.191	0.601	No
550 vs. 0.5	28.948	1.975	0.753	No
20 vs. 3100	28.705	1.958	0.750	No
270 vs. 50	28.616	1.952	0.738	No
20 vs. 27500	28.056	1.914	0.749	No
50 vs. 1270	23.045	1.572	0.926	No
140 vs. 10	24.611	1.542	0.927	No
550 vs. 50	22.075	1.506	0.929	No
10 vs. 20	23.404	1.467	0.932	No
10 vs. 0.5	18.799	1.178	0.986	No
1270 vs. 3100	17.138	1.169	0.983	No
1270 vs. 27500	16.489	1.125	0.984	No
0.5 vs. 1270	16.172	1.103	0.981	No
270 vs. 10	16.690	1.046	0.982	No
140 vs. 550	14.461	0.987	0.983	No
20 vs. 1270	11.567	0.789	0.994	No
50 vs. 20	11.478	0.783	0.990	No
10 vs. 50	11.926	0.747	0.987	No
550 vs. 10	10.149	0.636	0.989	No
140 vs. 270	7.920	0.540	0.989	No
50 vs. 0.5	6.873	0.469	0.984	No
270 vs. 550	6.541	0.446	0.960	No
0.5 vs. 20	4.605	0.314	0.940	No
27500 vs. 3100	0.648	0.0442	0.965	No

Elements in the plant tissues - Phosphate - Seeds

Two Way Analysis of Variance
General Linear Model (No Interactions)
Dependent Variable: P seeds
Normality Test (Shapiro-Wilk) Passed (P = 0.613)
Equal Variance Test: Passed (P = 0.312)

Source of Variation	DF	SS	MS	F	P
Exp	3	74.014	24.671	22.623	<0.001

Cd conc	3	11.829	3.943	3.616	0.019
Residual	52	56.708	1.091		
Total	58	141.755	2.444		

The difference in the mean values among the different levels of Exp is greater than would be expected by chance after allowing for effects of differences in Cd conc. There is a statistically significant difference (P = <0.001). To isolate which group(s) differ from the others use a multiple comparison procedure.

The difference in the mean values among the different levels of Cd conc is greater than would be expected by chance after allowing for effects of differences in Exp. There is a statistically significant difference (P = 0.019). To isolate which group(s) differ from the others use a multiple comparison procedure.

Power of performed test with alpha = 0.0500: for Exp : 1.000
Power of performed test with alpha = 0.0500: for Cd conc : 0.604

Least square means for Exp :

**Group**	**Mean**	**SEM**
272.000	11.795	0.315
273.000	10.383	0.261
278.000	9.713	0.270
280.000	8.525	0.261

Least square means for Cd conc :

**Group**	**Mean**	**SEM**
0.5	10.097	0.261
20	10.539	0.261
50	9.395	0.261
10	10.384	0.327

All Pairwise Multiple Comparison Procedures (Holm-Sidak method):
Overall significance level = 0.05

Comparisons for factor: **Exp**

**Comparison**	**Diff of Means**	**t**	**P**	**P<0.050**
272.000 vs. 280.000	3.269	7.997	<0.001	Yes
273.000 vs. 280.000	1.857	5.031	<0.001	Yes
272.000 vs. 278.000	2.081	5.018	<0.001	Yes
272.000 vs. 273.000	1.412	3.453	0.003	Yes
278.000 vs. 280.000	1.188	3.162	0.005	Yes
273.000 vs. 278.000	0.669	1.781	0.081	No

Comparisons for factor: **Cd conc**

**Comparison**	**Diff of Means**	**t**	**P**	**P<0.050**
20 vs. 50	1.144	3.099	0.019	Yes
10 vs. 50	0.989	2.363	0.105	No
0.5 vs. 50	0.703	1.903	0.228	No
20 vs. 0.5	0.442	1.196	0.556	No
10 vs. 0.5	0.287	0.685	0.746	No
20 vs. 10	0.155	0.370	0.713	No

Elements in the plant tissues - Sulphate - Harvested leaves

Two Way Analysis of Variance
General Linear Model (No Interactions)
Dependent Variable: S harvest
Normality Test (Shapiro-Wilk) Failed (P < 0.050)
Equal Variance Test: Failed (P < 0.050)

**Source of Variation**	**DF**	**SS**	**MS**	**F**	**P**

Exp	3	357.617	119.206	4.335	0.006
Cd conc	9	3618.175	402.019	14.619	<0.001
Residual	14	3134.965	27.500		
Total	126	7071.513	56.123		

The difference in the mean values among the different levels of Exp is greater than would be expected by chance after allowing for effects of differences in Cd conc. There is a statistically significant difference (P = 0.006). To isolate which group(s) differ from the others use a multiple comparison procedure.

The difference in the mean values among the different levels of Cd conc is greater than would be expected by chance after allowing for effects of differences in Exp. There is a statistically significant difference (P = <0.001). To isolate which group(s) differ from the others use a multiple comparison procedure.

Power of performed test with alpha = 0.0500: for Exp : 0.748
Power of performed test with alpha = 0.0500: for Cd conc : 1.000

Least square means for Exp :

**Group**	**Mean**	**SEM**
272.000	6.965	0.960
273.000	8.202	0.841
278.000	6.803	1.165
280.000	11.026	0.892

Least square means for Cd conc :

**Group**	**Mean**	**SEM**
0.5	4.452	1.311
20	4.520	1.311
50	4.708	1.615
140	5.056	1.595
270	4.890	1.519
550	7.648	1.519
1270	14.542	1.460
3100	13.351	1.696
27500	19.239	1.355
10	4.085	1.606

All Pairwise Multiple Comparison Procedures (Holm-Sidak method):
Overall significance level = 0.05

Comparisons for factor: **Exp**

**Comparison**	**Diff of Means**	**t**	**P**	**P<0.050**
280.000 vs. 272.000	4.062	3.100	0.015	Yes
280.000 vs. 278.000	4.223	2.879	0.024	Yes
280.000 vs. 273.000	2.824	2.305	0.089	No
273.000 vs. 278.000	1.399	0.974	0.702	No
273.000 vs. 272.000	1.237	0.970	0.557	No
272.000 vs. 278.000	0.162	0.107	0.915	No

Comparisons for factor: **Cd conc**

**Comparison**	**Diff of Means**	**t**	**P**	**P<0.050**
27500 vs. 0.5	14.787	7.842	<0.001	Yes
27500 vs. 20	14.719	7.806	<0.001	Yes
27500 vs. 10	15.153	7.212	<0.001	Yes
27500 vs. 270	14.349	7.050	<0.001	Yes
27500 vs. 50	14.530	6.893	<0.001	Yes
27500 vs. 140	14.183	6.776	<0.001	Yes
27500 vs. 550	11.591	5.695	<0.001	Yes
1270 vs. 0.5	10.090	5.141	<0.001	Yes
1270 vs. 20	10.023	5.107	<0.001	Yes
1270 vs. 10	10.457	4.818	<0.001	Yes
1270 vs. 270	9.652	4.581	<0.001	Yes

1270 vs. 50	9.834	4.517	<0.001	Yes
1270 vs. 140	9.486	4.387	<0.001	Yes
3100 vs. 0.5	8.899	4.152	0.002	Yes
3100 vs. 20	8.832	4.121	0.002	Yes
3100 vs. 10	9.266	3.968	0.004	Yes
3100 vs. 270	8.461	3.717	0.009	Yes
3100 vs. 50	8.643	3.692	0.010	Yes
3100 vs. 140	8.296	3.564	0.014	Yes
1270 vs. 550	6.895	3.273	0.036	Yes
27500 vs. 3100	5.887	2.712	0.176	No
3100 vs. 550	5.704	2.506	0.280	No
27500 vs. 1270	4.697	2.357	0.373	No
550 vs. 10	3.562	1.612	0.923	No
550 vs. 0.5	3.195	1.593	0.921	No
550 vs. 20	3.128	1.559	0.925	No
550 vs. 50	2.939	1.326	0.981	No
550 vs. 270	2.757	1.284	0.983	No
550 vs. 140	2.592	1.177	0.991	No
1270 vs. 3100	1.191	0.532	1.000	No
140 vs. 10	0.970	0.429	1.000	No
270 vs. 10	0.805	0.364	1.000	No
140 vs. 0.5	0.604	0.292	1.000	No
50 vs. 10	0.623	0.274	1.000	No
140 vs. 20	0.536	0.260	1.000	No
270 vs. 0.5	0.438	0.218	1.000	No
20 vs. 10	0.434	0.209	1.000	No
270 vs. 20	0.371	0.185	1.000	No
0.5 vs. 10	0.367	0.177	1.000	No
140 vs. 50	0.348	0.153	1.000	No
50 vs. 0.5	0.256	0.123	1.000	No
50 vs. 20	0.189	0.0907	1.000	No
270 vs. 50	0.182	0.0821	1.000	No
140 vs. 270	0.166	0.0752	0.996	No
20 vs. 0.5	0.0675	0.0364	0.971	No

Elements in the plant tissues - Sulphate - w10 leaves

Two Way Analysis of Variance
General Linear Model (No Interactions)
Dependent Variable: S w10
Normality Test (Shapiro-Wilk) Failed (P < 0.050)
Equal Variance Test: Failed (P < 0.050)

Source of Variation	DF	SS	MS	F	P
Exp	3	1205.325	401.775	2.402	0.081
Cd conc	8	2685.454	335.682	2.007	0.068
Residual	43	7191.186	167.237		
Total	54	11015.552	203.992		

The difference in the mean values among the different levels of Exp is not great enough to exclude the possibility that the difference is just due to random sampling variability after allowing for the effects of differences in Cd conc. There is not a statistically significant difference (P = 0.081).

The difference in the mean values among the different levels of Cd conc is not great enough to exclude the possibility that the difference is just due to random sampling variability after allowing for the effects of differences in Exp. There is not a statistically significant difference (P = 0.068).

Power of performed test with alpha = 0.0500: for Exp : 0.337
Power of performed test with alpha = 0.0500: for Cd conc : 0.401

Least square means for Exp :

Group	Mean	SEM
272.000	7.449	5.050
273.000	8.201	3.399
278.000	4.434	3.175
280.000	16.502	3.391

Least square means for Cd conc :

Group	Mean	SEM
0.5	3.669	4.921
20	2.562	4.921
50	3.321	4.921
140	22.024	4.921
270	7.009	4.921
550	7.506	5.316
1270	16.649	5.316
10	2.227	5.443
3100	17.353	9.339

Elements in the plant tissues - Sulphate - w5 leaves

Two Way Analysis of Variance
General Linear Model (No Interactions)
Dependent Variable: S w5
Normality Test (Shapiro-Wilk) Failed (P < 0.050)
Equal Variance Test: Failed (P < 0.050)

Source of Variation	DF	SS	MS	F	P
Exp	3	9.191	3.064	1.756	0.165
Cd conc	9	129.587	14.399	8.255	<0.001
Residual	62	108.144	1.744		
Total	74	243.207	3.287		

The difference in the mean values among the different levels of Exp is not great enough to exclude the possibility that the difference is just due to random sampling variability after allowing for the effects of differences in Cd conc. There is not a statistically significant difference (P = 0.165).

The difference in the mean values among the different levels of Cd conc is greater than would be expected by chance after allowing for effects of differences in Exp. There is a statistically significant difference (P = <0.001). To isolate which group(s) differ from the others use a multiple comparison procedure.

Power of performed test with alpha = 0.0500: for Exp : 0.197
Power of performed test with alpha = 0.0500: for Cd conc : 1.000

Least square means for Exp :

Group	Mean	SEM
272.000	3.967	0.316
273.000	4.261	0.295
278.000	4.939	0.304
280.000	4.494	0.317

Least square means for Cd conc :

Group	Mean	SEM
0.5	3.293	0.467
20	3.419	0.467
50	3.619	0.467
140	3.880	0.467
270	3.784	0.467
550	4.045	0.467

1270	4.604	0.467
3100	5.569	0.467
27500	8.591	0.600
10	3.348	0.547

All Pairwise Multiple Comparison Procedures (Holm-Sidak method):
Overall significance level = 0.05

Comparisons for factor: **Exp**

**Comparison**	**Diff of Means**	**t**	**P**	**P<0.050**
278.000 vs. 272.000	0.972	2.213	0.170	No
278.000 vs. 273.000	0.678	1.599	0.457	No
280.000 vs. 272.000	0.527	1.176	0.673	No
278.000 vs. 280.000	0.445	1.011	0.680	No
273.000 vs. 272.000	0.294	0.679	0.750	No
280.000 vs. 273.000	0.234	0.539	0.592	No

Comparisons for factor: **Cd conc**

**Comparison**	**Diff of Means**	**t**	**P**	**P<0.050**
27500 vs. 0.5	5.298	6.971	<0.001	Yes
27500 vs. 20	5.172	6.805	<0.001	Yes
27500 vs. 50	4.972	6.541	<0.001	Yes
27500 vs. 10	5.243	6.461	<0.001	Yes
27500 vs. 270	4.808	6.325	<0.001	Yes
27500 vs. 140	4.711	6.199	<0.001	Yes
27500 vs. 550	4.547	5.982	<0.001	Yes
27500 vs. 1270	3.987	5.245	<0.001	Yes
27500 vs. 3100	3.022	3.976	0.007	Yes
3100 vs. 0.5	2.276	3.447	0.036	Yes
3100 vs. 20	2.150	3.256	0.062	No
3100 vs. 10	2.221	3.090	0.097	No
3100 vs. 50	1.950	2.953	0.137	No
3100 vs. 270	1.786	2.704	0.247	No
3100 vs. 140	1.689	2.558	0.333	No
3100 vs. 550	1.525	2.309	0.522	No
1270 vs. 0.5	1.311	1.986	0.784	No
1270 vs. 20	1.185	1.795	0.896	No
1270 vs. 10	1.256	1.747	0.910	No
1270 vs. 50	0.985	1.491	0.981	No
3100 vs. 1270	0.965	1.461	0.982	No
1270 vs. 270	0.821	1.243	0.997	No
550 vs. 0.5	0.752	1.138	0.999	No
1270 vs. 140	0.724	1.097	0.999	No
550 vs. 10	0.697	0.969	1.000	No
550 vs. 20	0.625	0.947	1.000	No
140 vs. 0.5	0.587	0.889	1.000	No
1270 vs. 550	0.560	0.847	1.000	No
270 vs. 0.5	0.491	0.743	1.000	No
140 vs. 10	0.532	0.740	1.000	No
140 vs. 20	0.461	0.698	1.000	No
550 vs. 50	0.425	0.644	1.000	No
270 vs. 10	0.436	0.606	1.000	No
270 vs. 20	0.365	0.552	1.000	No
50 vs. 0.5	0.327	0.495	1.000	No
550 vs. 270	0.261	0.395	1.000	No
140 vs. 50	0.260	0.394	1.000	No
50 vs. 10	0.272	0.378	1.000	No
50 vs. 20	0.200	0.303	1.000	No
550 vs. 140	0.165	0.250	1.000	No
270 vs. 50	0.164	0.249	1.000	No
20 vs. 0.5	0.126	0.191	0.999	No

140 vs. 270	0.0961	0.146	0.998	No
20 vs. 10	0.0714	0.0993	0.994	No
10 vs. 0.5	0.0549	0.0763	0.939	No

Elements in the plant tissues - Sulphate - Stems

Two Way Analysis of Variance
General Linear Model (No Interactions)
Dependent Variable: S stems
Normality Test (Shapiro-Wilk) Failed (P < 0.050)
Equal Variance Test: Failed (P < 0.050)

Source of Variation	DF	SS	MS	F	P
Exp	3	29.807	9.936	0.441	0.724
Cd conc	9	1201.659	133.518	5.922	<0.001
Residual	127	2863.113	22.544		
Total	139	4099.437	29.492		

The difference in the mean values among the different levels of Exp is not great enough to exclude the possibility that the difference is just due to random sampling variability after allowing for the effects of differences in Cd conc. There is not a statistically significant difference (P = 0.724).

The difference in the mean values among the different levels of Cd conc is greater than would be expected by chance after allowing for effects of differences in Exp. There is a statistically significant difference (P = <0.001). To isolate which group(s) differ from the others use a multiple comparison procedure.

Power of performed test with alpha = 0.0500: for Exp : 0.0500
Power of performed test with alpha = 0.0500: for Cd conc : 0.999

Least square means for Exp :

Group	Mean	SEM
272.000	6.622	0.819
273.000	7.174	0.751
278.000	5.793	0.937
280.000	6.604	0.772

Least square means for Cd conc :

Group	Mean	SEM
0.5	6.103	1.187
20	4.464	1.187
50	2.715	1.187
140	4.890	1.274
270	5.311	1.395
550	4.930	1.274
1270	10.209	1.270
3100	10.394	1.383
27500	11.596	1.187
10	4.870	1.521

All Pairwise Multiple Comparison Procedures (Holm-Sidak method):
Overall significance level = 0.05

Comparisons for factor: **Exp**

Comparison	Diff of Means	t	P	P<0.050
273.000 vs. 278.000	1.381	1.149	0.826	No
280.000 vs. 278.000	0.811	0.668	0.970	No
272.000 vs. 278.000	0.829	0.666	0.941	No
273.000 vs. 280.000	0.569	0.529	0.935	No
273.000 vs. 272.000	0.552	0.497	0.856	No
272.000 vs. 280.000	0.0176	0.0156	0.988	No

Comparisons for factor: **Cd conc**

**Comparison**	**Diff of Means**	**t**	**P**	**P<0.050**
27500 vs. 50	8.880	5.290	<0.001	Yes
1270 vs. 50	7.494	4.310	0.001	Yes
27500 vs. 20	7.131	4.248	0.002	Yes
3100 vs. 50	7.679	4.214	0.002	Yes
27500 vs. 140	6.706	3.851	0.008	Yes
27500 vs. 550	6.665	3.828	0.008	Yes
27500 vs. 10	6.726	3.486	0.026	Yes
27500 vs. 270	6.285	3.431	0.030	Yes
1270 vs. 20	5.745	3.304	0.045	Yes
27500 vs. 0.5	5.493	3.272	0.048	Yes
3100 vs. 20	5.930	3.254	0.050	Yes
1270 vs. 140	5.319	2.957	0.119	No
1270 vs. 550	5.279	2.934	0.123	No
3100 vs. 140	5.505	2.928	0.122	No
3100 vs. 550	5.464	2.906	0.126	No
1270 vs. 10	5.339	2.695	0.214	No
3100 vs. 10	5.525	2.688	0.211	No
1270 vs. 270	4.898	2.596	0.257	No
3100 vs. 270	5.084	2.588	0.254	No
1270 vs. 0.5	4.106	2.362	0.404	No
3100 vs. 0.5	4.292	2.355	0.397	No
0.5 vs. 50	3.387	2.018	0.675	No
270 vs. 50	2.596	1.417	0.981	No
550 vs. 50	2.215	1.272	0.994	No
140 vs. 50	2.174	1.249	0.994	No
10 vs. 50	2.154	1.117	0.998	No
20 vs. 50	1.749	1.042	0.999	No
0.5 vs. 20	1.638	0.976	0.999	No
27500 vs. 1270	1.387	0.798	1.000	No
0.5 vs. 140	1.213	0.697	1.000	No
0.5 vs. 550	1.172	0.673	1.000	No
27500 vs. 3100	1.201	0.659	1.000	No
0.5 vs. 10	1.233	0.639	1.000	No
270 vs. 20	0.846	0.462	1.000	No
0.5 vs. 270	0.792	0.432	1.000	No
550 vs. 20	0.466	0.268	1.000	No
140 vs. 20	0.425	0.244	1.000	No
270 vs. 140	0.421	0.223	1.000	No
270 vs. 10	0.441	0.214	1.000	No
10 vs. 20	0.405	0.210	1.000	No
270 vs. 550	0.380	0.201	1.000	No
3100 vs. 1270	0.185	0.0987	1.000	No
550 vs. 10	0.0607	0.0306	1.000	No
550 vs. 140	0.0409	0.0227	1.000	No
140 vs. 10	0.0199	0.0100	0.992	No

Elements in the plant tissues - Sulphate - Roots

Two Way Analysis of Variance
General Linear Model (No Interactions)
Dependent Variable: S roots
Normality Test (Shapiro-Wilk) Passed (P = 0.962)
Equal Variance Test: Passed (P = 1.000)

**Source of Variation**	**DF**	**SS**	**MS**	**F**	**P**
Exp	3	192.065	64.022	2.690	0.067
Cd conc	9	356.962	39.662	1.667	0.148

Residual	26	618.778	23.799
Total	38	1173.205	30.874

The difference in the mean values among the different levels of Exp is not great enough to exclude the possibility that the difference is just due to random sampling variability after allowing for the effects of differences in Cd conc. There is not a statistically significant difference (P = 0.067).

The difference in the mean values among the different levels of Cd conc is not great enough to exclude the possibility that the difference is just due to random sampling variability after allowing for the effects of differences in Exp. There is not a statistically significant difference (P = 0.148).

Power of performed test with alpha = 0.0500: for Exp : 0.381
Power of performed test with alpha = 0.0500: for Cd conc : 0.250

Least square means for Exp :

**Group**	**Mean**	**SEM**
272.000	10.893	1.653
273.000	8.798	1.543
278.000	14.724	1.543
280.000	12.762	1.543

Least square means for Cd conc :

**Group**	**Mean**	**SEM**
0.5	11.006	2.439
20	11.350	2.439
50	12.411	2.439
140	9.408	2.439
270	16.241	2.439
550	8.738	2.439
1270	16.959	2.439
3100	7.401	2.439
27500	10.138	2.439
10	14.292	2.855

Elements in the plant tissues - Sulphate - Seeds

Two Way Analysis of Variance
General Linear Model (No Interactions)
Dependent Variable: S seeds
Normality Test (Shapiro-Wilk) Failed (P < 0.050)
Equal Variance Test: Passed (P = 0.186)

**Source of Variation**	**DF**	**SS**	**MS**	**F**	**P**
Exp	3	1.528	0.509	10.465	<0.001
Cd conc	3	0.122	0.0408	0.838	0.479
Residual	52	2.530	0.0487		
Total	58	4.182	0.0721		

The difference in the mean values among the different levels of Exp is greater than would be expected by chance after allowing for effects of differences in Cd conc. There is a statistically significant difference (P = <0.001). To isolate which group(s) differ from the others use a multiple comparison procedure.

The difference in the mean values among the different levels of Cd conc is not great enough to exclude the possibility that the difference is just due to random sampling variability after allowing for the effects of differences in Exp. There is not a statistically significant difference (P = 0.479).

Power of performed test with alpha = 0.0500: for Exp : 0.997
Power of performed test with alpha = 0.0500: for Cd conc : 0.0500

Least square means for Exp :

Group	Mean	SEM
272.000	2.664	0.0665
273.000	2.504	0.0551
278.000	2.287	0.0571
280.000	2.692	0.0551

Least square means for Cd conc :

Group	Mean	SEM
0.5	2.606	0.0551
20	2.500	0.0551
50	2.498	0.0551
10	2.543	0.0691

All Pairwise Multiple Comparison Procedures (Holm-Sidak method):
Overall significance level = 0.05

Comparisons for factor: **Exp**

Comparison	Diff of Means	t	P	P<0.050
280.000 vs. 278.000	0.405	5.107	<0.001	Yes
272.000 vs. 278.000	0.377	4.302	<0.001	Yes
273.000 vs. 278.000	0.217	2.737	0.033	Yes
280.000 vs. 273.000	0.188	2.412	0.057	No
272.000 vs. 273.000	0.160	1.848	0.136	No
280.000 vs. 272.000	0.0285	0.330	0.743	No

Comparisons for factor: **Cd conc**

Comparison	Diff of Means	t	P	P<0.050
0.5 vs. 50	0.108	1.379	0.682	No
0.5 vs. 20	0.106	1.360	0.629	No
0.5 vs. 10	0.0629	0.711	0.927	No
10 vs. 50	0.0447	0.505	0.943	No
10 vs. 20	0.0431	0.488	0.861	No
20 vs. 50	0.00151	0.0194	0.985	No

---

4) Oxygen production and Chlorophyll accumulation

Oxygen production in week 5

Two Way Analysis of Variance
General Linear Model
Dependent Variable: oxygen w5
Normality Test (Shapiro-Wilk) Failed (P < 0.050)
Equal Variance Test: Failed (P < 0.050)

Source of Variation	DF	SS	MS	F	P
Light %	6	199.796	33.299	8.705	<0.001
Cd conc	9	50.083	5.565	1.455	0.185
Light % x Cd conc	54	42.564	0.788	0.206	1.000
Residual	63	240.983	3.825		
Total	132	556.115	4.213		

The difference in the mean values among the different levels of Light % is greater than would be expected by chance after allowing for effects of differences in Cd conc. There is a statistically significant difference (P = <0.001). To isolate which group(s) differ from the others use a multiple comparison procedure.

The difference in the mean values among the different levels of Cd conc is not great enough to exclude the possibility that the difference is just due to random sampling variability after allowing for the effects of differences

in Light %. There is not a statistically significant difference (P = 0.185).

The effect of different levels of Light % does not depend on what level of Cd conc is present. There is not a statistically significant interaction between Light % and Cd conc. (P = 1.000)

Power of performed test with alpha = 0.0500: for Light % : 1.000
Power of performed test with alpha = 0.0500: for Cd conc : 0.202
Power of performed test with alpha = 0.0500: for Light % x Cd conc : 0.0500

Least square means for Light % :

**Group**	**Mean**
1%	0.147
2,5%	0.654
5%	0.883
10%	1.511
25%	2.976
50%	3.427
100%	3.212

Std Err of LS Mean = 0.459

Least square means for Cd conc :

**Group**	**Mean**	**SEM**
0.5	2.809	0.523
10	2.488	0.523
20	2.252	0.523
50	1.075	0.523
140	2.465	0.523
270	1.975	0.523
550	1.138	0.523
1270	1.656	0.523
3100	1.303	0.523
27500	1.138	0.739

All Pairwise Multiple Comparison Procedures (Holm-Sidak method):
Overall significance level = 0.05

Comparisons for factor: **Light %**

**Comparison**	**Diff of Means**	**t**	**P**	**P<0.050**
50% vs. 1%	3.280	5.056	<0.001	Yes
100% vs. 1%	3.065	4.726	<0.001	Yes
25% vs. 1%	2.829	4.361	<0.001	Yes
50% vs. 2,5%	2.773	4.275	0.001	Yes
100% vs. 2,5%	2.559	3.944	0.003	Yes
50% vs. 5%	2.544	3.922	0.004	Yes
100% vs. 5%	2.330	3.592	0.010	Yes
25% vs. 2,5%	2.322	3.579	0.009	Yes
25% vs. 5%	2.093	3.227	0.026	Yes
50% vs. 10%	1.916	2.954	0.052	No
100% vs. 10%	1.702	2.623	0.114	No
25% vs. 10%	1.465	2.258	0.243	No
10% vs. 1%	1.364	2.103	0.304	No
10% vs. 2,5%	0.857	1.321	0.817	No
5% vs. 1%	0.736	1.134	0.880	No
10% vs. 5%	0.628	0.969	0.915	No
2,5% vs. 1%	0.507	0.782	0.944	No
50% vs. 25%	0.451	0.696	0.932	No
100% vs. 25%	0.237	0.365	0.977	No
5% vs. 2,5%	0.229	0.353	0.925	No
50% vs. 100%	0.214	0.331	0.742	No

Comparisons for factor: **Cd conc**

Comparison	Diff of Means	t	P	P<0.050
0.5 vs. 50	1.734	2.345	0.635	No
0.5 vs. 550	1.671	2.261	0.703	No
0.5 vs. 3100	1.506	2.038	0.867	No
10 vs. 50	1.413	1.911	0.927	No
140 vs. 50	1.390	1.880	0.936	No
0.5 vs. 27500	1.671	1.845	0.944	No
10 vs. 550	1.350	1.826	0.947	No
140 vs. 550	1.327	1.795	0.953	No
10 vs. 3100	1.185	1.603	0.989	No
20 vs. 50	1.177	1.592	0.988	No
140 vs. 3100	1.162	1.572	0.989	No
0.5 vs. 1270	1.153	1.560	0.989	No
20 vs. 550	1.114	1.507	0.992	No
10 vs. 27500	1.350	1.491	0.992	No
140 vs. 27500	1.327	1.465	0.993	No
20 vs. 3100	0.950	1.285	0.999	No
20 vs. 27500	1.114	1.230	0.999	No
270 vs. 50	0.900	1.217	0.999	No
270 vs. 550	0.837	1.132	1.000	No
0.5 vs. 270	0.834	1.129	1.000	No
10 vs. 1270	0.832	1.126	1.000	No
140 vs. 1270	0.809	1.094	1.000	No
270 vs. 27500	0.836	0.924	1.000	No
270 vs. 3100	0.672	0.909	1.000	No
20 vs. 1270	0.596	0.807	1.000	No
1270 vs. 50	0.581	0.786	1.000	No
0.5 vs. 20	0.557	0.753	1.000	No
1270 vs. 550	0.518	0.701	1.000	No
10 vs. 270	0.513	0.694	1.000	No
140 vs. 270	0.490	0.663	1.000	No
1270 vs. 27500	0.518	0.572	1.000	No
1270 vs. 3100	0.353	0.478	1.000	No
0.5 vs. 140	0.344	0.466	1.000	No
0.5 vs. 10	0.321	0.434	1.000	No
270 vs. 1270	0.319	0.431	1.000	No
20 vs. 270	0.277	0.375	1.000	No
10 vs. 20	0.236	0.319	1.000	No
3100 vs. 50	0.227	0.308	1.000	No
140 vs. 20	0.213	0.288	1.000	No
3100 vs. 550	0.165	0.223	1.000	No
3100 vs. 27500	0.164	0.182	1.000	No
550 vs. 50	0.0628	0.0849	1.000	No
27500 vs. 50	0.0632	0.0698	1.000	No
10 vs. 140	0.0230	0.0312	0.999	No
27500 vs. 550	0.000389	0.000429	1.000	No

Oxygen production in week 10

Two Way Analysis of Variance
Dependent Variable: oxygen w10
Normality Test (Shapiro-Wilk) Passed (P = 0.062)
Equal Variance Test: Failed (P < 0.050)

Source of Variation	DF	SS	MS	F	P
Light	6	44.499	7.416	23.490	<0.001
Cd conc	8	14.708	1.839	5.823	<0.001
Light x Cd conc	48	5.828	0.121	0.385	1.000
Residual	63	19.891	0.316		
Total	125	84.925	0.679		

The difference in the mean values among the different levels of Light is greater than would be expected by chance after allowing for effects of differences in Cd conc. There is a statistically significant difference (P = <0.001). To isolate which group(s) differ from the others use a multiple comparison procedure.

The difference in the mean values among the different levels of Cd conc is greater than would be expected by chance after allowing for effects of differences in Light. There is a statistically significant difference (P = <0.001). To isolate which group(s) differ from the others use a multiple comparison procedure.

The effect of different levels of Light does not depend on what level of Cd conc is present. There is not a statistically significant interaction between Light and Cd conc. (P = 1.000)

Power of performed test with alpha = 0.0500: for Light : 1.000
Power of performed test with alpha = 0.0500: for Cd conc : 0.997
Power of performed test with alpha = 0.0500: for Light x Cd conc : 0.0500

All Pairwise Multiple Comparison Procedures (Holm-Sidak method):
Overall significance level = 0.05

Comparisons for factor: **Light**

**Comparison**	**Diff of Means**	**t**	**P**	**P<0.050**
100% vs. 1%	1.752	9.352	<0.001	Yes
50% vs. 1%	1.539	8.217	<0.001	Yes
25% vs. 1%	1.450	7.743	<0.001	Yes
100% vs. 2.5%	1.318	7.035	<0.001	Yes
50% vs. 2.5%	1.105	5.900	<0.001	Yes
100% vs. 5%	1.061	5.663	<0.001	Yes
25% vs. 2.5%	1.016	5.425	<0.001	Yes
10% vs. 1%	1.012	5.403	<0.001	Yes
50% vs. 5%	0.848	4.528	<0.001	Yes
25% vs. 5%	0.759	4.054	0.002	Yes
100% vs. 10%	0.740	3.949	0.002	Yes
5% vs. 1%	0.691	3.689	0.005	Yes
10% vs. 2.5%	0.578	3.086	0.027	Yes
50% vs. 10%	0.527	2.814	0.051	No
25% vs. 10%	0.438	2.340	0.147	No
2.5% vs. 1%	0.434	2.317	0.134	No
10% vs. 5%	0.321	1.714	0.381	No
100% vs. 25%	0.301	1.610	0.380	No
5% vs. 2.5%	0.257	1.372	0.438	No
100% vs. 50%	0.213	1.135	0.453	No
50% vs. 25%	0.0889	0.474	0.637	No

Comparisons for factor: **Cd conc**

**Comparison**	**Diff of Means**	**t**	**P**	**P<0.050**
1270 vs. 3100	1.218	5.737	<0.001	Yes
1270 vs. 270	1.121	5.277	<0.001	Yes
20 vs. 3100	0.810	3.815	0.011	Yes
1270 vs. 0.5	0.723	3.406	0.037	Yes
1270 vs. 140	0.713	3.359	0.042	Yes
20 vs. 270	0.713	3.355	0.041	Yes
1270 vs. 10	0.711	3.347	0.041	Yes
50 vs. 3100	0.666	3.135	0.073	No
550 vs. 3100	0.644	3.030	0.095	No
1270 vs. 550	0.575	2.707	0.211	No
50 vs. 270	0.568	2.675	0.220	No
1270 vs. 50	0.553	2.602	0.252	No
550 vs. 270	0.546	2.570	0.261	No
10 vs. 3100	0.508	2.390	0.369	No
140 vs. 3100	0.505	2.378	0.365	No
0.5 vs. 3100	0.495	2.330	0.386	No

10 vs. 270	0.410	1.930	0.698	No
1270 vs. 20	0.408	1.922	0.686	No
140 vs. 270	0.407	1.918	0.669	No
0.5 vs. 270	0.397	1.870	0.687	No
20 vs. 0.5	0.315	1.485	0.915	No
20 vs. 140	0.305	1.437	0.921	No
20 vs. 10	0.303	1.425	0.912	No
50 vs. 0.5	0.171	0.804	0.999	No
20 vs. 550	0.167	0.785	0.999	No
50 vs. 140	0.161	0.757	0.999	No
50 vs. 10	0.158	0.745	0.998	No
550 vs. 0.5	0.149	0.700	0.998	No
20 vs. 50	0.144	0.680	0.996	No
550 vs. 140	0.138	0.652	0.994	No
550 vs. 10	0.136	0.640	0.988	No
270 vs. 3100	0.0977	0.460	0.995	No
50 vs. 550	0.0222	0.105	1.000	No
10 vs. 0.5	0.0127	0.0596	1.000	No
140 vs. 0.5	0.0102	0.0478	0.999	No
10 vs. 140	0.00250	0.0118	0.991	No

Chlorophyll *a* and pheophytin *a* in week 5

Two Way Analysis of Variance
General Linear Model (No Interactions)
Dependent Variable: Chl a week5
Normality Test (Shapiro-Wilk) Passed (P = 0.191)
Equal Variance Test: Failed (P < 0.050)

**Source of Variation**	**DF**	**SS**	**MS**	**F**	**P**
Exp	3	51.187	17.062	7.613	<0.001
Cd conc	9	672.048	74.672	33.319	<0.001
Residual	58	129.983	2.241		
Total	70	843.111	12.044		

The difference in the mean values among the different levels of Exp is greater than would be expected by chance after allowing for effects of differences in Cd conc. There is a statistically significant difference (P = <0.001). To isolate which group(s) differ from the others use a multiple comparison procedure.

The difference in the mean values among the different levels of Cd conc is greater than would be expected by chance after allowing for effects of differences in Exp. There is a statistically significant difference (P = <0.001). To isolate which group(s) differ from the others use a multiple comparison procedure.

Power of performed test with alpha = 0.0500: for Exp : 0.972
Power of performed test with alpha = 0.0500: for Cd conc : 1.000

Least square means for Exp :

**Group**	**Mean**	**SEM**
272.000	5.250	0.388
273.000	5.180	0.345
278.000	5.839	0.345
280.000	3.521	0.371

Least square means for Cd conc :

**Group**	**Mean**	**SEM**
0.5	9.778	0.529
10	8.703	0.621
20	7.793	0.529
50	7.812	0.529
140	3.447	0.529

270	1.324	0.529
550	2.631	0.529
1270	1.766	0.567
3100	3.789	0.681
27500	2.434	0.681

All Pairwise Multiple Comparison Procedures (Holm-Sidak method):
Overall significance level = 0.05

Comparisons for factor: **Exp**

**Comparison**	**Diff of Means**	**t**	**P**	**P<0.050**
278.000 vs. 280.000	2.317	4.571	<0.001	Yes
273.000 vs. 280.000	1.659	3.273	0.009	Yes
272.000 vs. 280.000	1.729	3.220	0.008	Yes
278.000 vs. 273.000	0.659	1.351	0.453	No
278.000 vs. 272.000	0.588	1.133	0.455	No
272.000 vs. 273.000	0.0705	0.136	0.892	No

Comparisons for factor: **Cd conc**

**Comparison**	**Diff of Means**	**t**	**P**	**P<0.050**
0.5 vs. 270	8.455	11.295	<0.001	Yes
0.5 vs. 1270	8.012	10.325	<0.001	Yes
0.5 vs. 550	7.147	9.548	<0.001	Yes
10 vs. 270	7.380	9.045	<0.001	Yes
50 vs. 270	6.488	8.668	<0.001	Yes
20 vs. 270	6.469	8.643	<0.001	Yes
0.5 vs. 27500	7.345	8.519	<0.001	Yes
0.5 vs. 140	6.331	8.458	<0.001	Yes
10 vs. 1270	6.937	8.247	<0.001	Yes
50 vs. 1270	6.046	7.791	<0.001	Yes
20 vs. 1270	6.027	7.766	<0.001	Yes
10 vs. 550	6.072	7.442	<0.001	Yes
0.5 vs. 3100	5.989	6.946	<0.001	Yes
50 vs. 550	5.180	6.920	<0.001	Yes
20 vs. 550	5.161	6.895	<0.001	Yes
10 vs. 27500	6.270	6.805	<0.001	Yes
10 vs. 140	5.256	6.442	<0.001	Yes
50 vs. 27500	5.378	6.238	<0.001	Yes
20 vs. 27500	5.359	6.216	<0.001	Yes
50 vs. 140	4.364	5.831	<0.001	Yes
20 vs. 140	4.345	5.805	<0.001	Yes
10 vs. 3100	4.914	5.334	<0.001	Yes
50 vs. 3100	4.023	4.665	<0.001	Yes
20 vs. 3100	4.004	4.643	<0.001	Yes
3100 vs. 270	2.465	2.859	0.117	No
140 vs. 270	2.124	2.837	0.118	No
0.5 vs. 20	1.986	2.653	0.178	No
0.5 vs. 50	1.967	2.628	0.180	No
3100 vs. 1270	2.023	2.282	0.363	No
140 vs. 1270	1.681	2.167	0.429	No
550 vs. 270	1.308	1.747	0.740	No
3100 vs. 27500	1.355	1.408	0.919	No
3100 vs. 550	1.157	1.342	0.930	No
0.5 vs. 10	1.075	1.318	0.924	No
27500 vs. 270	1.110	1.287	0.918	No
140 vs. 27500	1.014	1.176	0.939	No
10 vs. 20	0.911	1.116	0.940	No
550 vs. 1270	0.866	1.115	0.919	No
10 vs. 50	0.892	1.093	0.899	No
140 vs. 550	0.816	1.090	0.861	No
27500 vs. 1270	0.668	0.753	0.952	No

1270 vs. 270	0.442	0.570	0.966	No
3100 vs. 140	0.342	0.396	0.971	No
550 vs. 27500	0.198	0.230	0.967	No
50 vs. 20	0.0189	0.0252	0.980	No

Chlorophyll *b* and pheophytin *b* in week 5

Two Way Analysis of Variance
General Linear Model (No Interactions)
Dependent Variable: Chl b week 5
Normality Test (Shapiro-Wilk) Failed (P < 0.050)
Equal Variance Test: Failed (P < 0.050)

Source of Variation	DF	SS	MS	F	P
Exp	3	21.116	7.039	4.303	0.008
Cd conc	9	155.808	17.312	10.584	<0.001
Residua l	58	94.866	1.636		
Total	70	266.929	3.813		

The difference in the mean values among the different levels of Exp is greater than would be expected by chance after allowing for effects of differences in Cd conc. There is a statistically significant difference (P = 0.008). To isolate which group(s) differ from the others use a multiple comparison procedure.

The difference in the mean values among the different levels of Cd conc is greater than would be expected by chance after allowing for effects of differences in Exp. There is a statistically significant difference (P = <0.001). To isolate which group(s) differ from the others use a multiple comparison procedure.

Power of performed test with alpha = 0.0500: for Exp : 0.727
Power of performed test with alpha = 0.0500: for Cd conc : 1.000

Least square means for Exp :

Group	Mean	SEM
272.000	2.573	0.331
273.000	3.047	0.294
278.000	2.763	0.295
280.000	1.571	0.317

Least square means for Cd conc :

Group	Mean	SEM
0.5	4.844	0.452
10	4.161	0.530
20	3.948	0.452
50	3.840	0.452
140	1.600	0.452
270	0.598	0.452
550	1.239	0.452
1270	2.090	0.485
3100	1.625	0.582
27500	0.940	0.581

All Pairwise Multiple Comparison Procedures (Holm-Sidak method):
Overall significance level = 0.05

Comparisons for factor: **Exp**

Comparison	Diff of Means	t	P	P<0.050
273.000 vs. 280.000	1.475	3.408	0.007	Yes
278.000 vs. 280.000	1.192	2.752	0.039	Yes
272.000 vs. 280.000	1.002	2.183	0.126	No
273.000 vs. 272.000	0.474	1.068	0.642	No
273.000 vs. 278.000	0.283	0.680	0.749	No

278.000 vs. 272.000	0.190	0.429	0.670	No

Comparisons for factor: **Cd conc**

**Comparison**	**Diff of Means**	**t**	**P**	**P<0.050**
0.5 vs. 270	4.246	6.640	<0.001	Yes
0.5 vs. 550	3.604	5.637	<0.001	Yes
0.5 vs. 27500	3.904	5.301	<0.001	Yes
20 vs. 270	3.350	5.239	<0.001	Yes
10 vs. 270	3.562	5.111	<0.001	Yes
0.5 vs. 140	3.244	5.072	<0.001	Yes
50 vs. 270	3.242	5.070	<0.001	Yes
0.5 vs. 3100	3.219	4.369	0.002	Yes
20 vs. 550	2.709	4.236	0.003	Yes
10 vs. 550	2.921	4.191	0.003	Yes
0.5 vs. 1270	2.754	4.153	0.004	Yes
10 vs. 27500	3.221	4.092	0.005	Yes
20 vs. 27500	3.009	4.085	0.005	Yes
50 vs. 550	2.601	4.067	0.005	Yes
50 vs. 27500	2.901	3.938	0.007	Yes
10 vs. 140	2.560	3.673	0.016	Yes
20 vs. 140	2.348	3.672	0.015	Yes
50 vs. 140	2.240	3.503	0.025	Yes
10 vs. 3100	2.535	3.221	0.055	No
20 vs. 3100	2.323	3.153	0.064	No
50 vs. 3100	2.215	3.007	0.093	No
10 vs. 1270	2.070	2.881	0.125	No
20 vs. 1270	1.858	2.802	0.147	No
50 vs. 1270	1.750	2.639	0.210	No
1270 vs. 270	1.492	2.251	0.452	No
0.5 vs. 50	1.004	1.570	0.926	No
140 vs. 270	1.002	1.567	0.917	No
1270 vs. 27500	1.151	1.520	0.925	No
0.5 vs. 20	0.896	1.401	0.955	No
3100 vs. 270	1.027	1.394	0.948	No
1270 vs. 550	0.851	1.283	0.968	No
550 vs. 270	0.641	1.003	0.995	No
0.5 vs. 10	0.683	0.980	0.995	No
140 vs. 27500	0.661	0.897	0.996	No
3100 vs. 27500	0.686	0.834	0.997	No
1270 vs. 140	0.490	0.739	0.998	No
1270 vs. 3100	0.465	0.614	0.999	No
140 vs. 550	0.361	0.564	0.999	No
3100 vs. 550	0.386	0.524	0.998	No
27500 vs. 270	0.341	0.464	0.998	No
10 vs. 50	0.320	0.460	0.995	No
550 vs. 27500	0.300	0.407	0.990	No
10 vs. 20	0.212	0.305	0.986	No
20 vs. 50	0.108	0.169	0.982	No
3100 vs. 140	0.0251	0.0340	0.973	No

Chlorophyll *a* and pheophytin *a* in week 10

Two Way Analysis of Variance
General Linear Model (No Interactions)
Dependent Variable: Chl a week 10
Normality Test (Shapiro-Wilk) Passed (P = 0.057)
Equal Variance Test: Failed (P < 0.050)

**Source of Variation**	**DF**	**SS**	**MS**	**F**	**P**
Exp	3	49.869	16.623	5.325	0.003

Cd conc	9	97.703	10.856	3.478	0.002
Residual	48	149.831	3.121		
Total	60	301.642	5.027		

The difference in the mean values among the different levels of Exp is greater than would be expected by chance after allowing for effects of differences in Cd conc. There is a statistically significant difference (P = 0.003). To isolate which group(s) differ from the others use a multiple comparison procedure.

The difference in the mean values among the different levels of Cd conc is greater than would be expected by chance after allowing for effects of differences in Exp. There is a statistically significant difference (P = 0.002). To isolate which group(s) differ from the others use a multiple comparison procedure.

Power of performed test with alpha = 0.0500: for Exp : 0.846
Power of performed test with alpha = 0.0500: for Cd conc : 0.885

Least square means for Exp :

**Group**	**Mean**	**SEM**
272.000	4.233	0.552
273.000	2.007	0.474
278.000	2.036	0.470
280.000	3.438	0.498

Least square means for Cd conc :

**Group**	**Mean**	**SEM**
0.5	4.041	0.625
10	3.186	0.915
20	3.421	0.625
50	5.179	0.625
140	3.147	0.625
270	1.396	0.625
550	1.152	0.625
1270	2.630	0.726
3100	2.915	1.271
27500	2.216	1.814

All Pairwise Multiple Comparison Procedures (Holm-Sidak method):
Overall significance level = 0.05

Comparisons for factor: **Exp**

**Comparison**	**Diff of Means**	**t**	**P**	**P<0.050**
272.000 vs. 273.000	2.226	3.058	0.022	Yes
272.000 vs. 278.000	2.197	3.029	0.020	Yes
280.000 vs. 273.000	1.431	2.080	0.161	No
280.000 vs. 278.000	1.401	2.046	0.132	No
272.000 vs. 280.000	0.795	1.069	0.496	No
278.000 vs. 273.000	0.0292	0.0437	0.965	No

Comparisons for factor: **Cd conc**

**Comparison**	**Diff of Means**	**t**	**P**	**P<0.050**
50 vs. 550	4.027	4.558	0.002	Yes
50 vs. 270	3.783	4.283	0.004	Yes
0.5 vs. 550	2.889	3.270	0.082	No
0.5 vs. 270	2.646	2.995	0.167	No
50 vs. 1270	2.549	2.663	0.352	No
20 vs. 550	2.269	2.568	0.417	No
50 vs. 140	2.031	2.300	0.640	No
20 vs. 270	2.026	2.293	0.636	No
140 vs. 550	1.995	2.258	0.657	No
50 vs. 20	1.758	1.990	0.856	No
140 vs. 270	1.752	1.983	0.852	No
10 vs. 550	2.033	1.836	0.923	No

50 vs. 10	1.993	1.799	0.932	No
10 vs. 270	1.790	1.616	0.978	No
50 vs. 3100	2.264	1.599	0.978	No
50 vs. 27500	2.962	1.544	0.984	No
1270 vs. 550	1.477	1.543	0.982	No
0.5 vs. 1270	1.411	1.474	0.988	No
1270 vs. 270	1.234	1.289	0.998	No
50 vs. 0.5	1.138	1.288	0.997	No
3100 vs. 550	1.762	1.244	0.998	No
3100 vs. 270	1.519	1.073	1.000	No
0.5 vs. 140	0.894	1.012	1.000	No
0.5 vs. 27500	1.825	0.951	1.000	No
20 vs. 1270	0.792	0.827	1.000	No
0.5 vs. 3100	1.126	0.795	1.000	No
0.5 vs. 10	0.855	0.772	1.000	No
0.5 vs. 20	0.620	0.702	1.000	No
20 vs. 27500	1.205	0.628	1.000	No
27500 vs. 550	1.064	0.555	1.000	No
140 vs. 1270	0.518	0.541	1.000	No
140 vs. 27500	0.931	0.485	1.000	No
10 vs. 27500	0.969	0.477	1.000	No
10 vs. 1270	0.556	0.476	1.000	No
27500 vs. 270	0.821	0.428	1.000	No
20 vs. 3100	0.507	0.358	1.000	No
3100 vs. 27500	0.698	0.315	1.000	No
20 vs. 140	0.274	0.310	1.000	No
270 vs. 550	0.243	0.275	1.000	No
20 vs. 10	0.235	0.213	1.000	No
1270 vs. 27500	0.413	0.211	1.000	No
3100 vs. 1270	0.285	0.195	0.999	No
10 vs. 3100	0.271	0.173	0.997	No
140 vs. 3100	0.233	0.164	0.983	No
10 vs. 140	0.0384	0.0347	0.972	No

Chlorophyll *b* and pheophytin *b* in week 10

Two Way Analysis of Variance
General Linear Model (No Interactions)
Dependent Variable: Chl *b* week 10
Normality Test (Shapiro-Wilk) Failed (P < 0.050)
Equal Variance Test: Failed (P < 0.050)

**Source of Variation**	**DF**	**SS**	**MS**	**F**	**P**
Exp	3	12.103	4.034	4.036	0.012
Cd conc	9	18.700	2.078	2.079	0.050
Residual	48	47.979	1.000		
Total	60	79.992	1.333		

The difference in the mean values among the different levels of Exp is greater than would be expected by chance after allowing for effects of differences in Cd conc. There is a statistically significant difference (P = 0.012). To isolate which group(s) differ from the others use a multiple comparison procedure.

The difference in the mean values among the different levels of Cd conc is not great enough to exclude the possibility that the difference is just due to random sampling variability after allowing for the effects of differences in Exp. There is not a statistically significant difference (P = 0.050).

Power of performed test with alpha = 0.0500: for Exp : 0.677
Power of performed test with alpha = 0.0500: for Cd conc : 0.463

Least square means for Exp :

Group	Mean	SEM
272.000	2.088	0.312
273.000	0.992	0.268
278.000	0.920	0.266
280.000	1.530	0.282

Least square means for Cd conc :

Group	Mean	SEM
0.5	1.877	0.353
10	1.420	0.518
20	1.517	0.353
50	2.330	0.353
140	1.784	0.353
270	0.669	0.353
550	0.685	0.353
1270	1.223	0.411
3100	1.371	0.719
27500	0.949	1.027

All Pairwise Multiple Comparison Procedures (Holm-Sidak method):
Overall significance level = 0.05

Comparisons for factor: **Exp**

Comparison	Diff of Means	t	P	P<0.050
272.000 vs. 278.000	1.168	2.846	0.038	Yes
272.000 vs. 273.000	1.095	2.659	0.052	No
280.000 vs. 278.000	0.610	1.575	0.405	No
280.000 vs. 273.000	0.538	1.382	0.435	No
272.000 vs. 280.000	0.557	1.325	0.346	No
273.000 vs. 278.000	0.0725	0.192	0.849	No

Comparisons for factor: **Cd conc**

Comparison	Diff of Means	t	P	P<0.050
50 vs. 270	1.661	3.323	0.074	No
50 vs. 550	1.645	3.291	0.079	No
0.5 vs. 270	1.208	2.417	0.571	No
0.5 vs. 550	1.192	2.385	0.591	No
140 vs. 270	1.115	2.230	0.719	No
140 vs. 550	1.099	2.198	0.736	No
50 vs. 1270	1.107	2.043	0.844	No
20 vs. 270	0.848	1.697	0.979	No
20 vs. 550	0.832	1.665	0.982	No
50 vs. 20	0.813	1.626	0.985	No
50 vs. 10	0.910	1.452	0.997	No
50 vs. 27500	1.381	1.271	1.000	No
0.5 vs. 1270	0.654	1.207	1.000	No
10 vs. 270	0.751	1.198	1.000	No
50 vs. 3100	0.959	1.197	1.000	No
10 vs. 550	0.735	1.173	1.000	No
50 vs. 140	0.546	1.093	1.000	No
140 vs. 1270	0.561	1.035	1.000	No
1270 vs. 270	0.554	1.023	1.000	No
1270 vs. 550	0.538	0.994	1.000	No
50 vs. 0.5	0.453	0.906	1.000	No
3100 vs. 270	0.702	0.876	1.000	No
3100 vs. 550	0.686	0.856	1.000	No
0.5 vs. 27500	0.928	0.854	1.000	No
140 vs. 27500	0.834	0.768	1.000	No
0.5 vs. 10	0.457	0.729	1.000	No
0.5 vs. 20	0.360	0.720	1.000	No

0.5 vs. 3100	0.506	0.632	1.000	No
140 vs. 10	0.364	0.580	1.000	No
20 vs. 1270	0.294	0.543	1.000	No
140 vs. 20	0.267	0.533	1.000	No
20 vs. 27500	0.568	0.523	1.000	No
140 vs. 3100	0.413	0.515	1.000	No
10 vs. 27500	0.470	0.409	1.000	No
3100 vs. 27500	0.421	0.336	1.000	No
10 vs. 1270	0.197	0.298	1.000	No
27500 vs. 270	0.280	0.258	1.000	No
1270 vs. 27500	0.274	0.247	1.000	No
27500 vs. 550	0.265	0.244	1.000	No
0.5 vs. 140	0.0934	0.187	1.000	No
20 vs. 3100	0.146	0.183	1.000	No
3100 vs. 1270	0.148	0.178	1.000	No
20 vs. 10	0.0972	0.155	0.998	No
10 vs. 3100	0.0492	0.0555	0.998	No
550 vs. 270	0.0158	0.0316	0.975	No

Chlorophyll *a* and pheophytin *a* in harvested leaves

Two Way Analysis of Variance
General Linear Model (No Interactions)
Dependent Variable: Chl *a* harvest
Normality Test (Shapiro-Wilk) Failed (P < 0.050)
Equal Variance Test: Passed (P = 0.113)

**Source of Variation**	**DF**	**SS**	**MS**	**F**	**P**
Exp	2	0.413	0.206	0.125	0.883
Cd conc	8	30.950	3.869	2.337	0.034
Residual	46	76.140	1.655		
Total	56	108.691	1.941		

The difference in the mean values among the different levels of Exp is not great enough to exclude the possibility that the difference is just due to random sampling variability after allowing for the effects of differences in Cd conc. There is not a statistically significant difference (P = 0.883).

The difference in the mean values among the different levels of Cd conc is greater than would be expected by chance after allowing for effects of differences in Exp. There is a statistically significant difference (P = 0.034). To isolate which group(s) differ from the others use a multiple comparison procedure.

Power of performed test with alpha = 0.0500: for Exp : 0.0500
Power of performed test with alpha = 0.0500: for Cd conc : 0.536

Least square means for Exp :

**Group**	**Mean**	**SEM**
272.000	2.076	0.343
278.000	2.160	0.326
280.000	1.945	0.293

Least square means for Cd conc :

**Group**	**Mean**	**SEM**
0.5	2.778	0.432
10	3.332	0.546
20	2.146	0.432
50	3.052	0.463
140	1.816	0.488
270	0.876	0.591
550	1.345	0.531
1270	2.329	0.592

3100	0.869	0.920

All Pairwise Multiple Comparison Procedures (Holm-Sidak method):
Overall significance level = 0.05

Comparisons for factor: **Exp**

**Comparison**	**Diff of Means**	**t**	**P**	**P<0.050**
278.000 vs. 280.000	0.214	0.489	0.948	No
272.000 vs. 280.000	0.130	0.289	0.949	No
278.000 vs. 272.000	0.0838	0.177	0.860	No

Comparisons for factor: **Cd conc**

**Comparison**	**Diff of Means**	**t**	**P**	**P<0.050**
10 vs. 270	2.456	3.052	0.127	No
50 vs. 270	2.176	2.900	0.182	No
10 vs. 550	1.987	2.610	0.341	No
0.5 vs. 270	1.902	2.599	0.340	No
50 vs. 550	1.707	2.425	0.464	No
10 vs. 3100	2.463	2.303	0.556	No
50 vs. 3100	2.182	2.120	0.701	No
0.5 vs. 550	1.433	2.095	0.709	No
10 vs. 140	1.516	2.071	0.717	No
0.5 vs. 3100	1.909	1.879	0.844	No
50 vs. 140	1.236	1.839	0.858	No
1270 vs. 270	1.453	1.737	0.903	No
20 vs. 270	1.270	1.736	0.894	No
10 vs. 20	1.186	1.704	0.900	No
0.5 vs. 140	0.962	1.477	0.969	No
50 vs. 20	0.906	1.432	0.974	No
1270 vs. 3100	1.460	1.335	0.985	No
20 vs. 3100	1.277	1.257	0.990	No
10 vs. 1270	1.003	1.245	0.988	No
1270 vs. 550	0.984	1.238	0.986	No
140 vs. 270	0.940	1.227	0.983	No
20 vs. 550	0.801	1.171	0.986	No
0.5 vs. 20	0.632	1.035	0.994	No
50 vs. 1270	0.723	0.962	0.996	No
140 vs. 3100	0.946	0.909	0.996	No
10 vs. 0.5	0.554	0.796	0.998	No
1270 vs. 140	0.513	0.669	0.999	No
140 vs. 550	0.471	0.653	0.999	No
0.5 vs. 1270	0.449	0.613	0.998	No
550 vs. 270	0.469	0.591	0.997	No
20 vs. 140	0.330	0.507	0.997	No
550 vs. 3100	0.476	0.448	0.995	No
50 vs. 0.5	0.274	0.433	0.988	No
10 vs. 50	0.280	0.391	0.972	No
1270 vs. 20	0.183	0.250	0.962	No
270 vs. 3100	0.00646	0.00591	0.995	No

Chlorophyll *b* and pheophytin *b* in harvested leaves

Two Way Analysis of Variance
General Linear Model (No Interactions)
Dependent Variable: Chl *b* harvest
Normality Test (Shapiro-Wilk) Failed (P < 0.050)
Equal Variance Test: Passed (P = 0.393)

**Source of Variation**	**DF**	**SS**	**MS**	**F**	**P**
Exp	2	0.211	0.105	0.272	0.763

Cd conc	8	5.591	0.699	1.799	0.102
Residual	46	17.866	0.388		
Total	56	23.804	0.425		

The difference in the mean values among the different levels of Exp is not great enough to exclude the possibility that the difference is just due to random sampling variability after allowing for the effects of differences in Cd conc. There is not a statistically significant difference (P = 0.763).

The difference in the mean values among the different levels of Cd conc is not great enough to exclude the possibility that the difference is just due to random sampling variability after allowing for the effects of differences in Exp. There is not a statistically significant difference (P = 0.102).

Power of performed test with alpha = 0.0500: for Exp : 0.0500
Power of performed test with alpha = 0.0500: for Cd conc : 0.320

Least square means for Exp :

**Group**	**Mean**	**SEM**
272.000	0.894	0.166
278.000	1.005	0.158
280.000	0.854	0.142

Least square means for Cd conc :

**Group**	**Mean**	**SEM**
0.5	1.189	0.209
10	1.475	0.265
20	0.892	0.209
50	1.336	0.224
140	0.900	0.236
270	0.397	0.286
550	0.641	0.257
1270	1.063	0.287
3100	0.366	0.445

5) Fluorescence parameters - F_v/F_m

Three Way Analysis of Variance
General Linear Model
Dependent Variable: Fv/Fm
Normality Test (Shapiro-Wilk) Failed (P < 0.050)
Equal Variance Test: Failed (P < 0.050)

**Source of Variation**	**DF**	**SS**	**MS**	**F**	**P**
Cd conc	8	0.415	0.0518	10.578	<0.001
tissue	1	0.164	0.164	33.442	<0.001
week	1	0.0230	0.0230	4.694	0.031
Cd conc x tissue	8	0.0167	0.00209	0.426	0.906
Cd conc x week	8	0.768	0.0960	19.607	<0.001
tissue x week	1	0.00546	0.00546	1.115	0.291
Cd conc x tissue x week	8	0.0272	0.00340	0.695	0.696
Residual	662	3.243	0.00490		
Total	697	4.685	0.00672		

The main effects for Cd conc cannot be properly interpreted since the size of the factor's effect depends upon the level of another factor.

The difference in the mean values among the different levels of tissue are greater than would be expected by chance after allowing for the effects of differences in Cd conc and week. There is a statistically significant difference (P = <0.001). To isolate which group(s) differ from the others use a multiple comparison procedure.

The main effects for week cannot be properly interpreted since the size of the factor's effect depends upon the level of another factor.

The effect of different levels of Cd conc does not depend on what level of tissue is present. There is not a statistically significant interaction between Cd conc and tissue. (P = 0.906)

The effect of different levels of Cd conc depends on what level of week is present. There is a statistically significant interaction between Cd conc and week. (P = <0.001)

The effect of different levels of tissue does not depend on what level of week is present. There is not a statistically significant interaction between tissue and week. (P = 0.291)

All Pairwise Multiple Comparison Procedures (Holm-Sidak method):
Overall significance level = 0.05

Comparisons for factor: **Cd conc**

**Comparison**	**Diff of Means**	**t**	**P**	**P<0.050**
550 vs. 270	0.0751	6.956	<0.001	Yes
10 vs. 270	0.0796	6.759	<0.001	Yes
50 vs. 270	0.0715	6.619	<0.001	Yes
1270 vs. 270	0.0663	6.137	<0.001	Yes
0.5 vs. 270	0.0618	5.722	<0.001	Yes
3100 vs. 270	0.0585	4.524	<0.001	Yes
10 vs. 140	0.0453	3.853	0.004	Yes
550 vs. 140	0.0408	3.787	0.005	Yes
20 vs. 270	0.0404	3.738	0.006	Yes
50 vs. 140	0.0372	3.450	0.016	Yes
10 vs. 20	0.0392	3.331	0.023	Yes
550 vs. 20	0.0347	3.217	0.033	Yes
140 vs. 270	0.0343	3.188	0.035	Yes
1270 vs. 140	0.0319	2.966	0.070	No
50 vs. 20	0.0311	2.881	0.086	No
0.5 vs. 140	0.0275	2.550	0.207	No
1270 vs. 20	0.0259	2.398	0.287	No
0.5 vs. 20	0.0214	1.984	0.605	No
3100 vs. 140	0.0242	1.873	0.681	No
10 vs. 3100	0.0211	1.534	0.898	No
10 vs. 0.5	0.0178	1.512	0.894	No
3100 vs. 20	0.0181	1.402	0.929	No
550 vs. 3100	0.0166	1.285	0.955	No
550 vs. 0.5	0.0133	1.234	0.959	No
10 vs. 1270	0.0133	1.132	0.972	No
50 vs. 3100	0.0130	1.005	0.985	No
50 vs. 0.5	0.00970	0.898	0.990	No
550 vs. 1270	0.00885	0.819	0.992	No
10 vs. 50	0.00811	0.689	0.996	No
1270 vs. 3100	0.00777	0.601	0.996	No
20 vs. 140	0.00604	0.561	0.994	No
50 vs. 1270	0.00522	0.483	0.993	No
1270 vs. 0.5	0.00448	0.415	0.989	No
10 vs. 550	0.00448	0.380	0.974	No
550 vs. 50	0.00363	0.336	0.931	No
0.5 vs. 3100	0.00329	0.255	0.799	No

Comparisons for factor: **tissue**

**Comparison**	**Diff of Means**	**t**	**P**	**P<0.050**
vein vs. mesophyll	0.0314	5.783	<0.001	Yes

Comparisons for factor: **week**

**Comparison**	**Diff of Means**	**t**	**P**	**P<0.050**
w5 vs. w10	0.0118	2.167	0.031	Yes

Comparisons for factor: **week within 0.5**

Comparison	Diff of Means	t	P	P<0.05
w5 vs. w10	0.000467	0.0306	0.976	No

Comparisons for factor: **week within 10**

Comparison	Diff of Means	t	P	P<0.05
w5 vs. w10	0.0204	1.137	0.256	No

Comparisons for factor: **week within 20**

Comparison	Diff of Means	t	P	P<0.05
w5 vs. w10	0.0516	3.379	<0.001	Yes

Comparisons for factor: **week within 50**

Comparison	Diff of Means	t	P	P<0.05
w5 vs. w10	0.0542	3.551	<0.001	Yes

Comparisons for factor: **week within 140**

Comparison	Diff of Means	t	P	P<0.05
w5 vs. w10	0.0801	5.273	<0.001	Yes

Comparisons for factor: **week within 270**

Comparison	Diff of Means	t	P	P<0.05
w5 vs. w10	0.158	10.375	<0.001	Yes

Comparisons for factor: **week within 550**

Comparison	Diff of Means	t	P	P<0.05
w5 vs. w10	0.00701	0.459	0.646	No

Comparisons for factor: **week within 1270**

Comparison	Diff of Means	t	P	P<0.05
w5 vs. w10	0.0209	1.368	0.172	No

Comparisons for factor: **week within 3100**

Comparison	Diff of Means	t	P	P<0.05
w5 vs. w10	0.00789	0.378	0.705	No

Comparisons for factor: **Cd conc within w5**

Comparison	Diff of Means	t	P	P<0.05
270 vs. 20	0.0647	4.233	<0.001	Yes
550 vs. 20	0.0641	4.194	0.001	Yes
140 vs. 20	0.0598	3.938	0.003	Yes
10 vs. 20	0.0548	3.308	0.032	Yes
0.5 vs. 20	0.0470	3.077	0.067	No
3100 vs. 20	0.0479	2.862	0.126	No
1270 vs. 20	0.0413	2.701	0.192	No
270 vs. 50	0.0349	2.282	0.488	No
550 vs. 50	0.0342	2.243	0.511	No
140 vs. 50	0.0300	1.975	0.740	No
50 vs. 20	0.0298	1.951	0.747	No
270 vs. 1270	0.0234	1.532	0.966	No
10 vs. 50	0.0250	1.510	0.966	No
550 vs. 1270	0.0228	1.493	0.965	No
140 vs. 1270	0.0185	1.221	0.996	No
270 vs. 0.5	0.0177	1.157	0.997	No
0.5 vs. 50	0.0172	1.125	0.998	No
550 vs. 0.5	0.0171	1.117	0.997	No
3100 vs. 50	0.0181	1.080	0.997	No
270 vs. 3100	0.0168	1.003	0.998	No
550 vs. 3100	0.0162	0.967	0.998	No
140 vs. 0.5	0.0128	0.844	1.000	No

10 vs. 1270	0.0136	0.819	0.999	No
1270 vs. 50	0.0115	0.750	1.000	No
140 vs. 3100	0.0119	0.716	1.000	No
270 vs. 10	0.00982	0.592	1.000	No
550 vs. 10	0.00922	0.556	1.000	No
10 vs. 0.5	0.00785	0.473	1.000	No
3100 vs. 1270	0.00662	0.396	1.000	No
10 vs. 3100	0.00696	0.388	1.000	No
0.5 vs. 1270	0.00573	0.375	0.999	No
270 vs. 140	0.00486	0.320	0.999	No
140 vs. 10	0.00496	0.301	0.997	No
550 vs. 140	0.00425	0.280	0.989	No
3100 vs. 0.5	0.000886	0.0530	0.998	No
270 vs. 550	0.000602	0.0394	0.969	No

Comparisons for factor: **Cd conc within w10**

**Comparison**	**Diff of Means**	**t**	**P**	**P<0.05**
50 vs. 270	0.178	11.643	<0.001	Yes
1270 vs. 270	0.156	10.210	<0.001	Yes
10 vs. 270	0.169	10.102	<0.001	Yes
550 vs. 270	0.151	9.876	<0.001	Yes
20 vs. 270	0.145	9.520	<0.001	Yes
0.5 vs. 270	0.141	9.248	<0.001	Yes
50 vs. 140	0.104	6.829	<0.001	Yes
3100 vs. 270	0.134	6.785	<0.001	Yes
10 vs. 140	0.0955	5.708	<0.001	Yes
1270 vs. 140	0.0824	5.396	<0.001	Yes
550 vs. 140	0.0773	5.062	<0.001	Yes
140 vs. 270	0.0735	4.814	<0.001	Yes
20 vs. 140	0.0719	4.706	<0.001	Yes
0.5 vs. 140	0.0677	4.435	<0.001	Yes
3100 vs. 140	0.0603	3.056	0.050	No
50 vs. 0.5	0.0366	2.395	0.301	No
50 vs. 3100	0.0441	2.234	0.407	No
50 vs. 20	0.0324	2.123	0.483	No
50 vs. 550	0.0270	1.767	0.767	No
10 vs. 3100	0.0352	1.689	0.805	No
10 vs. 0.5	0.0278	1.659	0.806	No
50 vs. 1270	0.0219	1.433	0.916	No
10 vs. 20	0.0236	1.411	0.911	No
1270 vs. 3100	0.0222	1.124	0.981	No
10 vs. 550	0.0182	1.086	0.980	No
1270 vs. 0.5	0.0147	0.962	0.989	No
550 vs. 3100	0.0171	0.865	0.993	No
10 vs. 1270	0.0131	0.781	0.994	No
1270 vs. 20	0.0105	0.690	0.995	No
550 vs. 0.5	0.00959	0.628	0.995	No
20 vs. 3100	0.0116	0.589	0.992	No
50 vs. 10	0.00881	0.527	0.990	No
0.5 vs. 3100	0.00747	0.379	0.992	No
550 vs. 20	0.00544	0.356	0.978	No
1270 vs. 550	0.00510	0.334	0.932	No
20 vs. 0.5	0.00415	0.272	0.786	No

Power of performed test with alpha = 0.0500: for Cd conc : 1.000
Power of performed test with alpha = 0.0500: for tissue : 1.000
Power of performed test with alpha = 0.0500: for week : 0.472
Power of performed test with alpha = 0.0500: for Cd conc x tissue : 0.0500
Power of performed test with alpha = 0.0500: for Cd conc x week : 1.000
Power of performed test with alpha = 0.0500: for tissue x week : 0.0597

Least square means for Cd conc :

Group	Mean	SEM
0.5	0.791	0.00764
10	0.809	0.00896
20	0.769	0.00764
50	0.801	0.00764
140	0.763	0.00759
270	0.729	0.00764
550	0.804	0.00764
1270	0.795	0.00764
3100	0.788	0.0104

Least square means for tissue:

Group	Mean	SEM
mesophyll	0.768	0.00383
vein	0.799	0.00384

Least square means for week:

Group	Mean	SEM
w5	0.789	0.00374
w10	0.777	0.00393

Fluorescence parameters - Saturation - Mesophyll

Two Way Analysis of Variance
General Linear Model (No Interactions)
Dependent Variable: Saturation
Normality Test (Shapiro-Wilk) Passed (P = 0.059)
Equal Variance Test: Failed (P < 0.050)

Source of Variation	DF	SS	MS	F	P
Cd conc	9	11.690	1.299	92.378	<0.001
week	1	0.00796	0.00796	0.566	0.452
Residual	357	5.020	0.0141		
Total	367	16.714	0.0455		

The difference in the mean values among the different levels of Cd conc is greater than would be expected by chance after allowing for effects of differences in week. There is a statistically significant difference (P = <0.001). To isolate which group(s) differ from the others use a multiple comparison procedure.

The difference in the mean values among the different levels of week is not great enough to exclude the possibility that the difference is just due to random sampling variability after allowing for the effects of differences in Cd conc. There is not a statistically significant difference (P = 0.452).

Power of performed test with alpha = 0.0500: for Cd conc : 1.000
Power of performed test with alpha = 0.0500: for week : 0.0500

Least square means for Cd conc :

Group	Mean	SEM
0.5	0.330	0.0183
10	0.278	0.0213
20	0.239	0.0183
50	0.276	0.0183
140	0.625	0.0181
270	0.686	0.0183
550	0.578	0.0183
1270	0.645	0.0183
3100	0.669	0.0243
27500	0.590	0.0287

Least square means for week :

Group	Mean	SEM
w5	0.487	0.00849
w10	0.496	0.00965

All Pairwise Multiple Comparison Procedures (Holm-Sidak method):
Overall significance level = 0.05

Comparisons for factor: **Cd conc**

Comparison	Diff of Means	t	P	P<0.050
270 vs. 20	0.447	17.273	<0.001	Yes
270 vs. 50	0.411	15.868	<0.001	Yes
1270 vs. 20	0.406	15.692	<0.001	Yes
140 vs. 20	0.386	14.993	<0.001	Yes
270 vs. 10	0.409	14.553	<0.001	Yes
1270 vs. 50	0.370	14.288	<0.001	Yes
3100 vs. 20	0.430	14.136	<0.001	Yes
270 vs. 0.5	0.357	13.781	<0.001	Yes
140 vs. 50	0.349	13.580	<0.001	Yes
1270 vs. 10	0.368	13.096	<0.001	Yes
550 vs. 20	0.338	13.074	<0.001	Yes
3100 vs. 50	0.393	12.940	<0.001	Yes
140 vs. 10	0.347	12.433	<0.001	Yes
1270 vs. 0.5	0.316	12.200	<0.001	Yes
3100 vs. 10	0.391	12.118	<0.001	Yes
550 vs. 50	0.302	11.669	<0.001	Yes
140 vs. 0.5	0.295	11.481	<0.001	Yes
3100 vs. 0.5	0.339	11.162	<0.001	Yes
550 vs. 10	0.300	10.683	<0.001	Yes
27500 vs. 20	0.351	10.308	<0.001	Yes
550 vs. 0.5	0.248	9.582	<0.001	Yes
27500 vs. 50	0.314	9.239	<0.001	Yes
27500 vs. 10	0.312	8.742	<0.001	Yes
27500 vs. 0.5	0.260	7.651	<0.001	Yes
270 vs. 550	0.109	4.199	<0.001	Yes
0.5 vs. 20	0.0904	3.492	0.011	Yes
3100 vs. 550	0.0912	3.002	0.053	No
270 vs. 27500	0.0964	2.836	0.083	No
1270 vs. 550	0.0678	2.619	0.145	No
270 vs. 140	0.0612	2.381	0.250	No
3100 vs. 27500	0.0790	2.104	0.424	No
0.5 vs. 50	0.0540	2.087	0.415	No
0.5 vs. 10	0.0520	1.853	0.581	No
140 vs. 550	0.0474	1.843	0.560	No
1270 vs. 27500	0.0555	1.633	0.699	No
270 vs. 1270	0.0409	1.580	0.705	No
3100 vs. 140	0.0438	1.448	0.765	No
50 vs. 20	0.0363	1.404	0.755	No
10 vs. 20	0.0383	1.365	0.736	No
140 vs. 27500	0.0352	1.038	0.882	No
1270 vs. 140	0.0204	0.791	0.939	No
3100 vs. 1270	0.0235	0.772	0.902	No
270 vs. 3100	0.0174	0.574	0.919	No
27500 vs. 550	0.0122	0.359	0.921	No
10 vs. 50	0.00199	0.0708	0.944	No

Comparisons for factor: **week**

Comparison	Diff of Means	t	P	P<0.050
w10 vs. w5	0.00956	0.744	0.458	No

Fluorescence parameters - Saturation - Veins

Two Way Analysis of Variance
General Linear Model (No Interactions)
Dependent Variable: Sat vein
Normality Test (Shapiro-Wilk) Passed (P = 0.291)
Equal Variance Test: Failed (P < 0.050)

Source of Variation	DF	SS	MS	F	P
Cd conc	9	9.715	1.079	86.471	<0.001
week	1	0.156	0.156	12.530	<0.001
Residual	355	4.431	0.0125		
Total	365	14.225	0.0390		

The difference in the mean values among the different levels of Cd conc is greater than would be expected by chance after allowing for effects of differences in week. There is a statistically significant difference (P = <0.001). To isolate which group(s) differ from the others use a multiple comparison procedure.

The difference in the mean values among the different levels of week is greater than would be expected by chance after allowing for effects of differences in Cd conc. There is a statistically significant difference (P = <0.001). To isolate which group(s) differ from the others use a multiple comparison procedure.

Power of performed test with alpha = 0.0500: for Cd conc : 1.000
Power of performed test with alpha = 0.0500: for week : 0.942

Least square means for Cd conc :

Group	Mean	SEM
0.5	0.256	0.0172
10	0.205	0.0204
20	0.196	0.0172
50	0.213	0.0172
140	0.515	0.0172
270	0.618	0.0172
550	0.476	0.0172
1270	0.543	0.0172
3100	0.575	0.0229
27500	0.476	0.0270

Least square means for week :

Group	Mean	SEM
w5	0.386	0.00804
w10	0.429	0.00909

All Pairwise Multiple Comparison Procedures (Holm-Sidak method):
Overall significance level = 0.05

Comparisons for factor: **Cd conc**

Comparison	Diff of Means	t	P	P<0.050
270 vs. 20	0.422	17.289	<0.001	Yes
270 vs. 50	0.405	16.621	<0.001	Yes
270 vs. 10	0.413	15.464	<0.001	Yes
270 vs. 0.5	0.362	14.837	<0.001	Yes
1270 vs. 20	0.347	14.241	<0.001	Yes
1270 vs. 50	0.331	13.573	<0.001	Yes
3100 vs. 20	0.378	13.219	<0.001	Yes
140 vs. 20	0.319	13.088	<0.001	Yes
1270 vs. 10	0.339	12.682	<0.001	Yes
3100 vs. 50	0.362	12.650	<0.001	Yes
140 vs. 50	0.303	12.420	<0.001	Yes
3100 vs. 10	0.370	12.076	<0.001	Yes
1270 vs. 0.5	0.287	11.789	<0.001	Yes

140 vs. 10	0.311	11.629	<0.001	Yes
550 vs. 20	0.280	11.496	<0.001	Yes
3100 vs. 0.5	0.319	11.131	<0.001	Yes
550 vs. 50	0.264	10.827	<0.001	Yes
140 vs. 0.5	0.259	10.636	<0.001	Yes
550 vs. 10	0.272	10.176	<0.001	Yes
550 vs. 0.5	0.220	9.044	<0.001	Yes
27500 vs. 20	0.280	8.730	<0.001	Yes
27500 vs. 50	0.263	8.222	<0.001	Yes
27500 vs. 10	0.271	8.014	<0.001	Yes
27500 vs. 0.5	0.220	6.864	<0.001	Yes
270 vs. 550	0.141	5.793	<0.001	Yes
270 vs. 27500	0.142	4.425	<0.001	Yes
270 vs. 140	0.102	4.201	<0.001	Yes
3100 vs. 550	0.0982	3.429	0.012	Yes
270 vs. 1270	0.0743	3.048	0.041	Yes
3100 vs. 27500	0.0987	2.790	0.085	No
1270 vs. 550	0.0669	2.745	0.091	No
0.5 vs. 20	0.0598	2.452	0.187	No
1270 vs. 27500	0.0675	2.106	0.379	No
3100 vs. 140	0.0594	2.074	0.378	No
0.5 vs. 10	0.0513	1.920	0.467	No
0.5 vs. 50	0.0435	1.784	0.543	No
140 vs. 550	0.0388	1.592	0.658	No
270 vs. 3100	0.0431	1.504	0.682	No
140 vs. 27500	0.0394	1.228	0.825	No
1270 vs. 140	0.0281	1.153	0.821	No
3100 vs. 1270	0.0312	1.092	0.801	No
50 vs. 20	0.0163	0.668	0.940	No
10 vs. 20	0.00850	0.318	0.984	No
50 vs. 10	0.00779	0.292	0.947	No
550 vs. 27500	0.000535	0.0167	0.987	No

Comparisons for factor: **week**

**Comparison**	**Diff of Means**	**t**	**P**	**P<0.050**
w10 vs. w5	0.0425	3.500	<0.001	Yes

6) OJIP - Φ_{RE1o}

Three Way Analysis of Variance
General Linear Model
Dependent Variable: Φ_{RE1o}
Normality Test (Shapiro-Wilk) Failed (P < 0.050)
Equal Variance Test: Failed (P < 0.050)

**Source of Variation**	**DF**	**SS**	**MS**	**F**	**P**
week	1	0.0351	0.0351	20.540	<0.001
Cd conc	8	0.483	0.0603	35.351	<0.001
Tissue	1	0.00146	0.00146	0.856	0.359
week x Cd conc	8	0.0854	0.0107	6.251	<0.001
week x Tissue	1	0.000507	0.000507	0.297	0.588
Cd conc x Tissue	8	0.00169	0.000211	0.124	0.998
week x Cd conc x Tissue	8	0.00343	0.000429	0.251	0.978
Residual	53	0.0905	0.00171		
Total	88	0.665	0.00756		

The main effects for week cannot be properly interpreted since the size of the factor's effect depends upon the level of another factor.

The main effects for Cd conc cannot be properly interpreted since the size of the factor's effect depends upon the

level of another factor.

The difference in the mean values among the different levels of Tissue are not great enough to exclude the possibility that the difference is just due to random sampling variability after allowing for the effects of differences in week and Cd conc. There is not a statistically significant difference (P = 0.359).

The effect of different levels of week depends on what level of Cd conc is present. There is a statistically significant interaction between week and Cd conc. (P = <0.001)

The effect of different levels of week does not depend on what level of Tissue is present. There is not a statistically significant interaction between week and Tissue. (P = 0.588)

The effect of different levels of Cd conc does not depend on what level of Tissue is present. There is not a statistically significant interaction between Cd conc and Tissue. (P = 0.998)

All Pairwise Multiple Comparison Procedures (Holm-Sidak method):
Overall significance level = 0.05

Comparisons for factor: **week**

**Comparison**	**Diff of Means**	**t**	**P**	**P<0.050**
w5 vs. w10	0.0409 4.532	<0.001	Yes	

Comparisons for factor: **Cd conc**

**Comparison**	**Diff of Means**	**t**	**P**	**P<0.050**
50 vs. 1270	0.198	10.027	<0.001	Yes
50 vs. 140	0.186	9.873	<0.001	Yes
50 vs. 550	0.193	9.737	<0.001	Yes
50 vs. 3100	0.189	9.542	<0.001	Yes
50 vs. 270	0.155	8.876	<0.001	Yes
0.5 vs. 1270	0.161	8.164	<0.001	Yes
0.5 vs. 140	0.149	7.919	<0.001	Yes
0.5 vs. 550	0.156	7.874	<0.001	Yes
0.5 vs. 3100	0.152	7.679	<0.001	Yes
10 vs. 1270	0.150	7.561	<0.001	Yes
20 vs. 1270	0.144	7.382	<0.001	Yes
10 vs. 140	0.137	7.287	<0.001	Yes
10 vs. 550	0.144	7.272	<0.001	Yes
20 vs. 140	0.132	7.101	<0.001	Yes
20 vs. 550	0.139	7.089	<0.001	Yes
10 vs. 3100	0.140	7.076	<0.001	Yes
20 vs. 3100	0.135	6.892	<0.001	Yes
0.5 vs. 270	0.118	6.769	<0.001	Yes
10 vs. 270	0.106	6.088	<0.001	Yes
20 vs. 270	0.101	5.876	<0.001	Yes
50 vs. 20	0.0540	2.899	0.084	No
50 vs. 10	0.0488	2.586	0.172	No
270 vs. 1270	0.0431	2.332	0.283	No
270 vs. 550	0.0374	2.022	0.474	No
50 vs. 0.5	0.0369	1.954	0.499	No
270 vs. 3100	0.0335	1.813	0.578	No
270 vs. 140	0.0310	1.771	0.577	No
0.5 vs. 20	0.0171	0.920	0.982	No
0.5 vs. 10	0.0119	0.632	0.998	No
140 vs. 1270	0.0121	0.613	0.996	No
3100 vs. 1270	0.00959	0.464	0.998	No
140 vs. 550	0.00640	0.323	0.999	No
10 vs. 20	0.00521	0.280	0.998	No
550 vs. 1270	0.00573	0.277	0.990	No
3100 vs. 550	0.00386	0.187	0.978	No
140 vs. 3100	0.00254	0.128	0.898	No

Comparisons for factor: **Tissue**

Comparison	Diff of Means	t	P	P<0.050
Vein vs. Mesophyll	0.00835	0.925	0.359	No

Comparisons for factor: **Cd conc within w5**

Comparison	Diff of Means	t	P	P<0.05
10 vs. 140	0.225	7.693	<0.001	Yes
10 vs. 550	0.223	7.626	<0.001	Yes
10 vs. 1270	0.220	7.527	<0.001	Yes
20 vs. 140	0.217	7.420	<0.001	Yes
20 vs. 550	0.215	7.353	<0.001	Yes
20 vs. 1270	0.212	7.254	<0.001	Yes
50 vs. 140	0.208	7.121	<0.001	Yes
50 vs. 550	0.206	7.054	<0.001	Yes
10 vs. 270	0.188	7.031	<0.001	Yes
50 vs. 1270	0.203	6.955	<0.001	Yes
10 vs. 3100	0.199	6.827	<0.001	Yes
20 vs. 270	0.180	6.732	<0.001	Yes
20 vs. 3100	0.191	6.554	<0.001	Yes
50 vs. 270	0.171	6.404	<0.001	Yes
50 vs. 3100	0.183	6.255	<0.001	Yes
0.5 vs. 140	0.166	5.670	<0.001	Yes
0.5 vs. 550	0.164	5.603	<0.001	Yes
0.5 vs. 1270	0.161	5.504	<0.001	Yes
0.5 vs. 270	0.128	4.815	<0.001	Yes
0.5 vs. 3100	0.140	4.804	<0.001	Yes
10 vs. 0.5	0.0591	2.023	0.546	No
20 vs. 0.5	0.0511	1.750	0.740	No
50 vs. 0.5	0.0424	1.451	0.902	No
270 vs. 140	0.0372	1.396	0.909	No
270 vs. 550	0.0353	1.323	0.922	No
270 vs. 1270	0.0324	1.215	0.944	No
3100 vs. 140	0.0253	0.866	0.993	No
3100 vs. 550	0.0233	0.799	0.993	No
3100 vs. 1270	0.0205	0.700	0.995	No
10 vs. 50	0.0167	0.572	0.997	No
270 vs. 3100	0.0119	0.448	0.998	No
20 vs. 50	0.00875	0.300	0.999	No
10 vs. 20	0.00797	0.273	0.998	No
1270 vs. 140	0.00485	0.166	0.998	No
1270 vs. 550	0.00289	0.0989	0.994	No
550 vs. 140	0.00196	0.0671	0.947	No

Comparisons for factor: **Cd conc within w10**

Comparison	Diff of Means	t	P	P<0.05
50 vs. 3100	0.195	7.302	<0.001	Yes
50 vs. 1270	0.193	7.254	<0.001	Yes
50 vs. 140	0.164	6.890	<0.001	Yes
50 vs. 550	0.179	6.716	<0.001	Yes
50 vs. 270	0.140	6.172	<0.001	Yes
0.5 vs. 3100	0.163	6.128	<0.001	Yes
0.5 vs. 1270	0.162	6.080	<0.001	Yes
0.5 vs. 140	0.133	5.577	<0.001	Yes
0.5 vs. 550	0.148	5.542	<0.001	Yes
50 vs. 20	0.117	5.053	<0.001	Yes
50 vs. 10	0.114	4.790	<0.001	Yes
0.5 vs. 270	0.108	4.788	<0.001	Yes
0.5 vs. 20	0.0854	3.697	0.012	Yes
0.5 vs. 10	0.0829	3.477	0.023	Yes

10 vs. 3100	0.0805	3.018	0.083	No
20 vs. 3100	0.0780	3.002	0.082	No
10 vs. 1270	0.0792	2.970	0.086	No
20 vs. 1270	0.0768	2.953	0.085	No
10 vs. 550	0.0649	2.432	0.285	No
20 vs. 550	0.0624	2.401	0.290	No
270 vs. 3100	0.0551	2.153	0.443	No
270 vs. 1270	0.0538	2.103	0.460	No
10 vs. 140	0.0501	2.100	0.439	No
20 vs. 140	0.0476	2.063	0.443	No
270 vs. 550	0.0394	1.542	0.809	No
50 vs. 0.5	0.0313	1.313	0.908	No
140 vs. 3100	0.0304	1.139	0.951	No
10 vs. 270	0.0254	1.123	0.939	No
140 vs. 1270	0.0291	1.091	0.928	No
270 vs. 140	0.0247	1.091	0.900	No
20 vs. 270	0.0230	1.052	0.880	No
140 vs. 550	0.0148	0.553	0.987	No
550 vs. 3100	0.0156	0.535	0.973	No
550 vs. 1270	0.0144	0.491	0.947	No
10 vs. 20	0.00245	0.106	0.993	No
1270 vs. 3100	0.00127	0.0436	0.965	No

Comparisons for factor: **week within 0.5**

**Comparison**	**Diff of Means**	**t**	**P**	**P<0.05**
w5 vs. w10	0.0136	0.510	0.612	No

Comparisons for factor: **week within 10**

**Comparison**	**Diff of Means**	**t**	**P**	**P<0.05**
w5 vs. w10	0.156	5.837	<0.001	Yes

Comparisons for factor: **week within20**

**Comparison**	**Diff of Means**	**t**	**P**	**P<0.05**
w5 vs. w10	0.150	5.776	<0.001	Yes

Comparisons for factor: **week within 50**

**Comparison**	**Diff of Means**	**t**	**P**	**P<0.05**
w5 vs. w10	0.0247	0.926	0.359	No

Comparisons for factor: **week within 140**

**Comparison**	**Diff of Means**	**t**	**P**	**P<0.05**
w5 vs. w10	0.0190	0.712	0.479	No

Comparisons for factor: **week within 270**

**Comparison**	**Diff of Means**	**t**	**P**	**P<0.05**
w5 vs. w10	0.00644	0.285	0.777	No

Comparisons for factor: **week within 550**

**Comparison**	**Diff of Means**	**t**	**P**	**P<0.05**
w5 vs. w10	0.00228	0.0782	0.938	No

Comparisons for factor: **week within 1270**

**Comparison**	**Diff of Means**	**t**	**P**	**P<0.05**
w5 vs. w10	0.0150	0.512	0.611	No

Comparisons for factor: **week within 3100**

**Comparison**	**Diff of Means**	**t**	**P**	**P<0.05**
w5 vs. w10	0.0367	1.256	0.215	No

Power of performed test with alpha = 0.0500: for week : 0.996
Power of performed test with alpha = 0.0500: for Cd conc : 1.000

Power of performed test with alpha = 0.0500: for Tissue : 0.0500
Power of performed test with alpha = 0.0500: for week x Cd conc : 0.998
Power of performed test with alpha = 0.0500: for week x Tissue : 0.0500
Power of performed test with alpha = 0.0500: for Cd conc x Tissue : 0.0500

Least square means for week :

Group	Mean	SEM
w5	0.208	0.00676
w10	0.167	0.00598

Least square means for Cd conc :

Group	Mean	SEM
0.5	0.268	0.0133
10	0.256	0.0133
20	0.251	0.0130
50	0.305	0.0133
140	0.119	0.0133
270	0.150	0.0113
550	0.113	0.0146
1270	0.107	0.0146
3100	0.117	0.0146

Least square means for Tissue :

Group	Mean	SEM
Mesophyll	0.183	0.00633
Vein	0.192	0.00642

7) Metabolites in Leaves - Glycine

One Way Analysis of Variance
Dependent Variable: Glycine
Normality Test (Shapiro-Wilk) Failed (P < 0.050)
Equal Variance Test: Failed (P < 0.050)

Group Name	N	Missing	Mean	Std Dev	SEM
0.5 nM Cd	3	0	0.0596	0.00496	0.00286
20 nM Cd	3	0	0.0533	0.00918	0.00530
50 nM Cd	3	0	0.0694	0.00876	0.00506
140 nM Cd	3	0	0.300	0.179	0.103
270 nM Cd	3	0	0.565	0.316	0.183

Source of Variation	DF	SS	MS	F	P
Between Groups	4	0.603	0.151	5.689	0.012
Residual	10	0.265	0.0265		
Total	14	0.867			

The differences in the mean values among the treatment groups are greater than would be expected by chance; there is a statistically significant difference (P = 0.012).

Power of performed test with alpha = 0.050: 0.796

All Pairwise Multiple Comparison Procedures (Holm-Sidak method):
Overall significance level = 0.05

Comparisons for factor: **Cd conc**

Comparison	Diff of Means	t	P	P<0.050
270 nM Cd vs. 20 nM Cd	0.511	3.850	0.032	Yes
270 nM Cd vs. 0.5 nM Cd	0.505	3.802	0.031	Yes
270 nM Cd vs. 50 nM Cd	0.495	3.729	0.031	Yes
270 nM Cd vs. 140 nM Cd	0.265	1.995	0.416	No

140 nM Cd vs. 20 nM Cd	0.246	1.855	0.444	No
140 nM Cd vs. 0.5 nM Cd	0.240	1.807	0.412	No
140 nM Cd vs. 50 nM Cd	0.230	1.734	0.383	No
50 nM Cd vs. 20 nM Cd	0.0161	0.121	0.999	No
50 nM Cd vs. 0.5 nM Cd	0.00974	0.0733	0.997	No
0.5 nM Cd vs. 20 nM Cd	0.00631	0.0475	0.963	No

Metabolites in Leaves - Proline

One Way Analysis of Variance
Dependent Variable: Proline
Normality Test (Shapiro-Wilk) Failed (P < 0.050)
Equal Variance Test: Passed (P = 0.226)

Group Name	N	Missing	Mean	Std Dev	SEM
0.5 nM Cd	3	0	0.0588	0.0128	0.00739
20 nM Cd	3	0	0.0586	0.0133	0.00767
50 nM Cd	3	0	0.0731	0.0156	0.00900
140 nM Cd	3	1	0.262	0.0535	0.0378
270 nM Cd	3	0	0.344	0.315	0.182

Source of Variation	DF	SS	MS	F	P
Between Groups	4	0.205	0.0513	2.283	0.140
Residual	9	0.202	0.0225		
Total	13	0.407			

The differences in the mean values among the treatment groups are not great enough to exclude the possibility that the difference is due to random sampling variability; there is not a statistically significant difference (P = 0.140).

Power of performed test with alpha = 0.050: 0.255

The power of the performed test (0.255) is below the desired power of 0.800.
Less than desired power indicates you are less likely to detect a difference when one actually exists. Negative results should be interpreted cautiously.

Metabolites in Leaves - GABA

One Way Analysis of Variance
Dependent Variable: GABA
Normality Test (Shapiro-Wilk) Passed (P = 0.310)
Equal Variance Test: Failed (P < 0.050)

Group Name	N	Missing	Mean	Std Dev	SEM
0.5 nM Cd	3	0	0.955	0.274	0.158
20 nM Cd	3	0	0.630	0.207	0.120
50 nM Cd	3	0	1.776	0.391	0.226
140 nM Cd	3	0	4.815	0.503	0.290
270 nM Cd	3	0	2.794	1.271	0.734

Source of Variation	DF	SS	MS	F	P
Between Groups	4	34.158	8.540	19.954	<0.001
Residual	10	4.280	0.428		
Total	14	38.438			

The differences in the mean values among the treatment groups are greater than would be expected by chance; there is a statistically significant difference (P = <0.001).

Power of performed test with alpha = 0.050: 1.000

All Pairwise Multiple Comparison Procedures (Holm-Sidak method):
Overall significance level = 0.05

Comparisons for factor: **Cd conc**

**Comparison**	**Diff of Means**	**t**	**P**	**P<0.050**
140 nM Cd vs. 10 nM Cd	4.185	7.834	<0.001	Yes
140 nM Cd vs. 0.5 nM Cd	3.861	7.227	<0.001	Yes
140 nM Cd vs. 50 nM Cd	3.040	5.690	0.002	Yes
270 nM Cd vs. 10 nM Cd	2.163	4.050	0.016	Yes
140 nM Cd vs. 270 nM Cd	2.021	3.784	0.021	Yes
270 nM Cd vs. 0.5 nM Cd	1.839	3.443	0.031	Yes
50 nM Cd vs. 10 nM Cd	1.145	2.144	0.211	No
270 nM Cd vs. 50 nM Cd	1.018	1.906	0.236	No
50 nM Cd vs. 0.5 nM Cd	0.821	1.537	0.286	No
0.5 nM Cd vs. 10 nM Cd	0.324	0.607	0.557	No

Metabolites in Leaves - Lactic Acid

One Way Analysis of Variance
Dependent Variable: Lactic acid
Normality Test (Shapiro-Wilk) Passed (P = 0.251)
Equal Variance Test: Passed (P = 0.699)

**Group Name**	**N**	**Missing**	**Mean**	**Std Dev**	**SEM**
0.5 nM Cd	3	0	0.151	0.0107	0.00616
20 nM Cd	3	0	0.142	0.00757	0.00437
50 nM Cd	3	0	0.202	0.0150	0.00867
140 nM Cd	3	0	0.160	0.000740	0.000427
270 nM Cd	3	0	0.168	0.00970	0.00560

**Source of Variation**	**DF**	**SS**	**MS**	**F**	**P**
Between Groups	4	0.00646	0.00162	16.457	<0.001
Residual	10	0.000982	0.0000982		
Total	14	0.00745			

The differences in the mean values among the treatment groups are greater than would be expected by chance; there is a statistically significant difference (P = <0.001).

Power of performed test with alpha = 0.050: 1.000

All Pairwise Multiple Comparison Procedures (Holm-Sidak method):
Overall significance level = 0.05

Comparisons for factor: **Cd conc**

**Comparison**	**Diff of Means**	**t**	**P**	**P<0.050**
50 nM Cd vs. 20 nM Cd	0.0602	7.442	<0.001	Yes
50 nM Cd vs. 0 nM Cd	0.0518	6.401	<0.001	Yes
50 nM Cd vs. 140 nM Cd	0.0420	5.197	0.003	Yes
50 nM Cd vs. 270 nM Cd	0.0347	4.289	0.011	Yes
270 nM Cd vs. 20 nM Cd	0.0255	3.153	0.060	No
140 nM Cd vs. 20 nM Cd	0.0182	2.246	0.220	No
270 nM Cd vs. 0.5 nM Cd	0.0171	2.112	0.222	No
140 nM Cd vs. 0.5 nM Cd	0.00975	1.204	0.588	No
0.5 nM Cd vs. 20 nM Cd	0.00842	1.041	0.541	No
270 nM Cd vs. 140 nM Cd	0.00734	0.907	0.386	No

Metabolites in Leaves - 2-oxo-glutarate

One Way Analysis of Variance
Dependent Variable: 2-oxo-glutarate
Normality Test (Shapiro-Wilk) Passed (P = 0.351)
Equal Variance Test: Failed (P < 0.050)

**Group Name**	**N**	**Missing**	**Mean**	**Std Dev**	**SEM**
0.5 nM Cd	3	0	1.067	0.458	0.265
20 nM Cd	3	0	0.984	0.620	0.358
50 nM Cd	3	0	1.168	0.244	0.141
140 nM Cd	3	0	0.567	0.101	0.0581
270 nM Cd	3	0	0.255	0.0264	0.0153

**Source of Variation**	**DF**	**SS**	**MS**	**F**	**P**
Between Groups	4	1.776	0.444	3.341	0.055
Residual	10	1.329	0.133		
Total	14	3.104			

The differences in the mean values among the treatment groups are not great enough to exclude the possibility that the difference is due to random sampling variability; there is not a statistically significant difference (P = 0.055).

Power of performed test with alpha = 0.050: 0.466

The power of the performed test (0.466) is below the desired power of 0.800.
Less than desired power indicates you are less likely to detect a difference when one actually exists. Negative results should be interpreted cautiously.

Metabolites in Leaves - Phosphoenolpyruvate (PEP)

One Way Analysis of Variance
Dependent Variable: Phosphoenolpyruvate
Normality Test (Shapiro-Wilk) Failed (P < 0.050)
Equal Variance Test: Passed (P = 0.634)

Group Name	N	Missing	Mean	Std Dev	SEM
0.5 nM Cd	3	0	0.0734	0.000691	0.000399
20 nM Cd	3	0	0.000	0.000	0.000
50 nM Cd	3	0	0.0501	0.0434	0.0251
140 nM Cd	3	0	0.103	0.0142	0.00819
270 nM Cd	3	0	0.101	0.0191	0.0110

Source of Variation	DF	SS	MS	F	P
Between Groups	4	0.0217	0.00542	11.065	0.001
Residual	10	0.00490	0.000490		
Total	14	0.0266			

The differences in the mean values among the treatment groups are greater than would be expected by chance; there is a statistically significant difference (P = 0.001).

Power of performed test with alpha = 0.050: 0.990

All Pairwise Multiple Comparison Procedures (Holm-Sidak method):
Overall significance level = 0.05

Comparisons for factor: Cd conc

**Comparison**	**Diff of Means**	**t**	**P**	**P<0.050**
140 nM Cd vs. 20 nM Cd	0.103	5.691	0.002	Yes
270 nM Cd vs. 20 nM Cd	0.101	5.577	0.002	Yes
0.5 nM Cd vs. 20 nM Cd	0.0734	4.058	0.018	Yes
140 nM Cd vs. 50 nM Cd	0.0527	2.917	0.103	No
270 nM Cd vs. 50 nM Cd	0.0507	2.804	0.107	No

50 nM Cd vs. 20 nM Cd	0.0501	2.773	0.095	No
140 nM Cd vs. 0.5 nM Cd	0.0295	1.632	0.437	No
270 nM Cd vs. 0.5 nM Cd	0.0275	1.519	0.407	No
0.5 nM Cd vs. 50 nM Cd	0.0232	1.285	0.404	No
140 nM Cd vs. 270 nM Cd	0.00205	0.113	0.912	No

Metabolites in Leaves - Malic acid

One Way Analysis of Variance
Dependent Variable: Malic acid
Normality Test (Shapiro-Wilk) Passed (P = 0.472)
Equal Variance Test: Passed (P = 0.417)

Group Name	N	Missing	Mean	Std Dev	SEM
0.5 nM Cd	3	0	97.310	4.717	2.724
20 nM Cd	3	0	100.353	7.686	4.438
50 nM Cd	3	0	91.589	1.227	0.708
140 nM Cd	3	0	18.760	8.906	5.142
270 nM Cd	3	0	5.332	2.321	1.340

Source of Variation	DF	SS	MS	F	P
Between Groups	4	26015.814	6503.954	194.114	<0.001
Residual	10	335.059	33.506		
Total	14	26350.873			

The differences in the mean values among the treatment groups are greater than would be expected by chance; there is a statistically significant difference (P = <0.001).

Power of performed test with alpha = 0.050: 1.000

All Pairwise Multiple Comparison Procedures (Holm-Sidak method):
Overall significance level = 0.05

Comparisons for factor: **Cd conc**

Comparison	Diff of Means	t	P	P<0.050
20 nM Cd vs. 270 nM Cd	95.021	20.105	<0.001	Yes
0.5 nM Cd vs. 270 nM Cd	91.978	19.461	<0.001	Yes
50 nM Cd vs. 270 nM Cd	86.257	18.251	<0.001	Yes
20 nM Cd vs. 140 nM Cd	81.592	17.264	<0.001	Yes
0.5 nM Cd vs. 140 nM Cd	78.549	16.620	<0.001	Yes
50 nM Cd vs. 140 nM Cd	72.829	15.409	<0.001	Yes
140 nM Cd vs. 270 nM Cd	13.429	2.841	0.068	No
20 nM Cd vs. 50 nM Cd	8.764	1.854	0.255	No
0.5 nM Cd vs. 50 nM Cd	5.721	1.210	0.443	No
20 nM Cd vs. 0.5 nM Cd	3.043	0.644	0.534	No

Metabolites in Leaves - Hexose

One Way Analysis of Variance
Dependent Variable: Hexose
Normality Test (Shapiro-Wilk) Passed (P = 0.172)
Equal Variance Test: Passed (P = 0.480)

Group Name	N	Missing	Mean	Std Dev	SEM
0.5 nM Cd	3	0	2.967	0.741	0.428
20 nM Cd	3	0	1.332	0.741	0.428
50 nM Cd	3	0	3.038	0.453	0.262
140 nM Cd	3	0	2.593	2.044	1.180

270 nM Cd	3	0	1.283	0.342	0.198

Source of Variation	DF	SS	MS	F	P
Between Groups	4	9.086	2.272	2.028	0.166
Residual	10	11.199	1.120		
Total	14	20.285			

The differences in the mean values among the treatment groups are not great enough to exclude the possibility that the difference is due to random sampling variability; there is not a statistically significant difference (P = 0.166).

Power of performed test with alpha = 0.050: 0.217

The power of the performed test (0.217) is below the desired power of 0.800.
Less than desired power indicates you are less likely to detect a difference when one actually exists. Negative results should be interpreted cautiously.

Metabolites in Leaves - Sorbitol/Manitol

One Way Analysis of Variance
Dependent Variable: Sorbitol/Manitol
Normality Test (Shapiro-Wilk) Passed (P = 0.340)
Equal Variance Test: Passed (P = 0.151)

Group Name	N	Missing	Mean	Std Dev	SEM
0.5 nM Cd	3	0	1.782	0.145	0.0838
20 nM Cd	3	0	1.739	0.0676	0.0390
50 nM Cd	3	0	2.125	0.238	0.138
140 nM Cd	3	0	2.130	0.327	0.189
270 nM Cd	3	0	2.879	0.712	0.411

Source of Variation	DF	SS	MS	F	P
Between Groups	4	2.505	0.626	4.499	0.025
Residual	10	1.392	0.139		
Total	14	3.897			

The differences in the mean values among the treatment groups are greater than would be expected by chance; there is a statistically significant difference (P = 0.025).

Power of performed test with alpha = 0.050: 0.655

All Pairwise Multiple Comparison Procedures (Holm-Sidak method):
Overall significance level = 0.05

Comparisons for factor: **Cd conc**

Comparison	Diff of Means	t	P	P<0.050
270 nM Cd vs. 20 nM Cd	1.140	3.742	0.038	Yes
270 nM Cd vs. 0.5 nM Cd	1.097	3.601	0.043	Yes
270 nM Cd vs. 50 nM Cd	0.754	2.476	0.234	No
270 nM Cd vs. 140 nM Cd	0.749	2.459	0.213	No
140 nM Cd vs. 20 nM Cd	0.391	1.283	0.789	No
50 nM Cd vs. 20 nM Cd	0.386	1.266	0.736	No
140 nM Cd vs. 0.5 nM Cd	0.348	1.142	0.731	No
50 nM Cd vs. 0.5 nM Cd	0.343	1.126	0.637	No
0.5 nM Cd vs. 20 nM Cd	0.0428	0.140	0.988	No
140 nM Cd vs. 50 nM Cd	0.00496	0.0163	0.987	No

Metabolites in Leaves - Glucose-x-phosphate

One Way Analysis of Variance
Dependent Variable: Glucose-x-phosphate
Normality Test (Shapiro-Wilk) Passed (P = 0.595)
Equal Variance Test: Passed (P = 0.350)

Group Name	N	Missing	Mean	Std Dev	SEM
0.5 nM Cd	3	0	1.603	0.784	0.453
20 nM Cd	3	0	0.868	1.110	0.641
50 nM Cd	3	0	1.107	0.501	0.289
140 nM Cd	3	0	0.170	0.238	0.138
270 nM Cd	3	0	1.989	2.054	1.186

Source of Variation	DF	SS	MS	F	P
Between Groups	4	5.853	1.463	1.148	0.389
Residual	10	12.744	1.274		
Total	14	18.597			

The differences in the mean values among the treatment groups are not great enough to exclude the possibility that the difference is due to random sampling variability; there is not a statistically significant difference (P = 0.389).

Power of performed test with alpha = 0.050: 0.070

The power of the performed test (0.070) is below the desired power of 0.800.
Less than desired power indicates you are less likely to detect a difference when one actually exists. Negative results should be interpreted cautiously.

Metabolites in Leaves - Arabitol

One Way Analysis of Variance
Dependent Variable: Arabitol
Normality Test (Shapiro-Wilk) Passed (P = 0.294)
Equal Variance Test: Passed (P = 0.789)

Group Name	N	Missing	Mean	Std Dev	SEM
0.5 nM Cd	3	0	1.831	0.562	0.324
20 nM Cd	3	0	1.505	0.652	0.376
50 nM Cd	3	0	1.719	0.602	0.347
140 nM Cd	3	0	1.049	0.918	0.530
270 nM Cd	3	0	4.042	1.330	0.768

Source of Variation	DF	SS	MS	F	P
Between Groups	4	16.268	4.067	5.476	0.013
Residual	10	7.427	0.743		
Total	14	23.695			

The differences in the mean values among the treatment groups are greater than would be expected by chance; there is a statistically significant difference (P = 0.013).

Power of performed test with alpha = 0.050: 0.775

All Pairwise Multiple Comparison Procedures (Holm-Sidak method):
Overall significance level = 0.05

Comparisons for factor: **Cd conc**

Comparison	Diff of Means	t	P	P<0.050
270 nM Cd vs. 140 nM Cd	2.993	4.254	0.017	Yes
270 nM Cd vs. 20 nM Cd	2.537	3.606	0.042	Yes
270 nM Cd vs. 50 nM Cd	2.323	3.302	0.062	No
270 nM Cd vs. 0.5 nM Cd	2.211	3.142	0.071	No

0.5 nM Cd vs. 140 nM Cd	0.782	1.111	0.874	No
50 nM Cd vs. 140 nM Cd	0.670	0.952	0.896	No
20 nM Cd vs. 140 nM Cd	0.456	0.648	0.952	No
0.5 nM Cd vs. 20 nM Cd	0.326	0.463	0.958	No
50 nM Cd vs. 20 nM Cd	0.214	0.304	0.946	No
0.5 nM Cd vs. 50 nM Cd	0.112	0.159	0.877	No

Metabolites in Leaves - Arabinose

One Way Analysis of Variance
Dependent Variable: Arabinose
Normality Test (Shapiro-Wilk) Passed (P = 0.219)
Equal Variance Test: Passed (P = 0.679)

Group Name	N	Missing	Mean	Std Dev	SEM
0.5 nM Cd	3	0	2.569	1.855	1.071
20 nM Cd	3	0	1.959	2.794	1.613
50 nM Cd	3	0	2.263	1.197	0.691
140 nM Cd	3	0	0.451	0.281	0.162
270 nM Cd	3	0	1.238	0.703	0.406

Source of Variation	DF	SS	MS	F	P
Between Groups	4	8.739	2.185	0.824	0.539
Residual	10	26.506	2.651		
Total	14	35.245			

The differences in the mean values among the treatment groups are not great enough to exclude the possibility that the difference is due to random sampling variability; there is not a statistically significant difference (P = 0.539).

Power of performed test with alpha = 0.050: 0.050

The power of the performed test (0.050) is below the desired power of 0.800.
Less than desired power indicates you are less likely to detect a difference when one actually exists. Negative results should be interpreted cautiously.

Metabolites in Roots - Glycine

One Way Analysis of Variance
Dependent Variable: Glycine (Roots)
Normality Test (Shapiro-Wilk) Failed (P < 0.050)
Equal Variance Test: Passed (P = 0.629)

Group Name	N	Missing	Mean	Std Dev	SEM
0.5 nM Cd	3	0	0.0348	0.0302	0.0174
20 nM Cd	3	0	0.0498	0.00229	0.00132
50 nM Cd	3	0	0.0457	0.00498	0.00287
140 nM Cd	3	0	0.0464	0.00765	0.00442
270 nM Cd	2	0	0.0510	0.00949	0.00671

Source of Variation	DF	SS	MS	F	P
Between Groups	4	0.000461	0.000115	0.498	0.739
Residual	9	0.00209	0.000232		
Total	13	0.00255			

The differences in the mean values among the treatment groups are not great enough to exclude the possibility that the difference is due to random sampling variability; there is not a statistically significant difference (P = 0.739).

Power of performed test with alpha = 0.050: 0.050

The power of the performed test (0.050) is below the desired power of 0.800.
Less than desired power indicates you are less likely to detect a difference when one actually exists. Negative results should be interpreted cautiously.

Metabolites in Roots - Proline

One Way Analysis of Variance
Dependent Variable: Proline (Roots)
Normality Test (Shapiro-Wilk) Passed (P = 0.345)
Equal Variance Test: Passed (P = 0.317)

Group Name	N	Missing	Mean	Std Dev	SEM
0.5 nM Cd	3	0	0.0210	0.00357	0.00206
20 nM Cd	3	0	0.0193	0.00180	0.00104
50 nM Cd	3	0	0.0135	0.0117	0.00677
140 nM Cd	3	0	0.0413	0.0145	0.00840
270 nM Cd	2	0	0.0679	0.0187	0.0132

Source of Variation	DF	SS	MS	F	P
Between Groups	4	0.00466	0.00116	9.704	0.003
Residual	9	0.00108	0.000120		
Total	13	0.00574			

The differences in the mean values among the treatment groups are greater than would be expected by chance; there is a statistically significant difference (P = 0.003).

Power of performed test with alpha = 0.050: 0.969

All Pairwise Multiple Comparison Procedures (Holm-Sidak method):
Overall significance level = 0.05

Comparisons for factor: **Cd conc**

Comparison	Diff of Means	t	P	P<0.050
270 nM Cd vs. 50 nM Cd	0.0544	5.437	0.004	Yes
270 nM Cd vs. 20 nM Cd	0.0486	4.862	0.008	Yes
270 nM Cd vs. 0.5 nM Cd	0.0469	4.691	0.009	Yes
140 nM Cd vs. 50 nM Cd	0.0278	3.105	0.085	No
270 nM Cd vs. 140 nM Cd	0.0266	2.659	0.147	No
140 nM Cd vs. 20 nM Cd	0.0220	2.463	0.167	No
140 nM Cd vs. 0.5 nM Cd	0.0203	2.271	0.183	No
0.5 nM Cd vs. 50 nM Cd	0.00746	0.834	0.811	No
20 nM Cd vs. 50 nM Cd	0.00574	0.642	0.786	No
0.5 nM Cd vs. 20 nM Cd	0.00172	0.192	0.852	No

Metabolites in Roots - GABA

One Way Analysis of Variance
Dependent Variable: GABA (Roots)
Normality Test (Shapiro-Wilk) Failed (P < 0.050)
Equal Variance Test: Failed (P < 0.050)

Group Name	N	Missing	Mean	Std Dev	SEM
0.5 nM Cd	3	0	0.00385	0.00358	0.00207
20 nM Cd	3	0	0.00407	0.000739	0.000427
50 nM Cd	3	0	0.00330	0.00295	0.00171
140 nM Cd	3	0	0.123	0.114	0.0659
270 nM Cd	2	0	0.711	0.742	0.525

Source of Variation	DF	SS	MS	F	P
Between Groups	4	0.819	0.205	3.196	0.068
Residual	9	0.577	0.0641		
Total	13	1.396			

The differences in the mean values among the treatment groups are not great enough to exclude the possibility that the difference is due to random sampling variability; there is not a statistically significant difference (P = 0.068).

Power of performed test with alpha = 0.050: 0.421

The power of the performed test (0.421) is below the desired power of 0.800.
Less than desired power indicates you are less likely to detect a difference when one actually exists. Negative results should be interpreted cautiously.

Metabolites in Roots - Histidine

One Way Analysis of Variance
Dependent Variable: Histidine (Roots)
Normality Test (Shapiro-Wilk) Failed (P < 0.050)
Equal Variance Test: Passed (P = 0.257)

Group Name	N	Missing	Mean	Std Dev	SEM
0.5 nM Cd	3	0	0.182	0.0105	0.00604
20 nM Cd	3	0	0.164	0.194	0.112
50 nM Cd	3	0	0.276	0.178	0.103
140 nM Cd	3	0	3.314	3.487	2.013
270 nM Cd	2	0	9.793	3.478	2.459

Source of Variation	DF	SS	MS	F	P
Between Groups	4	154.758	38.690	9.528	0.003
Residual	9	36.547	4.061		
Total	13	191.305			

The differences in the mean values among the treatment groups are greater than would be expected by chance; there is a statistically significant difference (P = 0.003).

Power of performed test with alpha = 0.050: 0.965

All Pairwise Multiple Comparison Procedures (Holm-Sidak method):
Overall significance level = 0.05

Comparisons for factor: **Cd conc**

Comparison	Diff of Means	t	P	P<0.050
270 nM Cd vs. 20 nM Cd	9.629	5.234	0.005	Yes
270 nM Cd vs. 0.5 nM Cd	9.611	5.224	0.005	Yes
270 nM Cd vs. 50 nM Cd	9.517	5.174	0.005	Yes
270 nM Cd vs. 140 nM Cd	6.479	3.522	0.045	Yes
140 nM Cd vs. 20 nM Cd	3.150	1.914	0.424	No
140 nM Cd vs. 0.5 nM Cd	3.132	1.904	0.374	No
140 nM Cd vs. 50 nM Cd	3.039	1.847	0.338	No
50 nM Cd vs. 20 nM Cd	0.111	0.0677	1.000	No
50 nM Cd vs. 0.5 nM Cd	0.0935	0.0569	0.998	No
0.5 nM Cd vs. 20 nM Cd	0.0179	0.0109	0.992	No

Metabolites in Roots - Lactic acid

One Way Analysis of Variance
Dependent Variable: Lactic acid (Roots)

Normality Test (Shapiro-Wilk) Passed (P = 0.969)
Equal Variance Test: Passed (P = 0.631)

**Group Name**	**N**	**Missing**	**Mean**	**Std Dev**	**SEM**
0.5 nM Cd	3	0	0.115	0.0680	0.0392
20 nM Cd	3	0	0.0938	0.0108	0.00624
50 nM Cd	3	0	0.126	0.0546	0.0315
140 nM Cd	3	0	0.0832	0.0300	0.0173
270 nM Cd	2	0	0.0829	0.0162	0.0114

**Source of Variation**	**DF**	**SS**	**MS**	**F**	**P**
Between Groups	4	0.00422	0.00106	0.543	0.709
Residual	9	0.0175	0.00194		
Total	13	0.0217			

The differences in the mean values among the treatment groups are not great enough to exclude the possibility that the difference is due to random sampling variability; there is not a statistically significant difference (P = 0.709).

Power of performed test with alpha = 0.050: 0.050

The power of the performed test (0.050) is below the desired power of 0.800.
Less than desired power indicates you are less likely to detect a difference when one actually exists. Negative results should be interpreted cautiously.

Metabolites in Roots - 2-oxo-glutarate

One Way Analysis of Variance
Dependent Variable: 2-oxo-glutarate (Roots)
Normality Test (Shapiro-Wilk) Passed (P = 0.060)
Equal Variance Test: Failed (P < 0.050)

**Group Name**	**N**	**Missing**	**Mean**	**Std Dev**	**SEM**
0.5 nM Cd	3	0	0.170	0.161	0.0932
20 nM Cd	3	0	0.196	0.0182	0.0105
50 nM Cd	3	0	0.178	0.0242	0.0140
140 nM Cd	3	0	0.000	0.000	0.000
270 nM Cd	2	0	0.112	0.158	0.112

**Source of Variation**	**DF**	**SS**	**MS**	**F**	**P**
Between Groups	4	0.0758	0.0189	2.160	0.155
Residual	9	0.0790	0.00877		
Total	13	0.155			

The differences in the mean values among the treatment groups are not great enough to exclude the possibility that the difference is due to random sampling variability; there is not a statistically significant difference (P = 0.155).

Power of performed test with alpha = 0.050: 0.232

The power of the performed test (0.232) is below the desired power of 0.800.
Less than desired power indicates you are less likely to detect a difference when one actually exists. Negative results should be interpreted cautiously.

Metabolites in Roots - Phosphoenolpyruvate (PEP)

One Way Analysis of Variance
Dependent Variable: PEP
Normality Test (Shapiro-Wilk) Passed (P = 0.650)
Equal Variance Test: Passed (P = 0.430)

Group Name	N	Missing	Mean	Std Dev	SEM
0.5 nM Cd	3	0	0.0631	0.0546	0.0315
20 nM Cd	3	0	0.0646	0.0560	0.0324
50 nM Cd	3	0	0.0331	0.0574	0.0331
140 nM Cd	3	0	0.111	0.0142	0.00817
270 nM Cd	2	0	0.120	0.0177	0.0125

Source of Variation	DF	SS	MS	F	P
Between Groups	4	0.0139	0.00347	1.597	0.257
Residual	9	0.0196	0.00217		
Total	13	0.0334			

The differences in the mean values among the treatment groups are not great enough to exclude the possibility that the difference is due to random sampling variability; there is not a statistically significant difference (P = 0.257).

Power of performed test with alpha = 0.050: 0.136

The power of the performed test (0.136) is below the desired power of 0.800.
Less than desired power indicates you are less likely to detect a difference when one actually exists. Negative results should be interpreted cautiously.

Metabolites in Roots - Malic acid

One Way Analysis of Variance
Dependent Variable: Malic acid
Normality Test (Shapiro-Wilk) Passed (P = 0.172)
Equal Variance Test: Passed (P = 0.200)

Group Name	N	Missing	Mean	Std Dev	SEM
0.5 nM Cd	3	0	5.813	5.062	2.923
20 nM Cd	3	0	7.975	5.973	3.448
50 nM Cd	3	0	2.506	2.176	1.257
140 nM Cd	3	0	0.238	0.0680	0.0392
270 nM Cd	2	0	1.152	0.748	0.529

Source of Variation	DF	SS	MS	F	P
Between Groups	4	121.436	30.359	2.060	0.169
Residual	9	132.642	14.738		
Total	13	254.078			

The differences in the mean values among the treatment groups are not great enough to exclude the possibility that the difference is due to random sampling variability; there is not a statistically significant difference (P = 0.169).

Power of performed test with alpha = 0.050: 0.215

The power of the performed test (0.215) is below the desired power of 0.800.
Less than desired power indicates you are less likely to detect a difference when one actually exists. Negative results should be interpreted cautiously.

Metabolites in Roots - Hexose

One Way Analysis of Variance
Dependent Variable: Hexose
Normality Test (Shapiro-Wilk) Passed (P = 0.554)
Equal Variance Test: Failed (P < 0.050)

Group Name	N	Missing	Mean	Std Dev	SEM
0.5 nM Cd	3	0	0.649	0.371	0.214

20 nM Cd	3	0	0.994	0.783	0.452
50 nM Cd	3	0	0.545	0.441	0.255
140 nM Cd	3	0	2.658	0.829	0.478
270 nM Cd	2	0	2.406	3.082	2.179

**Source of Variation**	**DF**	**SS**	**MS**	**F**	**P**
Between Groups	4	11.149	2.787	1.966	0.184
Residual	9	12.760	1.418		
Total	13	23.909			

The differences in the mean values among the treatment groups are not great enough to exclude the possibility that the difference is due to random sampling variability; there is not a statistically significant difference (P = 0.184).

Power of performed test with alpha = 0.050: 0.198

The power of the performed test (0.198) is below the desired power of 0.800.
Less than desired power indicates you are less likely to detect a difference when one actually exists. Negative results should be interpreted cautiously.

Metabolites in Roots - Sorbitol/Manitol

One Way Analysis of Variance
Dependent Variable: Sorbitol/Manitol
Normality Test (Shapiro-Wilk) Passed (P = 0.996)
Equal Variance Test: Failed (P < 0.050)

**Group Name**	**N**	**Missing**	**Mean**	**Std Dev**	**SEM**
0.5 nM Cd	3	0	1.843	0.313	0.181
20 nM Cd	3	0	0.983	0.186	0.107
50 nM Cd	3	0	1.054	0.218	0.126
140 nM Cd	3	0	3.487	0.389	0.225
270 nM Cd	2	0	4.291	0.803	0.568

**Source of Variation**	**DF**	**SS**	**MS**	**F**	**P**
Between Groups	4	22.484	5.621	38.669	<0.001
Residual	9	1.308	0.145		
Total	13	23.792			

The differences in the mean values among the treatment groups are greater than would be expected by chance; there is a statistically significant difference (P = <0.001).

Power of performed test with alpha = 0.050: 1.000

All Pairwise Multiple Comparison Procedures (Holm-Sidak method):
Overall significance level = 0.05

Comparisons for factor: Cd conc

**Comparison**	**Diff of Means**	**t**	**P**	**P<0.050**
270 nM Cd vs. 20 nM Cd	3.308	9.505	<0.001	Yes
270 nM Cd vs. 50 nM Cd	3.238	9.303	<0.001	Yes
140 nM Cd vs. 20 nM Cd	2.504	8.043	<0.001	Yes
140 nM Cd vs. 50 nM Cd	2.433	7.817	<0.001	Yes
270 nM Cd vs. 0 nM Cd	2.449	7.036	<0.001	Yes
140 nM Cd vs. 0 nM Cd	1.644	5.282	0.003	Yes
0 nM Cd vs. 20 nM Cd	0.859	2.761	0.085	No
0 nM Cd vs. 50 nM Cd	0.789	2.534	0.093	No
270 nM Cd vs. 140 nM Cd	0.805	2.311	0.090	No
50 nM Cd vs. 20 nM Cd	0.0705	0.226	0.826	No

Metabolites in Roots - Glucose-x-phosphate

One Way Analysis of Variance
Dependent Variable: Glucose-x-phosphate (Roots)
Normality Test (Shapiro-Wilk) Passed (P = 0.791)
Equal Variance Test: Passed (P = 0.538)

Group Name	N	Missing	Mean	Std Dev	SEM
0.5 nM Cd	3	0	1.854	0.408	0.236
20 nM Cd	3	0	1.761	0.162	0.0937
50 nM Cd	3	0	1.872	0.231	0.133
140 nM Cd	3	0	2.992	0.673	0.389
270 nM Cd	2	0	3.614	1.075	0.760

Source of Variation	DF	SS	MS	F	P
Between Groups	4	6.893	1.723	6.069	0.012
Residual	9	2.555	0.284		
Total	13	9.448			

The differences in the mean values among the treatment groups are greater than would be expected by chance; there is a statistically significant difference (P = 0.012).

Power of performed test with alpha = 0.050: 0.808

All Pairwise Multiple Comparison Procedures (Holm-Sidak method):
Overall significance level = 0.05

Comparisons for factor: **Cd conc**

Comparison	Diff of Means	t	P	P<0.050
270 nM Cd vs. 20 nM Cd	1.853	3.809	0.041	Yes
270 nM Cd vs. 0.5 nM Cd	1.760	3.618	0.049	Yes
270 nM Cd vs. 50 nM Cd	1.741	3.580	0.046	Yes
140 nM Cd vs. 20 nM Cd	1.232	2.831	0.130	No
140 nM Cd vs. 0.5 nM Cd	1.139	2.617	0.156	No
140 nM Cd vs. 50 nM Cd	1.120	2.574	0.141	No
270 nM Cd vs. 140 nM Cd	0.621	1.277	0.655	No
50 nM Cd vs. 20 nM Cd	0.111	0.256	0.992	No
0.5 nM Cd vs. 20 nM Cd	0.0930	0.214	0.973	No
50 nM Cd vs. 0.5 nM Cd	0.0185	0.0424	0.967	No

Metabolites in Roots - Arabitol

One Way Analysis of Variance
Dependent Variable: Arabitol (Roots)
Normality Test (Shapiro-Wilk) Passed (P = 0.616)
Equal Variance Test: Passed (P = 0.399)

Group Name	N	Missing	Mean	Std Dev	SEM
0.5 nM Cd	3	0	1.402	0.560	0.323
20 nM Cd	3	0	1.417	0.130	0.0750
50 nM Cd	3	0	1.904	0.661	0.382
140 nM Cd	3	0	2.729	1.017	0.587
270 nM Cd	2	0	3.067	1.196	0.846

Source of Variation	DF	SS	MS	F	P
Between Groups	4	5.974	1.493	2.671	0.102
Residual	9	5.032	0.559		
Total	13	11.006			

The differences in the mean values among the treatment groups are not great enough to exclude the possibility that the difference

is due to random sampling variability; there is not a statistically significant difference (P = 0.102).

Power of performed test with alpha = 0.050: 0.326

The power of the performed test (0.326) is below the desired power of 0.800.
Less than desired power indicates you are less likely to detect a difference when one actually exists. Negative results should be interpreted cautiously.

Metabolites in Roots - Arabinose

One Way Analysis of Variance
Dependent Variable: Arabinose (Roots)
Normality Test (Shapiro-Wilk) Passed (P = 0.496)
Equal Variance Test: Passed (P = 0.785)

Group Name	N	Missing	Mean	Std Dev	SEM
0.5 nM Cd	3	0	1.213	0.582	0.336
20 nM Cd	3	0	1.650	1.204	0.695
50 nM Cd	3	0	1.039	0.135	0.0777
140 nM Cd	3	0	2.829	0.822	0.475
270 nM Cd	2	0	5.113	0.637	0.450

Source of Variation	DF	SS	MS	F	P
Between Groups	4	26.022	6.506	10.908	0.002
Residual	9	5.368	0.596		
Total	13	31.390			

The differences in the mean values among the treatment groups are greater than would be expected by chance; there is a statistically significant difference (P = 0.002).

Power of performed test with alpha = 0.050: 0.984

All Pairwise Multiple Comparison Procedures (Holm-Sidak method):
Overall significance level = 0.05

Comparisons for factor: **Cd conc**

Comparison	Diff of Means	t	P	P<0.050
270 nM Cd vs. 50 nM Cd	4.074	5.778	0.003	Yes
270 nM Cd vs. 0.5 nM Cd	3.900	5.532	0.003	Yes
270 nM Cd vs. 20 nM Cd	3.463	4.912	0.007	Yes
270 nM Cd vs. 140 nM Cd	2.284	3.239	0.069	No
140 nM Cd vs. 50 nM Cd	1.790	2.839	0.111	No
140 nM Cd vs. 0.5 nM Cd	1.616	2.564	0.144	No
140 nM Cd vs. 20 nM Cd	1.179	1.870	0.327	No
20 nM Cd vs. 50 nM Cd	0.611	0.969	0.735	No
20 nM Cd vs. 0.5 nM Cd	0.437	0.693	0.756	No
0.5 nM Cd vs. 50 nM Cd	0.174	0.275	0.789	No
