# Supplementary material for: Chronic exposure of soybean plants to nanomolar cadmium reveals specific additional high-affinity targets of cadmium toxicity
Source: J Exp Bot. 2019 Nov 24;71(4):1628–44. doi: 10.1093/jxb/erz530 (PMC7242006; doi:10.1093/jxb/erz530)
Supplement: erz530_suppl_supplementary_figures_S1-S5andprotocols_S1-S3 [file exbotj_71_4_1628_s10.pdf]

Supplementary data

Chronic exposure of soybean plants to nanomolar cadmium reveals specific additional high-affinity targets of Cd toxicity.

Elisa Andresen[1], Lyudmila Lyubenova[1], Tomáš Hubáček[2], Syed Nadeem Hussain Bokhari[1], Šárka Matoušková[3], Ana Mijovilovich[1], Jan Rohovec[3] and Hendrik Küpper[1,4]*

1) Biology Centre of the Czech Academy of Sciences, Institute of Plant Molecular Biology, Department of Plant Biophysics and Biochemistry. Branišovská 31/1160. 370 05 České Budějovice. Czech Republic.
2) Biology Centre of the Czech Academy of Sciences, Institute of Hydrobiology, Department of Hydrochemistry and Ecosystem Modelling, Na Sádkách 7, CZ-37005 České Budějovice, Czech Republic;
3) Institute of Geology, Czech Academy of Sciences, Department of Geological Processes, Rozvojová 269, 165 00 Praha 6, Czech Republic.
4) University of South Bohemia, Faculty of Sciences, Department of Experimental Plant Biology, Branišovská 31/1160, 370 05 České Budějovice, Czech Republic.

Author for correspondence: Hendrik Küpper, Tel: +420 387 775 537, Email: Hendrik.Kuepper@umbr.cas.cz

The following Supporting Information is available for this manuscript:

In this file:

Fig. S1: Nutrient utilization efficiency and utilization index of soybean plants exposed to various Cd concentrations for 10 weeks.

Fig. S2: Acetone extracts from soybean leaves exposed to low, medium and high but sublethal Cd concentrations for 10 weeks with focus on the Chl molecules.

Fig. S3: Effect of Cd exposure on membrane proteins of single soybean leaves after 5 weeks of exposure. ICP-MS count rates and protein chromatographs were normalized to the Co signal of added Vit B12. Chromatographs from one of two experiments are shown exemplarily.

Fig. S4: XANES of sample 2 of the purified LHC II from Cd-exposed soybean leaves.

Fig. S5: Concentration of Mn in the hydroponic solutions (A) and accumulation of Mn in different tissues of plants after 5 weeks (leaves only) and 10 weeks of exposure to various Cd concentrations (B).

Protocol S1: Identification of isolated proteins from selected fractions of membrane proteins from the leaves of soybean plants exposed to 50 nM Cd.

Protocol S2: Metabolomics. Isolation and identification of (hydrophilic) metabolites from leaves of soybean plants exposed to various Cd concentrations.

Protocol S3: Lipidomics. Isolation and identification of lipophilic compounds from soybean leaves of soybean plants exposed to various Cd concentrations.

In separate Excel files:
Table S1 (Excel file): List of metabolites detected in leaves and roots of soybean plants exposed to various Cd concentrations for 10 weeks.

Table S2 (Excel file): List of detected, and partly identified lipophilic compounds isolated from **leaves** of soybean plants exposed to various Cd concentrations for 10 weeks. Area: Peak area for compounds detected in the samples. Red markings in this sheet indicate values below LOD. Log2 fold change: Changes in the concentrations in respect to the control sample. Values are mean of three experiments and given in log2 mode.

Table S3 (Excel file): List of detected, and partly identified lipophilic compounds isolated from **roots** of soybean plants exposed to various Cd concentrations for 10 weeks. Area: Peak area for compounds detected in the samples. Red markings in this sheet indicate values below LOD. Log2 fold change: Changes in the concentrations in respect to the control sample. Values are mean of three experiments and given in log2 mode.

Table S4 (Excel file): Identification of membrane proteins and peptides isolated from leaves of soybean plants exposed to 50 nM Cd. The fractions containing LHC II trimers, as well as a LHC II neighbouring fraction eluting together with Cd were subjected to MALDI-TOF analyses.

In a separate pdf file:
Table S5: Reports from all statistical tests that were used for the description of results in the manuscript.

Figure S1: Nutrient utilization efficiency and utilization index of soybean plants exposed to various Cd concentrations for 10 weeks.

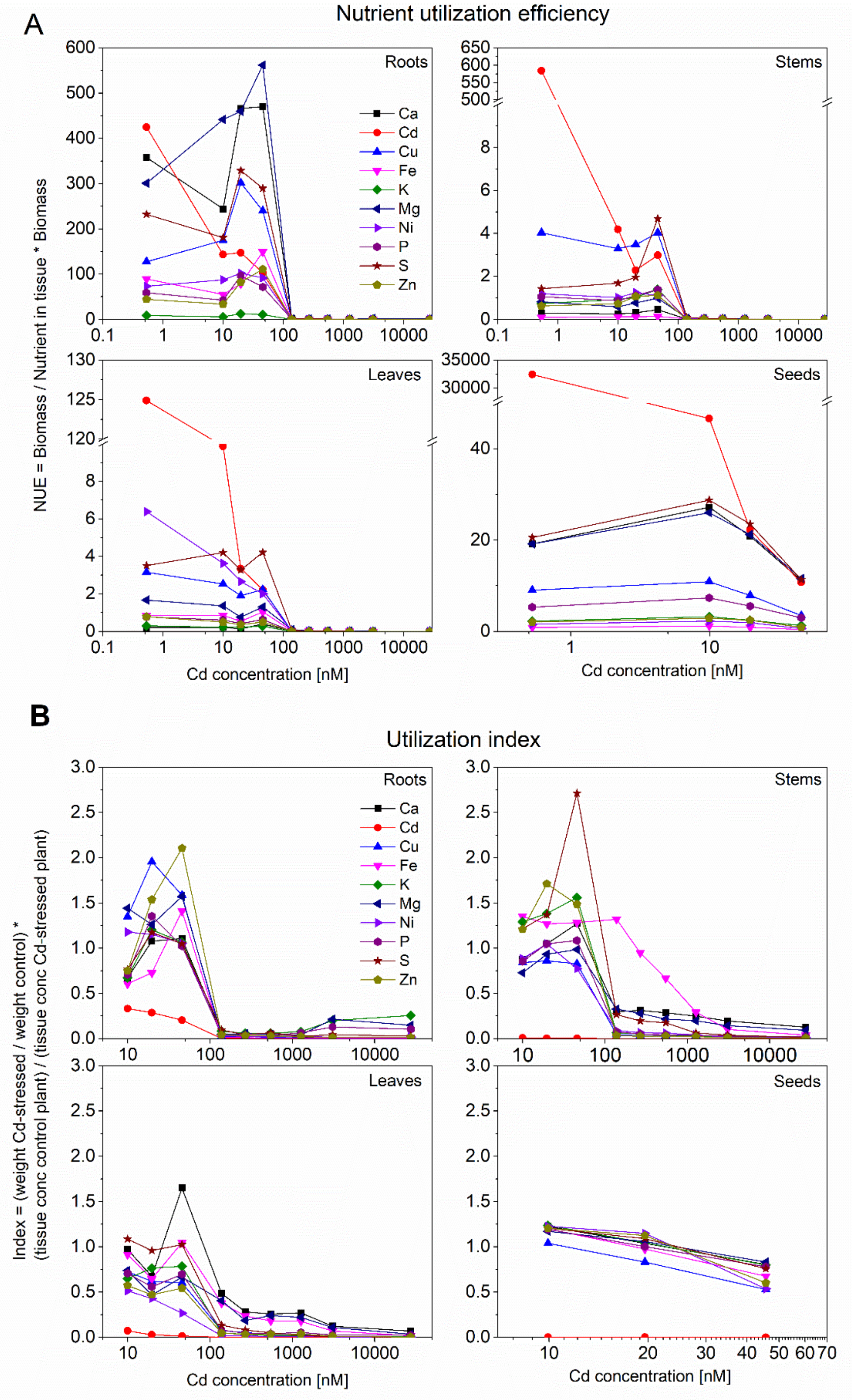

Figure S2 Acetone extracts from soybean leaves exposed to low, medium and high but sublethal Cd concentrations for 10 weeks with focus on the Chl molecules.

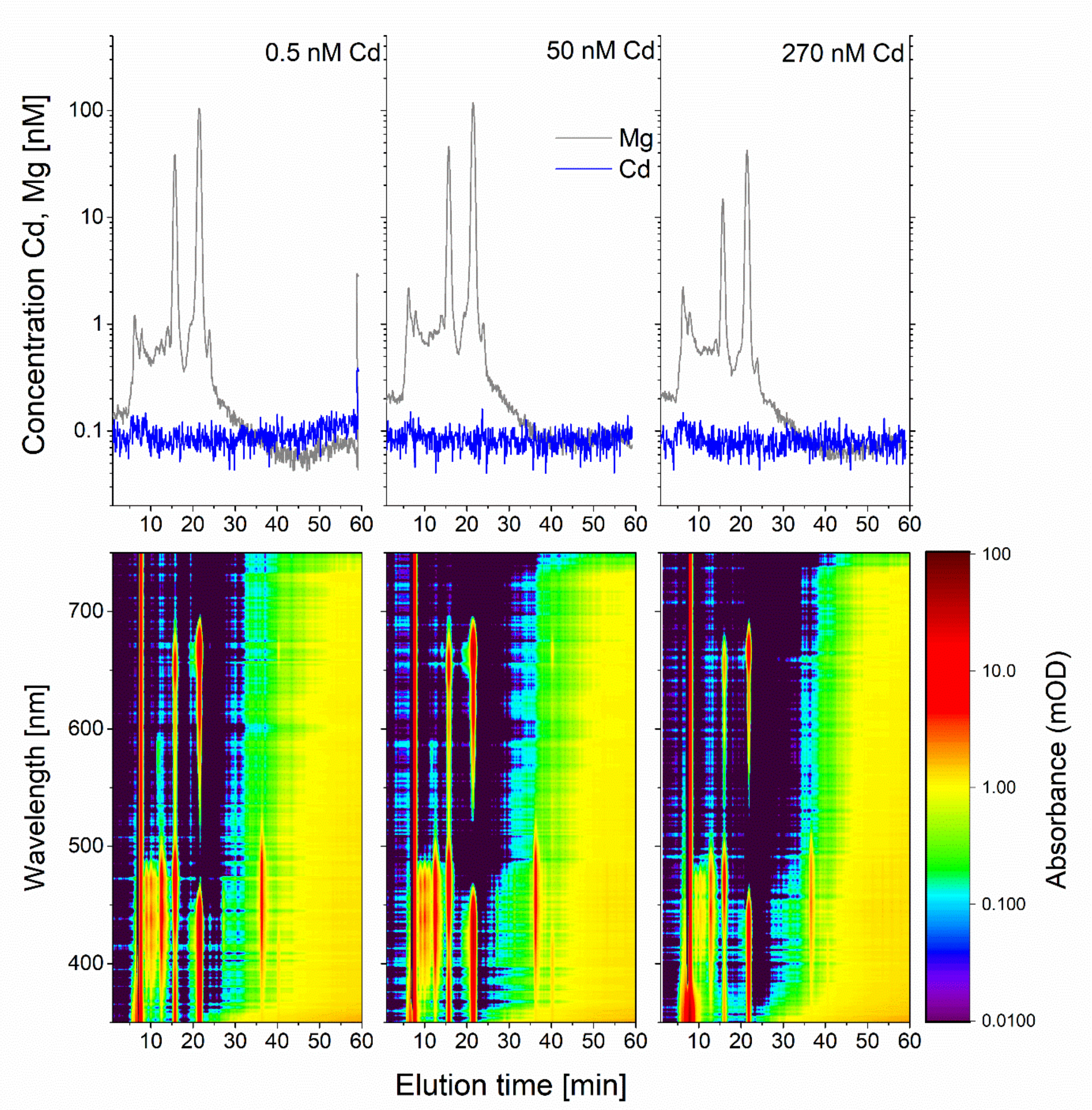

*

Figure S3. Effect of Cd exposure on membrane proteins of single soybean leaves after 5 weeks of exposure. ICP-MS count rates and protein chromatographs were normalized to the Co signal of added Vit B12. Chromatographs from one of two experiments are shown exemplarily.

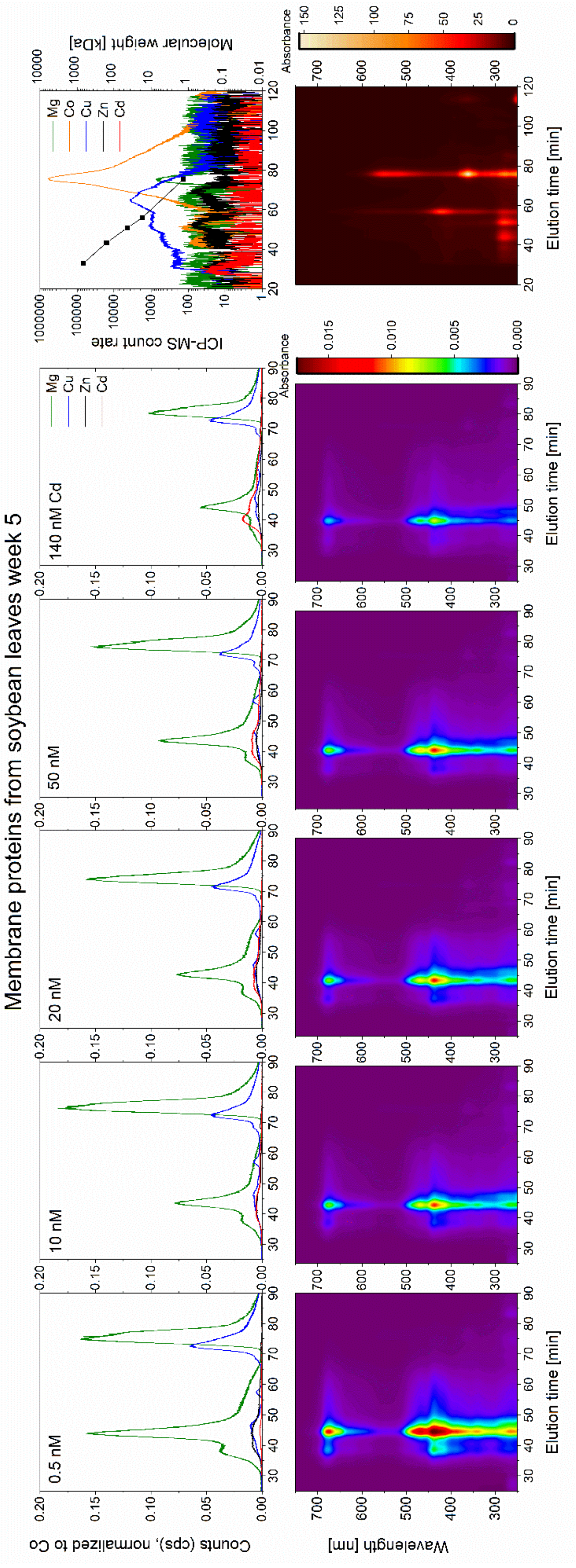

Figure S4. XANES of sample 2 of the purified LHC II from Cd-exposed soybean leaves.

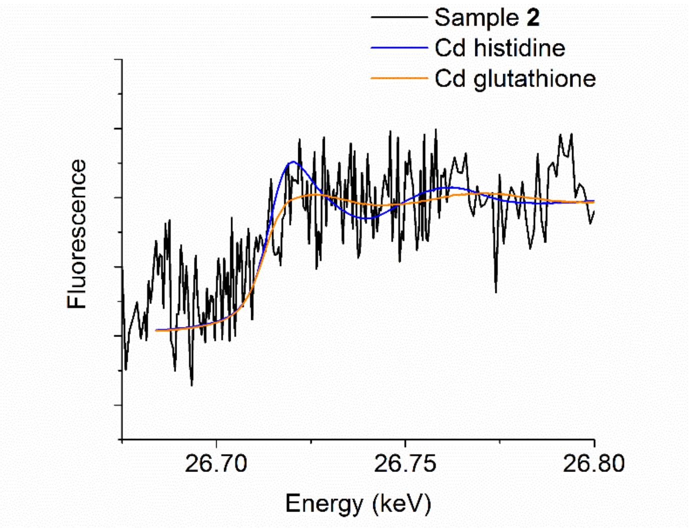

Figure S5: A. Concentration of Mn in the hydroponic nutrient solution in the barrels (n = 4) and in the pots after 5 (n=2) and 10 (n=4) weeks of treatment duration. Values are mean of 4 experiments and error bars represent the standard error of the mean (SE).**B.** Accumulation of Mn in different tissues of plants after 5 weeks (leaves only) and 10 weeks of exposure to various Cd concentrations. Values are mean from independent 4 experiments and up to 4 individual plants per measurement (n=16) for leaves harvest, stems and seeds. w5 and w10 leaves n=8. Error bars represent the SE.

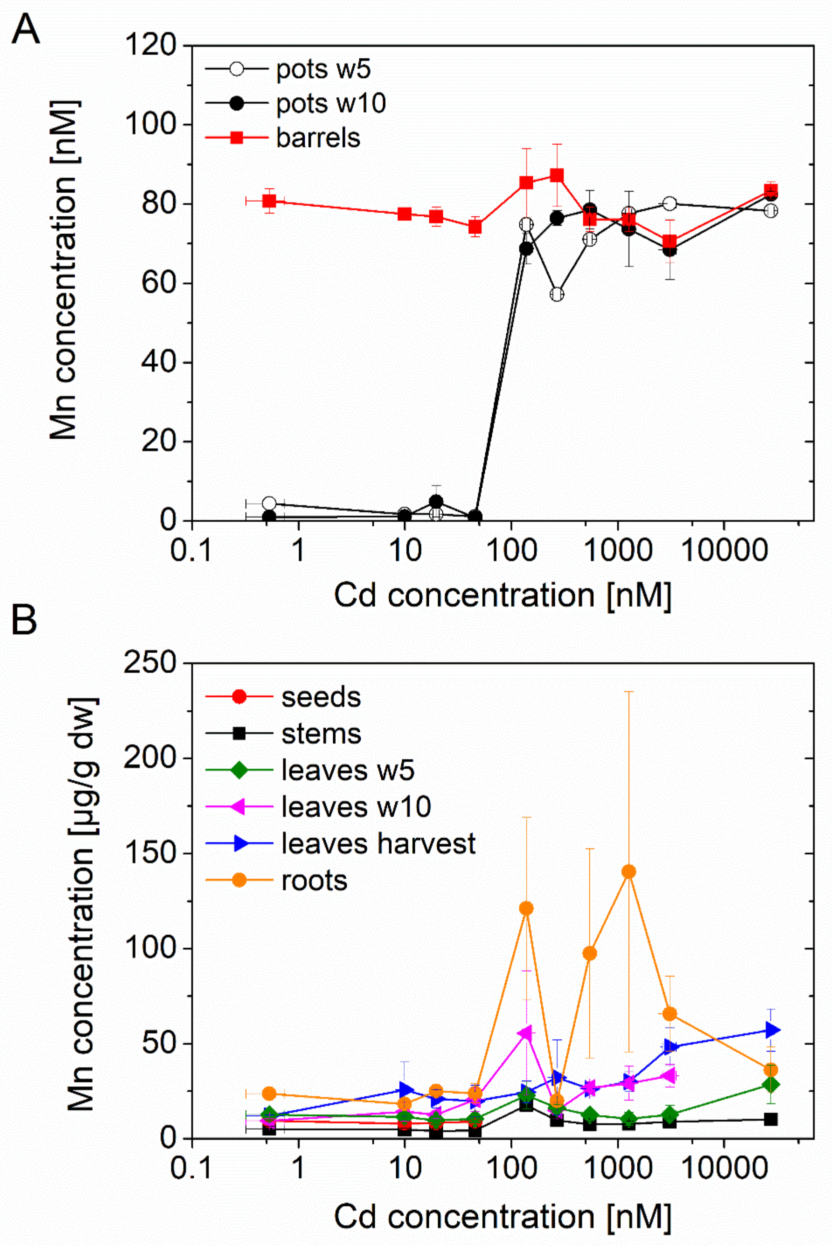

Protocol S1: Protein identification

Mass spectrometry analysis of protein

The protein in solution were reduced, alkylated and digested with trypsin. The sample was vacuum-dried and re-dissolved in 20 µl 50 mM ammonium bicarbonate (AMBI). Then the samples were reduced with dithioerytritol (DTT) in 25 mM AMBI up to a final concentration 10 mM for 60 min at 37ºC and subsequently alkylated with iodoacetamide in 25 mM ammonium bicarbonate up to 55 mM final concentration for 30 min in the dark. Finally, samples were digested with recombinant trypsin (Roche Molecular Biochemicals) in 25 mM AMBI (pH 8.5) in a trypsin/protein-ratio of 1/20, overnight at 37ºC.

After digestion, the peptides were desalted with a C18 ZipTiP® (Millipore) according to the manufacturer's instructions and eluted in 6 µl/l of 50% acetonitrile and 0.1% TFA, and 1 µl was spotted in a dried droplet application onto a MALDI plate.

MALDI-TOF MS analyses were performed in a 4800 plus Proteomics Analyzer MALDI-TOF/TOF mass spectrometer (Applied Biosystems, Framingham, MA) at the Proteomic Unit of Complutense University of Madrid. Maldi-TOF/TOF was operated in positive reflector mode with an accelerating voltage of 20000 V.

All mass spectra were calibrated internally using peptides from the auto digestion of trypsin.

The analysis by MALDI-TOF/TOF mass spectrometry produces peptide mass fingerprints and the peptides observed with a signal to noise greater than 12 can be collated and represented as a list of monoisotopic molecular weights.

Proteins ambiguously identified by peptide mass fingerprints were subjected to MS/MS sequencing analyses using the 4800 Proteomics Analyzer (Applied Biosystems, Framingham, MA).

Suitable precursors from the MS spectra were selected for MS/MS analyses by Colision Induced Disociation (CID) using atmospheric gas and 1 Kv ion reflector operating mode. The precursor mass windows isolation was +/- 4 Da. The plate model and default calibration were optimized for the MS-MS spectra processing.

For protein identification, the NCBI protein database (date: 20171024; 135744157 sequences) was used. Searches without taxonomy restriction were done using MASCOT 2.3 (www.matrixscience.com). Search parameters were:

- Carbamidomethyl cysteine as fixed modification and oxidized methionine as variable modification

- Peptide mass tolerance 50-100 ppm
- 1 missed trypsin cleavage site
- MS-MS fragments tolerance 0.3 Da

The parameters for the combined search (Peak list from Peptide mass fingerprint and MS-MS spectra) were the same as described above. In all protein identification the probability scores were greater than the score fixed by Mascot as significant with a p-value minor than 0.05.

Proteins that were not identified by Mascot database searching were subsequently subjected to *de novo* sequencing analyses, based on the fragmentation spectra of peptides, using DeNovo tool software (Applied Biosystems), tentative sequences were manually checked and validated. Homology search of the sequences was obtained by Blast (http://www.ncbi.nlm.nih.gov/BLAST)

Protocol S2: Metabolomics

Sample extraction

A slightly modified version of the protocol described by James *et. al.* (DOI: 10.1007/s11306-016-0956-2) was used to extract the metabolites. In short, the extraction consists of a beadbeating step and liquid-liqiud extraction using chloroform, ultrapure water and methanol. The aqueous and organic phases are collected and dried. The dried organic phase has been used for the lipidomics analysis (see below) and the dried aqueous phase for all other analysis (after reconstituting in 200 µL ultra-pure water).

Quality control samples

For quality control, a mixed pooled sample (QC sample) was created by taking a small aliquot from each sample. This sample was analysed with regular intervals throughout the sequence. Matrix effects have been tested for quantified compounds by spiking the QC sample in a minimum of two levels.

Target LC

For the detection of NADPH, AMP, ADP, ATP, Acetyl-CoA and Glyceraldehyde-3-phosphate a targeted LC-method were applied. The method is using a zwitterionic HILIC column and is based on the method described by West *et al.* (DOI 10.1007/s11306-016-0956-2). Besides from the compounds mentioned above also a range of sugars were extracted from the data obtained from this analysis.

LC-metabolites

Sample preparation

Samples reconstituted samples were diluted 5 times in eluent *A prior* to analysis.

Data processing

The data was analysed using both a targeted and an untargeted approach. The targeted approach was used to extract the response of compounds included in the standard list, which covered the 142 compounds listed below.

Glycine, Valine, Leucine, Isoleucine, Threonine, Proline, Asparagine, Glutamine, Aspartate, Serine, Glutamic acid, Methionine, Phenylalanine, Ornithine, Lysine, Tyrosine, Tryptophan,

Alanine, Arginine, Cysteine.

Pyruvic acid, Fumaric acid, Succinic acid, Lactic acid, Malic acid, 2-oxoglutarate, Phosphoenolpyruvate, cis aconitic acid, Citric acid, Isocitric acid, Hexadecanoic acid (C16:0), Octadecanoic acid (C18:0), Malonic acid, Salicylic acid, beta-Alanine, GABA, Acetylcarnitine (C2), Acetylmuramic acid, Carnitine, Cytidine, KAPA, Muramic acid, Nicotine amide, Propionylcarnitine (C3), Pyridoxamine, Spermidine, Thymine, Riboluse-5-phosphate, 3-Hydroxybutyric acid, Hexose, Pyroglutamic acid, Sorbitol/Manitol, Uridine

5-deoxyadenosine, Adenine, Adenosine, AMP, ADP, ATP, NADPH, Glucose-x-phosphate, cAMP, Glyceraldehyde-3-phosphate, Acetyl-CoA, Glucose/galactose/fructose, Arabitol, Arabinose, Maltose/trehalose/sucrose/melibiose/turanose.

For the untargeted approach feature extraction was conducted using mzMine. A feature is a peak characterized by a mass and a retention time. Since many compounds gives rise to a signal in more than one mass trace (e.g. naturally occurring C13 isotopes, adducts, and fragments) a compound will almost always be represented by more than one feature with the same retention time but different masses.

Features from mzMine will be identified with Xiiii (positive ionization) and Yiiii (negative ionization), where iiii is a number. These features have been tentatively annotated by a library search of the masses in the plantCyc database with a mass uncertainty of 0.0005 Da or 1 ppm. The search in plantCyc is assuming that all ions are originating from the [M+H]+ or [M+Na]+ (in positive ionization) or the [M-H]- (in negative ionisation) ions. Notice that the annotations are only based on the masses and therefore they are subject to a much higher uncertainty than the identified compounds. A more certain identification could be done by either MS/MS analysis of the compounds in question or by analysing authentic standards.

GC-metabolites

Gas Chromatography – Mass Spectrometry (GC-MS) is a widely applied analytical tool in metabolomics. Due to its high separation power, its capacity for reliable identification of hundreds of metabolites and its low cost, GC-MS is often the first choice for metabolite analysis. However, GC-MS systems is limited to detect volatile compounds and, consequently, chemical derivatization of non-volatile compounds is required. The GC-metabolites method converts amino and non-amino organic acids into volatile carbamates and esters.

Sample preparation

Samples (see above) have been derivatised using MCF (methyl chloroformate).

Data processing

The large amount of raw GC-MS data is processed by software developed by MS-Omics and collaborators. The software uses the powerful PARAFAC2 model and can extract more compounds and cleaner MS spectra than most other GC-MS software (see their homepage www.msomics.com for more information).

Protocol S3: Lipidomics

Metabolites were extracted as above. The aqueous and organic phases were collected and dried. The dried organic phase has been is used for the lipidomics analysis and the dried aqueous phase for all other analysis. Samples were reconstituted in an isopropanol / acetontril / water-mixture.

Data were processed using Compound Discoverer 3.0 (ThermoFisher Scientific). First, features were extracted from the raw data. One compound often give rise to a signal in more than one mass trace (due to e.g. naturally occurring C13 isotopes, adducts, and/or fragments), a compound will therefore almost always be represented by more than one feature with the same retention time but different masses. The feature detection was followed by grouping of features belonging to the same compound. This additional information (e.g. isotope pattern) was then used together with the accurate mass to determine the molecular formula.

Lipids clearly not belonging to the plant kingdom were omitted from the analyses.

The total information collected for each compound were then used in the following identification step. For these data there are generally three levels of annotation:

Level 1: Annotations on this level are the most secure identifications. They are based on three pieces of information: accurate mass, MSMS spectra and known retention time obtained from standards analysed on the same system.

Leve 2: Annotations on this level is based on two pieces of information; either accurate mass and MSMS spectra or accurate mass and known retention time as obtained from standards analysed on the same system.

Level 3: Annotations on this level is based on library searches using the accurate mass and elemental composition alone. Be aware that annotations on this level should be used with care, as more than one elemental composition could be matched with the same accurate mass, even with the high accuracy on the instruments we use, and it is impossible to distinguish between isomers on this annotation level.

For unidentified compounds the elemental composition is determined, if a proper match is found between the accurate mass obtained and the isotope pattern.

Accurate mass refers to a deviation on +/- 0.0005 Da. The annotations on level 3 are based on searches in LipidBlast library, which covers these 29 lipid classes:

Cardiolipins (CL), Ceramide-1-phosphates (CerP), Cholesteryl esters (CE), Diacylated

phosphatidylinositol, dimannoside (Ac2PIM2), Diacylated phosphatidylinositol monomannoside (Ac2PIM1), Diacylglycerols (DG), Digalactosyldiacylglycerols (DGDG), Diphosphorylated hexaacyl Lipid A (LipidA-PP), Gangliosides ([glycan]-Cer), Lysophosphatidic acids (lysoPA), Lysophosphatidylcholines (lysoPC), Lysophosphatidylethanolamines (lysoPE), Monoacylglycerols (MG), Monogalactosyldiacylglycerols (MGDG), N-acylsphingosines (ceramides) (Cer-d), Phosphatidic acids (PA), Phosphatidylcholines (PC), Phosphatidylethanolamines (PE), Phosphatidylglycerols (PG), Phosphatidylinositols (PI), Phosphatidylserines (PS), Plasmenylphosphatidylcholines (plasmenyl-PC), Plasmenylphosphatidylethanolamines (plasmenyl-PE), Sphingomyelines (SM), Sulfatides (ST), Sulfoquinovosyldiacylglycerols (SQDG), Tetraacylated phosphatidylinositol dimannoside (Ac4PIM2), Triacylated phosphatidylinositol dimannoside (Ac3PIM2), Triacylglycerols (TG).

Please note: Analyses of lipids were done without lipid standards and identities determined on basis of MSMS level only, which can lead to mis-identifications.
